# Genes encoding hub and bottleneck enzymes of the *Arabidopsis *metabolic network preferentially retain homeologs through whole genome duplication

**DOI:** 10.1186/1471-2148-10-145

**Published:** 2010-05-18

**Authors:** Xudong Wu, Xiaoquan Qi

**Affiliations:** 1 Key Laboratory of Photosynthesis and Environmental Molecular Physiology, the Institute of Botany, Chinese Academy of Sciences, Beijing 100093, China

## Abstract

**Background:**

Whole genome duplication (WGD) occurs widely in angiosperm evolution. It raises the intriguing question of how interacting networks of genes cope with this dramatic evolutionary event.

**Results:**

In study of the *Arabidopsis *metabolic network, we assigned each enzyme (node) with topological centralities (in-degree, out-degree and between-ness) to measure quantitatively their centralities in the network. The *Arabidopsis *metabolic network is highly modular and separated into 11 interconnected modules, which correspond well to the functional metabolic pathways. The enzymes with higher in-out degree and between-ness (defined as hub and bottleneck enzymes, respectively) tend to be more conserved and preferentially retain homeologs after WGD. Moreover, the simultaneous retention of homeologs encoding enzymes which catalyze consecutive steps in a pathway is highly favored and easily achieved, and enzyme-enzyme interactions contribute to the retention of one-third of WGD enzymes.

**Conclusions:**

Our analyses indicate that the hub and bottleneck enzymes of metabolic network obtain great benefits from WGD, and this event grants clear evolutionary advantages in adaptation to different environments.

## Background

Whole genome duplication is one of the most important evolutionary events in plants [1] and many duplicated genes retained as large blocks have been found in the *Arabidopsis *[2], rice [3,4] and *Populus *[5] genomes. In *Arabidopsis*, a genome-wide similarities search (Blast) was done among protein-coding genes and strong evidence for a whole genome duplication event was demonstrated by phylogenetic analysis. The analysis of the genomic duplication blocks revealed that about 80 percent of genes lost their sister genes following WGD [2,6]. In rice, analysis using the structural genomic data and phylogenetic analysis suggested that a polyploidization event occurred about 50~70 million years ago, which was before the divergence of the major cereals but after the divergence of the *Poales *from the *Liliales *and *Zingiberales *[3,4]. Analysis of the assembled *Populus *genome sequences revealed evidence for a whole-genome duplication event in the genome, where about 8000 pairs of homeologs survived after the event [5].

Research on the genome data from vertebrates and yeast has shown that factors, such as gene expression intensity [7,8], protein interaction [9], phylogenetic age [10], and dosage sensitivity [11] have influenced the evolutionary rates or the retention of homeologs after gene duplication. Recent research on the unicellular ciliate protozoa, *Paramecium *[12], showed that metabolic genes appear more retained than other types of genes after WGD. In *Arabidopsis*, dosage effects were suggested to be an important factor influencing the retention of homeologs. For example, transcription factors, which have roles in regulating other genes, normally show strong dosage sensitivity and genes encoding transcription factors were over-represented amongst WGD-homeologs as shown by GO analysis [11]. Although plants have evolved the ability to synthesize a vast array of metabolites which are essential for adaptation to diverse natural environments [13-15], the evolution of plant metabolic networks has not been studied extensively. It is of particular interest to know how plant metabolic networks cope with whole genome duplication events.

**Figure 1:**
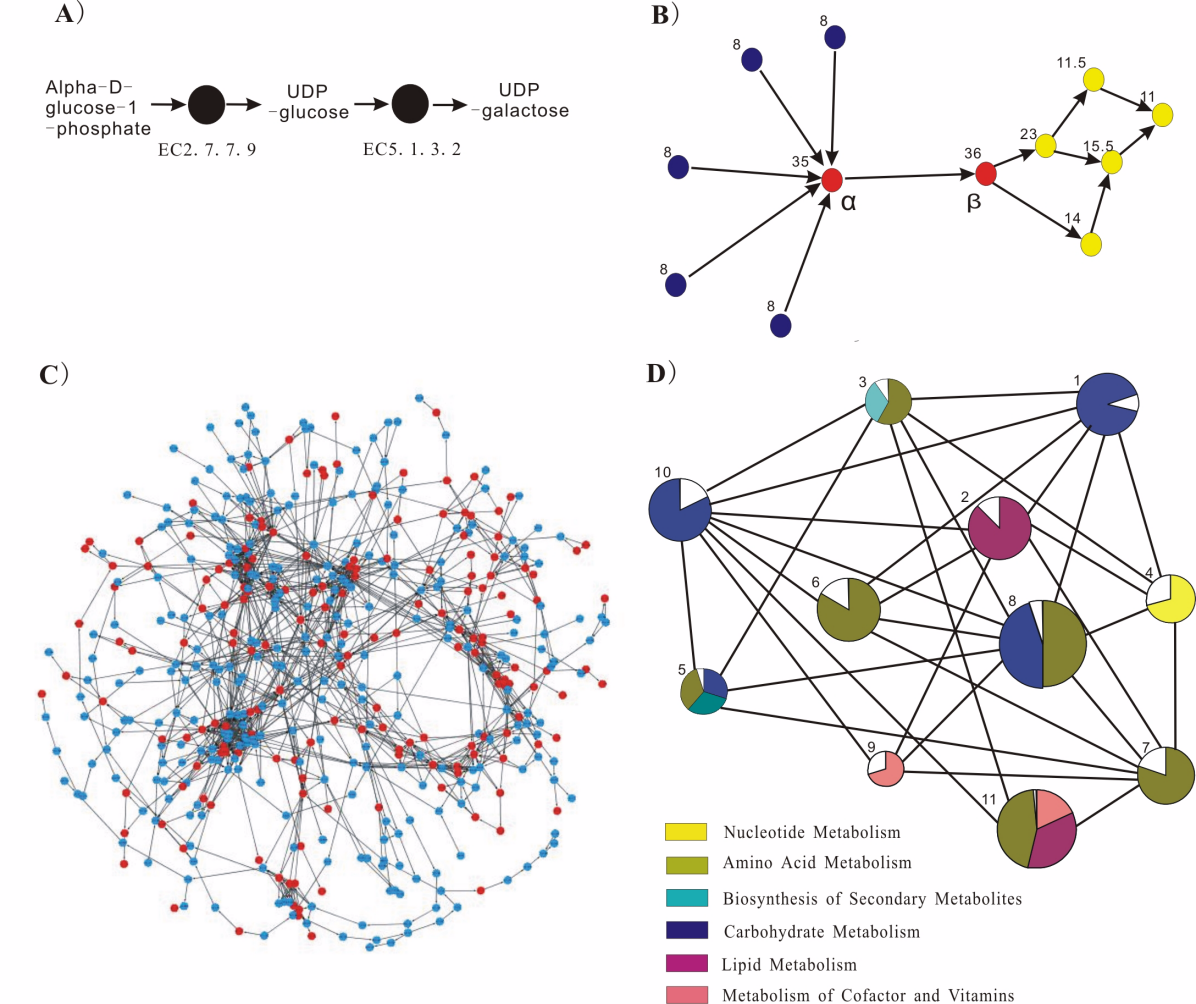
**The *Arabidopsis *enzyme-enzyme metabolic network**. (*A*) The interaction between two enzymes. Dots represent enzymes, directional edges represent metabolite flux. (*B*) Diagram showing in-degree, out-degree and bottlenecks. The in-degree of node α is 5 whereas its out-degree is 1, node α and node β are bottlenecks. The between-ness shown on the top left of each nodes. (*C*) The enzyme-enzyme network and the distribution of 173 WGD-enzymes. Dots represent enzymes and the lines represent the interactions among enzymes. Dots highlighted by red represent the WGD-enzymes. (*D*) Cartography of *Arabidopsis *metabolic network. The circles represent modules (1 to 11) and the lines reflect the connections among modules. The area of color in each module is proportional to the number of enzymes that belong to the corresponding functional pathways. The blanks represent the enzymes whose corresponding pathways are not represented in the module.

Graph theory provides paradigms to study networks [16]. The plant metabolic network is the well-known biological network [17]. Its enzymes can be represented by nodes and substrate-product metabolite flux can be represented by directional edges (as demonstrated in Figure 1A). Three topological centralities are used to measure the importance of nodes in the control of information transfer. In-degree refers to the number of links forwarded to the considered nodes, out-degree refers to the number of links outwards from the considered nodes and between-ness measures the propensity of shortest paths from any other nodes going through a certain node. In Figure 1B, five enzymes provide products for node α, which produces one product for the next biosynthesis step (node β), so the in-degree of node α is assigned by 5 whereas its out-degree by 1. Node α and node β are the essential nodes for successful information transfer from the blue nodes to the yellow nodes, if either of them is knocked out, the network would collapse. Obviously, in-degree and out-degree only consider the partners connected directly to any particular node, whereas between-ness considers a node's position in the network. Usually nodes with relatively higher degrees are termed hubs (see example, node α in Figure 1B) and nodes with higher between-ness are named bottlenecks [18] (see examples, node α and node β in Figure 1B).

In this study, we reconstructed the *Arabidopsis *metabolic network according to the recently updated Aracyc data (biochemical pathway database for *Arabidopsis thaliana*)[19]. By using graph theory for the analysis of the metabolic network, each enzyme was assigned with topological centralities (in-degree, out-degree and between-ness) to measure quantitatively its importance in the network. The observation that homeologs retained following WGD preferentially encode hub-bottleneck enzymes, provides us a first view of the relationship between metabolic networks and the retention of WGD-homeologs in *Arabidopsis*.

## Results

### Hubs and bottlenecks tend to express highly and evolve conservatively

The *Arabidopsis *enzyme-enzyme metabolic network was constructed using the recently updated Aracyc database [19] (see Methods). We retrieved 1785 directional interactions among 496 enzymes, of which 478 enzymes are included in a large network (Additional file 1) and the other 18 enzymes in 7 small clusters. The large connected network contains 1015 directional interactions and 379 bi-directional interactions (Figure 1C). In directional interaction, metabolite is the substrate or product of particular enzyme, whereas in bi-directional interaction, metabolite can be used as substrate as well as product by the same enzyme. The large connect network is highly modular and the 11 separated modules correspond to functional pathways (Figure 1D) (see Methods for modular analysis and Additional file 2 for description of 11 modules), confirming that the topological analysis of enzymes can reasonably reveal their functional status in the network. Topologically, the highly modular structure of the *Arabidopsis *metabolic network indicates the existence of bottleneck enzymes, which tend to connect different modules/pathways. Between-ness (see Methods), which refers to the centrality of the considered enzymes in control of substrate-product fluxes in the network, was used to quantify this propensity.

A previous study [20] showed that genes encoding enzymes in the same pathway tend to co-express and core-metabolic pathways display tighter levels of transcriptional coordination, but the relationship between the mRNA transcription profiles of genes encoding enzymes and their importance in the metabolic network remains unknown. We did a correlation analysis between the expression parameters (maximum intensity, expression variation, see Methods and Additional file 3) and topological centralities (in-degree, out-degree, between-ness) of enzymes. Spearman correlation analysis showed that the in-degree, out-degree and between-ness positively correlate with expression intensity (Table 1). This indicates that genes encoding the hub and bottleneck enzymes tend to express with higher intensity.

**Table 1:** The correlations between three topological centralities and the expression intensity, variation.

	**Max Intensity in developmental stages**		**Max Intensity in shoot after stress**		**Variation in developmental stages**	
	**rho**	**Q value^#^**	**rho**	**Q value**	**rho**	**Q value**
	
In-degree	0.155	3.96E-04**	0.151	4.08E-04**	0.026	0.11
Out-degree	0.183	6.47E-05**	0.173	1.19E-04**	0.039	0.08
Betweenness	0.130	1.81E-03**	0.144	3.08E-04**	0.005	0.16

^# ^The q-values were obtained by the FDR correction of p values from spearman correlation analysis, 2-tailed.

We further explored whether genes with higher in-degree, out-degree and between-ness evolve conservatively in orthologous gene pairs between *Arabidopsis *and *Populus*. The non-synonymous substitution rate, *Ka *was used to measure the evolutionary rate of the coding region (see Methods, Additional files 4 &5). By correlating the average *Ka *of genes with their topological centralities, we found that the in/out-degree, out-degree and between-ness negatively correlate with the average *Ka*. Also, the in-degree and between-ness negatively correlate with the average substitution rate of the 5' upstream and 3' downstream 1000-bp regions (Table 2). These results show that the genes encoding hub and bottleneck enzymes tend to be more conservative in their coding, 5'upstream and 3'downstream regions. Taken together, genes encoding hubs and bottleneck enzymes tend to express highly and evolve conservatively.

**Table 2:** The correlations between three topological centralities and substitution rates in coding or 5' upstream, 3'downstream 1000 bp regions.

	**Non-synonymous coding rate (n = 414)**		**3' downstream 1000 bp region (n = 378)**		**5' upstream 1000 bp region (n = 395)**	
	**rho**	**Q value^#^**	**rho**	**Q value**	**rho**	**Q value**
	
In-degree	-0.201	6.47E-05**	-0.110	0.007**	-0.161	5.93E-04**
Out-degree	-0.177	6.47E-05**	-0.066	0.041*	-0.082	0.026*
Betweenness	-0.155	5.93E-04**	-0.101	0.011*	-0.138	0.002**

^# ^The q-values were obtained by the FDR correction of p values from spearman correlation analysis, 2-tailed.

### Hub and bottleneck enzymes prefer to retain homeologs through WGD

**Figure 2:**
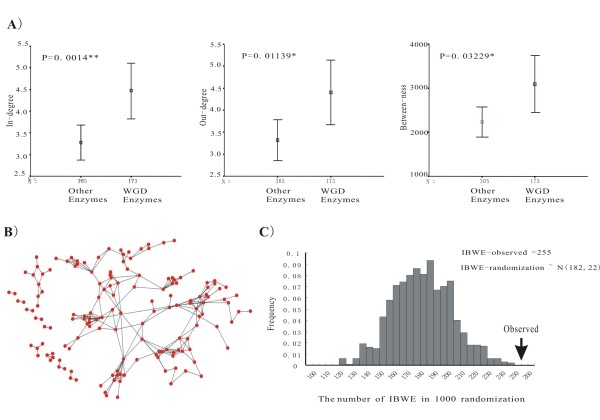
**Analysis of WGD-enzymes in metabolic network**. (*A*) Comparison of three topological centralities between WGD-enzymes and the other enzymes. (*B*) Sub-network of WGD-enzymes. Dots represent enzymes and the lines represent the interactions among enzymes. (*C*) The distribution of IBWEs in 1000 randomization simulations. The arrow on the right hand side represents the observed number of IBWE.

The reconstructed *Arabidopsis *metabolic network enabled us to investigate the relationship between the retention of enzyme-homeologs through WGD and their centralities in the metabolic network. We identified enzymes as WGD-enzymes if these enzymes have at least one pair of homeologs, which were retained through the processes of gene gains and losses following WGD. The dataset of homeologs generated by WGD was retrieved from the *Arabidopsis *polyploidy database http://Wolfe.gen.tcd.ie/athal/dup, and genes encoding 173 WGD-enzymes were identified (Additional file 6). Comparisons of in-degree, out-degree and between-ness distributions of WGD-enzymes with those of other enzymes show that the WGD-enzymes have significantly higher in in-degree, out-degree and between-ness scores (Figure 2A), indicating that the WGD-enzymes are preferentially located in hub and bottleneck positions of the network. In other words, genes encoding hub and bottleneck enzymes are preferentially retained as homeologs through WGD.

Previous research [21,22] indicated that more ancient enzymes tend to have higher connectivity. So, we investigated whether the observed enrichment of WGD-enzymes involved as hubs and bottlenecks in *Arabidopsis *metabolic network was due to their phylogenetic ages. Of the 173 WGD-enzymes in *Arabidopsis*, 162 were found to have at least one encoding-genes involved in *Arabidopsis*-*Populus *(Additional file 4) or *Arabidopsis-*rice ortholog groups (Additional file 5). Of the other 305 non-WGD enzymes, 281 were found to have at least one encoding genes involved in *Arabidopsis*-*Populus *or *Arabidopsis-*rice ortholog groups. *Chi-*square test showed that WGD-enzymes and non-WGD enzymes were not different in phylogenetic age (162/173 vs. 281/305, *2-sided, p = 0.59*). Since the genome duplication event in *Arabidopsis *occurred 20-40 million years ago [2], significantly later than the split of *Arabidopsis *and *Populus *[5], the significant differences in connectivity (in-degree, out-degree and between-ness) among the metabolic enzymes must have already existed before the genome duplication event in *Arabidopsis*. The enrichment of WGD-enzymes in the hubs and bottlenecks in the metabolic network were not significantly influenced by the phylogenetic ages.

Plant genomes contain significant numbers of tandem duplicate genes. We also tested whether tandem duplicate genes tended to encode hub and bottleneck enzymes. The subset of tandem duplicates genes in *Arabidopsis *was retrieved from the TIGR database, http://www.tigr.org/tdb/e2k1/ath1/TandemDups/TandemGenes.html (criteria: e-value < = 1e-20, only one unrelated gene was allowed to be interspersed within a cluster of tandem duplicated genes, ~2500 genes were identified). Of the 478 metabolic network enzymes, we identified genes encoding 25 metabolic enzymes which retained tandem-homeologs (named by tandem-enzymes, see Additional file 7). The comparison showed that the tandem-enzymes and the other enzymes have no significant difference in the in-degree, out-degree and between-ness (Additional file 8). So gene families encoding hub and bottleneck enzymes are not preferentially enlarged in their copy number by tandem duplication.

### WGD-enzymes tend to catalyze consecutive steps

**Figure 3:**
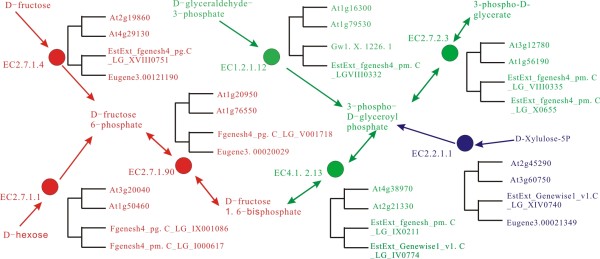
**A sub-network contains seven *Arabidopsis/Populus *WGD-enzymes which locate in the three inter-connected pathways**. The fructose metabolism, gluconeogenesis and the pentose phosphate pathways were marked by red, green blue fonts, respectively.

Random simulation was adopted to estimate the impact of preferential retention of WGD homeologs in hubs and bottlenecks. The identified 173 WGD-enzymes were used to retrieve the connected WGD-enzymes, and 255 interactions (named by interaction between WGD-enzymes, IBWE) were obtained (Figure 2B). Simulation analysis involved two steps, (a) the 173 enzymes were randomly assigned in the network, (b) edges connecting two selected enzymes were marked and the number of edges *k *was recorded. Then steps from (a) to (b) were repeated 10000 times, and resulted in a normal distribution *N (182, 22) *of *k *(Figure 2C). The observed number of 255 interactions obtained from the WGD-enzymes significantly deviated from random expectation (Z-score = (255-183)/22 = 3.27, p < 0.01). This indicated that WGD-enzymes tend to be connected, revealing that they tend to catalyze consecutive steps in the pathways (see example in Figure 3).

The simulation analysis indicated the retention of genes encoding enzyme-homeologs was not independent of the retention of the genes encoding their interacting enzyme-homeologs. For a metabolic interaction *E*_1_->*E*_2_, after the whole genome duplication, the retention of *E*_1 _could influenced by the retention of *E*_2_, or vice versa. Obviously, some interactions promote the simultaneous retention of connected enzymes and could significantly increase the number of IBWE. The number of this type of interactions followed a normal distribution because 255-*N(182,22) *= *N(72,22) *(see Figure 2C). To investigate how many WGD-enzymes are due to this type of interaction, we first (a), removed the information of WGD-enzymes in the network. Then a number, *m*, were drawn from a normal distribution *N (72, 22)*. (b) By randomly assigning *m *edges to the network, nodes connected by the selected edges were marked, and then the number of nodes, *f*, was recorded. Repeating two steps from (a) to (b) 10000 times, *f *followed a normal distribution as *N(62, 16)*, This indicates that this type of interactions render about 62 enzymes to be WGD-enzymes with the standard error of 16. In total, approximately one-third (62/173) of the WGD-enzymes were attributable to the impact of metabolic interaction.

We applied comparative genomics to explore whether the interactions connected by WGD enzymes in *Arabidopsis *were also likely connected by WGD enzymes in *Populus*. The datasets of *Populus *enzymes from http://genome.jgi-psf.org/Poptr1_1/, and the *Populus *WGD-homeologs were retrieved from http://chibba.agtec.uga.edu/duplication/[23]. Our analysis identified *Populus *genes encoding 226 enzymes which retain at least one pair of WGD-homeologs (Additional file 9). Among the 1394 *Arabidopsis *enzyme-enzyme interactions, 255 interactions were identified to be connected by *Arabidopsis *WGD enzymes (IBWE, Figure 2B), while 68 ortholog interactions (the interactions between orthologs in both *Arabidopsis *and *Populus*) were found to be connected by WGD-enzymes in *Populus*. Of the 255 *Arabidopsis *IBWE, 32 ortholog interactions also are connected by WGD-enzymes in *Populus*. (see the example, Figure 3). The propensity (12%, 32/255) is significantly higher than the expected value (5%, 68/1394) (*p < 0.01, two-tailed, chi-square test*), indicating that the interactions connected by WGD-enzymes in *Arabidopsis *indeed also tend to be connected by WGD-enzymes in *Populus*.

Figure 3 demonstrated a sub-network containing seven enzymes from the 32 IBWEs in both *Arabidopsis *and *Populus*. The seven enzymes locate in the inter-connected positions of three pathways, the fructose metabolism, gluconeogenesis and the pentose phosphate pathways. Phylogenetic analysis revealed that the seven genes were preferentially retained as parallel paralogs in the process of WGD after the two species split (see Methods). Genes encoding these important enzymes were simultaneously duplicated in the process of WGD, hence metabolic flux has simultaneously increased, maintaining the balance of the metabolite flux.

Finally, in the process of WGD, genes encoding hub and bottleneck enzyme can be easily retained their homeologs, providing not only one extra copy of individual enzymes but also another set of interconnected enzymes for the consecutive steps in the pathways.

## Discussion

The gain/loss of homeologs was an important event in the evolution of the plant genome. Previous analysis in *Arabidopsis *[11] showed that some chromosome islands of retention contain 'connected genes' following genome duplication. Those genes are mainly from families encoding components of the proteasome/protein modification complexes, signal transduction machinery, ribosomes and transcription factor complexes. Our analysis indicated that genes encoding hub and bottleneck enzymes in the *Arabidopsis *metabolic network tend to be highly expressed and more conserved. This results support the previous observations that highly expressed genes evolve slowly in yeast [8] and genes that have a lower propensity to be lost in the evolution accumulate fewer substitutions in their protein sequences and tend to be essential for the organism viability, tend to be highly expressed, and have many interacted proteins based on the analysis of the vertebrate genomes [24].

Our further analysis revealed that genes encoding hub and bottleneck enzymes in the *Arabidopsis *metabolic network tend to preferentially retain homeologs after WGD and the simultaneous retention of WGD-homeologs encoding enzymes which catalyze consecutive steps in a pathway is highly favored. This phenomena can be best explained by the dosage-sensitive relationship in the gene balance hypothesis which has been demonstrated in yeast and humans [25], maize and *Drosophila *[26] and also by the theoretical prediction [27]. In brief, this hypothesis presumes that after long term evolution, "connected genes" of multisubunit complexes in the present genomes have been in an optimum balance status and changes of the individual genes in the subunit would display dosage sensitivity, resulting in out-of-balance phenotypes which have disadvantages in fitness in the evolution [28-30]. In the *Arabidopsis *metabolic network, genes encoding hub enzymes are in the important positions and usually connected with many other enzymes, forming a sub-network, and are very likely to be more sensitive to the dosage effects and indeed preferentially retained after WGD.

The simulation analysis revealed that about one-third of the WGD-enzymes were attributable to the impact of metabolic interaction, the comparative genomics analysis demonstrated that 12% of interactions connected by WGD-enzymes in *Arabidopsis *are connected by WGD-enzymes in *Populus*. The results indicate that genes involving in this type of subnetwork-subnetwork connections tend to form an large evolutionary unit, requiring simultaneous retention of interconnected genes. We assume that the metabolism intermediates between the upstream and downstream enzymes are the key biological driving force. Maintaining balanced metabolic flux is important for the survival of plants. Recent experimental data [31] demonstrated that over-expression of a gene in the *OSC-Cyp708 *operon-like gene cluster resulted in the abnormal development of the *Arabidopsis *plant. Clear dosage effects were observed in the genetic analysis of saponin biosynthesis pathway in oats [32]. *Sad1 *encodes 2,3-oxidosqualene to produce β-Amyrin, which eventually is used to produce avenacin A-1 by Sad3 and Sad4. Double mutant analysis revealed that *Sad1Sad1-sad3sad3, Sad1Sad1-sad4sad4 *and *Sad1sad1-sad3sad3 *have abnormal root development, while *Sad1sad1-sad4sad4 *has the normal root development. In many cases, accumulation of metabolic intermediates would be toxic to plants and simultaneous duplication of consecutive steps in the metabolic pathways is required and favoured.

Apart from the potential dosage sensitivity of metabolic interaction, simultaneous expression divergence (subfunctionalization, DDC model [30]) of both interacting enzymes-homeologs could also promote the retention of homeologs, in which the coordinated expressional divergence has the strongest effect in achieving the simultaneous retention of enzyme-homeologs. Four interacting enzyme pairs showed concerted divergence in transcript expression of developmental stages (Additional file 10). That is, in the glycerophospholipid metabolism pathway, the interacting enzymes, EC4.1.1.65 and EC3.1.4.4, were found to be retained as WGD-paralogs, the retained paralogs of *At1g52570-At5g57190 *and *At3g15730-At4g25970 *have both coordinately diverged and express in different developmental stages for the benefit of the interaction. The other three divergently connected enzymes showing co-expression are EC2.3.1.12 and EC1.8.1.4, which were found in the gluconeogenesis pathway, EC2.3.16 and EC2.3.3.1, which mediate fatty acid metabolism and the citrate cycle, and EC2.6.1.2 and EC2.3.1.1 which mediate glutamate metabolism and metabolism of amine groups. The concerted divergence of WGD-enzymes may provide an easy route for the retention of consecutive steps. Since only four such interacting enzyme pairs were found, this mechanism seems to make only a small contribution to the retention of enzyme-homeologs.

The gene balance hypothesis also predicts that more "connected" genes are less likely to be retained as a tandem or transposed duplicate and are more likely to be retained postpaleotetraploidy [30]. It is indeed that genes encoding the hub and bottleneck enzymes in the *Arabidopsis *metabolic network prefer to retain homeologs through WGD but are not preferentially enlarged in their copy number by tandem duplication.

*Arabidopsis *is a good model for plant polyploidization studies. Many economically important plants such as cotton, Brassica rapa, potato, soybean, maize and wheat are polyploids. Through millennia of hybridization and domestication, wheat cultivars that are diploid, tetraploid and hexaploid have been produced. After the divergence from the ancestral sorghum genome, the tetra-ploidization of the progenitor genomes of maize occurred about 5~12 million years ago [33]. Polyploidization eventually leads to offspring that are distinguished from its progenitors. Analysis of the *Arabidopsis *metabolic network shows that both its robustness has been enhanced by the process of WGD. We predicted that the polyploidizations of these main agricultural plants would have increased their environmental adaptability and provided human-needed traits for domestication. Hence, polyploid breeding might be worth more attention in plant breeding programs. Also in transgenic-based plant breeding, the simultaneous engineering of a set of hub and bottleneck genes/enzymes would be a better strategy than manipulation of a single gene/enzyme. As more plant genomes are sequenced, a deeper view of the evolutionary impact of WGD can help us to develop better breeding strategies in modern agriculture.

## Conclusions

In this study, we analyzed the *Arabidopsis *metabolic network by assigning the enzymes with three topological measures, in-degree, out-degree and the between-ness. Comprehensive analyses were carried out between the three centralities and the characteristics of the encoded genes, such as expression intensity, evolutionary rate, and retention of homeologs through WGD. Our results revealed that genes encoding hub and bottleneck enzymes in the metabolic network are preferentially retained after WGD. Furthermore the finding suggested that the retention of metabolic genes was influenced by their interactions and validated that the preferential retention of WGD homeologs encoding hub and bottleneck enzymes is due mainly to the potential dosage effect among interacted genes encoding enzymes if exist). Our results could help us get a deeper view of the evolution of plant metabolic network.

## Methods

### Construction of *Arabidopsis *metabolic network

Three files were downloaded to reconstruct the *Arabidopsis *metabolic network: (a) an expert-curated list of *Arabidopsis *encoded enzymes and the corresponding genes from Aracyc (ftp://ftp.plantcyc.org/Pathways, june,2008 updated) [19], (b) a "reaction" file ftp://ftp.genome.jp/pub/kegg/ligand/reaction/reaction/ to scan all catalyzed reactions of Arabidopsis enzymes [34], (c) a "reaction_mapformula.lst" file ftp://ftp.genome.jp/pub/kegg/ligand/reaction/reaction_mapformula.lst to obtain the information of metabolites in reactions. The reactions between metabolites were used to determine the interactions among enzymes. In the Figure 1A, enzyme EC2.7.7.9 uses alpha-D-glucose-1-phosphate as substrate to produce UDP-glucose, which is then used by enzyme EC5.1.3.2, the interaction was defined as EC2.7.7.9 → EC 5.1.3.2. Because small molecules, H+, NADH, NADP, NADPH, NH3, ATP, ADP, AMP, NAD, CoA, O2, CO2, Glu and pyrophosphate, are involved in many reactions or are used as carriers for transferring electrons, they were excluded from the analysis [35,36].

### Calculation of node in-degree, out-degree and between-ness

The in-degree was calculated by the number of enzymes providing substrates for the considered enzyme, whereas the out-degree was calculated by the number of enzymes using the products of the considered enzyme as substrates. The between-ness was calculated by the "breath-first tree" based algorithm as following steps [18,37]. (a) The calculation was initialized by defining the between-ness of every vertex *j *in the network as *B*(*j*) = 0. (b) Starting from vertex *i*, a breadth-first tree http://en.wikipedia.org/wiki/Breadth-first_search was built with *i *on the top, those that were nearest to *i *directly below and those that were farthest from *i *at the bottom. Each node was placed at a certain level of the tree based on its shortest metabolic reaction step. (c) *P*(*n*) = 1 was assigned to every vertex *j *in the tree. For every vertex *j *, where *k *is the set of nodes that directly connect ("provides substrates") to *j*. (d) *B*(*j*) = 1 was assigned to every vertex *j *in the tree. (e) Starting from bottom vertex *j *of the tree, *B*(*j*) was added to the corresponding variable of the predecessor of *j*. If *j *had more than one predecessor ("enzyme *k *provides more than one substrate to the enzyme *j*"), each predecessor *k *was assigned the value of . (f) Step *(e) *was performed for every vertex in the tree. (g) Steps (*b*)-(*f*) were repeated for every vertex in the network. Finally, every vertex in the network was assigned with a between-ness value which is the sum of its between-ness of every sub-tree involved.

### Modular analysis of the metabolic network

The node distributions of P <In> and P <Out> were used to investigate the frequency of the in-degree and out-degree. The least-squares method was used to estimate power-law exponent of *p*(*k*)∞***k***^-*t *^for log-transformed data (t, power exponent; *k*, in/out-degree). Since the estimated power-law exponent was 1.67, methods for study of scale-free structure was applied in analysis the *Arabidopsis *metabolic network. The algorithm of Guimera and Amaral [37], with parameter settings as iteration factor = 1.0, cooling factor = 0.95 and number of randomization = 100, was used to measure the extent of modularity of network and separate the network into topological modules. The Kobas toolkit [38] was used to infer the frequent pathways in every topologically separated module.

### Analysis of transcription datasets of *Arabidopsis*

The gene transcription datasets of different developmental stages were obtained from the Affymetrix ATH1 data (TAIR accession number, ME00319) [39]. The raw data were normalized by the Affymetrix detection algorithms in the MAS5 library, the background levels and PM/MM ratios were corrected according to the Affymetrix Statistical Algorithms. Based the estimated expression values of probes, the expression values of corresponding 22,380 *Arabidopsis *genes. After filtering the mixture of RNA pools or measurement of the same developmental stages, 59 datasets of ME00319, which measured the developmental stages of *Arabidopsis*, were selected for downstream analysis (Additional file 3). Expression intensities were averaged among three replicates for every developmental stage.

The Expression Variation index *V*_*i *_was used to measure the variations of gene i in expressional level across developmental stages [40].

where n is the number of stages, *S*_*ij *_is the expression signal of gene i in tissue j, *S*_(*i*, max) _is the highest expression signal of gene i across the stages, if the *S*_*ij *_is lower than 50 we arbitrary let it be 50 to minimize the influence of noise from low intensities. The *V*_*i *_value ranges form 0 to 1, higher value indicating higher variations in expressional level across stages or tend to be stage specific genes.

The expression maximum density of enzymes were calculated by  (where *n *was the number of genes annotated with this enzyme, *S*_(*i*, max) _was the maximum expression density of gene *i *among the developmental stages). The expression variation of enzymes were calculated by  (where *n *was the number of genes annotated with this enzyme, *ν*_(*i*) _was Expression Variation index gene *i*).

The Pearson correlation coefficient (*P*_*ij*_) was used to measure the co-expression between gene *i *and gene *j *as following formula:

where n = the number of developmental stages, *S*_*ki *_and *S*_*kj *_were the expression value of gene *i *and *j *under condition *k*;  and  were the mean expression value of gene *i *and *j *across the stages, the *P*_*ij *_was between -1 and 1, with 1 standing for highly co-expressed whereas -1 standing for highly divergent expressed.

The datasets, which time-seriously measured the shoot tissues responding to various abiotic stress (exogenous factors: cold, genotixic, osmotic, salt, UV-B, wound, drought, and heat, see Table S3), were also analyzed with the same procedures.

### Identification of ortholog groups between *Arabidopsis *and *Populus*

The phylogenetic tree approach was used to infer orthologs between *Arabidopsis *and *Populus*. The proteomes of *Arabidopsis thaliana*, *Populus trichocarpa *and *Oryza sativa japonica *were downloaded from http://www.tigr.org/tdb/e2k1/ath1/, http://genome.jgi-psf.org/Poptr1_1/ and http://rice.plantbiology.msu.edu/index.shtml. The sequences of three species were used an all-against-all BLAST with an cutoff of 1e-10, by transforming the E-values with the absolute values of their logarithm[41], the score matrix were constructed and used for similarity clustering with Markov Clustering [42]. The protein clusters containing the *Arabidopsis *metabolic genes were used for phylogenetic analysis.

The protein sequences of members in each cluster were aligned with ClustalW [43] and the alignments were used to generate neighbor-joining trees with the two-parameter substitution correction. The phylogenetic trees were rooted at midpoints. By reconciling between phylogentic tree and the species tree ((*Arabidopsis thaliana*, *Populus trichocarpa*), *Oryza sativa japonica*)) with Notung [44], the ortholog groups were identified between *Arabidopsis *and *Populus*.

### Measurement of the evolutionary rates in coding region and 5' upstream, 3' downstream regions of genes encoding enzymes in *Arabidopsis*

The Clustalw was used to globally align two amino acid sequences of orthologs between *Arabidopsis *and *Populus*, and the corresponding coding sequences were realigned with the gaps in the alignment trimmed. The *Ka *was estimated from the codon-based nucleotide sequence alignment by using the Yang-Nielsen maximum-likelihood method implemented in the yn00 program of the PAML package[45]. To calculate the substitution rates in *Arabidopsis *genes 5' 1000bp upstream and 3' 1000 bp downstream region were calculated against that of *Populus *orthologs. The Clustalw software [43] were used to globally align the non-coding regions of orthologs, the substitution rate per sites *K*_5*u *_and *K*_3*u *_with the Kimura two-parameter model were calculated by dismat program of the EMBOSS package [46]. For a *Arabidopsis *gene *i *with more than one orthologs in *Populus*, the smallest of calculated *Ka*, *K*_5*u *_and *K*_3*u *_were selected as *Ka*_(*i*)_, *K*_5*u*(*i*) _and *K*_3*u*(*i*)_.

The average divergence in coding regions, 5'upstream and 3'downstream region of enzyme could be calculated by the formula ,  and , the *n *was the number of genes annotated by this enzyme, *Ka*_(*i*)_, *K*_5*u*(*i*) _and *K*_3*u*(*i*) _was the substitution rate in coding, upstream and downstream non-coding regions of *Arabidopsis *gene.

### Statistical analysis and computational methods

Spearman's rank correlation coefficients were estimated to evaluate the correlations between three topological centralities and expression intensities, expression variation, substitution rates of coding regions of gene encoding enzymes. The p-values were FDR-corrected by using the Q-value program in R package [47]. The comparison of topological centralities between WGD-enzymes and the other enzymes were done by using Manny-Whitney U with two-tail test. Computations were performed on a Linux cluster with 16 nodes (Intel 5130, 2.0 GHz CPU, 4G memory, Research Center for Systematic and Evolutionary Botany, Institute of Botany, CAS). Perl http://perl.org and R http://www.r-project.org/ scripts were used for analysis, and can be obtained on request.

## Abbreviations

Ka: non-synonymous substitution rates of coding region; K5u: substitution rate of 5'UTR; K3u: substitution rate of 3'UTR; WGD: whole genome duplication; WGD: enzyme, the enzyme retaining paralogs through whole genome duplication; IBWE: the interaction between WGD-enzymes.

## Authors' contributions

XW and XQ performed the experiments and analyzed the data, XQ directed the project and contributed the materials and analysis tools. XW and XQ wrote the paper. All authors read and approved the final manuscript.

## Supplementary Material

Additional file 1 Table S1. 478 enzymes in the largest network and their topological centralities. Click here for file

Additional file 2 Table S2. 11 topological modules and their major functions. Click here for file

Additional file 3 Table S3. The description of *Arabidopsis *microarry datasets. Click here for file

Additional file 4 Table S4. The identified ortholog groups between *Arabidopsis *and *Populus *by phylogenetic trees. Click here for file

Additional file 5 Table S5. The identified ortholog groups between *Arabidopsis *and rice by phylogenetic trees. Click here for file

Additional file 6 Table S6. 173 *Arabidopsis *WGD-enzymes and their coding homeologs. Click here for file

Additional file 7 Table S7. 25 *Arabidopsis *tandem-enzymes and their coding tandem homeologs in the *Arabidopsis *metabolic network. Click here for file

Additional file 8 Figure S1. Comparison of three topological centralities between enzymes retaining tandem-homeologs and the other enzymes. Click here for file

Additional file 9 Table S8. 226 *Populus *WGD-enzymes and their coding homeologs. Click here for file

Additional file 10 Figure S2. The expression profiles of four connected enzyme-homeologs in the 59 developmental stages. Click here for file
