# Supplementary material for: Genes encoding hub and bottleneck enzymes of the Arabidopsis metabolic network preferentially retain homeologs through whole genome duplication
Source: BMC Evol Biol. 2010 May 18;10:145. doi: 10.1186/1471-2148-10-145 (PMC2880986; doi:10.1186/1471-2148-10-145)
Supplement: Additional file 1 — Table S1. 478 enzymes in the largest network and their topological centralities. [file 1471-2148-10-145-S1.PDF]

Table S1. 478 *Arabidopsis* enzymes in the largest network and their topolical centralities

Enzyme	In-degree	Out-degree	Between-ness	Average Ka	Average K5u	Average K3u	Maximum intensity (developmental stages)	Maximum intensity (shoot after stress)	Expression Variation (developmental stages)
3.1.2.6	1.00	0.00	1.00	0.15	0.30	0.31	2232.57	1228.00	0.14
1.11.1.6	1.00	0.00	1.00	0.05	0.12	0.20	13826.22	7200.30	0.20
6.1.1.21	1.00	0.00	1.00	0.17	NA	NA	538.25	387.45	0.10
2.7.8.15	2.00	0.00	2.00	0.22	NA	0.27	500.58	182.58	0.14
2.4.1.117	1.00	2.00	3.00	0.19	0.47	0.50	616.55	328.65	0.11
2.7.1.108	0.00	3.00	4.00	0.29	NA	0.54	2466.20	567.40	0.25
5.5.1.9	0.00	1.00	5.00	0.14	NA	NA	1168.15	284.15	0.16
3.1.1.14	1.00	2.00	14.00	0.26	NA	NA	1795.68	1815.78	0.25
6.1.1.20	5.00	0.00	373.00	0.24	0.27	0.28	723.81	415.45	0.04
2.3.1.92	1.00	0.00	373.00	NA	NA	NA	3577.05	3899.70	0.23
4.2.3.15	3.00	0.00	373.00	0.46	NA	NA	3933.72	223.98	0.46
3.5.4.26	1.00	0.00	373.00	NA	NA	0.53	796.60	367.55	0.14
4.2.3.16	3.00	0.00	373.00	0.41	NA	NA	5478.45	212.18	0.49
2.7.1.100	1.00	0.00	373.00	0.17	0.45	0.56	968.80	432.85	0.16
1.-.-.-	1.00	0.00	373.00	0.23	0.18	0.20	1824.94	1622.92	0.27
1.14.99.30	1.00	0.00	373.00	0.10	0.58	0.62	2334.90	1843.85	0.13
1.13.99.1	1.00	0.00	373.00	0.16	0.16	0.30	15551.92	404.77	0.35
1.3.1.26	1.00	0.00	373.00	0.16	0.35	0.34	5065.80	1586.00	0.12
1.1.1.271	1.00	0.00	373.00	0.14	0.26	0.25	1039.10	223.08	0.20
2.4.1.83	3.00	0.00	373.00	0.05	0.52	0.49	625.95	313.35	0.11
2.3.3.13	15.00	0.00	373.00	0.29	NA	0.13	2865.40	1492.05	0.25
6.3.2.2	14.00	0.00	373.00	0.14	0.49	0.52	3949.15	2034.90	0.10
1.3.1.42	1.00	0.00	373.00	0.16	0.17	0.18	1496.38	1757.57	0.10
4.1.1.18	4.00	0.00	373.00	NA	NA	NA	23259.00	9325.25	0.12
1.14.17.4	1.00	0.00	373.00	0.46	0.09	0.08	3566.21	1767.34	0.24
3.2.1.1	1.00	0.00	373.00	0.22	0.26	0.32	1231.42	1034.62	0.18
6.1.1.12	5.00	0.00	373.00	0.21	0.26	0.29	905.69	570.36	0.06
2.4.1.25	2.00	0.00	373.00	0.19	NA	NA	803.75	1007.85	0.12
2.7.1.36	1.00	0.00	373.00	0.21	0.52	NA	992.35	200.85	0.18
4.99.1.4	1.00	0.00	373.00	NA	0.57	NA	878.80	495.20	0.10
6.1.1.14	5.00	0.00	373.00	0.10	0.53	0.49	1432.23	685.60	0.13
6.1.1.11	4.00	0.00	373.00	0.16	0.54	0.47	1547.30	749.65	0.11
2.7.8.11	1.00	0.00	373.00	0.19	NA	NA	961.70	475.45	0.07
4.2.3.14	3.00	0.00	373.00	NA	NA	NA	330.15	208.00	0.47
2.7.8.5	1.00	0.00	373.00	0.21	0.28	0.26	898.28	317.98	0.12
6.1.1.9	1.00	0.00	373.00	NA	NA	NA	22.60	21.50	0.01
5.1.3.5	1.00	0.00	373.00	0.12	0.18	0.16	1079.83	468.37	0.29
2.1.1.143	1.00	0.00	373.00	0.10	0.14	0.36	2749.05	1354.88	0.13
2.8.1.7	4.00	0.00	373.00	0.17	0.54	0.25	827.00	691.23	0.10
2.4.1.111	2.00	0.00	373.00	0.36	0.16	0.15	2319.33	237.53	0.39
2.5.1.8	3.00	0.00	373.00	0.27	0.27	0.25	316.65	186.25	0.17
2.5.1.27	1.00	0.00	373.00	0.37	0.09	NA	192.42	61.75	0.18
1.1.1.100	2.00	0.00	373.00	0.21	0.59	0.54	5067.95	1602.50	0.10
6.1.1.6	4.00	0.00	373.00	NA	NA	NA	2720.65	1414.70	0.09
1.17.1.3	1.00	0.00	373.00	0.26	0.51	0.52	5332.25	6.20	0.52
4.2.1.76	4.00	0.00	373.00	0.08	0.50	0.50	3882.00	1092.00	0.15
1.14.11.13	2.00	0.00	373.00	0.30	0.39	0.29	803.31	466.50	0.23

6.1.1.3	2.00	0.00	373.00	0.47	0.19	0.37	717.50	397.30	0.13
2.7.7.3	2.00	0.00	373.00	0.15	0.57	0.60	448.05	236.65	0.10
6.1.1.18	3.00	0.00	373.00	0.12	0.62	0.52	1402.05	713.20	0.10
6.1.1.10	5.00	0.00	373.00	0.14	0.22	NA	654.60	270.18	0.04
1.13.11.5	2.00	0.00	373.00	0.14	NA	0.49	2794.00	2636.90	0.24
3.13.1.1	4.00	0.00	373.00	0.14	0.47	0.53	3132.95	1191.45	0.15
2.7.2.8	1.00	0.00	373.00	NA	NA	NA	1145.75	642.15	0.11
2.6.1.62	1.00	0.00	373.00	0.17	0.36	0.50	2429.01	2167.65	0.14
2.3.1.91	1.00	0.00	373.00	NA	NA	NA	3977.20	114.90	0.48
1.3.-.-	1.00	0.00	373.00	0.11	0.46	0.50	6295.35	3119.00	0.11
3.2.1.2	1.00	0.00	373.00	0.46	0.18	0.19	2301.51	2600.05	0.19
6.1.1.16	4.00	0.00	373.00	0.24	0.29	0.37	556.93	384.72	0.14
6.1.1.22	2.00	0.00	373.00	0.21	0.41	0.28	301.36	115.33	0.10
3.2.1.4	1.00	0.00	373.00	0.21	0.18	0.22	4431.21	601.15	0.31
2.3.1.50	6.00	0.00	373.00	0.12	0.23	0.30	1781.58	158.85	0.18
2.4.1.91	1.00	0.00	374.00	NA	NA	NA	2552.10	705.75	0.39
2.1.1.41	1.00	0.00	375.00	0.10	0.16	0.36	2749.05	1354.88	0.14
1.14.11.15	1.00	1.00	375.00	0.32	0.15	0.19	410.77	315.53	0.27
1.14.11.9	2.00	2.00	380.00	0.14	0.48	0.56	7228.95	1963.65	0.22
1.1.1.169	1.00	1.00	401.25	NA	NA	NA	657.35	403.25	0.01
3.1.2.12	0.00	1.00	433.00	0.12	0.42	0.63	2610.00	1168.35	0.08
6.3.3.1	0.00	1.00	433.00	0.22	NA	NA	1176.60	333.85	0.16
2.3.1.129	0.00	1.00	433.00	NA	NA	NA	106.20	42.85	0.15
1.14.13.11	0.00	2.00	433.00	0.11	0.38	0.51	5904.65	2350.05	0.19
1.3.1.9	0.00	5.00	433.00	0.16	0.53	0.62	5183.35	1104.45	0.18
1.2.99.2	0.00	11.00	433.00	0.21	0.53	0.52	1713.32	784.78	0.13
2.8.2.-	0.00	2.00	433.00	0.36	NA	NA	3177.92	2279.23	0.23
3.5.1.19	0.00	1.00	433.00	0.21	0.33	0.35	573.27	82.72	0.29
2.4.1.132	0.00	1.00	433.00	0.23	0.51	0.52	108.95	57.20	0.06
2.7.7.14	0.00	1.00	433.00	NA	0.50	0.48	1284.45	869.35	0.10
5.5.1.-	0.00	2.00	433.00	0.14	0.56	0.53	2021.43	1562.50	0.15
4.1.1.33	0.00	5.00	433.00	0.13	0.26	0.24	957.58	230.18	0.17
4.1.3.38	0.00	1.00	433.00	0.26	0.49	0.62	635.05	617.20	0.09
2.4.1.115	0.00	2.00	433.00	NA	0.45	0.50	1824.05	2858.15	0.22
3.7.1.2	0.00	4.00	433.00	NA	0.68	0.58	1058.15	609.70	0.15
2.4.2.19	0.00	3.00	433.00	0.12	0.50	0.55	1705.00	756.55	0.13
1.8.7.1	0.00	1.00	433.00	0.14	NA	NA	2845.60	1657.15	0.11
1.13.11.54	0.00	2.00	433.00	0.13	0.25	0.26	3869.35	2838.69	0.14
5.1.1.7	0.00	1.00	434.00	0.20	0.50	0.60	1266.00	679.45	0.06
1.1.1.23	0.00	2.00	434.00	0.09	0.53	0.52	1159.10	636.80	0.13
2.7.1.130	0.00	1.00	434.00	0.33	0.46	NA	95.90	53.10	0.13
4.4.1.9	0.00	2.00	434.00	NA	0.58	0.50	6907.70	4701.95	0.10
4.4.1.5	0.00	2.00	434.00	0.18	0.29	0.27	5553.49	1527.94	0.26
2.7.4.7	0.00	1.00	434.00	0.24	0.55	0.46	363.95	198.60	0.11
5.4.99.-	0.00	2.00	434.00	0.15	NA	NA	1837.56	90.88	0.28
2.1.1.103	0.00	2.00	435.00	0.11	0.15	NA	3474.22	1922.19	0.22
2.7.1.32	1.00	1.00	435.00	0.23	0.15	NA	7013.15	782.28	0.36
1.14.13.90	1.00	1.00	435.00	0.21	NA	0.47	4671.60	5495.40	0.16
4.99.1.6	0.00	2.00	436.00	NA	NA	NA	126.40	1332.90	0.18
3.2.1.147	0.00	2.00	436.00	NA	NA	NA	374.95	138.05	0.08
4.2.1.65	1.00	3.00	451.40	NA	NA	NA	3402.60	901.40	0.38
4.1.1.36	2.00	1.00	543.02	0.17	NA	NA	483.23	85.65	0.10
3.5.1.4	1.00	1.00	628.92	0.26	0.51	0.49	1134.05	425.30	0.18
4.2.1.84	2.00	2.00	630.22	0.15	0.26	0.27	1827.03	513.28	0.24

2.3.1.41	16.00	1.00	668.99	0.16	NA	0.56	2376.70	977.85	0.15
3.5.4.25	2.00	1.00	748.00	0.13	0.17	NA	1089.43	936.07	0.12
1.1.1.34	2.00	1.00	748.00	0.15	0.23	0.24	5507.95	1200.50	0.25
3.2.2.16	2.00	1.00	748.00	0.20	0.27	0.27	2613.15	1386.63	0.13
2.1.1.107	1.00	1.00	748.00	NA	NA	NA	1430.25	1445.50	0.17
4.2.1.47	1.00	1.00	748.00	NA	NA	NA	1209.85	271.30	0.27
4.1.1.35	3.00	1.00	748.00	0.11	0.25	0.24	1528.27	264.84	0.15
4.4.1.14	1.00	1.00	748.00	0.22	0.18	0.17	2085.76	681.97	0.20
2.3.1.1	11.00	1.00	748.00	0.23	NA	0.20	338.43	205.70	0.18
4.2.1.52	2.00	1.00	748.00	0.14	0.26	0.26	735.78	430.10	0.12
2.4.1.12	4.00	1.00	748.00	0.20	0.20	0.23	3087.48	508.95	0.27
2.3.1.47	1.00	1.00	750.00	NA	0.53	0.61	389.90	82.20	0.18
5.4.99.8	1.00	1.00	752.00	0.12	0.47	0.48	1445.65	933.15	0.07
1.1.1.219	3.00	1.00	754.00	0.23	0.45	0.47	5040.85	848.90	0.42
5.3.99.6	1.00	1.00	754.00	0.42	NA	NA	1151.19	591.35	0.22
1.14.13.21	2.00	3.00	755.00	0.21	0.49	0.49	3506.35	137.15	0.28
2.5.1.62	2.00	1.00	757.00	0.13	0.48	0.57	2659.75	2070.85	0.17
5.3.3.5	1.00	1.00	760.00	0.23	0.45	0.54	2753.05	1100.35	0.11
2.7.1.33	1.00	3.00	764.28	0.13	0.53	0.49	1283.15	424.15	0.16
2.1.2.11	2.00	1.00	769.25	0.11	0.23	0.24	217.00	104.98	0.14
2.3.1.39	1.00	2.00	804.00	NA	0.49	0.56	1889.35	410.05	0.19
1.5.1.2	2.00	2.00	804.00	0.18	0.62	0.53	1889.20	1024.45	0.12
1.1.1.27	4.00	6.00	804.00	0.14	0.43	0.49	1269.45	1995.05	0.19
6.3.4.2	1.00	1.00	804.00	0.10	0.32	0.32	2035.18	755.83	0.19
4.2.1.46	1.00	2.00	804.00	0.13	0.49	0.51	445.90	286.85	0.01
6.1.1.7	1.00	1.00	804.00	0.18	0.48	0.49	874.65	621.97	0.03
6.3.3.2	1.00	3.00	804.00	NA	0.53	0.51	376.00	211.45	0.06
2.3.1.20	3.00	1.00	804.00	0.23	0.22	NA	5809.08	1955.00	0.23
2.6.1.11	1.00	1.00	804.00	0.21	0.53	0.46	2359.85	1419.30	0.10
1.14.13.36	1.00	1.00	804.00	0.12	0.51	0.49	3644.55	1053.50	0.21
1.5.1.9	1.00	1.00	804.00	0.20	0.51	NA	6294.50	8287.60	0.32
1.8.1.9	1.00	1.00	804.00	0.12	0.14	0.18	4137.35	2241.97	0.11
5.4.4.2	2.00	2.00	804.00	NA	0.24	NA	1015.70	778.10	0.34
3.1.3.37	1.00	2.00	804.00	0.12	0.47	0.52	11097.40	8328.40	0.16
2.1.1.104	4.00	2.00	804.00	0.22	NA	NA	573.15	90.55	0.36
2.7.6.3	1.00	1.00	804.00	NA	NA	NA	51.70	21.30	0.01
2.7.1.140	1.00	2.00	804.00	0.27	0.26	NA	781.73	504.20	0.09
2.3.1.158	3.00	1.00	804.00	0.17	NA	NA	895.00	606.25	0.17
5.4.2.3	1.00	1.00	804.00	0.24	0.52	0.48	229.30	115.85	0.10
3.5.2.2	1.00	1.00	804.00	NA	NA	NA	1387.85	673.55	0.18
3.4.19.9	1.00	8.00	804.00	0.26	0.17	0.17	2572.82	1356.95	0.19
6.1.1.19	1.00	5.00	804.00	0.13	0.29	0.27	1097.15	525.78	0.12
3.5.4.5	1.00	1.00	804.00	0.27	0.08	NA	657.55	74.75	0.19
1.6.5.3	1.00	1.00	804.00	0.16	0.25	0.29	2343.83	1463.12	0.06
2.5.1.22	1.00	3.00	804.00	0.14	0.51	0.53	2982.00	1538.80	0.20
2.4.2.9	3.00	2.00	804.00	0.10	0.23	0.28	997.88	345.39	0.19
2.4.1.67	2.00	2.00	804.00	0.26	NA	NA	1578.85	32.40	0.45
2.7.7.18	4.00	2.00	804.00	NA	0.50	NA	NA	NA	0.08
2.7.7.44	2.00	4.00	804.00	NA	0.54	0.51	1524.50	435.40	0.15
3.1.2.4	1.00	2.00	804.00	0.19	0.32	0.39	751.65	293.88	0.15
1.4.7.1	3.00	13.00	804.00	0.09	0.26	0.28	4617.90	3344.35	0.19
6.3.2.5	5.00	1.00	810.23	NA	0.56	0.52	197.55	164.75	0.00
1.2.1.68	3.00	2.00	822.00	0.17	0.49	0.52	4380.25	2614.20	0.19
1.5.1.20	3.00	1.00	831.33	0.13	0.28	0.25	2572.85	280.25	0.08

1.1.1.205	1.00	4.00	834.50	0.20	0.22	0.23	984.30	512.65	0.16
1.14.13.39	2.00	2.00	850.40	NA	0.58	0.60	630.50	339.60	0.21
1.4.1.14	7.00	13.00	851.38	0.10	0.59	0.55	5922.70	2275.90	0.14
3.1.3.24	2.00	2.00	853.97	0.22	NA	0.15	936.18	462.04	0.25
6.4.1.4	3.00	1.00	856.23	0.15	0.46	0.53	2114.28	1073.38	0.18
2.-.-.-	1.00	1.00	866.00	0.26	0.51	0.58	131.40	81.85	0.12
2.5.1.3	1.00	1.00	866.00	0.24	0.53	NA	363.95	198.60	0.10
4.1.1.20	1.00	4.00	866.00	0.13	0.28	0.26	110.55	36.35	0.05
1.10.99.3	1.00	2.00	867.00	0.19	0.52	0.58	1451.60	1387.60	0.19
2.4.1.123	3.00	2.00	875.15	0.15	0.29	0.30	1922.82	4085.54	0.33
2.4.1.14	4.00	2.00	875.95	0.66	0.37	0.27	1948.04	1501.26	0.28
2.4.1.13	4.00	2.00	879.67	0.25	0.33	0.28	4178.50	1758.76	0.34
2.5.1.32	1.00	1.00	880.55	NA	NA	NA	4835.40	3493.15	0.15
6.3.2.12	1.00	2.00	884.50	0.27	0.24	0.53	450.33	310.25	0.11
1.5.99.8	2.00	3.00	891.41	0.35	NA	NA	15146.85	1342.80	0.31
1.4.1.2	11.00	15.00	896.39	0.07	0.54	0.48	10273.55	1097.53	0.24
3.5.1.1	2.00	8.00	910.71	0.82	0.51	0.51	1091.15	356.05	0.21
3.5.1.10	3.00	8.00	921.31	0.31	0.16	0.27	1876.36	399.15	0.17
3.6.1.23	1.00	2.00	922.44	NA	0.54	0.57	1470.35	492.15	0.22
1.1.1.195	3.00	4.00	925.70	0.14	0.22	0.17	2912.21	1157.83	0.26
2.7.1.17	3.00	3.00	935.98	0.20	0.28	0.25	677.98	463.80	0.17
1.1.99.14	2.00	4.00	948.55	NA	NA	NA	388.85	281.25	0.10
1.1.3.15	2.00	4.00	948.55	0.11	0.11	0.18	2636.68	400.05	0.17
5.3.1.5	5.00	4.00	963.54	0.14	0.55	0.48	2483.60	670.60	0.22
3.1.3.18	1.00	3.00	965.39	0.14	0.19	0.20	167.20	85.43	0.08
2.7.7.10	3.00	5.00	974.93	0.16	0.51	0.52	3568.57	990.77	0.17
2.7.2.11	11.00	1.00	981.52	0.15	NA	NA	578.40	202.65	0.16
3.4.11.2	1.00	9.00	983.49	0.18	0.53	0.50	3026.30	1271.60	0.16
3.5.3.1	2.00	3.00	1009.57	0.06	0.42	0.54	7563.60	4049.95	0.01
1.8.5.1	1.00	2.00	1018.50	0.17	0.20	0.22	1385.64	657.56	0.13
1.11.1.11	1.00	2.00	1018.50	0.19	0.27	0.12	2426.44	2210.83	0.11
2.1.1.10	3.00	2.00	1027.89	0.17	0.50	0.49	4790.30	274.88	0.32
2.1.1.14	3.00	2.00	1027.89	0.06	0.17	0.17	1453.58	597.80	0.09
4.2.1.19	1.00	1.00	1034.47	NA	NA	NA	511.85	312.08	0.06
2.7.1.151	1.00	2.00	1034.50	0.27	0.25	NA	781.73	504.20	0.09
1.14.19.2	2.00	2.00	1041.32	0.17	0.08	0.11	1905.73	515.38	0.28
4.1.3.1	2.00	7.00	1062.79	0.15	0.25	0.25	13143.63	790.60	0.40
1.2.4.2	8.00	3.00	1092.86	0.08	0.22	0.25	1700.88	596.10	0.18
2.4.1.82	4.00	3.00	1102.56	0.21	0.57	0.51	1685.30	4594.00	0.29
3.5.5.1	2.00	1.00	1106.78	0.15	0.09	0.11	1715.71	278.81	0.19
4.2.1.3	3.00	4.00	1111.99	0.08	0.30	0.35	4295.90	2862.62	0.09
1.3.3.6	3.00	3.00	1121.76	0.15	0.22	0.26	2162.50	842.03	0.13
2.4.1.120	1.00	2.00	1122.00	0.37	0.13	NA	1654.55	524.06	0.30
3.1.2.-	4.00	1.00	1122.00	0.13	0.50	0.57	7303.60	2599.30	0.19
4.2.1.92	1.00	1.00	1128.00	0.24	0.45	0.52	3359.70	4037.15	0.28
1.3.1.75	2.00	1.00	1131.00	0.18	0.50	0.49	2419.95	1436.50	0.21
6.3.2.1	5.00	1.00	1136.28	0.22	0.50	0.52	191.95	223.90	0.11
1.3.1.70	1.00	1.00	1137.00	0.23	0.44	0.48	596.85	355.85	0.10
1.4.4.2	6.00	1.00	1166.92	0.12	0.31	0.30	7327.22	5487.16	0.17
2.7.1.134	3.00	2.00	1175.00	NA	NA	NA	2520.40	954.15	0.15
3.5.3.12	1.00	1.00	1175.00	0.18	0.54	0.53	905.95	275.40	0.18
5.3.3.2	2.00	5.00	1176.00	0.11	0.34	0.23	2185.25	450.43	0.18
2.6.1.42	1.00	5.00	1176.00	0.23	0.17	0.18	1910.07	692.36	0.20
2.7.1.11	7.00	3.00	1186.03	0.15	0.08	0.14	1807.58	735.29	0.21

2.7.2.3	6.00	5.00	1209.02	0.09	0.37	0.35	7523.40	6102.27	0.11
6.2.1.5	5.00	2.00	1219.40	0.10	0.28	0.34	2074.67	1321.77	0.08
5.3.1.1	6.00	7.00	1223.29	0.10	0.52	0.48	7724.93	4978.98	0.08
3.5.1.53	1.00	1.00	1234.00	0.08	0.51	0.51	1247.70	690.20	0.11
1.2.1.41	3.00	2.00	1234.00	0.15	NA	NA	578.40	202.65	0.16
3.1.3.3	1.00	6.00	1234.36	NA	0.51	0.59	560.20	252.80	0.12
4.1.1.21	2.00	1.00	1235.00	0.52	0.25	0.31	356.25	287.85	0.10
2.4.2.11	5.00	2.00	1235.00	0.11	0.23	0.20	612.50	413.40	0.12
1.5.1.3	10.00	8.00	1247.56	0.14	0.19	0.17	372.62	58.93	0.10
4.2.1.18	2.00	2.00	1268.18	NA	NA	NA	690.75	249.25	0.17
4.1.1.50	1.00	1.00	1280.54	0.25	0.28	0.13	6846.51	3521.45	0.18
2.7.1.30	1.00	2.00	1283.36	0.13	0.43	0.45	3837.20	547.40	0.22
2.4.1.241	2.00	1.00	1290.99	0.20	0.51	0.53	559.10	110.75	0.25
2.4.1.184	2.00	1.00	1290.99	0.19	0.52	0.46	1655.95	2621.50	0.19
2.7.7.15	2.00	1.00	1299.00	0.19	NA	NA	1321.48	873.38	0.22
3.1.1.4	4.00	3.00	1316.74	0.29	0.35	0.24	360.65	209.56	0.16
3.1.1.32	5.00	1.00	1320.73	0.21	0.54	0.19	845.77	346.68	0.25
6.3.4.3	11.00	4.00	1324.06	0.07	0.45	0.48	646.35	185.70	0.08
1.2.1.25	2.00	2.00	1343.84	0.22	0.14	0.12	1496.16	535.64	0.20
1.1.1.42	4.00	7.00	1354.82	0.11	0.35	0.41	3129.53	1744.55	0.10
1.3.99.12	2.00	1.00	1355.89	NA	0.46	NA	1307.65	769.15	0.29
1.3.99.10	2.00	2.00	1380.65	NA	0.45	NA	1307.65	769.15	0.29
3.1.4.11	2.00	7.00	1385.67	0.16	0.15	0.15	1137.73	601.86	0.19
2.4.1.215	4.00	3.00	1404.76	0.38	0.10	0.10	894.07	479.07	0.24
2.4.1.203	4.00	3.00	1404.76	0.38	0.12	0.11	894.07	479.07	0.23
2.6.1.5	6.00	11.00	1412.98	NA	0.22	0.27	3259.35	2178.65	0.35
4.1.1.31	2.00	5.00	1420.15	0.07	0.30	0.28	2446.50	736.75	0.23
5.1.3.1	4.00	4.00	1421.49	0.12	0.35	0.37	4291.80	2093.35	0.10
2.7.1.48	2.00	3.00	1432.13	0.08	0.38	0.63	840.35	325.20	0.13
1.2.1.24	4.00	3.00	1438.09	NA	0.56	NA	621.25	446.80	0.14
3.5.4.16	2.00	1.00	1440.22	0.25	0.47	0.52	1544.35	752.45	0.18
2.6.1.9	6.00	9.00	1444.47	0.19	0.24	NA	217.90	174.75	0.06
5.1.3.2	5.00	16.00	1462.53	0.11	0.17	0.18	1594.19	521.36	0.23
2.7.1.6	3.00	3.00	1469.27	0.16	0.30	0.31	1265.15	249.02	0.22
5.3.1.8	7.00	7.00	1479.64	0.28	0.23	0.27	1392.18	838.68	0.20
1.13.11.12	3.00	1.00	1500.00	0.21	0.34	0.34	3989.67	2236.13	0.32
1.14.13.81	1.00	1.00	1504.00	0.10	0.53	0.49	10745.95	8651.80	0.15
1.14.13.70	2.00	1.00	1512.00	0.11	0.49	0.50	1952.25	677.40	0.11
2.4.1.46	3.00	2.00	1520.53	0.17	0.36	0.32	9059.62	678.05	0.33
1.2.1.27	2.00	12.00	1524.81	NA	0.51	0.52	2721.80	1612.25	0.08
6.3.5.2	4.00	3.00	1536.96	NA	0.53	NA	1534.85	691.15	0.16
3.1.3.11	3.00	9.00	1540.50	0.11	0.50	0.53	5525.75	4654.85	0.20
1.4.3.-	2.00	1.00	1540.57	0.16	NA	NA	790.55	1672.85	0.27
1.1.1.49	3.00	1.00	1545.00	0.12	0.32	0.35	1408.90	685.97	0.19
2.4.1.15	4.00	1.00	1545.00	0.16	0.17	0.16	930.34	571.04	0.14
2.4.1.43	2.00	1.00	1545.00	0.17	0.23	0.19	2908.16	468.31	0.21
1.10.3.3	3.00	2.00	1546.00	0.25	0.26	0.21	1028.50	265.43	0.23
2.1.3.3	6.00	4.00	1564.51	0.16	NA	NA	1498.95	1075.80	0.08
3.6.1.31	1.00	1.00	1565.45	0.24	0.50	0.51	811.75	578.25	0.11
3.1.2.14	3.00	2.00	1567.59	0.15	0.24	0.25	5312.13	656.98	0.25
2.7.1.2	7.00	6.00	1582.58	0.14	0.27	0.28	2160.08	856.03	0.14
6.4.1.2	13.00	11.00	1603.00	0.19	0.41	0.43	3207.20	1610.80	0.12
3.1.1.11	1.00	1.00	1604.00	0.30	0.11	0.14	4017.20	337.50	0.24
3.1.1.31	1.00	1.00	1604.00	NA	NA	NA	3746.55	1094.23	0.36

3.1.3.12	1.00	1.00	1604.00	0.19	0.20	0.21	1918.55	768.84	0.26
1.5.1.8	4.00	4.00	1605.00	0.20	0.50	NA	6294.50	8287.60	0.33
2.1.1.64	2.00	2.00	1605.00	0.25	NA	NA	241.20	103.20	0.11
2.3.1.35	4.00	3.00	1605.00	0.15	0.55	0.59	573.90	415.20	0.08
3.5.1.6	4.00	3.00	1605.00	0.11	0.53	0.57	1644.35	629.75	0.18
2.3.1.4	3.00	2.00	1605.00	0.22	0.65	0.50	150.80	125.55	0.11
2.3.1.133	2.00	4.00	1605.00	0.13	0.49	0.57	7303.60	2599.30	0.20
2.7.7.41	2.00	3.00	1619.46	0.25	0.13	0.18	928.11	426.01	0.19
1.3.5.1	7.00	5.00	1621.00	0.19	0.30	0.29	2546.29	1230.72	0.11
3.5.4.19	1.00	1.00	1624.45	0.24	0.49	0.53	811.75	578.25	0.11
4.1.1.25	5.00	1.00	1634.25	0.15	0.24	0.24	1355.73	321.83	0.24
2.4.1.1	1.00	5.00	1659.00	0.11	0.56	0.45	3001.95	4073.20	0.11
3.2.1.28	1.00	3.00	1663.00	0.30	0.43	0.50	1163.60	171.00	0.29
1.1.1.44	1.00	3.00	1663.00	0.17	0.36	0.40	2051.58	790.18	0.16
3.2.1.15	1.00	1.00	1663.00	0.35	0.10	0.13	2752.21	211.19	0.21
3.1.2.2	2.00	3.00	1672.81	0.30	0.23	0.28	1032.60	240.67	0.21
2.7.4.9	4.00	3.00	1680.14	NA	NA	NA	1244.25	330.60	0.16
4.2.1.55	5.00	2.00	1682.35	0.19	0.50	0.47	2897.95	920.98	0.16
5.3.1.16	1.00	1.00	1683.45	0.19	NA	NA	392.60	186.15	0.13
5.3.1.9	9.00	10.00	1692.08	NA	0.31	0.25	1635.10	756.08	0.10
2.3.1.30	3.00	6.00	1711.44	0.18	0.29	0.32	2654.70	1222.03	0.17
2.1.3.2	7.00	1.00	1730.80	0.08	0.52	0.52	966.10	683.55	0.12
4.1.1.23	1.00	3.00	1756.42	0.15	0.49	0.47	NA	NA	0.00
2.6.1.52	1.00	3.00	1770.63	0.17	NA	NA	1883.90	1772.95	0.13
3.3.1.1	3.00	4.00	1802.89	0.05	0.23	0.25	12242.30	8816.30	0.10
3.5.2.3	2.00	1.00	1855.55	0.11	0.52	0.52	839.15	413.15	0.12
2.1.1.11	1.00	1.00	1875.00	NA	NA	NA	6646.65	4381.10	0.15
5.4.99.7	1.00	1.00	1880.00	NA	NA	NA	363.70	57.25	0.29
2.2.1.2	8.00	8.00	1882.69	0.17	0.23	0.25	3193.60	746.13	0.18
2.7.1.90	8.00	8.00	1887.97	0.10	0.26	0.15	2193.86	813.89	0.15
2.3.2.2	2.00	14.00	1914.31	0.26	0.14	0.15	646.37	458.55	0.21
2.4.2.17	2.00	4.00	1923.83	0.25	0.26	NA	1524.30	1013.28	0.13
1.2.1.13	7.00	8.00	1946.28	0.12	0.40	0.38	12990.83	11239.24	0.18
1.2.1.12	7.00	8.00	1946.28	0.12	0.34	0.33	8478.21	7154.01	0.13
2.5.1.47	2.00	6.00	1954.76	0.16	0.19	0.18	2355.60	1516.50	0.21
1.13.11.27	6.00	3.00	1957.99	0.18	0.49	0.57	2915.20	2813.15	0.27
2.7.1.4	9.00	7.00	2010.22	0.14	0.27	0.26	2160.08	856.03	0.13
2.4.2.14	6.00	1.00	2016.30	0.14	NA	NA	543.12	332.18	0.19
2.5.1.1	5.00	7.00	2025.21	0.17	0.29	0.31	984.51	596.76	0.15
2.5.1.10	5.00	7.00	2025.21	0.18	0.25	0.29	1023.08	450.28	0.12
6.3.2.6	2.00	2.00	2034.00	0.17	0.51	NA	498.10	265.85	0.17
2.6.1.13	5.00	4.00	2037.97	0.17	NA	NA	2424.10	789.65	0.25
4.1.2.5	5.00	6.00	2073.66	0.17	0.25	0.26	1854.10	627.33	0.29
6.3.4.13	1.00	1.00	2075.30	0.18	NA	NA	598.15	409.90	0.12
2.6.1.-	3.00	3.00	2078.88	0.25	0.31	NA	6391.45	3405.45	0.20
2.7.1.19	3.00	1.00	2096.73	0.15	0.52	0.48	12469.58	10796.85	0.17
1.1.1.3	1.00	2.00	2104.47	0.16	0.33	0.36	818.67	477.50	0.13
4.1.3.4	2.00	11.00	2106.15	0.17	0.49	0.49	4848.90	1250.95	0.23
2.4.2.7	3.00	4.00	2107.80	0.18	0.32	0.30	2234.51	871.76	0.18
4.1.1.39	1.00	5.00	2155.73	0.16	0.08	0.08	938.18	661.69	0.02
2.7.1.39	1.00	1.00	2161.47	0.21	0.30	0.28	393.20	171.88	0.04
6.2.1.3	3.00	5.00	2166.18	0.17	0.54	0.42	2641.69	776.49	0.22
3.5.3.11	1.00	1.00	2167.29	0.06	0.21	0.28	7563.60	4049.95	0.12
1.1.1.95	5.00	4.00	2176.72	0.15	NA	NA	3011.27	2607.15	0.20

2.7.8.1	5.00	3.00	2179.62	0.11	0.29	NA	2444.08	1785.13	0.13
4.1.1.15	15.00	4.00	2187.21	0.10	0.21	0.24	6362.53	2726.40	0.29
2.7.8.2	4.00	3.00	2195.17	0.11	0.29	NA	2444.08	1785.13	0.12
1.1.1.37	7.00	6.00	2200.84	0.17	0.31	0.30	3798.26	2162.44	0.14
2.3.1.168	1.00	4.00	2204.14	0.31	NA	NA	1921.10	500.85	0.21
1.2.1.44	4.00	6.00	2212.26	0.21	0.23	0.23	1760.01	830.45	0.25
2.3.1.15	2.00	1.00	2220.24	0.29	0.28	0.26	1412.46	826.44	0.16
5.3.1.6	5.00	5.00	2224.21	0.25	0.25	0.25	2466.98	1415.50	0.18
5.5.1.6	2.00	3.00	2244.00	0.24	0.11	0.38	1898.61	434.60	0.21
6.6.1.1	1.00	1.00	2244.00	0.12	0.40	0.25	5412.93	3776.74	0.19
2.3.3.10	14.00	3.00	2250.92	NA	0.44	0.52	2382.80	607.45	0.17
4.1.3.27	3.00	1.00	2276.00	0.20	0.11	0.10	1838.56	923.04	0.12
2.3.1.51	1.00	2.00	2279.24	NA	0.56	0.47	6809.55	55.15	0.49
2.4.1.21	1.00	1.00	2284.00	0.25	0.45	0.51	1053.95	530.58	0.15
1.14.99.-	2.00	3.00	2307.03	0.31	0.24	0.24	8093.24	2549.48	0.17
2.3.1.9	13.00	11.00	2324.20	0.10	0.30	0.36	3612.22	1239.44	0.16
2.4.2.18	2.00	1.00	2338.00	0.18	NA	NA	1436.65	1559.18	0.13
2.4.1.18	2.00	3.00	2346.00	0.14	0.28	0.27	1885.80	1538.88	0.11
3.5.4.10	2.00	3.00	2393.65	0.09	0.51	0.52	1346.10	609.35	0.14
5.3.1.24	2.00	2.00	2401.00	0.32	0.22	0.16	1969.70	652.35	0.04
2.7.7.13	1.00	3.00	2419.66	0.05	0.48	0.54	16223.50	2307.70	0.24
4.1.1.48	2.00	2.00	2460.00	0.21	0.26	0.25	1251.50	1961.50	0.13
5.4.2.8	2.00	2.00	2479.67	0.09	0.52	0.52	2316.40	1260.90	0.10
1.1.99.5	3.00	3.00	2489.26	0.12	0.45	0.55	1668.35	441.90	0.22
4.2.1.20	1.00	2.00	2515.00	0.14	0.23	0.24	364.35	233.20	0.15
2.1.1.68	4.00	6.00	2605.52	0.14	0.43	0.53	10772.65	3176.65	0.17
2.7.7.27	4.00	4.00	2651.00	0.20	0.33	0.33	1250.76	1188.60	0.17
1.8.1.4	3.00	5.00	2658.67	0.11	0.13	0.12	3345.53	1766.23	0.15
4.2.3.1	2.00	3.00	2669.59	0.14	NA	NA	4175.43	862.98	0.18
2.7.4.14	7.00	4.00	2678.99	0.13	0.41	0.37	932.97	486.20	0.11
4.2.1.91	6.00	8.00	2685.49	0.21	0.08	0.10	1093.89	588.29	0.16
1.2.4.4	6.00	4.00	2685.84	0.26	NA	NA	1101.03	337.28	0.23
3.6.1.-	2.00	1.00	2721.12	0.19	0.46	0.53	764.28	449.78	0.12
2.7.2.4	5.00	1.00	2730.47	0.11	0.10	0.12	3703.06	516.61	0.11
6.3.5.4	8.00	20.00	2768.31	0.11	0.25	0.14	2392.05	1570.73	0.18
2.1.2.2	11.00	8.00	2774.47	0.33	NA	0.58	259.30	156.75	0.07
4.1.2.25	1.00	2.00	2780.12	0.35	0.17	0.19	277.77	185.10	0.10
1.2.1.11	1.00	2.00	2789.47	0.10	0.49	0.43	2719.40	1649.25	0.15
6.3.4.5	7.00	9.00	2841.25	0.17	0.51	0.59	2067.85	822.90	0.13
1.14.11.-	1.00	4.00	2934.00	0.26	0.19	0.20	803.97	167.45	0.25
1.3.1.38	3.00	7.00	2942.36	0.12	0.52	0.55	3614.40	935.75	0.13
1.3.99.-	4.00	4.00	2949.58	0.25	0.40	0.42	1747.72	1330.06	0.13
1.14.99.7	1.00	2.00	3000.00	NA	NA	NA	5687.95	3587.55	0.29
2.5.1.54	4.00	1.00	3017.58	0.13	0.15	0.33	4905.08	2278.75	0.13
1.1.1.86	1.00	1.00	3025.75	0.08	0.55	0.48	8644.70	5610.10	0.08
3.5.1.-	4.00	5.00	3026.35	0.23	0.31	0.35	1021.38	746.43	0.09
2.6.1.57	7.00	11.00	3037.44	0.25	0.28	0.26	1120.65	500.48	0.18
2.7.1.31	6.00	5.00	3057.69	NA	NA	NA	4612.85	2781.55	0.16
4.2.3.4	1.00	1.00	3076.58	0.08	0.62	0.66	1536.80	1457.60	0.08
2.4.2.10	3.00	5.00	3084.19	0.15	0.49	0.49	NA	NA	0.01
4.2.1.9	1.00	5.00	3084.75	NA	0.52	NA	NA	NA	0.01
2.1.1.45	7.00	6.00	3091.82	0.14	0.17	0.16	372.62	58.93	0.10
4.2.1.10	2.00	1.00	3136.58	NA	0.53	NA	1579.10	840.85	0.12
2.7.4.3	3.00	3.00	3139.98	0.18	0.24	0.29	1876.05	835.49	0.11

1.1.1.25	1.00	2.00	3195.58	NA	0.52	NA	1579.10	840.85	0.10
2.7.7.9	6.00	14.00	3245.75	0.12	0.35	0.34	5209.17	1914.28	0.18
2.7.1.71	1.00	1.00	3253.58	0.31	0.34	0.33	1147.15	659.78	0.21
2.1.2.10	12.00	11.00	3269.07	0.19	0.34	0.35	4043.08	2637.90	0.24
2.7.1.-	5.00	4.00	3286.03	0.28	0.21	0.11	1703.47	1038.10	0.15
2.5.1.19	1.00	1.00	3312.58	0.16	0.22	0.26	129.10	78.75	0.05
4.3.2.1	6.00	9.00	3312.91	0.20	0.55	0.50	1102.40	555.40	0.15
2.5.1.15	3.00	2.00	3330.12	NA	NA	NA	81.98	44.83	0.07
4.2.3.5	1.00	3.00	3362.58	0.11	0.47	0.53	2124.80	1300.10	0.10
2.5.1.21	4.00	1.00	3366.00	0.13	0.27	0.26	777.15	720.28	0.12
1.5.99.6	2.00	1.00	3366.83	0.18	NA	NA	395.00	77.55	0.29
5.5.1.13	1.00	1.00	3385.00	0.32	0.48	0.44	74.15	38.65	0.08
2.3.1.74	5.00	2.00	3416.99	0.11	0.53	0.49	13390.50	4296.10	0.15
4.1.1.19	13.00	3.00	3425.78	0.20	0.26	0.28	7672.80	4425.70	0.21
4.2.3.19	1.00	1.00	3444.00	0.31	0.51	0.50	480.25	215.15	0.18
1.14.13.78	1.00	1.00	3503.00	NA	NA	NA	2461.75	451.70	0.22
2.6.1.45	9.00	5.00	3503.70	0.10	0.47	0.51	9193.90	8448.75	0.16
1.14.13.79	1.00	1.00	3562.00	0.21	0.28	0.26	1329.20	204.28	0.23
1.3.1.43	6.00	9.00	3593.98	0.28	NA	NA	538.95	267.73	0.15
3.5.1.2	10.00	19.00	3601.74	0.16	0.47	0.51	1251.60	702.80	0.17
6.3.1.2	10.00	19.00	3601.74	0.08	0.20	0.21	6694.59	3196.45	0.24
4.2.1.2	6.00	5.00	3612.85	0.07	0.27	0.26	1446.70	1131.10	0.07
1.14.11.12	1.00	3.00	3621.00	0.25	0.07	0.08	1069.51	44.32	0.24
3.2.1.26	11.00	8.00	3703.24	0.18	0.18	0.20	1802.64	875.79	0.21
2.1.2.3	11.00	9.00	3710.02	0.09	0.51	0.54	1346.10	609.35	0.14
1.1.1.1	4.00	2.00	3730.08	0.11	0.28	0.27	7473.40	4875.13	0.39
4.2.1.11	2.00	5.00	3743.36	0.09	0.54	0.51	3313.27	1927.48	0.13
6.3.2.17	9.00	9.00	3787.79	0.24	0.13	0.27	943.05	810.13	0.15
2.2.1.6	8.00	4.00	3795.58	NA	0.17	0.14	6007.28	1992.02	0.15
1.2.4.1	9.00	4.00	3929.31	0.11	0.36	0.37	4186.89	2024.24	0.18
2.5.1.16	4.00	3.00	3995.83	0.12	0.36	0.35	2799.62	1865.02	0.11
5.4.2.1	7.00	6.00	4018.50	0.12	0.34	0.38	2576.78	2172.98	0.12
6.3.4.4	7.00	8.00	4090.94	0.11	0.44	0.44	3006.25	1977.25	0.09
1.1.1.22	5.00	16.00	4108.78	0.07	0.11	0.12	2724.33	727.49	0.20
2.2.1.7	11.00	1.00	4113.40	0.14	0.15	0.34	2391.22	2099.25	0.22
1.1.1.267	1.00	1.00	4172.40	0.08	0.50	0.54	2709.05	1630.40	0.12
6.3.5.5	10.00	21.00	4196.28	0.18	0.55	0.57	2092.00	1164.60	0.12
6.2.1.12	5.00	6.00	4202.45	0.22	0.33	0.30	1784.65	559.07	0.25
2.7.7.60	1.00	1.00	4231.40	0.26	NA	0.50	1156.90	746.90	0.17
2.7.1.148	1.00	1.00	4290.40	0.20	NA	0.54	728.10	403.10	0.15
4.6.1.12	1.00	1.00	4349.40	NA	NA	NA	1936.15	1146.50	0.13
1.17.4.3	1.00	1.00	4408.40	0.09	0.51	0.54	4253.30	4288.00	0.13
1.17.1.2	1.00	5.00	4467.40	0.14	0.46	0.51	4176.85	3763.05	0.13
1.17.4.1	8.00	5.00	4505.84	0.10	0.40	0.43	2501.39	2427.03	0.11
5.1.3.6	3.00	5.00	4542.23	0.10	0.31	0.30	9714.02	1795.23	0.28
4.3.2.2	7.00	7.00	4542.81	0.21	0.22	NA	100.20	52.75	0.10
4.99.1.1	1.00	1.00	4572.00	0.16	NA	0.35	1543.27	1446.98	0.21
4.2.1.17	11.00	6.00	4649.72	0.18	0.39	0.48	2456.51	786.63	0.16
2.5.1.6	5.00	3.00	4737.88	0.04	0.27	0.24	12286.10	4721.01	0.14
2.7.4.8	6.00	5.00	4795.91	0.23	0.13	0.16	4201.95	689.92	0.23
2.7.6.1	5.00	6.00	4812.88	0.14	0.32	0.32	791.24	403.51	0.12
1.5.1.12	14.00	16.00	4917.27	0.11	0.45	0.56	1025.20	571.10	0.12
1.1.1.35	5.00	7.00	4976.46	0.13	0.55	0.50	3977.10	1061.50	0.18
2.1.1.13	12.00	9.00	5038.42	NA	NA	NA	593.45	238.60	0.18

4.1.1.65	1.00	3.00	5088.77	0.18	0.34	0.38	781.92	398.60	0.18
2.6.1.4	12.00	7.00	5634.96	0.08	0.28	0.28	6375.98	5552.88	0.18
3.1.1.3	4.00	7.00	5726.66	0.46	0.16	0.14	1108.04	302.45	0.21
4.1.1.49	6.00	8.00	5892.36	0.11	0.23	0.24	3422.65	681.25	0.08
1.1.1.29	11.00	7.00	5965.08	0.07	0.55	0.43	9634.25	11170.15	0.18
2.7.8.8	5.00	2.00	6132.91	NA	0.50	0.50	790.35	225.55	0.19
2.1.1.-	6.00	2.00	6261.54	0.17	0.42	0.45	5227.53	1396.89	0.22
3.1.3.-	3.00	4.00	6272.13	0.13	0.48	0.53	1619.05	883.05	0.13
5.4.99.5	4.00	5.00	6334.42	0.26	0.15	0.13	630.05	330.94	0.15
2.7.4.6	10.00	10.00	6486.44	0.16	0.30	0.33	4808.91	2664.76	0.16
4.1.1.37	1.00	1.00	6633.00	0.14	0.25	0.24	3103.23	1718.65	0.22
2.3.1.12	15.00	11.00	6671.04	0.17	0.29	0.33	2141.62	743.03	0.13
1.3.3.3	1.00	1.00	6692.00	0.15	0.27	0.22	1173.70	574.48	0.19
1.3.3.4	1.00	2.00	6751.00	0.18	0.46	0.49	1678.98	995.58	0.12
1.14.-.-	5.00	10.00	6805.57	0.16	0.27	0.36	6648.67	1368.25	0.19
2.3.1.16	14.00	17.00	6825.87	0.08	0.16	0.20	5337.07	2082.80	0.14
6.1.1.17	11.00	1.00	7025.00	0.16	0.25	0.26	1759.95	525.60	0.08
1.2.1.70	1.00	1.00	7084.00	0.15	0.16	0.19	2123.32	1108.52	0.27
5.4.3.8	1.00	1.00	7143.00	0.11	0.23	0.26	3702.25	1664.45	0.18
2.5.1.29	6.00	9.00	7201.19	0.27	0.14	0.09	572.97	185.50	0.16
4.2.1.24	1.00	1.00	7202.00	0.11	0.25	0.25	1604.70	1022.10	0.12
2.5.1.61	1.00	1.00	7261.00	0.17	0.64	0.50	4881.45	3245.70	0.17
4.2.1.75	1.00	2.00	7320.00	NA	NA	NA	917.90	597.50	0.15
3.1.3.4	2.00	7.00	7595.17	0.17	0.11	0.24	5024.38	440.31	0.29
2.6.1.16	7.00	3.00	7708.40	0.06	0.48	NA	287.45	112.05	0.21
2.7.1.1	13.00	14.00	7778.58	0.20	0.33	0.32	1745.60	1087.16	0.22
3.1.3.5	16.00	7.00	7921.63	0.16	0.51	0.29	559.25	376.52	0.12
2.6.1.19	5.00	5.00	7979.22	0.13	0.40	0.52	4630.00	4237.95	0.12
1.3.1.-	7.00	6.00	7991.46	0.22	0.34	0.36	4533.83	3262.12	0.26
2.7.7.12	8.00	18.00	8923.38	0.20	0.50	0.55	480.35	287.55	0.11
3.1.4.4	4.00	3.00	8988.68	0.16	0.20	0.24	1755.83	525.62	0.22
3.2.1.22	8.00	13.00	9108.10	0.20	0.53	0.53	1788.13	1065.18	0.16
6.2.1.1	15.00	12.00	10207.56	0.15	0.50	0.53	1475.15	897.85	0.14
2.3.3.8	17.00	14.00	10758.08	0.07	0.32	0.34	3066.99	951.56	0.13
2.3.3.1	17.00	14.00	10758.08	0.11	0.13	0.10	3139.70	816.38	0.35
4.3.1.19	9.00	11.00	11111.79	0.18	0.55	0.55	1090.20	544.75	0.15
2.3.3.9	22.00	15.00	11294.40	NA	NA	NA	14030.55	23.50	0.57
4.4.1.8	5.00	9.00	12425.81	0.18	0.23	0.26	3894.80	3148.35	0.22
2.4.1.-	11.00	5.00	12568.54	0.38	0.11	0.10	885.50	509.11	0.22
5.4.2.2	11.00	10.00	12740.85	0.10	0.37	0.36	2968.37	1821.20	0.16
2.1.2.1	16.00	18.00	13004.43	0.13	0.34	0.38	2423.84	1590.26	0.12
4.1.2.13	10.00	11.00	13880.41	0.09	0.26	0.27	6078.16	4828.52	0.19
1.1.1.-	11.00	16.00	15388.86	0.11	0.61	0.53	4692.65	1685.35	0.16
2.7.1.40	6.00	13.00	16912.70	0.10	0.20	0.21	2104.89	2064.49	0.12
2.2.1.1	18.00	18.00	17161.88	0.10	0.32	0.34	6447.05	3489.12	0.21
2.3.1.-	12.00	25.00	17780.40	0.14	0.24	0.23	2566.08	889.53	0.16
2.6.1.2	16.00	22.00	19285.64	0.09	0.16	0.13	3252.16	2781.91	0.16
2.5.1.-	6.00	9.00	20151.57	0.21	0.16	0.24	1393.76	1083.02	0.14
1.14.13.-	13.00	16.00	20511.76	0.76	0.18	0.11	1632.23	1067.29	0.20
1.2.1.3	10.00	8.00	21465.53	0.14	0.45	0.46	4155.77	1842.48	0.24
4.1.1.-	11.00	12.00	25262.11	0.14	0.15	0.16	1440.48	734.69	0.03
2.6.1.1	26.00	37.00	32257.63	0.15	0.38	0.38	2344.99	1139.96	0.14
