# Supplementary material for: Genes encoding hub and bottleneck enzymes of the Arabidopsis metabolic network preferentially retain homeologs through whole genome duplication
Source: BMC Evol Biol. 2010 May 18;10:145. doi: 10.1186/1471-2148-10-145 (PMC2880986; doi:10.1186/1471-2148-10-145)
Supplement: Additional file 2 — Table S2. 11 topological modules and their major functions. [file 1471-2148-10-145-S2.PDF]

Table S2. 11 topological modules and their major functions. The number in the bracket indicates the number of enzymes in the pathways.

Module	Intra-module interaction	Inter-module interaction	Functional Classification	Pathway Description	Enzymes
**1**	102	39	Carbohydrate Metabolism	Starch and sucrose metabolisms(18); Nucleotide sugars metabolisms(8); Galactose metabolisms(9); Ascorbate and aldarate metabolisms(2); Fructose and mannose metabolisms(3); Pentose and glucuronate interconversionss(2); Inositol phosphate metabolisms(1);	*2.7.7.44,2.4.1.12,5.1.3.2,2.4.1.203,1.1.1.22,2.7.7.9,3.13.1.1,2.4.1.15,4.1.1.35,4.2.1.76,5.1.3.6,2.7.7.12,4.2.1.46,2.4.1.215,3.2.1.4,2.4.1.13,5.1.3.5,2.4.1.14,2.7.7.27,2.4.1.1,1.10.99.3,2.4.1.115,3.2.1.26,2.4.1.123,3.2.1.28,2.4.1.25,3.2.1.22,2.4.1.132,2.4.1.18,1.14.13.90,2.7.7.13,2.7.8.15,2.7.7.10,2.4.1.67,2.4.1.21,3.2.1.2,2.4.1.82,3.1.3.24,2.4.1.-,2.7.1.6,4.2.1.47,2.7.1.108,2.4.1.83,1.13.99.1,3.2.1.1,1.1.1.271,2.4.1.117,3.1.3.12*
**2**	73	31	Lipid Metabolism	Glycerophospholipid metabolisms(16); Ether lipid metabolisms(5); alpha-Linolenic acid metabolisms(6); Glycerolipid metabolisms(7); Linoleic acid metabolisms(2); Sphingolipid metabolisms(2); Arachidonic acid metabolisms(1);	*4.2.3.1,2.6.1.52,4.3.1.19,2.3.1.30,2.1.1.103,1.2.1.11,4.2.1.52,3.1.3.3,2.7.8.11,1.1.1.3,6.1.1.11,2.7.8.8,2.7.7.41,2.3.1.50,2.7.1.39,1.3.1.26,2.7.8.5,2.7.1.32,2.7.7.15,2.3.1.51,5.3.99.6,1.13.11.12,1.3.1.42,3.1.1.4,3.1.1.32,4.1.1.65,3.1.2.2,4.2.1.92,2.7.8.1,2.4.1.46,3.1.4.11,3.1.3.4,2.4.1.241,2.3.1.158,2.4.1.184,3.1.1.3,2.7.8.2,2.7.7.14,2.3.1.20,3.1.4.4*
**3**	55	26	Amino Acid Metabolism	Tryptophan metabolisms(4); Phenylalanine, tyrosine and tryptophan biosynthesiss(4); Phenylalanine metabolisms(2); Tyrosine metabolisms(1); Urea cycle and metabolism of amino groupss(1);	1.14.-.-,1.14.13.36,2.3.1.74,1.8.5.1,2.3.1.133,1.3.-.-,1.2.1.44,2.3.1.91,2.3.1.92,2.4.1.120,2.1.1.104,4.2.1.20,1.10.3.3,1.11.1.11,4.1.1.48,5.3.1.24,2.4.2.18,6.2.1.12,1.14.13.11,3.5.5.1,3.2.1.147,4.2.1.84,4.99.1.6,3.5.1.4,1.13.11.5,1.2.1.68,1.1.1.195,1.14.13.-,2.1.1.68,2.4.1.111,2.1.1.64,1.6.5.3
			Biosynthesis of Secondary Metabolites	Phenylpropanoid biosynthesiss(7); Flavonoid biosynthesiss(3);	
**4**	60	34	Nucleotide Metabolism	Pyrimidine metabolisms(12); Purine metabolisms(10);	*2.7.7.18,2.7.1.,2.7.1.151,2.7.1.134,2.4.2.19,2.7.4.6,3.5.4.26,3.6.1.23,2.7.4.9,1.17.4.1,6.3.4.2,2.4.2.11,2.7.1.140,1.8.1.9,3.5.1.19,3.5.4.25,2.7.1.40,3.5.4.16,3.6.1.-,2.7.4.3,3.1.3.5,3.5.4.5,2.7.1.48,3.5.4.10,1.1.1.205,2.4.2.9,4.1.1.23,2.7.4.14,2.4.2.7,6.3.5.2,2.7.4.8*

**5**	48	44	Amino Acid Metabolism	Glycine, serine and threonine metabolisms(6); Glutamate metabolisms(1);	2.6.1.45,4.1.2.5,1.14.11.-,6.1.1.3,6.1.1.14,2.6.1.4,1.4.4.2,3.4.11.2,1.1.99.14,1.1.1.29,3.1.3.18,1.1.3.15,6.2.1.5,4.1.3.1,1.2.1.24,1.14.11.13,1.14.11.15,1.14.11.12,2.7.1.33,2.7.7.3,4.1.1.36,6.3.2.5,1.14.13.79,1.14.13.78,4.2.3.19,5.5.1.13
			Biosynthesis of Secondary Metabolites	Diterpenoid biosynthesis(7);	
			Carbohydrate Metabolism	Glyoxylate and dicarboxylate metabolisms(4); C5-Branched dibasic acid metabolisms(1); Propanoate metabolisms(1); Citrate cycle (TCA cycle)s(1); Butanoate metabolisms(1);	
**6**	80	69	Amino Acid Metabolism	Phenylalanine, tyrosine and tryptophan biosynthesiss(14); Methionine metabolisms(8); Tyrosine metabolisms(6); Urea cycle and metabolism of amino groupss(8); Phenylalanine metabolisms(4); Valine, leucine and isoleucine biosynthesiss(3); Histidine metabolis	*4.2.3.4,2.5.1.6,1.14.17.4,4.1.1.50,2.5.1.16,3.2.2.16,3.5.3.11,2.5.1.22,6.3.2.1,1.1.1.169,3.5.1.53,2.6.1.19,2.5.1.19,1.5.99.6,4.4.1.14,3.5.3.12,3.5.1.6,3.5.2.2,1.2.1.3,4.2.1.10,1.1.1.25,2.7.1.71,4.2.3.5,5.4.4.2,4.1.3.27,5.4.99.5,1.1.1.1,2.7.1.100,2.6.1.9,1.3.1.43,2.6.1.57,1.13.11.54,1.13.11.27,4.2.1.19,4.1.1.25,2.6.1.5,6.1.1.20,4.2.1.91,2.2.1.6,1.1.1.86,1.1.1.27,4.1.1.-,1.2.4.1*
**7**	208	63	Amino Acid Metabolism	Arginine and proline metabolisms(11); Alanine and aspartate metabolisms(13); Glutamate metabolisms(13); Urea cycle and metabolism of amino groupss(11); Tyrosine metabolisms(2); Cysteine metabolisms(1); Phenylalanine metabolisms(1); Lysine degradations(1);	*4.3.2.2,4.3.2.1,2.1.3.3,6.3.2.6,3.5.3.1,2.3.1.35,3.7.1.2,6.3.4.4,6.3.5.4,4.1.1.21,2.7.2.4,6.3.4.5,6.3.3.1,4.2.1.2,6.1.1.12,1.4.3.-,1.3.5.1,6.1.1.19,4.2.1.65,2.6.1.11,3.5.1.1,1.14.13.39,6.1.1.22,2.6.1.13,2.1.3.2,4.1.1.15,2.8.2.-,2.6.1.1,1.5.99.8,1.5.1.2,4.4.1.9,1.4.1.2,4.1.1.19,3.5.1.2,1.4.7.1,6.3.5.5,2.3.1.1,6.1.1.18,6.3.2.2,2.7.2.11,6.1.1.17,2.6.1.2,1.5.1.12,6.3.1.2,6.1.1.7,2.3.2.2,1.4.1.14,1.1.1.42,1.2.4.2,2.7.2.8,1.2.1.41*
**8**	239	63	Amino Acid Metabolism	Valine, leucine and isoleucine degradations(15); Lysine degradations(6); Valine, leucine and isoleucine biosynthesiss(4); Lysine biosynthesiss(4); Tryptophan metabolisms(3); Alanine and aspartate metabolisms(2); Glycine, serine and threonine metabolisms(1	*3.1.1.11,2.4.1.43,2.3.3.1,1.1.1.37,4.1.1.31,4.1.1.49,6.2.1.1,2.3.1.12,1.2.99.2,2.3.1.16,6.4.1.2,3.2.1.15,1.1.1.-,1.-.-.-,2.3.3.13,2.3.3.9,2.3.1.41,2.3.3.8,4.1.3.4,2.3.1.9,1.2.1.27,4.2.1.3,2.3.3.10,2.3.1.39,4.4.1.5,3.1.2.6,3.1.2.14,1.3.1.9,2.3.1.-,1.5.1.8,4.1.1.20,3.5.1.-,3.1.2.-,2.3.1.47,1.2.4.4,2.6.1.62,1.3.99.12,2.3.1.168,1.8.1.4,1.3.1.-,1.3.1.38,5.5.1.6,1.14.13.21,4.2.1.17,6.2.1.3,1.3.99.-,1.14.19.2,6.1.1.6,1.5.1.9,1.1.1.219,1.3.3.6,2.6.1.-,5.5.1.-,4.2.1.55,6.1.1.9,2.6.1.42,4.1.1.18,1.2.1.25,1.14.11.9,2.-.-.-,1.17.1.3,1.3.99.10,4.2.1.9,2.1.2.11,5.1.1.7,2.3.1.129,6.4.1.4,2.4.1.91,3.1.2.4,2.7.1.130,1.1.1.35,1.1.1.100,4.2.1.18,1.1.1.34,2.7.1.36*
			Carbohydrate Metabolism	Pyruvate metabolisms(12); Propanoate metabolisms(6); Citrate cycle (TCA cycle)s(6); Butanoate metabolisms(6); Glyoxylate and dicarboxylate metabolisms(4); Pentose and glucuronate interconversionss(2); Inositol metabolisms(1); Glycolysis / Gluconeogenesiss	
**9**	65	27	Metabolism of Cofactors and Vitamins	One carbon pool by folate(11); Folate biosynthesis(8);	*6.3.2.12,2.1.1.45,2.5.1.15,1.5.1.20,2.1.2.10,4.1.2.25,2.7.6.3,4.1.3.38,6.3.2.17,1.5.1.3,2.1.2.1,6.3.4.13,2.1.2.2,3.4.19.9,3.1.2.12,2.1.1.13,6.3.4.3,2.1.2.3,3.5.1.10,6.3.3.2*

**10**	134	47	Carbohydrate Metabolism	Pentose phosphate pathways(13); Fructose and mannose metabolisms(10); Glycolysis / Gluconeogenesiss(12); Aminosugars metabolisms(4); Pentose and glucuronate interconversionss(3); Galactose metabolisms(4); Starch and sucrose metabolisms(5); Inositol metabo	*5.3.1.8,2.7.1.11,5.4.2.8,2.7.1.1,2.7.1.2,2.7.1.4,5.3.1.9,2.6.1.16,2.4.2.10,1.3.99.11,3.5.2.3,2.7.1.90,1.1.1.49,5.4.2.2,2.3.1.4,2.7.6.1,5.4.2.3,2.4.2.14,2.4.2.17,3.1.3.11,3.1.3.-,5.3.1.5,2.5.1.3,3.6.1.31,2.7.4.7,3.5.4.19,5.3.1.16,3.1.1.31,5.4.2.1,2.7.1.31,4.1.1.39,1.1.1.95,2.7.2.3,4.2.1.11,2.2.1.2,2.2.1.7,4.1.2.13,5.3.1.1,1.2.1.13,1.2.1.12,1.1.99.5,3.1.3.37,2.7.1.30,2.3.1.15,2.5.1.54,2.2.1.1,2.7.1.19,5.1.3.1,5.3.1.6,2.7.1.17,1.1.1.44*
**11**	93	31	Amino Acid Metabolism	Methionine metabolisms(5); Cysteine metabolisms(3); Histidine metabolisms(2); Tryptophan metabolisms(1);	3.3.1.1,2.5.1.-,6.1.1.10,4.2.3.16,4.2.3.15,2.5.1.10,2.5.1.29,2.5.1.1,2.5.1.21,2.1.1.10,4.2.3.14,2.1.1.14,4.4.1.8,2.5.1.32,1.14.99.-,1.14.99.30,4.1.1.33,5.3.3.2,2.5.1.8,1.17.1.2,2.5.1.27,2.8.1.7,1.3.1.70,4.2.1.24,5.3.3.5,2.5.1.61,5.4.99.8,2.1.1.143,5.4.99.-,1.14.99.7,1.11.1.6,1.14.13.70,5.5.1.9,2.5.1.47,2.1.1.41,6.1.1.16,5.4.3.8,1.2.1.70,4.1.1.37,2.1.1.-,4.2.1.75,5.4.99.7,1.8.7.1,1.1.1.23,2.1.1.107,6.1.1.21,4.99.1.4,4.99.1.1,1.3.3.3,1.3.3.4,2.1.1.11,2.7.1.148,4.6.1.12,1.14.13.81,3.1.1.14,2.5.1.62,6.6.1.1,1.3.1.75,1.17.4.3,2.7.7.60,1.1.1.267
			Lipid Metabolism	Biosynthesis of steroidss(21);	
			Metabolism of Cofactors and Vitamins	Porphyrin and chlorophyll metabolisms(16); Thiamine metabolisms(1);	
