# Supplementary material for: Genes encoding hub and bottleneck enzymes of the Arabidopsis metabolic network preferentially retain homeologs through whole genome duplication
Source: BMC Evol Biol. 2010 May 18;10:145. doi: 10.1186/1471-2148-10-145 (PMC2880986; doi:10.1186/1471-2148-10-145)
Supplement: Additional file 3 — Table S3. The description of Arabidopsis microarry datasets. [file 1471-2148-10-145-S3.PDF]

Table S3. The *Arabidopsis* microarry datasets.

Types	Expression measurement	Datasets
**Developmental Stages**		**(3 replicates)**
cotyledons	7 days	ME00319/1A,1B,1C
hypocotyl	7 days	ME00319/2A,2B,2C
roots	7 days	ME00319/3A,3B,3C
shoot apex, vegetative + young leaves	7 days	ME00319/4A,4B,4C
leaves 1 + 2	7 days	ME00319/5A,5B,5C
shoot apex, vegetative	7 days	ME00319/6A,6B,6C
seedling, green parts	7 days	ME00319/7A2,7B2,7C2
shoot apex, transition (before bolting)	14 days	ME00319/8A,8B,8C
roots	17 days	ME00319/9A,9B,9C
rosette leaf # 4, 1cm long	10 days	ME00319/11A,11B,11C
rosette leaf # 2	17 days	ME00319/12A,12B,12C
rosette leaf # 4	17 days	ME00319/13A,13B,13C
rosette leaf # 6	17 days	ME00319/14A,14B,14C
rosette leaf # 8	17 days	ME00319/15A,15B,15C
rosette leaf # 10	17 days	ME00319/16A,16B,16C
rosette leaf # 12	17 days	ME00319/18A,18B,18C
Leaf 7, petiole	17 days	ME00319/19A,19B,19C
Leaf 7, proximal half	17 days	ME00319/20A,20B,20C
Leaf 7, distal half	17 days	ME00319/21A,21B,21C
entire rossette after transition, but before bolting	21 days	ME00319/22A,22B,22C
entire rossette after transition, but before bolting	22 days	ME00319/23A,23B,23C
entire rossette after transition, but before bolting	23 days	ME00319/24A,24B,24C
senescing leaves	35 days	ME00319/25A,25B,25C
cauline leaves	21+ days	ME00319/26A,26B,26C
stem, 2nd internode	21+ days	ME00319/27A,27B,27C
1st node	21+ days	ME00319/28A2,28B2,28C2
shoot apex, inflorescence after bolting	21+ days	ME00319/29A2,29B2,29C2
flowers stage 10	21+ days	ME00319/31A2,31B2,31C2
flowers stage 12, sepals	21+ days	ME00319/34A,34B,34C
flowers stage 12, petals	21+ days	ME00319/35A,35B,35C
flowers stage 12, stamens	21+ days	ME00319/36A,36B,36C
flowers stage 12, carpels	21+ days	ME00319/37A,37B,37C
flowers stage 15, pedicels	21+ days	ME00319/40A,40B,40C
flowers stage 15, sepals	21+ days	ME00319/41A,41B,41C
flowers stage 15, stamen	21+ days	ME00319/43A,43B,43C
flowers stage 15, carpels	21+ days	ME00319/45A,45B,45C
pollen	mature pollen	ME00319/73A,73B,73C
siliques, w/ seeds 3, mid globular to early heart	8 wk	ME00319/76A,76B,76C
siliques, w/ seeds 4, early heart to late heart embryos	8 wk	ME00319/77D,77E,77F
siliques, w/ seeds 5, late heart embryos to mid torpedo	8 wk	ME00319/78D,78E,78F
seeds, 6, w/o siliques, mid torpedo to late torpedo	8wk	ME00319/79A,79B,79C
seeds, 7, w/o siliques, late torpedo to early walking-stick	8wk	ME00319/81A,81B,81C
seeds, 8, w/o siliques, walking-stick to early curled cotyledons embryos	8wk	ME00319/82A,82B,82C

seeds, 9, w/o siliques, curled cotyledons to early green cotyledons embryos	8wk	ME00319/83A,83B,83C
seeds, 10, w/o siliques, green cotyledons embryos	8wk	ME00319/84A,84B,84C
vegetative rosette for phase change	7 days	ME00319/87A,87B,87C
vegetative rosette for phase change	14 days	ME00319/89A,89B,89C
vegetative rosette for phase change	21 days	ME00319/90A,90B,90C
leaf	15 days	ME00319/91A,91B,91C
flower	28 days	ME00319/92A,92B,92C
root	8 days	ME00319/93A,93B,93C
root, 1X MS agar	8 days	ME00319/94A,94B,94C
root, 1X MS agar, 1% sucrose	8 days	ME00319/95A,95B,95C
seedling, green parts, 1X MS agar	8 days	ME00319/96A,96B,96C
seedling, green parts, 1X MS agar, 1% sucrose	8 days	ME00319/97A,97B,97C
root, 1X MS agar	21 days	ME00319/98A,98B,98C
root, 1X MS agar, 1X MS agar, 1% sucrose	21 days	ME00319/99A,99B,99C
seedling, green parts, 1X MS agar, 1% sucrose	21 days	ME00319/100A,100B,100C
seedling, green parts, 1X MS agar	21 days	ME00319/101A,101B,101C

**Aboitic Stages**		**(2 replicates)**
Cold stress	Shoot, 0, 0.5h, 1h, 3h, 6h, 12h, 24h after treatment	ME00325
Genotixic stress	Shoot, 0, 0.5h, 1h, 3h, 6h, 12h, 24h after treatment	ME00326
Osmotic stress	Shoot, 0, 0.5h, 1h, 3h, 6h, 12h, 24h after treatment	ME00327
Salt stress	Shoot, 0, 0.5h, 1h, 3h, 6h, 12h, 24h after treatment	ME00328
Oxidative stress	Shoot, 0, 0.5h, 1h, 3h, 6h, 12h, 24h after treatment	ME00340
UV-B	Shoot, 0, 0.25h, 0.5h, 1h, 3h, 6h, 12h, 24h after treatment	ME00329
Wound	Shoot, 0, 0.25h, 0.5h, 1h, 3h, 6h, 12h, 24h after treatment	ME00330
Droought stress	Shoot, 0, 0.5h, 1h, 3h, 6h, 12h, 24h after treatment	ME00338
Heat	Shoot, 0, 0.25h, 0.5h, 1h, 3h, 6h, 12h, 24h after treatment	ME00339
