# Supplementary material for: Genes encoding hub and bottleneck enzymes of the Arabidopsis metabolic network preferentially retain homeologs through whole genome duplication
Source: BMC Evol Biol. 2010 May 18;10:145. doi: 10.1186/1471-2148-10-145 (PMC2880986; doi:10.1186/1471-2148-10-145)
Supplement: Additional file 4 — Table S4. The identified ortholog groups between Arabidopsis and Populus by phylogenetic trees. [file 1471-2148-10-145-S4.PDF]

Table S4. The identified ortholog groups between *Arabidopsis* and *Populus* by phylogenetic tree.

Arabidopsis metabolic gene	Ortholog groups	Orthologs in *Populus*
At1g01090	((At1g01090), (gw1.X.1458.1))	gw1.X.1458.1
At1g01120	((At1g01120), (fgenesh4_pg.C_LG_II001639))	fgenesh4_pg.C_LG_II001639
At1g01390	((At4g01070, At1g01420, At1g01390), (estExt_fgenesh4_pg.C_LG_II1536, estExt_Genewise1_v1.C_LG_XIV1619))	estExt_fgenesh4_pg.C_LG_II1536, estExt_Genewise1_v1.C_LG_XIV1619
At1g01420	((At4g01070, At1g01420, At1g01390), (estExt_fgenesh4_pg.C_LG_II1536, estExt_Genewise1_v1.C_LG_XIV1619))	estExt_fgenesh4_pg.C_LG_II1536, estExt_Genewise1_v1.C_LG_XIV1619
At1g01710	((At1g01710), (grail3.0035017001))	grail3.0035017001
At1g02000	((At1g02000), (grail3.0035002801))	grail3.0035002801
At1g02400	((At1g02400), (gw1.II.529.1, gw1.XIV.2239.1))	gw1.II.529.1, gw1.XIV.2239.1
At1g02500	((At4g01850, At1g02500), (estExt_fgenesh4_pm.C_LG_XIV0257, grail3.0050014702))	estExt_fgenesh4_pm.C_LG_XIV0257, grail3.0050014702
At1g02660	((At1g02660), (estExt_fgenesh4_pm.C_LG_II0883))	estExt_fgenesh4_pm.C_LG_II0883
At1g02730	((At1g02730), (eugene3.00021804, gw1.XIV.2418.1))	eugene3.00021804, gw1.XIV.2418.1
At1g02790	((At1g02790), (eugene3.00021787))	eugene3.00021787
At1g02810	((At1g02810), (fgenesh4_pm.C_LG_II000882))	fgenesh4_pm.C_LG_II000882
At1g03090	((At1g03090), (eugene3.00051206))	eugene3.00051206
At1g03310	((At1g03310), (gw1.163.8.1))	gw1.163.8.1
At1g03475	((At1g03475), (gw1.119.43.1))	gw1.119.43.1
At1g04290	((At1g04290), (estExt_fgenesh4_pg.C_LG_IV1060, eugene3.00040996))	estExt_fgenesh4_pg.C_LG_IV1060, eugene3.00040996
At1g04410	((At5g43330, At1g04410), (estExt_Genewise1_v1.C_2730019, eugene3.00081537))	estExt_Genewise1_v1.C_2730019, eugene3.00081537
At1g04920	((At1g04920), (fgenesh4_pm.C_LG_I000988, fgenesh4_pg.C_scaffold_88000008))	fgenesh4_pm.C_LG_I000988, fgenesh4_pg.C_scaffold_88000008
At1g05010	((At1g05010), (estExt_Genewise1_v1.C_1660131, eugene3.00110176))	estExt_Genewise1_v1.C_1660131, eugene3.00110176
At1g05160	((At1g05160), (gw1.XIV.1750.1))	gw1.XIV.1750.1
At1g05310	((At1g13450, At1g05310), (gw1.XVI.799.1))	gw1.XVI.799.1
At1g05560	((At1g05560, At1g05530), (eugene3.13510003))	eugene3.13510003
At1g05610	((At4g27710, At1g05610), (fgenesh4_pg.C_LG_VI000196))	fgenesh4_pg.C_LG_VI000196
At1g05680	((At1g05680), (fgenesh4_pm.C_scaffold_64000028, fgenesh4_pg.C_scaffold_1119000002))	fgenesh4_pm.C_scaffold_64000028, fgenesh4_pg.C_scaffold_1119000002

At1g05790	((At4g33330, At1g05790), (eugene3.00002133))	eugene3.00002133
At1g06130	((At1g06130), (fgenesh4_pg.C_LG_IX001432, estExt_Genewise1_v1.C_LG_IX1501))	fgenesh4_pg.C_LG_IX001432, estExt_Genewise1_v1.C_LG_IX1501
At1g06290	((At1g06310, At1g06290), (estExt_fgenesh4_pg.C_LG_II0001))	estExt_fgenesh4_pg.C_LG_II0001
At1g06310	((At1g06310, At1g06290), (estExt_fgenesh4_pg.C_LG_II0001))	estExt_fgenesh4_pg.C_LG_II0001
At1g06410	((At1g06410), (fgenesh4_pg.C_LG_IV000367))	fgenesh4_pg.C_LG_IV000367
At1g06550	((At1g06550), (eugene3.00020542))	eugene3.00020542
At1g06570	((At1g06570), (eugene3.00051120))	eugene3.00051120
At1g06780	((At2g30575, At1g06780), (estExt_fgenesh4_pm.C_LG_V0534))	estExt_fgenesh4_pm.C_LG_V0534
At1g06800	((At1g06800), (fgenesh4_pg.C_LG_I002888))	fgenesh4_pg.C_LG_I002888
At1g07240	((At1g07260, At1g07250, At1g07240), (eugene3.00091201))	eugene3.00091201
At1g07260	((At1g07260, At1g07250, At1g07240), (eugene3.00091201))	eugene3.00091201
At1g07780	((At5g05590, At1g29410, At1g07780), (estExt_Genewise1_v1.C_LG_XII0225, gw1.XVII.219.1))	estExt_Genewise1_v1.C_LG_XII0225, gw1.XVII.219.1
At1g08110	((At1g08110), (fgenesh4_kg.C_LG_IX000057))	fgenesh4_kg.C_LG_IX000057
At1g08200	((At1g08200), (grail3.0001015401))	grail3.0001015401
At1g08250	((At5g50850, At1g08250), (estExt_fgenesh4_pm.C_LG_I0194))	estExt_fgenesh4_pm.C_LG_I0194
At1g08480	((At1g08480), (gw1.XIX.1648.1, gw1.I.7711.1))	gw1.XIX.1648.1, gw1.I.7711.1
At1g08510	((At1g08510), (estExt_fgenesh4_pm.C_LG_VI0445))	estExt_fgenesh4_pm.C_LG_VI0445
At1g08520	((At1g08520), (gw1.IX.3663.1))	gw1.IX.3663.1
At1g08550	((At1g08550), (estExt_Genewise1_v1.C_LG_XIII1884))	estExt_Genewise1_v1.C_LG_XIII1884
At1g08630	((At1g08630), (estExt_fgenesh4_pg.C_LG_XIX0195, estExt_Genewise1_v1.C_LG_XIII1664))	estExt_fgenesh4_pg.C_LG_XIX0195, estExt_Genewise1_v1.C_LG_XIII1664
At1g08980	((At1g08980), (grail3.0016020901))	grail3.0016020901
At1g09430	((At1g09430), (estExt_fgenesh4_pg.C_1310041, estExt_fgenesh4_pg.C_LG_XIII0014))	estExt_fgenesh4_pg.C_1310041, estExt_fgenesh4_pg.C_LG_XIII0014
At1g09780	((At1g09780), (estExt_fgenesh4_pg.C_1660043))	estExt_fgenesh4_pg.C_1660043
At1g09795	((At1g09795), (eugene3.00101416, eugene3.00080964))	eugene3.00101416, eugene3.00080964
At1g09830	((At1g09830), (estExt_Genewise1_v1.C_LG_I0264))	estExt_Genewise1_v1.C_LG_I0264
At1g09940	((At1g58290, At1g09940), (estExt_fgenesh4_pg.C_LG_II0918))	estExt_fgenesh4_pg.C_LG_II0918
At1g10070	((At1g10070), (gw1.XVIII.2623.1))	gw1.XVIII.2623.1
At1g10670	((At1g60810, At1g10670), (estExt_fgenesh4_pm.C_LG_VIII0801))	estExt_fgenesh4_pm.C_LG_VIII0801

At1g10700	((At1g10700), (gw1.X.2067.1))	gw1.X.2067.1
At1g11580	((At1g11580), (estExt_fgenesh4_pg.C_LG_XI0229))	estExt_fgenesh4_pg.C_LG_XI0229
At1g11680	((At1g11680), (fgenesh4_pg.C_LG_I000592, eugene3.00031308))	fgenesh4_pg.C_LG_I000592, eugene3.00031308
At1g11720	((At1g11720), (gw1.IV.1616.1))	gw1.IV.1616.1
At1g11790	((At1g11790), (estExt_fgenesh4_pg.C_LG_IV0012))	estExt_fgenesh4_pg.C_LG_IV0012
At1g11840	((At1g11840), (estExt_Genewise1_v1.C_LG_IV1582))	estExt_Genewise1_v1.C_LG_IV1582
At1g11860	((At1g11860), (estExt_fgenesh4_pg.C_2520014))	estExt_fgenesh4_pg.C_2520014
At1g11870	((At1g11870), (gw1.XIV.1672.1))	gw1.XIV.1672.1
At1g12230	((At1g12230), (gw1.IX.2398.1))	gw1.IX.2398.1
At1g12240	((At1g12240), (estExt_fgenesh4_pg.C_LG_III0902))	estExt_fgenesh4_pg.C_LG_III0902
At1g12900	((At3g26650, At1g12900), (estExt_fgenesh4_pg.C_LG_XIV0805))	estExt_fgenesh4_pg.C_LG_XIV0805
At1g13250	((At1g13250), (fgenesh4_pg.C_LG_X001896))	fgenesh4_pg.C_LG_X001896
At1g13280	((At3g25770, At3g25760, At1g13280), (gw1.IV.4073.1))	gw1.IV.4073.1
At1g13440	((At4g39620, At3g04120, At1g13440), (estExt_fgenesh4_pg.C_LG_X0484, estExt_Genewise1_v1.C_LG_XII1463, estExt_Genewise1_v1.C_LG_I2848))	estExt_fgenesh4_pg.C_LG_X0484, estExt_Genewise1_v1.C_LG_XII1463, [illegible]
At1g13560	((At3g25585, At1g13560), (estExt_Genewise1_v1.C_LG_VIII2313, gw1.X.421.1))	estExt_Genewise1_v1.C_LG_VIII2313, gw1.X.421.1
At1g14290	((At1g14290, At1g69640), (estExt_fgenesh4_pm.C_LG_V0449, estExt_fgenesh4_pg.C_LG_II0572))	estExt_fgenesh4_pm.C_LG_V0449, estExt_fgenesh4_pg.C_LG_II0572
At1g14520	((At1g14520), (estExt_Genewise1_v1.C_1450155))	estExt_Genewise1_v1.C_1450155
At1g14810	((At1g14810), (eugene3.00100987, eugene3.00081272))	eugene3.00100987, eugene3.00081272
At1g15080	((At1g15080), (gw1.X.37.1))	gw1.X.37.1
At1g15110	((At1g15110), (eugene3.00081172))	eugene3.00081172
At1g15380	((At1g80160, At1g15380), (grail3.0084001901))	grail3.0084001901
At1g15550	((At1g15550), (fgenesh4_pg.C_LG_III000353))	fgenesh4_pg.C_LG_III000353
At1g15710	((At5g01690, At4g01770, At4g01750, At4g01220, At1g15710), (gw1.97.33.1))	gw1.97.33.1
At1g16300	((At1g42970, At1g16300), (gw1.II.3569.1))	gw1.II.3569.1
At1g16350	((At1g79470, At1g16350), (estExt_fgenesh4_pg.C_42040001, fgenesh4_pg.C_LG_X001563, fgenesh4_pg.C_LG_VIII000715))	estExt_fgenesh4_pg.C_42040001, fgenesh4_pg.C_LG_X001563, [illegible]
At1g16700	((At1g79010, At1g16700), (estExt_fgenesh4_pm.C_LG_IX0254))	estExt_fgenesh4_pm.C_LG_IX0254
At1g17050	((At1g17050), (eugene3.00012780, eugene3.00012778))	eugene3.00012780, eugene3.00012778
At1g17420	((At1g17420), (gw1.I.2683.1))	gw1.I.2683.1

At1g17890	((At1g17890), (fgenesh4_pg.C_scaffold_252000007, grail3.0111002302))	fgenesh4_pg.C_scaffold_252000007, grail3.0111002302
At1g17960	((At5g44550, At4g20390, At1g17960), (estExt_Genewise1_v1.C_LG_XIV3629, grail3.0018041501))	estExt_Genewise1_v1.C_LG_XIV3629, grail3.0018041501
At1g18460	((At1g73920, At1g18460), (estExt_fgenesh4_pg.C_LG_XII0523))	estExt_fgenesh4_pg.C_LG_XII0523
At1g18500	((At1g74040, At1g18500), (estExt_Genewise1_v1.C_LG_XIV0085))	estExt_Genewise1_v1.C_LG_XIV0085
At1g18590	((At1g18590), (gw1.XVI.341.1))	gw1.XVI.341.1
At1g18870	((At1g18870), (gw1.XI.1735.1))	gw1.XI.1735.1
At1g19170	((At1g19170), (estExt_Genewise1_v1.C_LG_XVIII0730, grail3.0013022501))	estExt_Genewise1_v1.C_LG_XVIII0730, grail3.0013022501
At1g20050	((At1g20050), (eugene3.00031298))	eugene3.00031298
At1g20330	((At1g20330), (estExt_fgenesh4_pg.C_LG_II0150, eugene3.00051503))	estExt_fgenesh4_pg.C_LG_II0150, eugene3.00051503
At1g20510	((At1g20510), (estExt_fgenesh4_pm.C_LG_XII0345))	estExt_fgenesh4_pm.C_LG_XII0345
At1g20575	((At1g20575), (estExt_Genewise1_v1.C_LG_II0203))	estExt_Genewise1_v1.C_LG_II0203
At1g21440	((At1g21440), (gw1.I.8260.1))	gw1.I.8260.1
At1g22340	((At1g22400, At1g22380, At1g22340), (gw1.XVII.811.1))	gw1.XVII.811.1
At1g22400	((At1g22400, At1g22380, At1g22340), (gw1.XVII.811.1))	gw1.XVII.811.1
At1g22410	((At1g22410), (estExt_fgenesh4_pm.C_LG_II0453, eugene3.00050687))	estExt_fgenesh4_pm.C_LG_II0453, eugene3.00050687
At1g22940	((At1g22940), (fgenesh4_pg.C_LG_II002252))	fgenesh4_pg.C_LG_II002252
At1g23190	((At1g23190), (estExt_fgenesh4_pm.C_LG_VIII0571, estExt_fgenesh4_pg.C_LG_X0989))	estExt_fgenesh4_pm.C_LG_VIII0571, estExt_fgenesh4_pg.C_LG_X0989
At1g23200	((At1g23200), (eugene3.00021511))	eugene3.00021511
At1g23310	((At1g70580, At1g23310), (estExt_fgenesh4_pm.C_LG_X0229, eugene3.00081764))	estExt_fgenesh4_pm.C_LG_X0229, eugene3.00081764
At1g23460	((At1g70500, At1g23460), (grail3.0013015401))	grail3.0013015401
At1g23760	((At1g23760), (fgenesh4_pm.C_LG_X000251))	fgenesh4_pm.C_LG_X000251
At1g23800	((At1g23800), (eugene3.00061514))	eugene3.00061514
At1g23870	((At1g60140, At1g23870), (eugene3.00150531))	eugene3.00150531
At1g24280	((At1g24280), (estExt_Genewise1_v1.C_LG_I7789))	estExt_Genewise1_v1.C_LG_I7789
At1g24360	((At1g24360), (estExt_Genewise1_v1.C_LG_X4893, eugene3.26170001))	estExt_Genewise1_v1.C_LG_X4893, eugene3.26170001
At1g25220	((At1g25220), (eugene3.00100957))	eugene3.00100957
At1g25350	((At1g25350), (gw1.X.163.1))	gw1.X.163.1
At1g27450	((At1g27450), (estExt_fgenesh4_pg.C_LG_XIV0085))	estExt_fgenesh4_pg.C_LG_XIV0085

At1g27680	((At1g27680), (grail3.0053016101))	grail3.0053016101
At1g29410	((At5g05590, At1g29410, At1g07780), (estExt_Genewise1_v1.C_LG_XII0225, gw1.XVII.219.1))	estExt_Genewise1_v1.C_LG_XII0225, gw1.XVII.219.1
At1g29880	((At1g29880), (estExt_fgenesh4_pm.C_LG_III0547, estExt_Genewise1_v1.C_LG_I7940))	estExt_fgenesh4_pm.C_LG_III0547, estExt_Genewise1_v1.C_LG_I7940
At1g30040	((At1g30040), (gw1.IV.685.1))	gw1.IV.685.1
At1g30120	((At2g34590, At1g30120), (estExt_Genewise1_v1.C_LG_VII0255))	estExt_Genewise1_v1.C_LG_VII0255
At1g30370	((At1g30370), (eugene3.00290085))	eugene3.00290085
At1g30530	((At1g30530), (eugene3.00131128))	eugene3.00131128
At1g30620	((At1g30620), (estExt_fgenesh4_pg.C_LG_XI1340, eugene3.00012996))	estExt_fgenesh4_pg.C_LG_XI1340, eugene3.00012996
At1g30820	((At1g30820), (estExt_Genewise1_v1.C_LG_III0729))	estExt_Genewise1_v1.C_LG_III0729
At1g31220	((At1g66520, At4g17360, At1g31220), (eugene3.00002342))	eugene3.00002342
At1g31860	((At1g31860), (estExt_fgenesh4_pm.C_1460004))	estExt_fgenesh4_pm.C_1460004
At1g32060	((At1g32060), (estExt_Genewise1_v1.C_290458, estExt_Genewise1_v1.C_LG_III2205))	estExt_Genewise1_v1.C_290458, estExt_Genewise1_v1.C_LG_III2205
At1g32200	((At1g32200), (estExt_Genewise1_v1.C_290280))	estExt_Genewise1_v1.C_290280
At1g32440	((At1g32440), (eugene3.01180041))	eugene3.01180041
At1g32470	((At1g32470), (eugene3.01180035))	eugene3.01180035
At1g34430	((At1g34430), (estExt_Genewise1_v1.C_LG_XI2803))	estExt_Genewise1_v1.C_LG_XI2803
At1g35250	((At1g68280, At1g35290, At1g35250, At2g16790), (estExt_Genewise1_v1.C_LG_XIX0814))	estExt_Genewise1_v1.C_LG_XIX0814
At1g35290	((At1g68280, At1g35290, At1g35250, At2g16790), (estExt_Genewise1_v1.C_LG_XIX0814))	estExt_Genewise1_v1.C_LG_XIX0814
At1g35580	((At1g35580), (eugene3.00410102, eugene3.00190739))	eugene3.00410102, eugene3.00190739
At1g36160	((At1g36160), (estExt_fgenesh4_pm.C_LG_II0424, eugene3.00050761))	estExt_fgenesh4_pm.C_LG_II0424, eugene3.00050761
At1g36370	((At1g36370, At1g22020), (fgenesh4_pm.C_LG_V000326, eugene3.00020834))	fgenesh4_pm.C_LG_V000326, eugene3.00020834
At1g41830	((At1g41830), (gw1.XIII.112.1))	gw1.XIII.112.1
At1g42970	((At1g42970, At1g16300), (gw1.II.3569.1))	gw1.II.3569.1
At1g43080	((At4g13760, At2g40310, At2g26620, At2g15470, At2g15460, At2g15450, At1g43100, At1g43090, At1g43080), (eugene3.00091271))	eugene3.00091271
At1g43090	((At4g13760, At2g40310, At2g26620, At2g15470, At2g15460, At2g15450, At1g43100, At1g43090, At1g43080), (eugene3.00091271))	eugene3.00091271
At1g43100	((At4g13760, At2g40310, At2g26620, At2g15470, At2g15460, At2g15450, At1g43100, At1g43090, At1g43080), (eugene3.00091271))	eugene3.00091271
At1g43670	((At1g43670), (estExt_Genewise1_v1.C_LG_V0287, grail3.0003059501))	estExt_Genewise1_v1.C_LG_V0287, grail3.0003059501
At1g43710	((At1g43710), (gw1.IX.4016.1))	gw1.IX.4016.1

At1g43800	((At1g43800), (estExt_fgenesh4_pg.C_1470039, estExt_Genewise1_v1.C_LG_XVIII2221))	estExt_fgenesh4_pg.C_1470039, estExt_Genewise1_v1.C_LG_XVIII2221
At1g45201	((At1g45201), (eugene3.00400311, gw1.40.800.1))	eugene3.00400311, gw1.40.800.1
At1g47420	((At1g47420), (eugene3.00400264))	eugene3.00400264
At1g47840	((At1g47840), (estExt_fgenesh4_pg.C_LG_I1452))	estExt_fgenesh4_pg.C_LG_I1452
At1g47990	((At1g47990), (fgenesh4_pg.C_LG_VIII000899))	fgenesh4_pg.C_LG_VIII000899
At1g48030	((At3g17240, At1g48030), (grail3.0010030801, gw1.X.713.1))	grail3.0010030801, gw1.X.713.1
At1g48100	((At1g48100), ())	
At1g48320	((At1g48320), (fgenesh4_pg.C_LG_XIV000522, fgenesh4_pg.C_LG_II001917))	fgenesh4_pg.C_LG_XIV000522, fgenesh4_pg.C_LG_II001917
At1g48600	((At1g48600), (gw1.XV.1021.1))	gw1.XV.1021.1
At1g48850	((At1g48850), (estExt_Genewise1_v1.C_LG_X2626))	estExt_Genewise1_v1.C_LG_X2626
At1g48860	((At5g35170, At1g48860), (gw1.XVIII.3392.1))	gw1.XVIII.3392.1
At1g49140	((At3g18410, At1g49140), (estExt_fgenesh4_pg.C_LG_XII0484, grail3.0082003901))	estExt_fgenesh4_pg.C_LG_XII0484, grail3.0082003901
At1g49430	((At1g49430), (estExt_fgenesh4_pm.C_LG_IX0278))	estExt_fgenesh4_pm.C_LG_IX0278
At1g49820	((At1g49820), (fgenesh4_pm.C_LG_I001056, estExt_Genewise1_v1.C_LG_IX2396))	fgenesh4_pm.C_LG_I001056, estExt_Genewise1_v1.C_LG_IX2396
At1g50200	((At1g50200), (eugene3.00050325))	eugene3.00050325
At1g50480	((At1g50480), (estExt_fgenesh4_pm.C_LG_I0033, estExt_Genewise1_v1.C_LG_III0099))	estExt_fgenesh4_pm.C_LG_I0033, estExt_Genewise1_v1.C_LG_III0099
At1g51410	((At1g51410), (estExt_fgenesh4_pg.C_LG_IX1058, estExt_fgenesh4_pg.C_LG_I1820))	estExt_fgenesh4_pg.C_LG_IX1058, estExt_fgenesh4_pg.C_LG_I1820
At1g51420	((At1g51420), (eugene3.00160593))	eugene3.00160593
At1g51440	((At5g18610, At1g51440), (grail3.0027010001))	grail3.0027010001
At1g51760	((At1g51780, At1g51760), (fgenesh4_pm.C_LG_II000277))	fgenesh4_pm.C_LG_II000277
At1g51780	((At1g51780, At1g51760), (fgenesh4_pm.C_LG_II000277))	fgenesh4_pm.C_LG_II000277
At1g52570	((At3g15730, At1g52570), (estExt_fgenesh4_pm.C_LG_I0571, fgenesh4_pm.C_scaffold_44000016))	estExt_fgenesh4_pm.C_LG_I0571, fgenesh4_pm.C_scaffold_44000016
At1g53240	((At3g15020, At1g53240), (estExt_fgenesh4_pg.C_LG_XI0680))	estExt_fgenesh4_pg.C_LG_XI0680
At1g53310	((At3g14940, At1g53310), (estExt_Genewise1_v1.C_LG_XI3607))	estExt_Genewise1_v1.C_LG_XI3607
At1g53830	((At3g14310, At1g53830), (eugene3.00011934))	eugene3.00011934
At1g53840	((At1g53840), (gw1.I.5056.1))	gw1.I.5056.1
At1g53920	((At1g53920), (estExt_fgenesh4_pm.C_LG_III0548, gw1.28.867.1))	estExt_fgenesh4_pm.C_LG_III0548, gw1.28.867.1
At1g53990	((At2g25570, At1g53990), (gw1.64.134.1, gw1.X.4133.1))	gw1.64.134.1, gw1.X.4133.1

At1g55020	((At1g55020), (estExt_Genewise1_v1.C_LG_XIII0990))	estExt_Genewise1_v1.C_LG_XIII0990
At1g55180	((At1g55180), (fgenesh4_pg.C_scaffold_44000079))	fgenesh4_pg.C_scaffold_44000079
At1g55510	((At1g55510), (gw1.X.2863.1))	gw1.X.2863.1
At1g55790	((At4g27870, At3g51140, At2g40600, At2g23820, At1g55790), (fgenesh4_pg.C_LG_III000798, gw1.28.69.1))	fgenesh4_pg.C_LG_III000798, gw1.28.69.1
At1g55810	((At1g55810), (estExt_fgenesh4_pg.C_LG_I3337, estExt_Genewise1_v1.C_1400168))	estExt_fgenesh4_pg.C_LG_I3337, estExt_Genewise1_v1.C_1400168
At1g55850	((At1g55850), (fgenesh4_pm.C_LG_I001167, eugene3.00060053))	fgenesh4_pm.C_LG_I001167, eugene3.00060053
At1g56560	((At1g56560), (gw1.XVI.617.1))	gw1.XVI.617.1
At1g56600	((At1g56600), (gw1.130.1.1, gw1.V.5114.1))	gw1.130.1.1, gw1.V.5114.1
At1g56710	((At5g47860, At4g25000, At1g56710), (grail3.0047003202, gw1.XIII.514.1))	grail3.0047003202, gw1.XIII.514.1
At1g58290	((At1g58290, At1g09940), (estExt_fgenesh4_pg.C_LG_II0918))	estExt_fgenesh4_pg.C_LG_II0918
At1g59900	((At1g61130, At1g59900), (eugene3.00010915, gw1.XVIII.2398.1))	eugene3.00010915, gw1.XVIII.2398.1
At1g60140	((At1g60140, At1g23870), (eugene3.00150531))	eugene3.00150531
At1g60440	((At1g60440), (eugene3.00002395))	eugene3.00002395
At1g60470	((At1g60470), (grail3.0009037801))	grail3.0009037801
At1g60590	((At1g60590), (estExt_fgenesh4_pg.C_LG_V1044, eugene3.00020688))	estExt_fgenesh4_pg.C_LG_V1044, eugene3.00020688
At1g60600	((At1g60600), (eugene3.07260002, gw1.XVII.321.1))	eugene3.07260002, gw1.XVII.321.1
At1g60810	((At1g60810, At1g10670), (estExt_fgenesh4_pm.C_LG_VIII0801))	estExt_fgenesh4_pm.C_LG_VIII0801
At1g61720	((At2g18850, At1g61720), (gw1.XIII.1257.1))	gw1.XIII.1257.1
At1g62640	((At1g62640), (estExt_Genewise1_v1.C_LG_XIV2606))	estExt_Genewise1_v1.C_LG_XIV2606
At1g62960	((At1g62960), (eugene3.00030902))	eugene3.00030902
At1g63290	((At1g63290), (estExt_Genewise1_v1.C_LG_XVI1008))	estExt_Genewise1_v1.C_LG_XVI1008
At1g63660	((At1g63660, At1g01980), (estExt_fgenesh4_pm.C_LG_XI0449, grail3.0051010801))	estExt_fgenesh4_pm.C_LG_XI0449, grail3.0051010801
At1g63770	((At1g63770), (estExt_fgenesh4_pm.C_LG_III0416))	estExt_fgenesh4_pm.C_LG_III0416
At1g64190	((At5g41670, At1g64190), (fgenesh4_pm.C_LG_III000453, grail3.0008041101))	fgenesh4_pm.C_LG_III000453, grail3.0008041101
At1g65060	((At1g65060, At1g12110), (eugene3.00030833))	eugene3.00030833
At1g65570	((At1g65570), (estExt_fgenesh4_pg.C_LG_X1589))	estExt_fgenesh4_pg.C_LG_X1589
At1g65930	((At1g65930), (grail3.0038019202))	grail3.0038019202
At1g66200	((At5g37600, At1g66200), (estExt_fgenesh4_pm.C_LG_XII0003, estExt_fgenesh4_pg.C_1220090, estExt_Genewise1_v1.C_LG_II2125))	estExt_fgenesh4_pm.C_LG_XII0003, estExt_fgenesh4_pg.C_1220090, [illegible]

At1g66520	((At1g66520, At4g17360, At1g31220), (eugene3.00002342))	eugene3.00002342
At1g66530	((At1g66530, At4g26300), (estExt_Genewise1_v1.C_LG_II3255))	estExt_Genewise1_v1.C_LG_II3255
At1g67070	((At1g67070), (fgenesh4_pg.C_LG_IV000576))	fgenesh4_pg.C_LG_IV000576
At1g67090	((At5g38430, At5g38420, At5g38410, At1g67090), (gw1.XV.1174.1))	gw1.XV.1174.1
At1g67280	((At1g67280), (eugene3.01570036))	eugene3.01570036
At1g67980	((At1g67990, At1g67980), (estExt_fgenesh4_pg.C_LG_VIII1209, fgenesh4_pm.C_LG_X000399))	estExt_fgenesh4_pg.C_LG_VIII1209, fgenesh4_pm.C_LG_X000399
At1g68000	((At4g38570, At1g68000), (gw1.IV.1202.1))	gw1.IV.1202.1
At1g68010	((At1g68010), (estExt_fgenesh4_pg.C_LG_IV1470))	estExt_fgenesh4_pg.C_LG_IV1470
At1g68020	((At1g68020), (estExt_Genewise1_v1.C_LG_X6311, grail3.0010065002))	estExt_Genewise1_v1.C_LG_X6311, grail3.0010065002
At1g68750	((At1g68750), (eugene3.00081064))	eugene3.00081064
At1g69640	((At1g14290, At1g69640), (estExt_fgenesh4_pm.C_LG_V0449, estExt_fgenesh4_pg.C_LG_II0572))	estExt_fgenesh4_pm.C_LG_V0449, estExt_fgenesh4_pg.C_LG_II0572
At1g69740	((At1g69740), (eugene3.00020758))	eugene3.00020758
At1g69830	((At1g69830), (gw1.X.6063.1))	gw1.X.6063.1
At1g69940	((At5g07430, At5g07420, At5g07410, At1g69940), (gw1.142.161.1, gw1.XVII.471.1))	gw1.142.161.1, gw1.XVII.471.1
At1g70090	((At1g70090, At1g24170), (fgenesh4_pm.C_LG_X000512, fgenesh4_pg.C_LG_VIII001038, , , , , gw1.VII.3310.1))	fgenesh4_pm.C_LG_X000512, fgenesh4_pg.C_LG_VIII001038, , , , , [illegible]
At1g70290	((At1g70290), (fgenesh4_pm.C_LG_XII000278))	fgenesh4_pm.C_LG_XII000278
At1g70310	((At5g19530, At1g70310), (estExt_Genewise1_v1.C_LG_VI2502, eugene3.113020001))	estExt_Genewise1_v1.C_LG_VI2502, eugene3.113020001
At1g70500	((At1g70500, At1g23460), (grail3.0013015401))	grail3.0013015401
At1g70570	((At1g70570), (gw1.X.5255.1))	gw1.X.5255.1
At1g70580	((At1g70580, At1g23310), (estExt_fgenesh4_pm.C_LG_X0229, eugene3.00081764))	estExt_fgenesh4_pm.C_LG_X0229, eugene3.00081764
At1g70710	((At1g70710, At1g23210), (estExt_Genewise1_v1.C_LG_VIII0544))	estExt_Genewise1_v1.C_LG_VIII0544
At1g71170	((At1g71170), (fgenesh4_pg.C_LG_I001242))	fgenesh4_pg.C_LG_I001242
At1g71180	((At1g71180), (fgenesh4_pg.C_scaffold_11112000001))	fgenesh4_pg.C_scaffold_11112000001
At1g71920	((At5g10330, At1g71920), (gw1.XVI.1990.1))	gw1.XVI.1990.1
At1g72520	((At1g72520), (fgenesh4_pg.C_LG_III000259))	fgenesh4_pg.C_LG_III000259
At1g72550	((At1g72550), (estExt_fgenesh4_pg.C_1450059))	estExt_fgenesh4_pg.C_1450059
At1g72590	((At1g72590), (gw1.I.6795.1))	gw1.I.6795.1
At1g72680	((At1g72680), (gw1.XI.816.1))	gw1.XI.816.1

At1g72810	((At1g72810, At1g13630), (grail3.0033013101, gw1.X.498.1))	grail3.0033013101, gw1.X.498.1
At1g73250	((At1g73250), (estExt_Genewise1_v1.C_LG_XVIII3067, eugene3.00061335))	estExt_Genewise1_v1.C_LG_XVIII3067, eugene3.00061335
At1g73370	((At1g73370), (estExt_Genewise1_v1.C_1220111, eugene3.00120074))	estExt_Genewise1_v1.C_1220111, eugene3.00120074
At1g73920	((At1g73920, At1g18460), (estExt_fgenesh4_pg.C_LG_XII0523))	estExt_fgenesh4_pg.C_LG_XII0523
At1g74040	((At1g74040, At1g18500), (estExt_Genewise1_v1.C_LG_XIV0085))	estExt_Genewise1_v1.C_LG_XIV0085
At1g74090	((At1g74090), (fgenesh4_pg.C_LG_XIV000513, eugene3.00640114, gw1.VII.784.1))	fgenesh4_pg.C_LG_XIV000513, eugene3.00640114, gw1.VII.784.1
At1g74100	((At1g74100), (fgenesh4_pg.C_scaffold_124000004, eugene3.01240005, eugene3.00110262))	fgenesh4_pg.C_scaffold_124000004, eugene3.01240005, eugene3.00110262
At1g74320	((At1g74320), (gw1.I.6971.1))	gw1.I.6971.1
At1g74470	((At1g74470), (eugene3.00120625))	eugene3.00120625
At1g74710	((At1g74710, At1g24290), (eugene3.10840002, eugene3.00012168))	eugene3.10840002, eugene3.00012168
At1g74910	((At1g74910), (estExt_fgenesh4_pm.C_LG_XV0175))	estExt_fgenesh4_pm.C_LG_XV0175
At1g74960	((At4g00990, At3g26020, At1g74960), (gw1.XVIII.628.1))	gw1.XVIII.628.1
At1g75270	((At1g75270), (estExt_fgenesh4_pm.C_LG_X0841, eugene3.00080432))	estExt_fgenesh4_pm.C_LG_X0841, eugene3.00080432
At1g75330	((At1g75330), (fgenesh4_pg.C_scaffold_13902000001))	fgenesh4_pg.C_scaffold_13902000001
At1g75790	((At1g75790), (fgenesh4_pg.C_LG_I000006))	fgenesh4_pg.C_LG_I000006
At1g75900	((At1g75900), (estExt_Genewise1_v1.C_LG_V1864))	estExt_Genewise1_v1.C_LG_V1864
At1g76130	((At1g76130), (grail3.1829000101))	grail3.1829000101
At1g76490	((At1g76490), (gw1.XV.361.1))	gw1.XV.361.1
At1g76550	((At1g76550), (fgenesh4_pg.C_LG_V001718, eugene3.00020029))	fgenesh4_pg.C_LG_V001718, eugene3.00020029
At1g76690	((At1g76690), (gw1.XI.3877.1))	gw1.XI.3877.1
At1g77120	((At1g77120), (eugene3.00070374))	eugene3.00070374
At1g77590	((At1g77590), (grail3.0003071602))	grail3.0003071602
At1g78570	((At1g78570), (estExt_fgenesh4_pg.C_1680019))	estExt_fgenesh4_pg.C_1680019
At1g78580	((At1g78580), (estExt_fgenesh4_pg.C_1680018))	estExt_fgenesh4_pg.C_1680018
At1g78680	((At1g78680), (eugene3.00101302))	eugene3.00101302
At1g78955	((At1g78955, At1g78950), (fgenesh4_pg.C_scaffold_135000058, fgenesh4_pg.C_LG_VII001304))	fgenesh4_pg.C_scaffold_135000058, fgenesh4_pg.C_LG_VII001304
At1g79010	((At1g79010, At1g16700), (estExt_fgenesh4_pm.C_LG_IX0254))	estExt_fgenesh4_pm.C_LG_IX0254
At1g79460	((At3g29410, At1g79460), (eugene3.10760001))	eugene3.10760001

At1g79530	((At1g79530), (estExt_fgenesh4_pm.C_LG_VIII0332))	estExt_fgenesh4_pm.C_LG_VIII0332
At1g79550	((At1g79550), (grail3.0154005402, eugene3.00080776))	grail3.0154005402, eugene3.00080776
At1g80160	((At1g80160, At1g15380), (grail3.0084001901))	grail3.0084001901
At1g80170	((At1g80170), (gw1.XIX.2897.1))	gw1.XIX.2897.1
At1g80340	((At1g80340, At1g21710), (eugene3.00161222))	eugene3.00161222
At1g80460	((At1g80460), (fgenesh4_pm.C_scaffold_44000008))	fgenesh4_pm.C_scaffold_44000008
At1g80600	((At1g80600), (eugene3.00070748))	eugene3.00070748
At1g80820	((At1g80820), (estExt_fgenesh4_kg.C_LG_III0056))	estExt_fgenesh4_kg.C_LG_III0056
At2g01140	((At2g01140), (estExt_fgenesh4_pm.C_LG_VIII0532, estExt_fgenesh4_pg.C_LG_X1062))	estExt_fgenesh4_pm.C_LG_VIII0532, estExt_fgenesh4_pg.C_LG_X1062
At2g01290	((At2g01290), (estExt_Genewise1_v1.C_LG_VIII0741))	estExt_Genewise1_v1.C_LG_VIII0741
At2g01350	((At2g01350), (gw1.V.1402.1))	gw1.V.1402.1
At2g02050	((At2g02050), (estExt_fgenesh4_pg.C_LG_X0909, grail3.0010069702))	estExt_fgenesh4_pg.C_LG_X0909, grail3.0010069702
At2g02500	((At2g02500), (estExt_fgenesh4_pg.C_LG_II2226))	estExt_fgenesh4_pg.C_LG_II2226
At2g04400	((At2g04400), (eugene3.00130881))	eugene3.00130881
At2g05710	((At2g05710), (estExt_fgenesh4_pg.C_LG_II2062, gw1.XIV.3318.1))	estExt_fgenesh4_pg.C_LG_II2062, gw1.XIV.3318.1
At2g05990	((At2g05990), (grail3.0116012802, eugene3.00160402, eugene3.00031909))	grail3.0116012802, eugene3.00160402, eugene3.00031909
At2g06050	((At5g20380, At2g06050), (gw1.X.5887.1))	gw1.X.5887.1
At2g06925	((At2g06925), (gw1.XIV.3411.1))	gw1.XIV.3411.1
At2g07050	((At2g07050), (fgenesh4_pg.C_scaffold_164000040, estExt_Genewise1_v1.C_LG_VI1514))	fgenesh4_pg.C_scaffold_164000040, estExt_Genewise1_v1.C_LG_VI1514
At2g13360	((At2g13360), (estExt_fgenesh4_pg.C_LG_I1607))	estExt_fgenesh4_pg.C_LG_I1607
At2g15230	((At2g15230), (gw1.I.2997.1))	gw1.I.2997.1
At2g15450	((At4g13760, At2g40310, At2g26620, At2g15470, At2g15460, At2g15450, At1g43100, At1g43090, At1g43080), (eugene3.00091271))	eugene3.00091271
At2g15460	((At4g13760, At2g40310, At2g26620, At2g15470, At2g15460, At2g15450, At1g43100, At1g43090, At1g43080), (eugene3.00091271))	eugene3.00091271
At2g15470	((At4g13760, At2g40310, At2g26620, At2g15470, At2g15460, At2g15450, At1g43100, At1g43090, At1g43080), (eugene3.00091271))	eugene3.00091271
At2g15490	((At2g15490), (eugene3.12010001))	eugene3.12010001
At2g16370	((At2g16370), (estExt_Genewise1_v1.C_LG_V5276))	estExt_Genewise1_v1.C_LG_V5276
At2g16500	((At2g16500), (gw1.I.164.1))	gw1.I.164.1
At2g16530	((At2g16530), (fgenesh4_pg.C_LG_VIII000107))	fgenesh4_pg.C_LG_VIII000107

At2g16790	((At1g68280, At1g35290, At1g35250, At2g16790), (estExt_Genewise1_v1.C_LG_XIX0814))	estExt_Genewise1_v1.C_LG_XIX0814
At2g17265	((At2g17265), (fgenesh4_pg.C_scaffold_66000196))	fgenesh4_pg.C_scaffold_66000196
At2g17420	((At5g51550, At4g35460, At2g26600, At2g17420), (estExt_fgenesh4_pg.C_LG_XVIII1099, eugene3.00140843))	estExt_fgenesh4_pg.C_LG_XVIII1099, eugene3.00140843
At2g17630	((At2g17630), (gw1.1541.2.1))	gw1.1541.2.1
At2g17640	((At2g17640, At1g06240), (grail3.0044012101))	grail3.0044012101
At2g18250	((At2g18250), (eugene3.00570163))	eugene3.00570163
At2g18560	((At2g18560), (gw1.IX.334.1))	gw1.IX.334.1
At2g18700	((At2g18700), (fgenesh4_pm.C_LG_XVIII000320, eugene3.00061363))	fgenesh4_pm.C_LG_XVIII000320, eugene3.00061363
At2g19450	((At2g19450), (estExt_Genewise1_v1.C_LG_VI1174, gw1.145.33.1))	estExt_Genewise1_v1.C_LG_VI1174, gw1.145.33.1
At2g19570	((At2g19570), (grail3.0996000101, grail3.0145001501))	grail3.0996000101, grail3.0145001501
At2g19590	((At2g19590), (estExt_Genewise1_v1.C_LG_X0681))	estExt_Genewise1_v1.C_LG_X0681
At2g19690	((At2g19690), (fgenesh4_pg.C_LG_I002303))	fgenesh4_pg.C_LG_I002303
At2g19860	((At4g29130, At2g19860), (estExt_fgenesh4_pg.C_LG_XVIII0751))	estExt_fgenesh4_pg.C_LG_XVIII0751
At2g20340	((At2g20340), (estExt_fgenesh4_pg.C_LG_II2533))	estExt_fgenesh4_pg.C_LG_II2533
At2g20360	((At2g20360), (estExt_fgenesh4_kg.C_LG_XIV0043, grail3.0021035002))	estExt_fgenesh4_kg.C_LG_XIV0043, grail3.0021035002
At2g20420	((At2g20420), (estExt_Genewise1_v1.C_LG_XIV0188))	estExt_Genewise1_v1.C_LG_XIV0188
At2g20810	((At2g20810), (fgenesh4_pg.C_scaffold_120000045))	fgenesh4_pg.C_scaffold_120000045
At2g21170	((At2g21170), (estExt_Genewise1_v1.C_LG_IX1362))	estExt_Genewise1_v1.C_LG_IX1362
At2g21330	((At4g38970, At2g21330), (estExt_fgenesh4_pm.C_LG_IX0211, fgenesh4_pm.C_LG_VII000464, estExt_Genewise1_v1.C_LG_IV0774))	estExt_fgenesh4_pm.C_LG_IX0211, fgenesh4_pm.C_LG_VII000464, [illegible]
At2g21610	((At2g21610), (eugene3.00011087, gw1.V.5398.1))	eugene3.00011087, gw1.V.5398.1
At2g21770	((At2g21770), (estExt_fgenesh4_pg.C_LG_VII0650, eugene3.00020623))	estExt_fgenesh4_pg.C_LG_VII0650, eugene3.00020623
At2g21790	((At2g21790), (estExt_fgenesh4_pg.C_LG_VII0648))	estExt_fgenesh4_pg.C_LG_VII0648
At2g21940	((At2g21940), (estExt_fgenesh4_pg.C_LG_VII0602, gw1.V.3099.1))	estExt_fgenesh4_pg.C_LG_VII0602, gw1.V.3099.1
At2g22250	((At2g22250), (eugene3.00070097))	eugene3.00070097
At2g22330	((At2g22330), (estExt_Genewise1_v1.C_LG_VIII1052, grail3.0022041801))	estExt_Genewise1_v1.C_LG_VIII1052, grail3.0022041801
At2g22570	((At2g22570), (estExt_fgenesh4_pg.C_LG_V0623))	estExt_fgenesh4_pg.C_LG_V0623
At2g22780	((At2g22780), (estExt_fgenesh4_pg.C_LG_VII1232))	estExt_fgenesh4_pg.C_LG_VII1232
At2g22910	((At2g22910), (gw1.V.528.1))	gw1.V.528.1

At2g23420	((At4g16310, At2g23420), (gw1.V.1634.1))	gw1.V.1634.1
At2g23890	((At2g23890), (gw1.X.3652.1))	gw1.X.3652.1
At2g23910	((At4g30470, At2g23910), (estExt_fgenesh4_pm.C_LG_VI0550, estExt_fgenesh4_pg.C_LG_XVIII0872))	estExt_fgenesh4_pm.C_LG_VI0550, estExt_fgenesh4_pg.C_LG_XVIII0872
At2g24210	((At2g24210), (gw1.234.14.1))	gw1.234.14.1
At2g24630	((At4g31590, At2g24630), (fgenesh4_pg.C_LG_VI001955, eugene3.00180589))	fgenesh4_pg.C_LG_VI001955, eugene3.00180589
At2g26080	((At4g33010, At2g26080), (estExt_fgenesh4_pm.C_LG_VI0678))	estExt_fgenesh4_pm.C_LG_VI0678
At2g26400	((At4g14716, At4g14710, At2g26400), (estExt_Genewise1_v1.C_LG_VIII0915))	estExt_Genewise1_v1.C_LG_VIII0915
At2g26440	((At2g26440), (gw1.XV.413.1))	gw1.XV.413.1
At2g26450	((At4g33230, At2g26450), (eugene3.00180192))	eugene3.00180192
At2g26560	((At5g04980, At2g26560), (fgenesh4_pg.C_LG_XVIII001217, gw1.XVII.1442.1))	fgenesh4_pg.C_LG_XVIII001217, gw1.XVII.1442.1
At2g26620	((At4g13760, At2g40310, At2g26620, At2g15470, At2g15460, At2g15450, At1g43100, At1g43090, At1g43080), (eugene3.00091271))	eugene3.00091271
At2g26800	((At2g26800), (estExt_fgenesh4_pm.C_LG_IX0450))	estExt_fgenesh4_pm.C_LG_IX0450
At2g26930	((At2g26930), (gw1.IX.3601.1, gw1.I.1482.1))	gw1.IX.3601.1, gw1.I.1482.1
At2g27450	((At2g27450), (grail3.0054007001))	grail3.0054007001
At2g27760	((At2g27760), (gw1.IX.724.1))	gw1.IX.724.1
At2g27820	((At2g27820), (gw1.IX.688.1))	gw1.IX.688.1
At2g27860	((At2g27860), (estExt_fgenesh4_pm.C_660024))	estExt_fgenesh4_pm.C_660024
At2g28420	((At2g28420), (gw1.XV.73.1))	gw1.XV.73.1
At2g28760	((At2g28760), (estExt_Genewise1_v1.C_LG_I1353))	estExt_Genewise1_v1.C_LG_I1353
At2g29560	((At2g29560), (gw1.IX.3951.1))	gw1.IX.3951.1
At2g29590	((At2g29590), (grail3.0001109301))	grail3.0001109301
At2g29690	((At4g08210, At3g48500, At3g06510, At2g47750, At2g29690), (gw1.XI.2977.1))	gw1.XI.2977.1
At2g30140	((At2g30140), (fgenesh4_pg.C_LG_IX000843, grail3.0053022001))	fgenesh4_pg.C_LG_IX000843, grail3.0053022001
At2g30490	((At2g30490), (estExt_fgenesh4_pg.C_LG_XIII0519))	estExt_fgenesh4_pg.C_LG_XIII0519
At2g30575	((At2g30575, At1g06780), (estExt_fgenesh4_pm.C_LG_V0534))	estExt_fgenesh4_pm.C_LG_V0534
At2g30770	((At2g30770, At2g30750), (gw1.123.295.1))	gw1.123.295.1
At2g30920	((At2g30920), (estExt_fgenesh4_pg.C_LG_II0371))	estExt_fgenesh4_pg.C_LG_II0371
At2g30970	((At2g30970), (estExt_fgenesh4_pg.C_LG_II0269))	estExt_fgenesh4_pg.C_LG_II0269

At2g31170	((At2g31170), (fgenesh4_pm.C_LG_X000211, eugene3.30420001))	fgenesh4_pm.C_LG_X000211, eugene3.30420001
At2g31350	((At5g65000, At2g31350), (estExt_Genewise1_v1.C_640417, estExt_Genewise1_v1.C_LG_VII0731))	estExt_Genewise1_v1.C_640417, estExt_Genewise1_v1.C_LG_VII0731
At2g31490	((At2g31490), (eugene3.00060644))	eugene3.00060644
At2g32090	((At2g32090), (estExt_fgenesh4_pg.C_LG_X0791))	estExt_fgenesh4_pg.C_LG_X0791
At2g32260	((At2g32260, At1g06620), (eugene3.00170489))	eugene3.00170489
At2g32440	((At2g32440), (estExt_fgenesh4_pm.C_LG_XIV0527, eugene3.00131319))	estExt_fgenesh4_pm.C_LG_XIV0527, eugene3.00131319
At2g32530	((At4g15320, At4g15290, At2g32620, At2g32610, At2g32540, At2g32530), (estExt_fgenesh4_pg.C_LG_II2039, gw1.XIV.3402.1))	estExt_fgenesh4_pg.C_LG_II2039, gw1.XIV.3402.1
At2g32540	((At4g15320, At4g15290, At2g32620, At2g32610, At2g32540, At2g32530), (estExt_fgenesh4_pg.C_LG_II2039, gw1.XIV.3402.1))	estExt_fgenesh4_pg.C_LG_II2039, gw1.XIV.3402.1
At2g32610	((At4g15320, At4g15290, At2g32620, At2g32610, At2g32540, At2g32530), (estExt_fgenesh4_pg.C_LG_II2039, gw1.XIV.3402.1))	estExt_fgenesh4_pg.C_LG_II2039, gw1.XIV.3402.1
At2g32620	((At4g15320, At4g15290, At2g32620, At2g32610, At2g32540, At2g32530), (estExt_fgenesh4_pg.C_LG_II2039, gw1.XIV.3402.1))	estExt_fgenesh4_pg.C_LG_II2039, gw1.XIV.3402.1
At2g33100	((At2g33100), (fgenesh4_pg.C_LG_III001500, fgenesh4_pg.C_LG_I000442))	fgenesh4_pg.C_LG_III001500, fgenesh4_pg.C_LG_I000442
At2g33150	((At2g33150), (fgenesh4_pm.C_LG_I000162))	fgenesh4_pm.C_LG_I000162
At2g33220	((At2g33220), (eugene3.00010448))	eugene3.00010448
At2g34555	((At2g34555), (estExt_fgenesh4_pg.C_LG_XI0670, eugene3.00012757))	estExt_fgenesh4_pg.C_LG_XI0670, eugene3.00012757
At2g34590	((At2g34590, At1g30120), (estExt_Genewise1_v1.C_LG_VII0255))	estExt_Genewise1_v1.C_LG_VII0255
At2g34630	((At2g34630), (estExt_fgenesh4_pg.C_280076))	estExt_fgenesh4_pg.C_280076
At2g35040	((At2g35040), (estExt_fgenesh4_pm.C_LG_X0439))	estExt_fgenesh4_pm.C_LG_X0439
At2g35120	((At2g35120), (estExt_fgenesh4_pg.C_LG_XII1299, estExt_Genewise1_v1.C_LG_XV2768))	estExt_fgenesh4_pg.C_LG_XII1299, estExt_Genewise1_v1.C_LG_XV2768
At2g35370	((At2g35370), (estExt_fgenesh4_pg.C_290176))	estExt_fgenesh4_pg.C_290176
At2g35390	((At2g35390), (eugene3.01180044))	eugene3.01180044
At2g35500	((At2g35500), (estExt_Genewise1_v1.C_LG_III1965))	estExt_Genewise1_v1.C_LG_III1965
At2g35690	((At4g16760, At2g35690), (estExt_fgenesh4_pm.C_290034))	estExt_fgenesh4_pm.C_290034
At2g35840	((At2g35840), (eugene3.00061137))	eugene3.00061137
At2g36230	((At2g36230), (, gw1.IV.3492.1))	, gw1.IV.3492.1
At2g36460	((At3g52930, At2g36460), (estExt_fgenesh4_pm.C_1210019, eugene3.00180798))	estExt_fgenesh4_pm.C_1210019, eugene3.00180798
At2g36530	((At2g36530), (eugene3.00151093))	eugene3.00151093
At2g36580	((At3g52990, At2g36580), (eugene3.00280289))	eugene3.00280289
At2g36710	((At2g36710), (fgenesh4_pg.C_scaffold_28000302, estExt_Genewise1_v1.C_LG_IX2261))	fgenesh4_pg.C_scaffold_28000302, estExt_Genewise1_v1.C_LG_IX2261

At2g36880	((At2g36880), (estExt_Genewise1_v1.C_280751))	estExt_Genewise1_v1.C_280751
At2g37250	((At2g37250, At1g71500, At1g28140), (estExt_fgenesh4_pg.C_LG_I0883, eugene3.00111119, gw1.70.218.1, gw1.XIII.2928.1))	estExt_fgenesh4_pg.C_LG_I0883, eugene3.00111119, gw1.70.218.1, [illegible]
At2g37500	((At2g37500), (gw1.VII.2836.1))	gw1.VII.2836.1
At2g37690	((At2g37690), (estExt_fgenesh4_pm.C_LG_XIII0184, fgenesh4_pm.C_LG_V000018))	estExt_fgenesh4_pm.C_LG_XIII0184, fgenesh4_pm.C_LG_V000018
At2g38040	((At2g38040), (eugene3.00060828))	eugene3.00060828
At2g38650	((At2g38650), (gw1.XVI.562.1))	gw1.XVI.562.1
At2g38700	((At3g54250, At2g38700), (grail3.0106013901, grail3.0090014401))	grail3.0106013901, grail3.0090014401
At2g39290	((At2g39290), (fgenesh4_pm.C_LG_I000710, gw1.IX.4341.1))	fgenesh4_pm.C_LG_I000710, gw1.IX.4341.1
At2g39630	((At2g39630), (estExt_fgenesh4_pg.C_LG_X1846, estExt_fgenesh4_pg.C_LG_VIII0457))	estExt_fgenesh4_pg.C_LG_X1846, estExt_fgenesh4_pg.C_LG_VIII0457
At2g39770	((At2g39770), (grail3.0022021203))	grail3.0022021203
At2g39930	((At2g39930), (fgenesh4_pg.C_LG_II002136, gw1.XVIII.258.1))	fgenesh4_pg.C_LG_II002136, gw1.XVIII.258.1
At2g40310	((At4g13760, At2g40310, At2g26620, At2g15470, At2g15460, At2g15450, At1g43100, At1g43090, At1g43080), (eugene3.00091271))	eugene3.00091271
At2g40490	((At2g40490), (estExt_fgenesh4_pg.C_LG_XIX0326))	estExt_fgenesh4_pg.C_LG_XIX0326
At2g40890	((At2g40890), (estExt_fgenesh4_pm.C_LG_VI0096))	estExt_fgenesh4_pm.C_LG_VI0096
At2g41530	((At2g41530), (grail3.0024031501))	grail3.0024031501
At2g41680	((At4g28700, At2g41680), (fgenesh4_pm.C_LG_XVIII000153, fgenesh4_pm.C_LG_II001196, grail3.0021033901))	fgenesh4_pm.C_LG_XVIII000153, fgenesh4_pm.C_LG_II001196, [illegible]
At2g41880	((At3g57550, At2g41880), (fgenesh4_pm.C_LG_XVI000165))	fgenesh4_pm.C_LG_XVI000165
At2g42010	((At2g42010), (fgenesh4_pg.C_LG_II001399))	fgenesh4_pg.C_LG_II001399
At2g42450	((At2g42450), (estExt_fgenesh4_pg.C_LG_II0377))	estExt_fgenesh4_pg.C_LG_II0377
At2g42690	((At5g01500, At2g42690, At4g18550), (fgenesh4_pm.C_LG_III000281))	fgenesh4_pm.C_LG_III000281
At2g42790	((At2g42790, At2g44350), (estExt_fgenesh4_pm.C_1480010))	estExt_fgenesh4_pm.C_1480010
At2g42910	((At2g42910), (estExt_fgenesh4_pg.C_LG_II0556, fgenesh4_pg.C_LG_V001185, gw1.164.104.1))	estExt_fgenesh4_pg.C_LG_II0556, fgenesh4_pg.C_LG_V001185, [illegible]
At2g43750	((At3g51280, At2g43750), (fgenesh4_pm.C_scaffold_57000031))	fgenesh4_pm.C_scaffold_57000031
At2g43820	((At2g43840, At2g43820), (estExt_Genewise1_v1.C_LG_VII0493, eugene3.13510003, gw1.VII.1165.1))	estExt_Genewise1_v1.C_LG_VII0493, eugene3.13510003, gw1.VII.1165.1
At2g43840	((At2g43840, At2g43820), (estExt_Genewise1_v1.C_LG_VII0493, eugene3.13510003, gw1.VII.1165.1))	estExt_Genewise1_v1.C_LG_VII0493, eugene3.13510003, gw1.VII.1165.1
At2g43890	((At2g43890), (eugene3.01700083, eugene3.00070023, gw1.XIX.575.1, gw1.XIII.1705.1))	eugene3.01700083, eugene3.00070023, gw1.XIX.575.1, gw1.XIII.1705.1
At2g44040	((At2g44040), (estExt_Genewise1_v1.C_LG_IV3834))	estExt_Genewise1_v1.C_LG_IV3834
At2g44160	((At3g59970, At2g44160), (estExt_fgenesh4_pg.C_1700003))	estExt_fgenesh4_pg.C_1700003

At2g44350	((At2g42790, At2g44350), (estExt_fgenesh4_pm.C_1480010))	estExt_fgenesh4_pm.C_1480010
At2g44520	((At2g44520), (fgenesh4_pg.C_LG_II001967, eugene3.31010003))	fgenesh4_pg.C_LG_II001967, eugene3.31010003
At2g44530	((At2g44530), (gw1.X.5251.1))	gw1.X.5251.1
At2g44810	((At2g44810), (gw1.40.286.1))	gw1.40.286.1
At2g45220	((At2g45220), (eugene3.00140126, eugene3.00021342))	eugene3.00140126, eugene3.00021342
At2g45290	((At3g60750, At2g45290), (eugene3.00021349))	eugene3.00021349
At2g45300	((At2g45300), (eugene3.00021350, gw1.XIV.746.1))	eugene3.00021350, gw1.XIV.746.1
At2g45440	((At2g45440), (estExt_fgenesh4_pg.C_LG_II1351))	estExt_fgenesh4_pg.C_LG_II1351
At2g45790	((At2g45790), (estExt_fgenesh4_pg.C_LG_XIV0248))	estExt_fgenesh4_pg.C_LG_XIV0248
At2g45970	((At2g45970), (gw1.XIV.1308.1))	gw1.XIV.1308.1
At2g46110	((At3g61530, At2g46110), (fgenesh4_pm.C_LG_XIV000150, eugene3.172360001, , gw1.II.2913.1, gw1.3273.2.1))	fgenesh4_pm.C_LG_XIV000150, eugene3.172360001, , gw1.II.2913.1, [illegible]
At2g46505	((At2g46505), (grail3.0046014601, gw1.IX.3020.1))	grail3.0046014601, gw1.IX.3020.1
At2g47030	((At3g62170, At2g47040, At2g47030), (fgenesh4_pg.C_LG_I001266, eugene3.00030089, eugene3.00030085, eugene3.00011207))	fgenesh4_pg.C_LG_I001266, eugene3.00030089, eugene3.00030085, [illegible]
At2g47040	((At3g62170, At2g47040, At2g47030), (fgenesh4_pg.C_LG_I001266, eugene3.00030089, eugene3.00030085, eugene3.00011207))	fgenesh4_pg.C_LG_I001266, eugene3.00030089, eugene3.00030085, [illegible]
At2g47180	((At2g47180), (estExt_fgenesh4_pm.C_LG_II0906, eugene3.00140617))	estExt_fgenesh4_pm.C_LG_II0906, eugene3.00140617
At2g47240	((At2g47240), (eugene3.00010532))	eugene3.00010532
At2g47510	((At2g47510), (eugene3.00021795))	eugene3.00021795
At2g47650	((At3g62830, At2g47650), (gw1.I.4975.1))	gw1.I.4975.1
At2g47690	((At2g47690), (grail3.0050002101))	grail3.0050002101
At3g01040	((At5g15470, At3g01040), (eugene3.00141554, eugene3.00140791))	eugene3.00141554, eugene3.00140791
At3g01120	((At3g01120), (gw1.V.1420.1))	gw1.V.1420.1
At3g01420	((At3g01420), (estExt_fgenesh4_pg.C_LG_VIII0936))	estExt_fgenesh4_pg.C_LG_VIII0936
At3g01640	((At3g01640), (eugene3.00012009))	eugene3.00012009
At3g02020	((At3g02020), (fgenesh4_pm.C_LG_I001164))	fgenesh4_pm.C_LG_I001164
At3g02100	((At3g02100), (eugene3.00141051))	eugene3.00141051
At3g02230	((At5g15650, At3g02230), (estExt_Genewise1_v1.C_LG_IV0305, grail3.0155001302))	estExt_Genewise1_v1.C_LG_IV0305, grail3.0155001302
At3g02350	((At3g02350), (eugene3.03090007))	eugene3.03090007
At3g02600	((At3g02600), (gw1.IV.3349.1))	gw1.IV.3349.1

At3g02630	((At3g02630), (fgenesh4_pm.C_LG_VIII000307))	fgenesh4_pm.C_LG_VIII000307
At3g02760	((At3g02760), (gw1.XIX.1762.1))	gw1.XIX.1762.1
At3g02780	((At5g16440, At3g02780), (fgenesh4_pm.C_LG_XIX000155, fgenesh4_pg.C_LG_III001573, eugene3.00031793))	fgenesh4_pm.C_LG_XIX000155, fgenesh4_pg.C_LG_III001573, [illegible]
At3g02870	((At3g02870), (fgenesh4_pm.C_LG_XVI000023, estExt_Genewise1_v1.C_LG_VI0423))	fgenesh4_pm.C_LG_XVI000023, estExt_Genewise1_v1.C_LG_VI0423
At3g02875	((At3g02875), (fgenesh4_pg.C_LG_VI001355, fgenesh4_pg.C_LG_VI001354))	fgenesh4_pg.C_LG_VI001355, fgenesh4_pg.C_LG_VI001354
At3g03050	((At5g16910, At3g03050), (estExt_fgenesh4_pg.C_870007, eugene3.00190332))	estExt_fgenesh4_pg.C_870007, eugene3.00190332
At3g03100	((At3g03100), (estExt_fgenesh4_pg.C_2580005))	estExt_fgenesh4_pg.C_2580005
At3g03250	((At3g03250), (eugene3.00440223))	eugene3.00440223
At3g03310	((At3g03310), (estExt_Genewise1_v1.C_LG_VIII1590))	estExt_Genewise1_v1.C_LG_VIII1590
At3g03780	((At5g17920, At3g03780), (estExt_fgenesh4_pg.C_LG_XIII0289, estExt_Genewise1_v1.C_LG_XIX1125))	estExt_fgenesh4_pg.C_LG_XIII0289, estExt_Genewise1_v1.C_LG_XIX1125
At3g04120	((At4g39620, At3g04120, At1g13440), (estExt_fgenesh4_pg.C_LG_X0484, estExt_Genewise1_v1.C_LG_XII1463, estExt_Genewise1_v1.C_LG_I2848))	estExt_fgenesh4_pg.C_LG_X0484, estExt_Genewise1_v1.C_LG_XII1463, [illegible]
At3g04790	((At3g04790), (gw1.V.701.1))	gw1.V.701.1
At3g04870	((At3g04870), (gw1.XIV.3183.1))	gw1.XIV.3183.1
At3g05620	((At3g05620), (gw1.133.141.1))	gw1.133.141.1
At3g06200	((At3g06200), (gw1.XV.2566.1))	gw1.XV.2566.1
At3g06260	((At3g06260), (fgenesh4_pg.C_LG_XII000713))	fgenesh4_pg.C_LG_XII000713
At3g06310	((At3g06310, At5g18800), (estExt_fgenesh4_pm.C_LG_X0097, estExt_Genewise1_v1.C_LG_VIII2811))	estExt_fgenesh4_pm.C_LG_X0097, estExt_Genewise1_v1.C_LG_VIII2811
At3g06580	((At3g06580), (estExt_fgenesh4_pg.C_LG_VIII0903, estExt_Genewise1_v1.C_LG_X0656))	estExt_fgenesh4_pg.C_LG_VIII0903, estExt_Genewise1_v1.C_LG_X0656
At3g06650	((At5g49460, At3g06650), (grail3.0010034901))	grail3.0010034901
At3g06850	((At3g06850, At1g06730), (fgenesh4_pg.C_LG_II000395))	fgenesh4_pg.C_LG_II000395
At3g06860	((At3g06860), (estExt_Genewise1_v1.C_LG_X3923, eugene3.00770104))	estExt_Genewise1_v1.C_LG_X3923, eugene3.00770104
At3g07270	((At3g07270), (eugene3.00141442, eugene3.00002525))	eugene3.00141442, eugene3.00002525
At3g07330	((At3g07330), (estExt_fgenesh4_pm.C_LG_II1148))	estExt_fgenesh4_pm.C_LG_II1148
At3g07420	((At3g07420), (estExt_fgenesh4_pm.C_LG_II1158))	estExt_fgenesh4_pm.C_LG_II1158
At3g07630	((At3g07630), (gw1.XIV.1999.1))	gw1.XIV.1999.1
At3g07820	((At5g48140, At3g07840, At3g07830, At3g07820), (fgenesh4_pm.C_LG_VII000366))	fgenesh4_pm.C_LG_VII000366
At3g07830	((At5g48140, At3g07840, At3g07830, At3g07820), (fgenesh4_pm.C_LG_VII000366))	fgenesh4_pm.C_LG_VII000366
At3g07840	((At5g48140, At3g07840, At3g07830, At3g07820), (fgenesh4_pm.C_LG_VII000366))	fgenesh4_pm.C_LG_VII000366

At3g07850	((At3g14040, At3g07850), (gw1.VII.274.1))	gw1.VII.274.1
At3g07970	((At3g07970, At4g01890), (fgenesh4_pg.C_LG_II001877))	fgenesh4_pg.C_LG_II001877
At3g08510	((At3g55940, At3g08510), (eugene3.00101828, eugene3.00080618))	eugene3.00101828, eugene3.00080618
At3g08590	((At3g08590), (estExt_fgenesh4_pg.C_LG_XVI1334, estExt_Genewise1_v1.C_281127))	estExt_fgenesh4_pg.C_LG_XVI1334, estExt_Genewise1_v1.C_281127
At3g08610	((At3g08610), (eugene3.00161359))	eugene3.00161359
At3g09930	((At4g39970, At3g09930), (gw1.XIV.2624.1, gw1.VII.1749.1, gw1.IX.3754.1))	gw1.XIV.2624.1, gw1.VII.1749.1, gw1.IX.3754.1
At3g10050	((At3g10050), (estExt_fgenesh4_pg.C_280257))	estExt_fgenesh4_pg.C_280257
At3g10160	((At3g55630, At3g10160), (eugene3.00101917))	eugene3.00101917
At3g10230	((At3g10230), (grail3.0004004801, grail3.0024027901))	grail3.0004004801, grail3.0024027901
At3g10370	((At3g10370), (estExt_fgenesh4_pg.C_LG_X2045))	estExt_fgenesh4_pg.C_LG_X2045
At3g10710	((At3g10710), (eugene3.00102398, eugene3.00080087))	eugene3.00102398, eugene3.00080087
At3g10850	((At3g10850), (estExt_fgenesh4_pg.C_1250018))	estExt_fgenesh4_pg.C_1250018
At3g11340	((At3g11340), (gw1.X.1927.1))	gw1.X.1927.1
At3g11670	((At3g11670), (estExt_fgenesh4_pm.C_LG_XVI0236))	estExt_fgenesh4_pm.C_LG_XVI0236
At3g11750	((At5g03050, At3g11750), (fgenesh4_pg.C_LG_XIV000509, eugene3.00021717))	fgenesh4_pg.C_LG_XIV000509, eugene3.00021717
At3g11950	((At3g11950), (gw1.XVI.1986.1))	gw1.XVI.1986.1
At3g12120	((At3g12120), (grail3.0004009301, eugene3.00061217))	grail3.0004009301, eugene3.00061217
At3g12670	((At3g12670), (fgenesh4_pm.C_LG_VIII000317, gw1.X.1387.1))	fgenesh4_pm.C_LG_VIII000317, gw1.X.1387.1
At3g12780	((At3g12780), (estExt_fgenesh4_pm.C_LG_X0655, estExt_fgenesh4_pm.C_LG_VIII0335))	estExt_fgenesh4_pm.C_LG_X0655, estExt_fgenesh4_pm.C_LG_VIII0335
At3g13110	((At4g15240, At3g13110), (estExt_fgenesh4_pm.C_LG_I1139))	estExt_fgenesh4_pm.C_LG_I1139
At3g13790	((At3g13790, At3g13784, At1g55120), (eugene3.09880001, eugene3.00640067))	eugene3.09880001, eugene3.00640067
At3g14040	((At3g14040, At3g07850), (gw1.VII.274.1))	gw1.VII.274.1
At3g14075	((At3g14075), (estExt_fgenesh4_pg.C_LG_III0255))	estExt_fgenesh4_pg.C_LG_III0255
At3g14130	((At3g14150, At3g14130), (eugene3.00040312))	eugene3.00040312
At3g14225	((At5g40990, At3g14225), (fgenesh4_pg.C_LG_III000507))	fgenesh4_pg.C_LG_III000507
At3g14310	((At3g14310, At1g53830), (eugene3.00011934))	eugene3.00011934
At3g14360	((At3g14360), (fgenesh4_pg.C_LG_III000532))	fgenesh4_pg.C_LG_III000532
At3g14390	((At3g14390), (estExt_fgenesh4_pg.C_LG_IV1314, eugene3.00090483))	estExt_fgenesh4_pg.C_LG_IV1314, eugene3.00090483

At3g14415	((At3g14420, At3g14415), (estExt_fgenesh4_pg.C_LG_XI0914))	estExt_fgenesh4_pg.C_LG_XI0914
At3g14420	((At3g14420, At3g14415), (estExt_fgenesh4_pg.C_LG_XI0914))	estExt_fgenesh4_pg.C_LG_XI0914
At3g14930	((At3g51820, At3g14930), (gw1.XVIII.3460.1))	gw1.XVIII.3460.1
At3g14940	((At3g14940, At1g53310), (estExt_Genewise1_v1.C_LG_XI3607))	estExt_Genewise1_v1.C_LG_XI3607
At3g15020	((At3g15020, At1g53240), (estExt_fgenesh4_pg.C_LG_XI0680))	estExt_fgenesh4_pg.C_LG_XI0680
At3g15730	((At3g15730, At1g52570), (estExt_fgenesh4_pm.C_LG_I0571, fgenesh4_pm.C_scaffold_44000016))	estExt_fgenesh4_pm.C_LG_I0571, fgenesh4_pm.C_scaffold_44000016
At3g15850	((At3g15850), (gw1.XIX.482.1))	gw1.XIX.482.1
At3g16150	((At3g16150), (fgenesh4_pg.C_LG_II001124, estExt_Genewise1_v1.C_400931))	fgenesh4_pg.C_LG_II001124, estExt_Genewise1_v1.C_400931
At3g16175	((At3g16175), (eugene3.00030372))	eugene3.00030372
At3g16565	((At3g16565), (estExt_fgenesh4_pg.C_LG_X0299))	estExt_fgenesh4_pg.C_LG_X0299
At3g16785	((At3g16785), (estExt_fgenesh4_pm.C_LG_X0035))	estExt_fgenesh4_pm.C_LG_X0035
At3g16850	((At3g16850, At4g33440), (gw1.X.3724.1))	gw1.X.3724.1
At3g16950	((At4g16155, At3g16950), (gw1.X.574.1))	gw1.X.574.1
At3g17060	((At3g17060), (estExt_fgenesh4_pm.C_LG_VII0457))	estExt_fgenesh4_pm.C_LG_VII0457
At3g17240	((At3g17240, At1g48030), (grail3.0010030801, gw1.X.713.1))	grail3.0010030801, gw1.X.713.1
At3g17390	((At3g17390), (estExt_Genewise1_v1.C_LG_XIII0489))	estExt_Genewise1_v1.C_LG_XIII0489
At3g18030	((At3g18030), (estExt_Genewise1_v1.C_LG_IV1427, gw1.IX.946.1))	estExt_Genewise1_v1.C_LG_IV1427, gw1.IX.946.1
At3g19450	((At3g19450), (fgenesh4_pm.C_LG_III000695))	fgenesh4_pm.C_LG_III000695
At3g19820	((At3g19820, At1g03100), (estExt_fgenesh4_pg.C_LG_X1518, fgenesh4_pg.C_LG_VIII000736, eugene3.00080606))	estExt_fgenesh4_pg.C_LG_X1518, fgenesh4_pg.C_LG_VIII000736, [illegible]
At3g20040	((At3g20040), (fgenesh4_pm.C_LG_I000617, fgenesh4_pg.C_LG_IX001086))	fgenesh4_pm.C_LG_I000617, fgenesh4_pg.C_LG_IX001086
At3g20330	((At3g20330), (grail3.0028024201))	grail3.0028024201
At3g20480	((At3g20480), (gw1.XIII.2878.1))	gw1.XIII.2878.1
At3g21110	((At3g21110, At2g38320), (gw1.163.139.1))	gw1.163.139.1
At3g21240	((At3g21240), (fgenesh4_pg.C_LG_V001627, eugene3.00020113))	fgenesh4_pg.C_LG_V001627, eugene3.00020113
At3g21720	((At3g21720), (estExt_Genewise1_v1.C_640140, eugene3.00070229))	estExt_Genewise1_v1.C_640140, eugene3.00070229
At3g21760	((At3g21760), (fgenesh4_pg.C_scaffold_40000171, gw1.494.4.1))	fgenesh4_pg.C_scaffold_40000171, gw1.494.4.1
At3g21790	((At3g21790), (grail3.1094000101, eugene3.00160106))	grail3.1094000101, eugene3.00160106
At3g22200	((At3g22200), (grail3.0024013201))	grail3.0024013201

At3g22360	((At3g22360), (gw1.XV.292.1))	gw1.XV.292.1
At3g22370	((At3g22370), (eugene3.00101758, eugene3.00080687))	eugene3.00101758, eugene3.00080687
At3g22400	((At3g22400), (estExt_fgenesh4_pm.C_LG_VIII0652, estExt_fgenesh4_pg.C_LG_X0813))	estExt_fgenesh4_pm.C_LG_VIII0652, estExt_fgenesh4_pg.C_LG_X0813
At3g22740	((At3g22740), (grail3.0175003501))	grail3.0175003501
At3g22960	((At3g22960), (estExt_fgenesh4_pm.C_LG_VIII0682, estExt_fgenesh4_pg.C_LG_X0733))	estExt_fgenesh4_pm.C_LG_VIII0682, estExt_fgenesh4_pg.C_LG_X0733
At3g23580	((At3g23580), (eugene3.31660002))	eugene3.31660002
At3g23810	((At4g13940, At3g23810), (estExt_Genewise1_v1.C_LG_I3343, eugene3.00880022))	estExt_Genewise1_v1.C_LG_I3343, eugene3.00880022
At3g23820	((At5g65560, At3g23820), (estExt_fgenesh4_pg.C_LG_I2408, eugene3.00880019))	estExt_fgenesh4_pg.C_LG_I2408, eugene3.00880019
At3g23920	((At3g23920), (grail3.0064001202))	grail3.0064001202
At3g24090	((At3g24090, At4g34740), (gw1.XIX.1506.1))	gw1.XIX.1506.1
At3g24360	((At4g13360, At3g24360), (estExt_fgenesh4_pg.C_LG_VI1023, gw1.107.236.1))	estExt_fgenesh4_pg.C_LG_VI1023, gw1.107.236.1
At3g24503	((At3g24503), (fgenesh4_pm.C_LG_XVIII000233))	fgenesh4_pm.C_LG_XVIII000233
At3g25110	((At4g13050, At3g25110), (gw1.I.9590.1))	gw1.I.9590.1
At3g25530	((At3g25530), (estExt_fgenesh4_pm.C_LG_XIV0502))	estExt_fgenesh4_pm.C_LG_XIV0502
At3g25585	((At3g25585, At1g13560), (estExt_Genewise1_v1.C_LG_VIII2313, gw1.X.421.1))	estExt_Genewise1_v1.C_LG_VIII2313, gw1.X.421.1
At3g25760	((At3g25770, At3g25760, At1g13280), (gw1.IV.4073.1))	gw1.IV.4073.1
At3g25770	((At3g25770, At3g25760, At1g13280), (gw1.IV.4073.1))	gw1.IV.4073.1
At3g25780	((At3g25780), (, gw1.123.188.1))	, gw1.123.188.1
At3g25860	((At3g25860), (estExt_fgenesh4_pg.C_LG_VIII0227))	estExt_fgenesh4_pg.C_LG_VIII0227
At3g25900	((At3g25900), (estExt_fgenesh4_pg.C_LG_X1132))	estExt_fgenesh4_pg.C_LG_X1132
At3g26380	((At3g26380), (eugene3.00141394))	eugene3.00141394
At3g26610	((At3g26610), (eugene3.00081775))	eugene3.00081775
At3g26650	((At3g26650, At1g12900), (estExt_fgenesh4_pg.C_LG_XIV0805))	estExt_fgenesh4_pg.C_LG_XIV0805
At3g26900	((At3g26900), (estExt_fgenesh4_pg.C_LG_III0749))	estExt_fgenesh4_pg.C_LG_III0749
At3g27060	((At3g27060), (fgenesh4_pg.C_LG_I002334, estExt_Genewise1_v1.C_880196))	fgenesh4_pg.C_LG_I002334, estExt_Genewise1_v1.C_880196
At3g27190	((At5g40870, At3g27190), (fgenesh4_pg.C_scaffold_88000094))	fgenesh4_pg.C_scaffold_88000094
At3g27300	((At5g40760, At3g27300), (estExt_Genewise1_v1.C_LG_XVII0625, grail3.0054015801))	estExt_Genewise1_v1.C_LG_XVII0625, grail3.0054015801
At3g27440	((At3g27440), (gw1.V.687.1))	gw1.V.687.1

At3g27740	((At3g27740), (estExt_fgenesh4_pg.C_290028, gw1.IV.1772.1))	estExt_fgenesh4_pg.C_290028, gw1.IV.1772.1
At3g29090	((At3g29090), (estExt_fgenesh4_pg.C_LG_IV0964))	estExt_fgenesh4_pg.C_LG_IV0964
At3g29200	((At3g29200), (, gw1.VIII.1802.1))	, gw1.VIII.1802.1
At3g29360	((At5g39320, At5g15490, At3g29360), (estExt_fgenesh4_pm.C_LG_XVII0156, eugene3.00041110))	estExt_fgenesh4_pm.C_LG_XVII0156, eugene3.00041110
At3g43190	((At5g20830, At3g43190), (estExt_fgenesh4_pg.C_280066))	estExt_fgenesh4_pg.C_280066
At3g44880	((At4g36220, At3g44880), (estExt_Genewise1_v1.C_280159))	estExt_Genewise1_v1.C_280159
At3g45040	((At3g45040), (gw1.IX.4952.1))	gw1.IX.4952.1
At3g45140	((At3g45140), (estExt_fgenesh4_pm.C_LG_XVII0021, fgenesh4_pg.C_LG_I000375))	estExt_fgenesh4_pm.C_LG_XVII0021, fgenesh4_pg.C_LG_I000375
At3g46100	((At3g46100), (gw1.28.724.1))	gw1.28.724.1
At3g46440	((At4g14820, At3g46440), (estExt_Genewise1_v1.C_LG_VIII2330))	estExt_Genewise1_v1.C_LG_VIII2330
At3g46660	((At3g46660), (eugene3.11070001))	eugene3.11070001
At3g46680	((At5g59590, At3g46690, At3g46680), (eugene3.00011560))	eugene3.00011560
At3g46690	((At5g59590, At3g46690, At3g46680), (eugene3.00011560))	eugene3.00011560
At3g46940	((At3g46940), (estExt_fgenesh4_pg.C_LG_II2596))	estExt_fgenesh4_pg.C_LG_II2596
At3g46970	((At3g46970), (estExt_fgenesh4_pg.C_LG_IX1158))	estExt_fgenesh4_pg.C_LG_IX1158
At3g47190	((At5g58660, At3g47190), (estExt_Genewise1_v1.C_LG_I2124))	estExt_Genewise1_v1.C_LG_I2124
At3g47340	((At3g47340), (estExt_Genewise1_v1.C_LG_IX3026))	estExt_Genewise1_v1.C_LG_IX3026
At3g47400	((At3g47400), (estExt_fgenesh4_pg.C_LG_XVIII0183))	estExt_fgenesh4_pg.C_LG_XVIII0183
At3g47450	((At3g47450), ())	
At3g48000	((At3g48000), (grail3.0005008802))	grail3.0005008802
At3g48080	((At3g48090, At3g48080), (estExt_fgenesh4_pg.C_LG_XII0735, fgenesh4_pm.C_LG_XV000173))	estExt_fgenesh4_pg.C_LG_XII0735, fgenesh4_pm.C_LG_XV000173
At3g48090	((At3g48090, At3g48080), (estExt_fgenesh4_pg.C_LG_XII0735, fgenesh4_pm.C_LG_XV000173))	estExt_fgenesh4_pg.C_LG_XII0735, fgenesh4_pm.C_LG_XV000173
At3g48680	((At3g48680), (estExt_fgenesh4_kg.C_LG_XV0016))	estExt_fgenesh4_kg.C_LG_XV0016
At3g48730	((At3g48730), (estExt_fgenesh4_pg.C_LG_I0874))	estExt_fgenesh4_pg.C_LG_I0874
At3g48780	((At5g23670, At3g48780), (estExt_fgenesh4_pm.C_LG_XII0379, estExt_Genewise1_v1.C_LG_XV2341))	estExt_fgenesh4_pm.C_LG_XII0379, estExt_Genewise1_v1.C_LG_XV2341
At3g48950	((At3g48950), (gw1.44.613.1))	gw1.44.613.1
At3g49050	((At3g49050), (fgenesh4_pm.C_LG_XII000429, gw1.XV.3413.1))	fgenesh4_pm.C_LG_XII000429, gw1.XV.3413.1
At3g49680	((At3g49680, At1g52510), (eugene3.00140246))	eugene3.00140246

At3g50660	((At3g50660), (fgenesh4_pm.C_LG_IV000367))	fgenesh4_pm.C_LG_IV000367
At3g50740	((At3g50740), (eugene3.00031105))	eugene3.00031105
At3g51240	((At3g51240), (estExt_fgenesh4_pm.C_570040))	estExt_fgenesh4_pm.C_570040
At3g51520	((At3g51520), (gw1.XI.734.1))	gw1.XI.734.1
At3g51820	((At3g51820, At3g14930), (gw1.XVIII.3460.1))	gw1.XVIII.3460.1
At3g51840	((At3g51840), (estExt_Genewise1_v1.C_LG_XVI3692, estExt_Genewise1_v1.C_LG_VI1706))	estExt_Genewise1_v1.C_LG_XVI3692, estExt_Genewise1_v1.C_LG_VI1706
At3g52180	((At3g52180), (grail3.0005006601))	grail3.0005006601
At3g52200	((At4g40050, At3g52200), (fgenesh4_pg.C_LG_VII000454, gw1.XVIII.1983.1))	fgenesh4_pg.C_LG_VII000454, gw1.XVIII.1983.1
At3g52430	((At3g52430), (gw1.VII.1589.1))	gw1.VII.1589.1
At3g52930	((At3g52930, At2g36460), (estExt_fgenesh4_pm.C_1210019, eugene3.00180798))	estExt_fgenesh4_pm.C_1210019, eugene3.00180798
At3g52940	((At3g52940, At3g06190), (estExt_Genewise1_v1.C_LG_IV1489, grail3.0001032301))	estExt_Genewise1_v1.C_LG_IV1489, grail3.0001032301
At3g52990	((At3g52990, At2g36580), (eugene3.00280289))	eugene3.00280289
At3g53130	((At3g53130), (eugene3.00280258))	eugene3.00280258
At3g53150	((At3g53150), (gw1.IX.3299.1, gw1.I.1811.1))	gw1.IX.3299.1, gw1.I.1811.1
At3g53180	((At3g53180), (eugene3.00280245))	eugene3.00280245
At3g53520	((At3g53520), (estExt_Genewise1_v1.C_LG_XVI2527))	estExt_Genewise1_v1.C_LG_XVI2527
At3g53580	((At3g53580), (estExt_Genewise1_v1.C_LG_VI1708, gw1.XVI.3802.1))	estExt_Genewise1_v1.C_LG_VI1708, gw1.XVI.3802.1
At3g53900	((At3g53900), (eugene3.00880092))	eugene3.00880092
At3g54050	((At3g54050), (estExt_Genewise1_v1.C_LG_XVI3399))	estExt_Genewise1_v1.C_LG_XVI3399
At3g54250	((At3g54250, At2g38700), (grail3.0106013901, grail3.0090014401))	grail3.0106013901, grail3.0090014401
At3g54470	((At3g54470), (estExt_Genewise1_v1.C_LG_I7094, grail3.0074007001))	estExt_Genewise1_v1.C_LG_I7094, grail3.0074007001
At3g55010	((At3g55010), (fgenesh4_pm.C_LG_XIV000303,))	fgenesh4_pm.C_LG_XIV000303,
At3g55120	((At4g09350, At3g55120), (grail3.0114003501))	grail3.0114003501
At3g55360	((At3g55360), (estExt_Genewise1_v1.C_LG_VIII1722))	estExt_Genewise1_v1.C_LG_VIII1722
At3g55410	((At5g65750, At3g55410), (estExt_fgenesh4_pg.C_LG_VIII0421, estExt_Genewise1_v1.C_LG_X2288))	estExt_fgenesh4_pg.C_LG_VIII0421, estExt_Genewise1_v1.C_LG_X2288
At3g55440	((At3g55440), (estExt_Genewise1_v1.C_LG_X2172, grail3.0049021504))	estExt_Genewise1_v1.C_LG_X2172, grail3.0049021504
At3g55610	((At3g55610, At2g39800), (estExt_fgenesh4_pm.C_LG_X0783))	estExt_fgenesh4_pm.C_LG_X0783
At3g55700	((At3g55710, At3g55700), (gw1.X.1932.1))	gw1.X.1932.1

At3g55710	((At3g55710, At3g55700), (gw1.X.1932.1))	gw1.X.1932.1
At3g55800	((At3g55800), (estExt_fgenesh4_pg.C_LG_VIII0539, eugene3.00101874))	estExt_fgenesh4_pg.C_LG_VIII0539, eugene3.00101874
At3g56940	((At3g56940), (fgenesh4_pg.C_LG_VI000236, estExt_Genewise1_v1.C_LG_XVI1018))	fgenesh4_pg.C_LG_VI000236, estExt_Genewise1_v1.C_LG_XVI1018
At3g57050	((At3g57050), ())	
At3g57220	((At3g57220), (estExt_Genewise1_v1.C_770059))	estExt_Genewise1_v1.C_770059
At3g57550	((At3g57550, At2g41880), (fgenesh4_pm.C_LG_XVI000165))	fgenesh4_pm.C_LG_XVI000165
At3g57610	((At3g57610), (estExt_fgenesh4_pg.C_LG_XVI0748))	estExt_fgenesh4_pg.C_LG_XVI0748
At3g57790	((At3g57790), (gw1.I.8594.1))	gw1.I.8594.1
At3g58140	((At3g58140), (eugene3.00570137))	eugene3.00570137
At3g58610	((At3g58610), (estExt_fgenesh4_pg.C_1240037))	estExt_fgenesh4_pg.C_1240037
At3g58750	((At3g58750), (estExt_Genewise1_v1.C_LG_XIV2957))	estExt_Genewise1_v1.C_LG_XIV2957
At3g58790	((At3g58790), (fgenesh4_pg.C_LG_XIV000829))	fgenesh4_pg.C_LG_XIV000829
At3g59380	((At3g59380), (eugene3.00070186))	eugene3.00070186
At3g59760	((At3g59760), (estExt_fgenesh4_pg.C_660249, grail3.0145000602))	estExt_fgenesh4_pg.C_660249, grail3.0145000602
At3g59850	((At3g59850), (fgenesh4_pm.C_scaffold_170000012))	fgenesh4_pm.C_scaffold_170000012
At3g59890	((At5g22000, At3g59890, At2g01110), (gw1.X.6706.1))	gw1.X.6706.1
At3g59970	((At3g59970, At2g44160), (estExt_fgenesh4_pg.C_1700003))	estExt_fgenesh4_pg.C_1700003
At3g60750	((At3g60750, At2g45290), (eugene3.00021349))	eugene3.00021349
At3g61130	((At3g61130), (gw1.XIV.923.1))	gw1.XIV.923.1
At3g61200	((At3g61200), (grail3.0035013301, eugene3.00021446))	grail3.0035013301, eugene3.00021446
At3g61400	((At3g61400), (estExt_fgenesh4_pg.C_LG_XV0636))	estExt_fgenesh4_pg.C_LG_XV0636
At3g61510	((At3g61510), (estExt_fgenesh4_pm.C_LG_II0741))	estExt_fgenesh4_pm.C_LG_II0741
At3g61680	((At3g61680), (eugene3.45740001))	eugene3.45740001
At3g62110	((At3g62110), (fgenesh4_pg.C_LG_II001910))	fgenesh4_pg.C_LG_II001910
At3g62170	((At3g62170, At2g47040, At2g47030), (fgenesh4_pg.C_LG_I001266, eugene3.00030089, eugene3.00030085, eugene3.00011207))	fgenesh4_pg.C_LG_I001266, eugene3.00030089, eugene3.00030085, [illegible]
At3g62660	((At3g62660), (grail3.0061016301, eugene3.00021805))	grail3.0061016301, eugene3.00021805
At3g62830	((At3g62830, At2g47650), (gw1.I.4975.1))	gw1.I.4975.1
At3g63110	((At3g63110), (fgenesh4_pg.C_LG_XVIII000329, fgenesh4_pg.C_LG_VI001718))	fgenesh4_pg.C_LG_XVIII000329, fgenesh4_pg.C_LG_VI001718

At3g63250	((At3g63250), (estExt_Genewise1_v1.C_LG_XIV0065))	estExt_Genewise1_v1.C_LG_XIV0065
At3g63410	((At3g63410), (estExt_fgenesh4_pm.C_LG_VIII0681, grail3.0003038902, eugene3.00051228))	estExt_fgenesh4_pm.C_LG_VIII0681, grail3.0003038902, eugene3.00051228
At4g00110	((At4g00110), (grail3.0033029301))	grail3.0033029301
At4g00400	((At4g00400), (estExt_fgenesh4_pm.C_LG_XIV0124))	estExt_fgenesh4_pm.C_LG_XIV0124
At4g00490	((At4g00490), (estExt_fgenesh4_pm.C_LG_VIII0731))	estExt_fgenesh4_pm.C_LG_VIII0731
At4g00500	((At4g00500), (estExt_fgenesh4_pm.C_LG_XIV0119, estExt_fgenesh4_pg.C_LG_II1450))	estExt_fgenesh4_pm.C_LG_XIV0119, estExt_fgenesh4_pg.C_LG_II1450
At4g00550	((At4g00550), (estExt_fgenesh4_pm.C_LG_XIV0099))	estExt_fgenesh4_pm.C_LG_XIV0099
At4g00590	((At4g00590, At5g61540), (estExt_fgenesh4_pm.C_LG_XIV0105))	estExt_fgenesh4_pm.C_LG_XIV0105
At4g01690	((At4g01690, At1g27980), (fgenesh4_pg.C_LG_XIV000523, fgenesh4_pg.C_LG_II001916))	fgenesh4_pg.C_LG_XIV000523, fgenesh4_pg.C_LG_II001916
At4g01850	((At4g01850, At1g02500), (estExt_fgenesh4_pm.C_LG_XIV0257, grail3.0050014702))	estExt_fgenesh4_pm.C_LG_XIV0257, grail3.0050014702
At4g01890	((At3g07970, At4g01890), (fgenesh4_pg.C_LG_II001877))	fgenesh4_pg.C_LG_II001877
At4g01970	((At4g01970), (eugene3.00140639))	eugene3.00140639
At4g02120	((At4g02120), (estExt_Genewise1_v1.C_LG_XIV2383))	estExt_Genewise1_v1.C_LG_XIV2383
At4g02280	((At4g02280), (estExt_fgenesh4_pm.C_LG_II0867))	estExt_fgenesh4_pm.C_LG_II0867
At4g02300	((At4g02300), (estExt_fgenesh4_pm.C_LG_X0184, grail3.0009029402))	estExt_fgenesh4_pm.C_LG_X0184, grail3.0009029402
At4g02320	((At4g02320), (eugene3.00280097))	eugene3.00280097
At4g02330	((At4g02330), (eugene3.00140717))	eugene3.00140717
At4g02580	((At4g02580), (estExt_fgenesh4_pm.C_LG_V0527, estExt_Genewise1_v1.C_LG_II0234))	estExt_fgenesh4_pm.C_LG_V0527, estExt_Genewise1_v1.C_LG_II0234
At4g02780	((At4g02780), (fgenesh4_pg.C_LG_V001411, fgenesh4_pg.C_LG_V001263, fgenesh4_pg.C_LG_II000338))	fgenesh4_pg.C_LG_V001411, fgenesh4_pg.C_LG_V001263, [illegible]
At4g05160	((At4g05160), (estExt_fgenesh4_pm.C_1230033, estExt_fgenesh4_pm.C_LG_IV0315))	estExt_fgenesh4_pm.C_1230033, estExt_fgenesh4_pm.C_LG_IV0315
At4g07960	((At4g07960), (estExt_fgenesh4_pg.C_LG_V0618, estExt_fgenesh4_pg.C_LG_II1042))	estExt_fgenesh4_pg.C_LG_V0618, estExt_fgenesh4_pg.C_LG_II1042
At4g08790	((At4g08790), (gw1.107.231.1, gw1.XI.7.1))	gw1.107.231.1, gw1.XI.7.1
At4g08900	((At4g08900), (eugene3.00021348))	eugene3.00021348
At4g09020	((At4g09020), (gw1.XIX.1316.1))	gw1.XIX.1316.1
At4g09510	((At4g09510), (gw1.66.49.1, gw1.IX.1371.1))	gw1.66.49.1, gw1.IX.1371.1
At4g09760	((At4g09760), (fgenesh4_pg.C_LG_II000596))	fgenesh4_pg.C_LG_II000596
At4g10120	((At4g10120), (gw1.XIII.3112.1))	gw1.XIII.3112.1
At4g10955	((At5g45760, At4g10955), (estExt_Genewise1_v1.C_LG_XIV0433))	estExt_Genewise1_v1.C_LG_XIV0433

At4g12130	((At4g12130), (fgenesh4_pg.C_scaffold_64000067))	fgenesh4_pg.C_scaffold_64000067
At4g12430	((At4g22590, At4g12430), (estExt_fgenesh4_pm.C_1270030, gw1.III.870.1))	estExt_fgenesh4_pm.C_1270030, gw1.III.870.1
At4g12440	((At4g12440), (estExt_fgenesh4_pg.C_1270057))	estExt_fgenesh4_pg.C_1270057
At4g13550	((At4g13550), (fgenesh4_pg.C_scaffold_29000017, gw1.V.1092.1))	fgenesh4_pg.C_scaffold_29000017, gw1.V.1092.1
At4g13760	((At4g13760, At2g40310, At2g26620, At2g15470, At2g15460, At2g15450, At1g43100, At1g43090, At1g43080), (eugene3.00091271))	eugene3.00091271
At4g13780	((At4g13780), (gw1.88.24.1))	gw1.88.24.1
At4g13930	((At4g13930), (estExt_fgenesh4_pm.C_880008, estExt_fgenesh4_pm.C_LG_I0969))	estExt_fgenesh4_pm.C_880008, estExt_fgenesh4_pm.C_LG_I0969
At4g13940	((At4g13940, At3g23810), (estExt_Genewise1_v1.C_LG_I3343, eugene3.00880022))	estExt_Genewise1_v1.C_LG_I3343, eugene3.00880022
At4g14210	((At4g14210), (eugene3.00002400))	eugene3.00002400
At4g14710	((At4g14716, At4g14710, At2g26400), (estExt_Genewise1_v1.C_LG_VIII0915))	estExt_Genewise1_v1.C_LG_VIII0915
At4g14716	((At4g14716, At4g14710, At2g26400), (estExt_Genewise1_v1.C_LG_VIII0915))	estExt_Genewise1_v1.C_LG_VIII0915
At4g14880	((At4g14880), (estExt_fgenesh4_pg.C_700106, fgenesh4_pg.C_LG_VIII001376, grail3.1757000202, eugene3.00130338))	estExt_fgenesh4_pg.C_700106, fgenesh4_pg.C_LG_VIII001376, [illegible]
At4g14940	((At4g14940), (estExt_Genewise1_v1.C_1270098))	estExt_Genewise1_v1.C_1270098
At4g15210	((At4g15210), (gw1.I.8484.1))	gw1.I.8484.1
At4g15290	((At4g15320, At4g15290, At2g32620, At2g32610, At2g32540, At2g32530), (estExt_fgenesh4_pg.C_LG_II2039, gw1.XIV.3402.1))	estExt_fgenesh4_pg.C_LG_II2039, gw1.XIV.3402.1
At4g15320	((At4g15320, At4g15290, At2g32620, At2g32610, At2g32540, At2g32530), (estExt_fgenesh4_pg.C_LG_II2039, gw1.XIV.3402.1))	estExt_fgenesh4_pg.C_LG_II2039, gw1.XIV.3402.1
At4g15490	((At4g15490), (gw1.IX.2384.1))	gw1.IX.2384.1
At4g15560	((At4g15560), (fgenesh4_pg.C_LG_VIII001771))	fgenesh4_pg.C_LG_VIII001771
At4g16070	((At4g16070), (fgenesh4_pg.C_scaffold_77000076, estExt_Genewise1_v1.C_LG_X3716))	fgenesh4_pg.C_scaffold_77000076, estExt_Genewise1_v1.C_LG_X3716
At4g16130	((At4g16130), (fgenesh4_pm.C_scaffold_164000028, gw1.XIX.729.1))	fgenesh4_pm.C_scaffold_164000028, gw1.XIX.729.1
At4g16155	((At4g16155, At3g16950), (gw1.X.574.1))	gw1.X.574.1
At4g16450	((At4g16450), (estExt_fgenesh4_pg.C_LG_XVI0037, eugene3.00060155))	estExt_fgenesh4_pg.C_LG_XVI0037, eugene3.00060155
At4g16700	((At4g16700), (estExt_Genewise1_v1.C_LG_V0786, gw1.XVIII.2449.1))	estExt_Genewise1_v1.C_LG_V0786, gw1.XVIII.2449.1
At4g16740	((At4g16740), (eugene3.00170127))	eugene3.00170127
At4g16760	((At4g16760, At2g35690), (estExt_fgenesh4_pm.C_290034))	estExt_fgenesh4_pm.C_290034
At4g16800	((At4g16800), (estExt_fgenesh4_pg.C_LG_III0605))	estExt_fgenesh4_pg.C_LG_III0605
At4g16820	((At4g16820), (eugene3.00030555))	eugene3.00030555
At4g17090	((At4g17090), (eugene3.01180078))	eugene3.01180078

At4g17190	((At4g17190), (eugene3.00060040))	eugene3.00060040
At4g17260	((At4g17260), (fgenesh4_pg.C_LG_IV000431))	fgenesh4_pg.C_LG_IV000431
At4g17300	((At4g17300), (eugene3.00640117))	eugene3.00640117
At4g17360	((At1g66520, At4g17360, At1g31220), (eugene3.00002342))	eugene3.00002342
At4g17770	((At4g17770), (fgenesh4_pg.C_LG_III000738))	fgenesh4_pg.C_LG_III000738
At4g18440	((At4g18440), (estExt_fgenesh4_pg.C_LG_IV0231))	estExt_fgenesh4_pg.C_LG_IV0231
At4g18480	((At4g18480, At5g45930), (gw1.IV.2852.1))	gw1.IV.2852.1
At4g18550	((At5g01500, At2g42690, At4g18550), (fgenesh4_pm.C_LG_III000281))	fgenesh4_pm.C_LG_III000281
At4g18550	((At5g01500, At2g42690, At4g18550), (fgenesh4_pm.C_LG_III000281))	fgenesh4_pm.C_LG_III000281
At4g18780	((At4g18780), (gw1.XI.3218.1))	gw1.XI.3218.1
At4g19010	((At4g19010, At4g11690), (eugene3.00002248))	eugene3.00002248
At4g19710	((At4g19710), (gw1.XIV.3082.1))	gw1.XIV.3082.1
At4g20050	((At4g20050), (grail3.0029003101, eugene3.26080001))	grail3.0029003101, eugene3.26080001
At4g20150	((At4g20150), (estExt_fgenesh4_pg.C_LG_I0649, grail3.0047001901))	estExt_fgenesh4_pg.C_LG_I0649, grail3.0047001901
At4g20930	((At4g20930), (eugene3.00013051))	eugene3.00013051
At4g20960	((At4g20960), (grail3.0035016801))	grail3.0035016801
At4g21200	((At4g21200), (gw1.XIII.2181.1))	gw1.XIII.2181.1
At4g22340	((At4g26770, At4g22340, At3g60620, At2g45150, At1g62430), (estExt_fgenesh4_pm.C_1660030))	estExt_fgenesh4_pm.C_1660030
At4g22590	((At4g22590, At4g12430), (estExt_fgenesh4_pm.C_1270030, gw1.III.870.1))	estExt_fgenesh4_pm.C_1270030, gw1.III.870.1
At4g22880	((At4g22880), (fgenesh4_pg.C_LG_VIII000593))	fgenesh4_pg.C_LG_VIII000593
At4g22930	((At4g22930), (gw1.XVIII.1745.1))	gw1.XVIII.1745.1
At4g23100	((At4g23100), (estExt_fgenesh4_pm.C_LG_III0405, estExt_Genewise1_v1.C_LG_I8937))	estExt_fgenesh4_pm.C_LG_III0405, estExt_Genewise1_v1.C_LG_I8937
At4g23660	((At4g23660), (estExt_fgenesh4_pg.C_LG_X0871, eugene3.00081367))	estExt_fgenesh4_pg.C_LG_X0871, eugene3.00081367
At4g23820	((At4g23820), (fgenesh4_pm.C_LG_VI000363, eugene3.00161173, eugene3.00161171))	fgenesh4_pm.C_LG_VI000363, eugene3.00161173, eugene3.00161171
At4g23900	((At4g23900), (grail3.0008036902))	grail3.0008036902
At4g23920	((At4g23920), (estExt_fgenesh4_pg.C_LG_I0797, grail3.0018042501))	estExt_fgenesh4_pg.C_LG_I0797, grail3.0018042501
At4g23990	((At4g23990), (eugene3.00031146))	eugene3.00031146
At4g24040	((At4g24040), (estExt_fgenesh4_pg.C_LG_VI1454, eugene3.00180242))	estExt_fgenesh4_pg.C_LG_VI1454, eugene3.00180242

At4g24620	((At4g24620), (eugene3.00002669,))	eugene3.00002669,
At4g24830	((At4g24830), (gw1.XVIII.665.1))	gw1.XVIII.665.1
At4g25000	((At5g47860, At4g25000, At1g56710), (grail3.0047003202, gw1.XIII.514.1))	grail3.0047003202, gw1.XIII.514.1
At4g25080	((At4g25080), (grail3.0015033802, gw1.XV.2421.1))	grail3.0015033802, gw1.XV.2421.1
At4g25420	((At4g25420), (fgenesh4_pm.C_LG_XIV000079))	fgenesh4_pm.C_LG_XIV000079
At4g25570	((At4g25570), (estExt_fgenesh4_pg.C_LG_XV1133, grail3.0079004101))	estExt_fgenesh4_pg.C_LG_XV1133, grail3.0079004101
At4g25700	((At4g25700), (gw1.IV.3981.1))	gw1.IV.3981.1
At4g25970	((At5g57190, At4g25970), (estExt_fgenesh4_pg.C_LG_III0575, fgenesh4_pg.C_scaffold_6993000001, grail3.0037005801, [illegible]	estExt_fgenesh4_pg.C_LG_III0575, fgenesh4_pg.C_scaffold_6993000001, [illegible]
At4g26200	((At4g26200), ())	
At4g26270	((At5g56630, At4g26270), (estExt_Genewise1_v1.C_1450141))	estExt_Genewise1_v1.C_1450141
At4g26300	((At1g66530, At4g26300), (estExt_Genewise1_v1.C_LG_II3255))	estExt_Genewise1_v1.C_LG_II3255
At4g26530	((At4g26530), (estExt_Genewise1_v1.C_1400173, estExt_Genewise1_v1.C_LG_I6144))	estExt_Genewise1_v1.C_1400173, estExt_Genewise1_v1.C_LG_I6144
At4g27070	((At5g54810, At4g27070, At4g13700, At4g11920), (gw1.I.9225.1))	gw1.I.9225.1
At4g29010	((At4g29010), (estExt_fgenesh4_pg.C_LG_XVIII0682))	estExt_fgenesh4_pg.C_LG_XVIII0682
At4g29130	((At4g29130, At2g19860), (estExt_fgenesh4_pg.C_LG_XVIII0751))	estExt_fgenesh4_pg.C_LG_XVIII0751
At4g29210	((At4g39650, At4g29210), (fgenesh4_pg.C_scaffold_18583000001))	fgenesh4_pg.C_scaffold_18583000001
At4g30000	((At4g30000), (eugene3.00002262))	eugene3.00002262
At4g30440	((At4g30440), (eugene3.00180906, eugene3.00061339))	eugene3.00180906, eugene3.00061339
At4g30470	((At4g30470, At2g23910), (estExt_fgenesh4_pm.C_LG_VI0550, estExt_fgenesh4_pg.C_LG_XVIII0872))	estExt_fgenesh4_pm.C_LG_VI0550, estExt_fgenesh4_pg.C_LG_XVIII0872
At4g30950	((At4g30950), (estExt_fgenesh4_pm.C_LG_VI0352, estExt_fgenesh4_pg.C_LG_XVI1077))	estExt_fgenesh4_pm.C_LG_VI0352, estExt_fgenesh4_pg.C_LG_XVI1077
At4g31590	((At4g31590, At2g24630), (fgenesh4_pg.C_LG_VI001955, eugene3.00180589))	fgenesh4_pg.C_LG_VI001955, eugene3.00180589
At4g31780	((At4g31780), (estExt_fgenesh4_pm.C_LG_XVIII0180, eugene3.00061974))	estExt_fgenesh4_pm.C_LG_XVIII0180, eugene3.00061974
At4g32180	((At4g32180), (estExt_fgenesh4_pg.C_LG_XV0519, eugene3.00120751))	estExt_fgenesh4_pg.C_LG_XV0519, eugene3.00120751
At4g32320	((At4g32320), (estExt_fgenesh4_pg.C_LG_VI1797))	estExt_fgenesh4_pg.C_LG_VI1797
At4g32410	((At4g32410), (estExt_fgenesh4_pm.C_LG_XVIII0125, estExt_fgenesh4_pg.C_13980001, fgenesh4_pg.C_LG_VI001789))	estExt_fgenesh4_pm.C_LG_XVIII0125, estExt_fgenesh4_pg.C_13980001, [illegible]
At4g32520	((At4g32520), (estExt_fgenesh4_pg.C_LG_VI1584))	estExt_fgenesh4_pg.C_LG_VI1584
At4g33010	((At4g33010, At2g26080), (estExt_fgenesh4_pm.C_LG_VI0678))	estExt_fgenesh4_pm.C_LG_VI0678
At4g33030	((At4g33030), (estExt_fgenesh4_pg.C_LG_VI1540))	estExt_fgenesh4_pg.C_LG_VI1540

At4g33150	((At4g33150), (estExt_Genewise1_v1.C_280333))	estExt_Genewise1_v1.C_280333
At4g33230	((At4g33230, At2g26450), (eugene3.00180192))	eugene3.00180192
At4g33440	((At3g16850, At4g33440), (gw1.X.3724.1))	gw1.X.3724.1
At4g33670	((At4g33670), (estExt_fgenesh4_pm.C_LG_IX0390))	estExt_fgenesh4_pm.C_LG_IX0390
At4g33680	((At4g33680), (eugene3.00070099))	eugene3.00070099
At4g34200	((At4g34200), (grail3.0042006001))	grail3.0042006001
At4g34350	((At4g34350), (gw1.X.1884.1))	gw1.X.1884.1
At4g34640	((At4g34640), (estExt_fgenesh4_pg.C_LG_IV1345))	estExt_fgenesh4_pg.C_LG_IV1345
At4g34710	((At4g34710), (estExt_Genewise1_v1.C_LG_IV0804))	estExt_Genewise1_v1.C_LG_IV0804
At4g34740	((At4g34740, At3g24090), (gw1.XIX.1506.1))	gw1.XIX.1506.1
At4g34860	((At4g34860, At1g16540), (fgenesh4_pg.C_LG_IV001415, estExt_Genewise1_v1.C_LG_VII2665))	fgenesh4_pg.C_LG_IV001415, estExt_Genewise1_v1.C_LG_VII2665
At4g34880	((At4g34880), (eugene3.00011242))	eugene3.00011242
At4g35000	((At4g35000), (estExt_fgenesh4_pm.C_LG_IV0530, eugene3.00090344))	estExt_fgenesh4_pm.C_LG_IV0530, eugene3.00090344
At4g35090	((At4g35090), (estExt_fgenesh4_pm.C_LG_V0695, eugene3.00020082))	estExt_fgenesh4_pm.C_LG_V0695, eugene3.00020082
At4g35360	((At4g35360), (estExt_fgenesh4_pm.C_660095, estExt_Genewise1_v1.C_LG_IX0139))	estExt_fgenesh4_pm.C_660095, estExt_Genewise1_v1.C_LG_IX0139
At4g35460	((At5g51550, At4g35460, At2g26600, At2g17420), (estExt_fgenesh4_pg.C_LG_XVIII1099, eugene3.00140843))	estExt_fgenesh4_pg.C_LG_XVIII1099, eugene3.00140843
At4g35630	((At4g35630), (gw1.V.3607.1))	gw1.V.3607.1
At4g35640	((At4g35640), (fgenesh4_pg.C_LG_III001579, estExt_Genewise1_v1.C_4870010, eugene3.02080002, eugene3.00031548))	fgenesh4_pg.C_LG_III001579, estExt_Genewise1_v1.C_4870010, [illegible]
At4g35790	((At4g35790), (fgenesh4_pm.C_scaffold_181000001, eugene3.00570012))	fgenesh4_pm.C_scaffold_181000001, eugene3.00570012
At4g35830	((At4g35830), (eugene3.00570034, eugene3.01810009))	eugene3.00570034, eugene3.01810009
At4g36220	((At4g36220, At3g44880), (estExt_Genewise1_v1.C_280159))	estExt_Genewise1_v1.C_280159
At4g36250	((At4g36250, At2g05940), (gw1.I.8414.1))	gw1.I.8414.1
At4g36480	((At4g36480), (fgenesh4_pg.C_LG_XII000006))	fgenesh4_pg.C_LG_XII000006
At4g36810	((At4g36810), (estExt_Genewise1_v1.C_570227))	estExt_Genewise1_v1.C_570227
At4g37000	((At4g37000), (estExt_fgenesh4_pm.C_LG_III0624, estExt_fgenesh4_pg.C_2080026))	estExt_fgenesh4_pm.C_LG_III0624, estExt_fgenesh4_pg.C_2080026
At4g37070	((At4g37070, At4g37060), (eugene3.00070928))	eugene3.00070928
At4g37770	((At4g37770), (eugene3.00140049))	eugene3.00140049
At4g37840	((At4g37840), (fgenesh4_pg.C_LG_VII001238))	fgenesh4_pg.C_LG_VII001238

At4g37870	((At4g37870), (estExt_fgenesh4_pg.C_LG_II0983, estExt_Genewise1_v1.C_LG_VII3984))	estExt_fgenesh4_pg.C_LG_II0983, estExt_Genewise1_v1.C_LG_VII3984
At4g37930	((At4g37930), (estExt_fgenesh4_pm.C_LG_X0942, gw1.VIII.2633.1))	estExt_fgenesh4_pm.C_LG_X0942, gw1.VIII.2633.1
At4g38190	((At4g38190), (fgenesh4_pg.C_LG_IX000009, eugene3.00660228))	fgenesh4_pg.C_LG_IX000009, eugene3.00660228
At4g38270	((At4g38270, At1g02020), (gw1.66.585.1))	gw1.66.585.1
At4g38460	((At4g38460), (grail3.0001025501))	grail3.0001025501
At4g38690	((At4g38690), (estExt_Genewise1_v1.C_LG_IV1055))	estExt_Genewise1_v1.C_LG_IV1055
At4g38800	((At4g38800, At4g34840), (estExt_fgenesh4_pg.C_LG_IV1398, estExt_fgenesh4_kg.C_LG_IX0009))	estExt_fgenesh4_pg.C_LG_IV1398, estExt_fgenesh4_kg.C_LG_IX0009
At4g38970	((At4g38970, At2g21330), (estExt_fgenesh4_pm.C_LG_IX0211, fgenesh4_pm.C_LG_VII000464, estExt_Genewise1_v1.C_LG_IV0774))	estExt_fgenesh4_pm.C_LG_IX0211, fgenesh4_pm.C_LG_VII000464, [illegible]
At4g39280	((At4g39280), (estExt_fgenesh4_pg.C_LG_IX0484, gw1.IV.516.1))	estExt_fgenesh4_pg.C_LG_IX0484, gw1.IV.516.1
At4g39350	((At4g39350), (gw1.V.3193.1))	gw1.V.3193.1
At4g39640	((At4g39640), (eugene3.00160552))	eugene3.00160552
At4g39830	((At4g39830), (eugene3.00101633))	eugene3.00101633
At5g03490	((At5g55730, At5g03490, At3g26950, At1g51210, At1g24030), (gw1.X.192.1))	gw1.X.192.1
At5g03650	((At5g03650), (fgenesh4_pg.C_LG_I002811))	fgenesh4_pg.C_LG_I002811
At5g03770	((At5g03770), (eugene3.00160899, gw1.197.19.1))	eugene3.00160899, gw1.197.19.1
At5g03820	((At5g03820, At5g03810), (fgenesh4_pg.C_scaffold_28000260))	fgenesh4_pg.C_scaffold_28000260
At5g04040	((At5g04040), (eugene3.00060391, gw1.XVI.1394.1))	eugene3.00060391, gw1.XVI.1394.1
At5g04140	((At5g04140), (estExt_fgenesh4_pg.C_LG_XVI0276, estExt_fgenesh4_pg.C_LG_VI0304))	estExt_fgenesh4_pg.C_LG_XVI0276, estExt_fgenesh4_pg.C_LG_VI0304
At5g04360	((At5g04360), (estExt_fgenesh4_pm.C_LG_X0918))	estExt_fgenesh4_pm.C_LG_X0918
At5g04490	((At5g04490), (gw1.V.3137.1))	gw1.V.3137.1
At5g04590	((At5g04590), (eugene3.00070600))	eugene3.00070600
At5g05170	((At5g05170), (estExt_fgenesh4_pg.C_LG_IX0979, estExt_Genewise1_v1.C_LG_I1792))	estExt_fgenesh4_pg.C_LG_IX0979, estExt_Genewise1_v1.C_LG_I1792
At5g05270	((At5g05270), (estExt_fgenesh4_pm.C_LG_XIX0169))	estExt_fgenesh4_pm.C_LG_XIX0169
At5g05590	((At5g05590, At1g29410, At1g07780), (estExt_Genewise1_v1.C_LG_XII0225, gw1.XVII.219.1))	estExt_Genewise1_v1.C_LG_XII0225, gw1.XVII.219.1
At5g05980	((At5g05980), (gw1.VIII.881.1))	gw1.VIII.881.1
At5g07360	((At5g07360), (gw1.I.9626.1))	gw1.I.9626.1
At5g07410	((At5g07430, At5g07420, At5g07410, At1g69940), (gw1.142.161.1, gw1.XVII.471.1))	gw1.142.161.1, gw1.XVII.471.1
At5g07420	((At5g07430, At5g07420, At5g07410, At1g69940), (gw1.142.161.1, gw1.XVII.471.1))	gw1.142.161.1, gw1.XVII.471.1

At5g07430	((At5g07430, At5g07420, At5g07410, At1g69940), (gw1.142.161.1, gw1.XVII.471.1))	gw1.142.161.1, gw1.XVII.471.1
At5g07440	((At5g07440), (eugene3.00150904))	eugene3.00150904
At5g07990	((At5g07990), (estExt_fgenesh4_pg.C_LG_XIII0337))	estExt_fgenesh4_pg.C_LG_XIII0337
At5g08100	((At5g08100), (fgenesh4_pm.C_LG_VII000124))	fgenesh4_pm.C_LG_VII000124
At5g08170	((At5g08170), (estExt_fgenesh4_pm.C_820017))	estExt_fgenesh4_pm.C_820017
At5g08280	((At5g08280), (gw1.XIV.2636.1, gw1.XIV.277.1))	gw1.XIV.2636.1, gw1.XIV.277.1
At5g08300	((At5g08300), (gw1.40.461.1))	gw1.40.461.1
At5g08370	((At5g08370), (fgenesh4_pg.C_scaffold_164000005))	fgenesh4_pg.C_scaffold_164000005
At5g08380	((At5g08380), (estExt_fgenesh4_pg.C_LG_III1156))	estExt_fgenesh4_pg.C_LG_III1156
At5g08530	((At5g08530), (estExt_fgenesh4_pg.C_LG_X0463))	estExt_fgenesh4_pg.C_LG_X0463
At5g08570	((At5g63680, At5g08570), (grail3.0106001101, eugene3.00080009))	grail3.0106001101, eugene3.00080009
At5g09420	((At5g09420), (fgenesh4_pg.C_scaffold_7289000001))	fgenesh4_pg.C_scaffold_7289000001
At5g09660	((At5g09660), (grail3.0001073802))	grail3.0001073802
At5g09760	((At5g09760), (fgenesh4_pg.C_LG_V000014, estExt_Genewise1_v1.C_LG_VII1401))	fgenesh4_pg.C_LG_V000014, estExt_Genewise1_v1.C_LG_VII1401
At5g10100	((At5g10100), (grail3.0011016001, grail3.0026014501))	grail3.0011016001, grail3.0026014501
At5g10240	((At5g65010, At5g10240), (gw1.V.2829.1))	gw1.V.2829.1
At5g10330	((At5g10330, At1g71920), (gw1.XVI.1990.1))	gw1.XVI.1990.1
At5g10870	((At5g10870), (fgenesh4_pg.C_LG_XVIII000491, grail3.0026036101))	fgenesh4_pg.C_LG_XVIII000491, grail3.0026036101
At5g10920	((At5g10920), (gw1.I.3271.1))	gw1.I.3271.1
At5g11160	((At5g11160), (estExt_fgenesh4_pg.C_LG_VI1808, grail3.0007027201))	estExt_fgenesh4_pg.C_LG_VI1808, grail3.0007027201
At5g11380	((At5g11380), (estExt_Genewise1_v1.C_LG_XVIII1471))	estExt_Genewise1_v1.C_LG_XVIII1471
At5g11520	((At5g11520), (estExt_fgenesh4_pg.C_LG_VI1672))	estExt_fgenesh4_pg.C_LG_VI1672
At5g11770	((At5g11770), (eugene3.00061644))	eugene3.00061644
At5g11880	((At5g11880), (fgenesh4_pg.C_LG_II000144, gw1.V.1951.1))	fgenesh4_pg.C_LG_II000144, gw1.V.1951.1
At5g12200	((At5g12200), (estExt_Genewise1_v1.C_LG_IX3131))	estExt_Genewise1_v1.C_LG_IX3131
At5g12890	((At5g12890), (fgenesh4_pm.C_LG_I000127))	fgenesh4_pm.C_LG_I000127
At5g13110	((At5g13110), (eugene3.00040482))	eugene3.00040482
At5g13420	((At5g13420), (gw1.163.36.1))	gw1.163.36.1

At5g13520	((At5g13520), (estExt_fgenesh4_pm.C_LG_VIII0150))	estExt_fgenesh4_pm.C_LG_VIII0150
At5g13630	((At5g13630), (estExt_fgenesh4_pg.C_LG_VI0422))	estExt_fgenesh4_pg.C_LG_VI0422
At5g13640	((At5g13640), (fgenesh4_pg.C_LG_I001065))	fgenesh4_pg.C_LG_I001065
At5g13700	((At5g13700, At1g18010, At1g18000), (eugene3.00010865))	eugene3.00010865
At5g13710	((At5g13710), (gw1.IX.3368.1))	gw1.IX.3368.1
At5g13930	((At5g13930), (eugene3.00110848))	eugene3.00110848
At5g14220	((At5g14220), (fgenesh4_pm.C_LG_I000925))	fgenesh4_pm.C_LG_I000925
At5g14590	((At5g14590), (fgenesh4_pm.C_LG_I000856))	fgenesh4_pm.C_LG_I000856
At5g14700	((At5g14700), (grail3.0062002901))	grail3.0062002901
At5g14760	((At5g14760), (gw1.18740.2.1))	gw1.18740.2.1
At5g14800	((At5g14800), (fgenesh4_pg.C_scaffold_133000014, eugene3.00130177))	fgenesh4_pg.C_scaffold_133000014, eugene3.00130177
At5g14850	((At5g14850), (eugene3.07690001, eugene3.00061354))	eugene3.07690001, eugene3.00061354
At5g14930	((At5g14930), (eugene3.00190020))	eugene3.00190020
At5g15470	((At5g15470, At3g01040), (eugene3.00141554, eugene3.00140791))	eugene3.00141554, eugene3.00140791
At5g15490	((At5g39320, At5g15490, At3g29360), (estExt_fgenesh4_pm.C_LG_XVII0156, eugene3.00041110))	estExt_fgenesh4_pm.C_LG_XVII0156, eugene3.00041110
At5g15770	((At5g15770), (estExt_Genewise1_v1.C_LG_X3413, estExt_Genewise1_v1.C_LG_VIII0441))	estExt_Genewise1_v1.C_LG_X3413, estExt_Genewise1_v1.C_LG_VIII0441
At5g15950	((At5g15950, At3g25570, At3g02470), (gw1.X.4229.1))	gw1.X.4229.1
At5g16010	((At5g16010), (estExt_fgenesh4_pg.C_LG_X2215))	estExt_fgenesh4_pg.C_LG_X2215
At5g16240	((At5g16240), (eugene3.01270057))	eugene3.01270057
At5g16390	((At5g16390), (grail3.0095003402))	grail3.0095003402
At5g16910	((At5g16910, At3g03050), (estExt_fgenesh4_pg.C_870007, eugene3.00190332))	estExt_fgenesh4_pg.C_870007, eugene3.00190332
At5g17040	((At5g17040), (gw1.X.5125.1))	gw1.X.5125.1
At5g17310	((At5g17310), (grail3.0038019301, eugene3.40280001))	grail3.0038019301, eugene3.40280001
At5g17330	((At5g17330), (grail3.0038018902))	grail3.0038018902
At5g17380	((At5g17380), (estExt_fgenesh4_pm.C_LG_IV0225))	estExt_fgenesh4_pm.C_LG_IV0225
At5g17420	((At5g17420), (estExt_Genewise1_v1.C_LG_VI2188))	estExt_Genewise1_v1.C_LG_VI2188
At5g17920	((At5g17920, At3g03780), (estExt_fgenesh4_pg.C_LG_XIII0289, estExt_Genewise1_v1.C_LG_XIX1125))	estExt_fgenesh4_pg.C_LG_XIII0289, estExt_Genewise1_v1.C_LG_XIX1125
At5g17990	((At5g17990), (grail3.0008009901))	grail3.0008009901

At5g18070	((At5g18070), (eugene3.00290147))	eugene3.00290147
At5g18170	((At5g18170, At3g03910), (estExt_fgenesh4_pg.C_LG_XIX0409, eugene3.00130573))	estExt_fgenesh4_pg.C_LG_XIX0409, eugene3.00130573
At5g18200	((At5g18200), (estExt_fgenesh4_pg.C_LG_XIX0404, eugene3.00061661))	estExt_fgenesh4_pg.C_LG_XIX0404, eugene3.00061661
At5g18630	((At5g18630), (fgenesh4_kg.C_LG_X000010))	fgenesh4_kg.C_LG_X000010
At5g18640	((At5g18640), (fgenesh4_pg.C_LG_V000522))	fgenesh4_pg.C_LG_V000522
At5g18660	((At5g18660), (estExt_fgenesh4_pg.C_LG_VIII1844))	estExt_fgenesh4_pg.C_LG_VIII1844
At5g18800	((At3g06310, At5g18800), (estExt_fgenesh4_pm.C_LG_X0097, estExt_Genewise1_v1.C_LG_VIII2811))	estExt_fgenesh4_pm.C_LG_X0097, estExt_Genewise1_v1.C_LG_VIII2811
At5g19040	((At5g19040), (gw1.162.1.1))	gw1.162.1.1
At5g19220	((At5g19220), (estExt_Genewise1_v1.C_LG_VIII1252))	estExt_Genewise1_v1.C_LG_VIII1252
At5g19440	((At5g19440), (gw1.IX.3396.1))	gw1.IX.3396.1
At5g19550	((At5g19550, At1g62800), (estExt_fgenesh4_pm.C_LG_XVIII0241))	estExt_fgenesh4_pm.C_LG_XVIII0241
At5g19730	((At5g19730, At5g55590), (estExt_fgenesh4_pg.C_1450045))	estExt_fgenesh4_pg.C_1450045
At5g20040	((At5g20040), (grail3.0032015901))	grail3.0032015901
At5g20280	((At5g20280), (estExt_Genewise1_v1.C_1520214, eugene3.00181112))	estExt_Genewise1_v1.C_1520214, eugene3.00181112
At5g20410	((At5g20410), (fgenesh4_pm.C_scaffold_152000030))	fgenesh4_pm.C_scaffold_152000030
At5g20830	((At5g20830, At3g43190), (estExt_fgenesh4_pg.C_280066))	estExt_fgenesh4_pg.C_280066
At5g20990	((At5g20990), (eugene3.00090140))	eugene3.00090140
At5g21060	((At5g21060), (eugene3.00660115))	eugene3.00660115
At5g21105	((At5g21105), (gw1.I.700.1))	gw1.I.700.1
At5g22510	((At5g22510), (eugene3.00150408))	eugene3.00150408
At5g22800	((At5g22800), (estExt_fgenesh4_pg.C_LG_IX0259))	estExt_fgenesh4_pg.C_LG_IX0259
At5g23250	((At5g23250), (estExt_fgenesh4_pg.C_LG_X0618))	estExt_fgenesh4_pg.C_LG_X0618
At5g23300	((At5g23300), (estExt_fgenesh4_pg.C_LG_V0296))	estExt_fgenesh4_pg.C_LG_V0296
At5g23670	((At5g23670, At3g48780), (estExt_fgenesh4_pm.C_LG_XII0379, estExt_Genewise1_v1.C_LG_XV2341))	estExt_fgenesh4_pm.C_LG_XII0379, estExt_Genewise1_v1.C_LG_XV2341
At5g25900	((At5g25900), (grail3.0009040502))	grail3.0009040502
At5g26030	((At5g26030), (gw1.XI.1296.1, gw1.IV.1600.1))	gw1.XI.1296.1, gw1.IV.1600.1
At5g26310	((At5g66690, At5g26310), (eugene3.00071041, gw1.VII.3365.1))	eugene3.00071041, gw1.VII.3365.1
At5g26667	((At5g26667), (estExt_Genewise1_v1.C_400378, eugene3.00021246))	estExt_Genewise1_v1.C_400378, eugene3.00021246

At5g26710	((At5g49930, At5g26710), (fgenesh4_pg.C_LG_VIII000894, gw1.131.39.1))	fgenesh4_pg.C_LG_VIII000894, gw1.131.39.1
At5g26780	((At5g26780), (grail3.0003095602))	grail3.0003095602
At5g26830	((At5g26830), (gw1.V.2553.1))	gw1.V.2553.1
At5g27450	((At5g27450), (gw1.70.604.1))	gw1.70.604.1
At5g27470	((At5g27470), (estExt_Genewise1_v1.C_LG_V3371))	estExt_Genewise1_v1.C_LG_V3371
At5g27530	((At5g27530), (eugene3.09330001))	eugene3.09330001
At5g27600	((At5g27600), (eugene3.00130195))	eugene3.00130195
At5g28020	((At5g28030, At5g28020), (eugene3.00700109, eugene3.00130335))	eugene3.00700109, eugene3.00130335
At5g28030	((At5g28030, At5g28020), (eugene3.00700109, eugene3.00130335))	eugene3.00700109, eugene3.00130335
At5g35360	((At5g35360), (gw1.2858.2.1))	gw1.2858.2.1
At5g35630	((At5g35630), (estExt_fgenesh4_pg.C_LG_VIII1790, estExt_Genewise1_v1.C_LG_X4165))	estExt_fgenesh4_pg.C_LG_VIII1790, estExt_Genewise1_v1.C_LG_X4165
At5g35790	((At5g35790), (estExt_Genewise1_v1.C_LG_II4018))	estExt_Genewise1_v1.C_LG_II4018
At5g36160	((At5g36160), (eugene3.00070098))	eugene3.00070098
At5g36700	((At5g36790, At5g36700), (eugene3.00660153, eugene3.00090081, gw1.X.4936.1))	eugene3.00660153, eugene3.00090081, gw1.X.4936.1
At5g36790	((At5g36790, At5g36700), (eugene3.00660153, eugene3.00090081, gw1.X.4936.1))	eugene3.00660153, eugene3.00090081, gw1.X.4936.1
At5g36880	((At5g36880), (eugene3.00140876))	eugene3.00140876
At5g37180	((At5g37180), (fgenesh4_pm.C_LG_IV000286, fgenesh4_pg.C_LG_IV000763, eugene3.00440147))	fgenesh4_pm.C_LG_IV000286, fgenesh4_pg.C_LG_IV000763, [illegible]
At5g37510	((At5g37510), (estExt_Genewise1_v1.C_LG_IV3721))	estExt_Genewise1_v1.C_LG_IV3721
At5g37600	((At5g37600, At1g66200), (estExt_fgenesh4_pm.C_LG_XII0003, estExt_fgenesh4_pg.C_1220090, estExt_Genewise1_v1.C_LG_II2125))	estExt_fgenesh4_pm.C_LG_XII0003, estExt_fgenesh4_pg.C_1220090, [illegible]
At5g37690	((At5g37690), (gw1.IV.3612.1))	gw1.IV.3612.1
At5g37710	((At5g37710), (gw1.IV.3606.1))	gw1.IV.3606.1
At5g38410	((At5g38430, At5g38420, At5g38410, At1g67090), (gw1.XV.1174.1))	gw1.XV.1174.1
At5g38420	((At5g38430, At5g38420, At5g38410, At1g67090), (gw1.XV.1174.1))	gw1.XV.1174.1
At5g38430	((At5g38430, At5g38420, At5g38410, At1g67090), (gw1.XV.1174.1))	gw1.XV.1174.1
At5g38630	((At5g38630), (estExt_Genewise1_v1.C_1230153))	estExt_Genewise1_v1.C_1230153
At5g38710	((At5g38710), (eugene3.00070994))	eugene3.00070994
At5g38830	((At5g38830), (estExt_fgenesh4_pm.C_LG_X0474))	estExt_fgenesh4_pm.C_LG_X0474
At5g39320	((At5g39320, At5g15490, At3g29360), (estExt_fgenesh4_pm.C_LG_XVII0156, eugene3.00041110))	estExt_fgenesh4_pm.C_LG_XVII0156, eugene3.00041110

At5g39910	((At5g39910), (gw1.XV.1899.1))	gw1.XV.1899.1
At5g40280	((At5g40280), (eugene3.04410005))	eugene3.04410005
At5g40390	((At5g40390), (eugene3.00640058))	eugene3.00640058
At5g40650	((At5g40650, At3g27380), (estExt_fgenesh4_pg.C_LG_I2224))	estExt_fgenesh4_pg.C_LG_I2224
At5g40760	((At5g40760, At3g27300), (estExt_Genewise1_v1.C_LG_XVII0625, grail3.0054015801))	estExt_Genewise1_v1.C_LG_XVII0625, grail3.0054015801
At5g40870	((At5g40870, At3g27190), (fgenesh4_pg.C_scaffold_88000094))	fgenesh4_pg.C_scaffold_88000094
At5g40990	((At5g40990, At3g14225), (fgenesh4_pg.C_LG_III000507))	fgenesh4_pg.C_LG_III000507
At5g41670	((At5g41670, At1g64190), (fgenesh4_pm.C_LG_III000453, grail3.0008041101))	fgenesh4_pm.C_LG_III000453, grail3.0008041101
At5g42650	((At5g42650, At3g18810, At1g49270), (fgenesh4_pm.C_LG_II000599, gw1.IX.1845.1))	fgenesh4_pm.C_LG_II000599, gw1.IX.1845.1
At5g42800	((At5g42800), (estExt_Genewise1_v1.C_LG_II0799, gw1.V.1411.1))	estExt_Genewise1_v1.C_LG_II0799, gw1.V.1411.1
At5g42810	((At5g42810), (estExt_fgenesh4_pg.C_LG_V1435))	estExt_fgenesh4_pg.C_LG_V1435
At5g43330	((At5g43330, At1g04410), (estExt_Genewise1_v1.C_2730019, eugene3.00081537))	estExt_Genewise1_v1.C_2730019, eugene3.00081537
At5g43850	((At5g43850), (estExt_Genewise1_v1.C_LG_VIII2401))	estExt_Genewise1_v1.C_LG_VIII2401
At5g43860	((At5g43860, At1g70210), (eugene3.00150918))	eugene3.00150918
At5g44030	((At5g44030), (eugene3.00002636))	eugene3.00002636
At5g44520	((At5g44520, At1g80840), (gw1.X.6047.1))	gw1.X.6047.1
At5g45930	((At4g18480, At5g45930), (gw1.IV.2852.1))	gw1.IV.2852.1
At5g45960	((At5g45960), (gw1.41.577.1))	gw1.41.577.1
At5g46180	((At5g46180), (estExt_Genewise1_v1.C_LG_XV2426))	estExt_Genewise1_v1.C_LG_XV2426
At5g47500	((At5g47500, At2g17230), (estExt_fgenesh4_pg.C_LG_V0044))	estExt_fgenesh4_pg.C_LG_V0044
At5g47720	((At5g47720), (eugene3.07970004))	eugene3.07970004
At5g47760	((At5g47760), (eugene3.01370007))	eugene3.01370007
At5g47810	((At5g47810), (gw1.137.65.1))	gw1.137.65.1
At5g48140	((At5g48140, At3g07840, At3g07830, At3g07820), (fgenesh4_pm.C_LG_VII000366))	fgenesh4_pm.C_LG_VII000366
At5g48230	((At5g48230), (estExt_Genewise1_v1.C_LG_XIV3807))	estExt_Genewise1_v1.C_LG_XIV3807
At5g48300	((At5g48300), (eugene3.00141188))	eugene3.00141188
At5g48370	((At5g48370), (estExt_fgenesh4_pm.C_LG_II1112, fgenesh4_pg.C_LG_XIV001138))	estExt_fgenesh4_pm.C_LG_II1112, fgenesh4_pg.C_LG_XIV001138
At5g48840	((At5g48840), (gw1.I.3546.1))	gw1.I.3546.1

At5g48930	((At5g48930), (eugene3.02080010))	eugene3.02080010
At5g48960	((At5g48960), (gw1.XV.3036.1))	gw1.XV.3036.1
At5g49460	((At5g49460, At3g06650), (grail3.0010034901))	grail3.0010034901
At5g49650	((At5g49650), (fgenesh4_pg.C_LG_II001033, grail3.0048004901))	fgenesh4_pg.C_LG_II001033, grail3.0048004901
At5g49720	((At5g49720), (estExt_fgenesh4_pg.C_LG_I0683, grail3.0263001401))	estExt_fgenesh4_pg.C_LG_I0683, grail3.0263001401
At5g50370	((At5g50370), (estExt_fgenesh4_pm.C_LG_XV0275))	estExt_fgenesh4_pm.C_LG_XV0275
At5g50375	((At5g50375), (gw1.I.1204.1))	gw1.I.1204.1
At5g50850	((At5g50850, At1g08250), (estExt_fgenesh4_pm.C_LG_I0194))	estExt_fgenesh4_pm.C_LG_I0194
At5g51460	((At5g51460), (estExt_Genewise1_v1.C_LG_XV2838))	estExt_Genewise1_v1.C_LG_XV2838
At5g51810	((At5g51810), (estExt_fgenesh4_pg.C_LG_XV1053, fgenesh4_pg.C_LG_XII001220))	estExt_fgenesh4_pg.C_LG_XV1053, fgenesh4_pg.C_LG_XII001220
At5g51820	((At5g51820), (gw1.I.9492.1))	gw1.I.9492.1
At5g52570	((At5g52570), (estExt_fgenesh4_pg.C_440224))	estExt_fgenesh4_pg.C_440224
At5g52840	((At5g52840), (grail3.0044018701))	grail3.0044018701
At5g52920	((At5g52920), (eugene3.00120107))	eugene3.00120107
At5g53120	((At5g53120), (fgenesh4_pg.C_LG_XII000148, estExt_Genewise1_v1.C_7280001))	fgenesh4_pg.C_LG_XII000148, estExt_Genewise1_v1.C_7280001
At5g53460	((At5g53460), (estExt_fgenesh4_pg.C_LG_XV0172, eugene3.06620001))	estExt_fgenesh4_pg.C_LG_XV0172, eugene3.06620001
At5g54080	((At5g54080), (estExt_Genewise1_v1.C_LG_X2572))	estExt_Genewise1_v1.C_LG_X2572
At5g54160	((At5g54160), (estExt_fgenesh4_pm.C_LG_XII0129))	estExt_fgenesh4_pm.C_LG_XII0129
At5g54690	((At5g54690), (estExt_fgenesh4_pm.C_LG_XIII0357, eugene3.00111083))	estExt_fgenesh4_pm.C_LG_XIII0357, eugene3.00111083
At5g54810	((At5g54810, At4g27070, At4g13700, At4g11920), (gw1.I.9225.1))	gw1.I.9225.1
At5g55590	((At5g19730, At5g55590), (estExt_fgenesh4_pg.C_1450045))	estExt_fgenesh4_pg.C_1450045
At5g55810	((At5g55810), (fgenesh4_pm.C_scaffold_308000002, gw1.70.673.1, gw1.XIII.983.1))	fgenesh4_pm.C_scaffold_308000002, gw1.70.673.1, gw1.XIII.983.1
At5g56350	((At5g56350, At4g21060, At1g78610), (fgenesh4_pm.C_scaffold_28000055))	fgenesh4_pm.C_scaffold_28000055
At5g56630	((At5g56630, At4g26270), (estExt_Genewise1_v1.C_1450141))	estExt_Genewise1_v1.C_1450141
At5g56680	((At5g56680, At1g70980), (gw1.XVIII.2695.1))	gw1.XVIII.2695.1
At5g56760	((At5g56760), (estExt_fgenesh4_pm.C_LG_XV0472, estExt_fgenesh4_pg.C_410108))	estExt_fgenesh4_pm.C_LG_XV0472, estExt_fgenesh4_pg.C_410108
At5g57590	((At5g57590), (fgenesh4_pg.C_LG_XIV000034))	fgenesh4_pg.C_LG_XIV000034
At5g57655	((At5g57655), (estExt_Genewise1_v1.C_LG_XVIII2895, gw1.118.85.1))	estExt_Genewise1_v1.C_LG_XVIII2895, gw1.118.85.1

At5g57850	((At5g57850), (estExt_fgenesh4_pm.C_LG_XI0275, eugene3.00700188))	estExt_fgenesh4_pm.C_LG_XI0275, eugene3.00700188
At5g58330	((At5g58330), (estExt_fgenesh4_pg.C_LG_VIII0243))	estExt_fgenesh4_pg.C_LG_VIII0243
At5g58560	((At5g58560), (eugene3.00100788, eugene3.00081451))	eugene3.00100788, eugene3.00081451
At5g59590	((At5g59590, At3g46690, At3g46680), (eugene3.00011560))	eugene3.00011560
At5g59750	((At5g59750), (gw1.X.6002.1))	gw1.X.6002.1
At5g60540	((At5g60540), (estExt_Genewise1_v1.C_LG_IX4777))	estExt_Genewise1_v1.C_LG_IX4777
At5g60600	((At5g60600), (estExt_fgenesh4_pg.C_LG_IX1415))	estExt_fgenesh4_pg.C_LG_IX1415
At5g61410	((At5g61410), (estExt_Genewise1_v1.C_1290095))	estExt_Genewise1_v1.C_1290095
At5g61540	((At4g00590, At5g61540), (estExt_fgenesh4_pm.C_LG_XIV0105))	estExt_fgenesh4_pm.C_LG_XIV0105
At5g61760	((At5g61760), (gw1.XV.2524.1))	gw1.XV.2524.1
At5g62530	((At5g62530), (estExt_fgenesh4_pg.C_LG_XII0768))	estExt_fgenesh4_pg.C_LG_XII0768
At5g62575	((At5g62575, At2g40170), (gw1.X.1681.1))	gw1.X.1681.1
At5g62790	((At5g62790), (estExt_Genewise1_v1.C_LG_XV1514, estExt_Genewise1_v1.C_LG_XII0355))	estExt_Genewise1_v1.C_LG_XV1514, estExt_Genewise1_v1.C_LG_XII0355
At5g62980	((At5g62980), (estExt_fgenesh4_pg.C_LG_XVI0540))	estExt_fgenesh4_pg.C_LG_XVI0540
At5g63400	((At5g63400), (estExt_fgenesh4_pg.C_LG_XII0928))	estExt_fgenesh4_pg.C_LG_XII0928
At5g63510	((At5g63510), (grail3.0015028601))	grail3.0015028601
At5g63570	((At5g63570), (eugene3.00150799, gw1.XII.1787.1))	eugene3.00150799, gw1.XII.1787.1
At5g63680	((At5g63680, At5g08570), (grail3.0106001101, eugene3.00080009))	grail3.0106001101, eugene3.00080009
At5g63890	((At5g63890), (estExt_fgenesh4_pm.C_1400029, eugene3.00013048))	estExt_fgenesh4_pm.C_1400029, eugene3.00013048
At5g64050	((At5g64050), (fgenesh4_pm.C_LG_IX000007))	fgenesh4_pm.C_LG_IX000007
At5g64300	((At5g64300), (gw1.I.1636.1))	gw1.I.1636.1
At5g64370	((At5g64370), (estExt_fgenesh4_pg.C_LG_VII0287))	estExt_fgenesh4_pg.C_LG_VII0287
At5g64440	((At5g64440, At5g40200), (estExt_Genewise1_v1.C_LG_V0611))	estExt_Genewise1_v1.C_LG_V0611
At5g64860	((At5g64860), (gw1.VII.2145.1))	gw1.VII.2145.1
At5g65010	((At5g65010, At5g10240), (gw1.V.2829.1))	gw1.V.2829.1
At5g65110	((At5g65110), (estExt_fgenesh4_pm.C_LG_VII0199))	estExt_fgenesh4_pm.C_LG_VII0199
At5g65140	((At5g65140), (fgenesh4_pg.C_LG_XII000191))	fgenesh4_pg.C_LG_XII000191
At5g65685	((At5g65685), (gw1.IV.3363.1))	gw1.IV.3363.1

At5g65720	((At5g65720), (grail3.0035009402))	grail3.0035009402
At5g65750	((At5g65750, At3g55410), (estExt_fgenesh4_pg.C_LG_VIII0421, estExt_Genewise1_v1.C_LG_X2288))	estExt_fgenesh4_pg.C_LG_VIII0421, estExt_Genewise1_v1.C_LG_X2288
At5g65940	((At5g65940, At3g20320, At2g30660, At2g30650), (estExt_Genewise1_v1.C_LG_XIV4108))	estExt_Genewise1_v1.C_LG_XIV4108
At5g66120	((At5g66120), (gw1.XVI.1270.1))	gw1.XVI.1270.1
At5g66690	((At5g66690, At5g26310), (eugene3.00071041, gw1.VII.3365.1))	eugene3.00071041, gw1.VII.3365.1
At5g66760	((At5g66760), (gw1.VII.3485.1))	gw1.VII.3485.1
At5g67030	((At5g67030), (fgenesh4_pg.C_LG_V000519))	fgenesh4_pg.C_LG_V000519
At5g67590	((At5g67590), (estExt_Genewise1_v1.C_LG_XV2896))	estExt_Genewise1_v1.C_LG_XV2896
