# Supplementary material for: Genes encoding hub and bottleneck enzymes of the Arabidopsis metabolic network preferentially retain homeologs through whole genome duplication
Source: BMC Evol Biol. 2010 May 18;10:145. doi: 10.1186/1471-2148-10-145 (PMC2880986; doi:10.1186/1471-2148-10-145)
Supplement: Additional file 5 — Table S5. The identified ortholog groups between Arabidopsis and rice by phylogenetic trees. [file 1471-2148-10-145-S5.PDF]

**Table S5. The identified ortholog groups between *Arabidopsis* and rice by phylogenetic tree.**

| *Arabidopsis* metabolic gene | Ortholog groups | Orthologs in rice |
|---|---|---|
| At1g01090 | ((At1g01090),(LOC_Os04g02900)) | LOC_Os04g02900 |
| At1g01120 | ((At1g01120),(LOC_Os03g10350)) | LOC_Os03g10350 |
| At1g01220 | ((At1g01220),(LOC_Os03g02410)) | LOC_Os03g02410 |
| At1g01710 | ((At1g01710),(LOC_Os04g47120)) | LOC_Os04g47120 |
| At1g02460 | ((At1g02460,At4g01890),(LOC_Os01g19170)) | LOC_Os01g19170 |
| At1g02660 | ((At1g02660),(LOC_Os01g15000)) | LOC_Os01g15000 |
| At1g02730 | ((At1g02730),(LOC_Os12g36890)) | LOC_Os12g36890 |
| At1g03090 | ((At1g03090),(LOC_Os12g41250)) | LOC_Os12g41250 |
| At1g03310 | ((At1g03310),(LOC_Os01g40870)) | LOC_Os01g40870 |
| At1g03475 | ((At1g03475),(LOC_Os01g12560)) | LOC_Os01g12560 |
| At1g04290 | ((At1g04290),(LOC_Os01g61680)) | LOC_Os01g61680 |
| At1g04410 | ((At5g43330,At1g04410),(LOC_Os10g33800)) | LOC_Os10g33800 |
| At1g04710 | ((At1g04710),(LOC_Os02g57260)) | LOC_Os02g57260 |
| At1g04920 | ((At1g04920),(LOC_Os01g69030)) | LOC_Os01g69030 |
| At1g05010 | ((At1g05010),(LOC_Os06g05520)) | LOC_Os06g05520 |
| At1g05160 | ((At1g05160),(LOC_Os03g61700)) | LOC_Os03g61700 |
| At1g05310 | ((At1g13450,At1g05310),(LOC_Os08g10604)) | LOC_Os08g10604 |
| At1g05790 | ((At4g33330,At1g05790),(LOC_Os01g67420)) | LOC_Os01g67420 |
| At1g06290 | ((At1g06310,At1g06290),(LOC_Os06g24704)) | LOC_Os06g24704 |
| At1g06310 | ((At1g06310,At1g06290),(LOC_Os06g24704)) | LOC_Os06g24704 |
| At1g06410 | ((At1g06410),(LOC_Os01g53000,LOC_Os01g54560,LOC_Os05g44100)) | LOC_Os01g53000,LOC_Os01g54560,LOC_Os05g44100 |
| At1g06550 | ((At1g06550),(LOC_Os01g54860)) | LOC_Os01g54860 |
| At1g06570 | ((At1g06570),(LOC_Os06g10770)) | LOC_Os06g10770 |
| At1g06800 | ((At1g06800),(LOC_Os07g46580)) | LOC_Os07g46580 |

| | | |
|---|---|---|
| At1g07240 | ((At1g07260,At1g07250,At1g07240),(LOC_Os03g53690,LOC_Os07g32020,LOC_Os07g32620,LOC_Os07g32630,LOC_Os07g37690)) | LOC_Os03g53690,LOC_Os07g32020,LOC_Os07g32620,LOC_Os07g32630,LOC_Os07g37690 |
| At1g07260 | ((At1g07260,At1g07250,At1g07240),(LOC_Os03g53690,LOC_Os07g32020,LOC_Os07g32620,LOC_Os07g32630,LOC_Os07g37690)) | LOC_Os03g53690,LOC_Os07g32020,LOC_Os07g32620,LOC_Os07g32630,LOC_Os07g37690 |
| At1g07890 | ((At1g07890),(LOC_Os03g17690)) | LOC_Os03g17690 |
| At1g08110 | ((At1g08110),(LOC_Os05g28190)) | LOC_Os05g28190 |
| At1g08480 | ((At1g08480),(LOC_Os08g02080)) | LOC_Os08g02080 |
| At1g08490 | ((At1g08490),(LOC_Os12g18900)) | LOC_Os12g18900 |
| At1g08510 | ((At1g08510),(LOC_Os02g43090,LOC_Os06g05130,LOC_Os11g43820)) | LOC_Os02g43090,LOC_Os06g05130,LOC_Os11g43820 |
| At1g08520 | ((At1g08520),(LOC_Os03g59640)) | LOC_Os03g59640 |
| At1g08550 | ((At1g08550),(LOC_Os04g31040)) | LOC_Os04g31040 |
| At1g08630 | ((At1g08630),(LOC_Os04g43650)) | LOC_Os04g43650 |
| At1g08980 | ((At1g08980),(LOC_Os04g02780)) | LOC_Os04g02780 |
| At1g09350 | ((At1g09350),(LOC_Os07g48830)) | LOC_Os07g48830 |
| At1g09430 | ((At1g09430),(LOC_Os11g47330,LOC_Os12g37870)) | LOC_Os11g47330,LOC_Os12g37870 |
| At1g09780 | ((At1g09780),(LOC_Os02g50330,LOC_Os09g06560)) | LOC_Os02g50330,LOC_Os09g06560 |
| At1g09830 | ((At1g09830),(LOC_Os08g09210)) | LOC_Os08g09210 |
| At1g09940 | ((At1g58290,At1g09940),(LOC_Os10g35840)) | LOC_Os10g35840 |
| At1g10400 | ((At1g10400,At2g16890),(LOC_Os09g21160)) | LOC_Os09g21160 |
| At1g11680 | ((At1g11680),(LOC_Os11g32240)) | LOC_Os11g32240 |
| At1g11720 | ((At1g11720),(LOC_Os04g53310,LOC_Os08g09230)) | LOC_Os04g53310,LOC_Os08g09230 |
| At1g11860 | ((At1g11860),(LOC_Os04g53230)) | LOC_Os04g53230 |
| At1g12000 | ((At1g12000),(LOC_Os06g13810)) | LOC_Os06g13810 |
| At1g12010 | ((At1g12010,At1g62380),(LOC_Os09g27750,LOC_Os09g27820)) | LOC_Os09g27750,LOC_Os09g27820 |
| At1g12050 | ((At1g12050),(LOC_Os03g63910)) | LOC_Os03g63910 |
| At1g12240 | ((At1g12240),(LOC_Os04g45290)) | LOC_Os04g45290 |
| At1g12350 | ((At1g12350),(LOC_Os08g07880)) | LOC_Os08g07880 |
| At1g12900 | ((At3g26650,At1g12900),(LOC_Os03g51900,LOC_Os04g38600)) | LOC_Os03g51900,LOC_Os04g38600 |
| At1g13440 | ((At4g39620,At3g04120,At1g13440),(LOC_Os01g16900)) | LOC_Os01g16900 |

| | | |
|---|---|---|
| At1g13560 | ((At3g25585,At1g13560),(LOC_Os02g02750)) | LOC_Os02g02750 |
| At1g14290 | ((At1g14290,At1g69640),(LOC_Os06g12250)) | LOC_Os06g12250 |
| At1g14810 | ((At1g14810),(LOC_Os03g55280)) | LOC_Os03g55280 |
| At1g15080 | ((At1g15080),(LOC_Os08g27030,LOC_Os08g27040)) | LOC_Os08g27030,LOC_Os08g27040 |
| At1g15110 | ((At1g15110),(LOC_Os01g02890)) | LOC_Os01g02890 |
| At1g15550 | ((At1g15550),(LOC_Os02g44220)) | LOC_Os02g44220 |
| At1g15710 | ((At5g01690,At4g01770,At4g01750,At4g01220,At1g15710),(LOC_Os10g30420)) | LOC_Os10g30420 |
| At1g15950 | ((At1g15950,At1g80820),(LOC_Os02g07350,LOC_Os09g25150)) | LOC_Os02g07350,LOC_Os09g25150 |
| At1g16300 | ((At1g42970,At1g16300),(LOC_Os12g13950)) | LOC_Os12g13950 |
| At1g16350 | ((At1g79470,At1g16350),(LOC_Os08g33390)) | LOC_Os08g33390 |
| At1g16700 | ((At1g79010,At1g16700),(LOC_Os03g56300)) | LOC_Os03g56300 |
| At1g17050 | ((At1g17050),(LOC_Os12g17320)) | LOC_Os12g17320 |
| At1g17290 | ((At1g17290,At1g72330),(LOC_Os07g42600)) | LOC_Os07g42600 |
| At1g17410 | ((At1g17410),(LOC_Os02g35700)) | LOC_Os02g35700 |
| At1g17420 | ((At1g17420),(LOC_Os03g08220)) | LOC_Os03g08220 |
| At1g17650 | ((At1g17650),(LOC_Os03g46740)) | LOC_Os03g46740 |
| At1g17890 | ((At1g17890),(LOC_Os06g44270)) | LOC_Os06g44270 |
| At1g18270 | ((At1g18270),(LOC_Os06g14740)) | LOC_Os06g14740 |
| At1g18460 | ((At1g73920,At1g18460),(LOC_Os06g03930)) | LOC_Os06g03930 |
| At1g18590 | ((At1g18590),(LOC_Os04g44460)) | LOC_Os04g44460 |
| At1g19200 | ((At1g19200,At1g74940),(LOC_Os02g07820)) | LOC_Os02g07820 |
| At1g19300 | ((At1g19300),(LOC_Os03g47530)) | LOC_Os03g47530 |
| At1g20050 | ((At1g20050),(LOC_Os01g01369)) | LOC_Os01g01369 |
| At1g20330 | ((At1g20330,At1g76090),(LOC_Os03g04340)) | LOC_Os03g04340 |
| At1g20510 | ((At1g20510),(LOC_Os05g38570)) | LOC_Os05g38570 |
| At1g20575 | ((At1g20575),(LOC_Os03g60939)) | LOC_Os03g60939 |
| At1g20630 | ((At1g20630,At4g35090),(LOC_Os03g03910)) | LOC_Os03g03910 |
| At1g20950 | ((At1g20950),(LOC_Os06g22060)) | LOC_Os06g22060 |

| | | |
|---|---|---|
| At1g22020 | ((At4g38270,At1g02020),(LOC_Os10g21890)) | LOC_Os10g21890 |
| At1g22340 | ((At1g22400,At1g22380,At1g22340),(LOC_Os02g51900)) | LOC_Os02g51900 |
| At1g22360 | ((At1g22360),(LOC_Os02g51910)) | LOC_Os02g51910 |
| At1g22400 | ((At1g22400,At1g22380,At1g22340),(LOC_Os02g51900)) | LOC_Os02g51900 |
| At1g22940 | ((At1g22940),(LOC_Os12g09000)) | LOC_Os12g09000 |
| At1g23190 | ((At1g23190),(LOC_Os03g50480)) | LOC_Os03g50480 |
| At1g23460 | ((At1g70500,At1g23460),(LOC_Os09g29170)) | LOC_Os09g29170 |
| At1g23820 | ((At1g23820),(LOC_Os12g13570)) | LOC_Os12g13570 |
| At1g23870 | ((At1g60140,At1g23870),(LOC_Os09g20990)) | LOC_Os09g20990 |
| At1g24170 | ((At1g24170,At5g14180),(LOC_Os02g58070,LOC_Os03g60 240)) | LOC_Os02g58070,LOC_Os03g60240 |
| At1g24180 | ((At1g24180,At1g59900),(LOC_Os06g13720)) | LOC_Os06g13720 |
| At1g24280 | ((At1g24280),(LOC_Os07g22350)) | LOC_Os07g22350 |
| At1g24360 | ((At1g24360),(LOC_Os04g30760)) | LOC_Os04g30760 |
| At1g25350 | ((At1g25350),(LOC_Os01g09000,LOC_Os05g08990)) | LOC_Os01g09000,LOC_Os05g08990 |
| At1g27450 | ((At1g27450),(LOC_Os12g39860)) | LOC_Os12g39860 |
| At1g28570 | ((At1g28570,At2g27360,At4g01290),(LOC_Os09g16090)) | LOC_Os09g16090 |
| At1g28580 | ((At1g28580,At1g28650),(LOC_Os01g42730,LOC_Os01g46 120)) | LOC_Os01g42730,LOC_Os01g46120 |
| At1g28610 | ((At1g28600,At1g28610),(LOC_Os06g47600)) | LOC_Os06g47600 |
| At1g29810 | ((At1g29810),(LOC_Os01g47420)) | LOC_Os01g47420 |
| At1g29880 | ((At1g29880),(LOC_Os02g41650,LOC_Os04g43800)) | LOC_Os02g41650,LOC_Os04g43800 |
| At1g30040 | ((At1g30040),(LOC_Os05g43880,LOC_Os05g48700)) | LOC_Os05g43880,LOC_Os05g48700 |
| At1g30120 | ((At2g34590,At1g30120),(LOC_Os12g42230)) | LOC_Os12g42230 |
| At1g30530 | ((At1g30530),(LOC_Os04g42690)) | LOC_Os04g42690 |
| At1g30620 | ((At1g30620),(LOC_Os07g04690)) | LOC_Os07g04690 |
| At1g31220 | ((At1g66520,At4g17360,At1g31220),(LOC_Os09g04490)) | LOC_Os09g04490 |
| At1g31230 | ((At1g31230),(LOC_Os01g72310,LOC_Os08g25390)) | LOC_Os01g72310,LOC_Os08g25390 |
| At1g31480 | ((At1g31480),(LOC_Os07g07070)) | LOC_Os07g07070 |
| At1g31800 | ((At1g31800),(LOC_Os01g11160)) | LOC_Os01g11160 |

| | | |
|---|---|---|
| At1g31860 | ((At1g31860),(LOC_Os01g16940)) | LOC_Os01g16940 |
| At1g32060 | ((At1g32060),(LOC_Os04g20164)) | LOC_Os04g20164 |
| At1g32200 | ((At1g32200),(LOC_Os08g25460)) | LOC_Os08g25460 |
| At1g32440 | ((At1g32440),(LOC_Os02g50040)) | LOC_Os02g50040 |
| At1g32470 | ((At1g32470),(LOC_Os10g37180)) | LOC_Os10g37180 |
| At1g34430 | ((At1g34430),(LOC_Os12g08170)) | LOC_Os12g08170 |
| At1g35580 | ((At1g35580),(LOC_Os02g34560)) | LOC_Os02g34560 |
| At1g36160 | ((At1g36160),(LOC_Os10g21910)) | LOC_Os10g21910 |
| At1g36280 | ((At1g36280),(LOC_Os02g56520)) | LOC_Os02g56520 |
| At1g36370 | ((At1g36370,At1g22020),(LOC_Os05g23600)) | LOC_Os05g23600 |
| At1g41830 | ((At1g41830),(LOC_Os07g32660)) | LOC_Os07g32660 |
| At1g42970 | ((At1g42970,At1g16300),(LOC_Os12g13950)) | LOC_Os12g13950 |
| At1g43670 | ((At1g43670),(LOC_Os05g36270)) | LOC_Os05g36270 |
| At1g43710 | ((At1g43710),(LOC_Os02g33710)) | LOC_Os02g33710 |
| At1g47260 | ((At1g47260),(LOC_Os12g07220)) | LOC_Os12g07220 |
| At1g47420 | ((At1g47420),(LOC_Os02g15760,LOC_Os08g41320)) | LOC_Os02g15760,LOC_Os08g41320 |
| At1g47840 | ((At1g47840),(LOC_Os11g31110)) | LOC_Os11g31110 |
| At1g48030 | ((At3g17240,At1g48030),(LOC_Os01g22520,LOC_Os05g06750)) | LOC_Os01g22520,LOC_Os05g06750 |
| At1g48100 | ((At1g48100),(LOC_Os01g46230)) | LOC_Os01g46230 |
| At1g48320 | ((At1g48320),(LOC_Os03g48480)) | LOC_Os03g48480 |
| At1g48850 | ((At1g48850),(LOC_Os03g14990)) | LOC_Os03g14990 |
| At1g48860 | ((At5g35170,At1g48860),(LOC_Os09g39570)) | LOC_Os09g39570 |
| At1g49430 | ((At1g49430),(LOC_Os04g52290,LOC_Os11g35400)) | LOC_Os04g52290,LOC_Os11g35400 |
| At1g50200 | ((At1g50200),(LOC_Os10g10244)) | LOC_Os10g10244 |
| At1g50460 | ((At1g50460,At3g20040),(LOC_Os01g71320)) | LOC_Os01g71320 |
| At1g51680 | ((At1g51680),(LOC_Os08g14760)) | LOC_Os08g14760 |
| At1g51760 | ((At1g51780,At1g51760),(LOC_Os01g37960)) | LOC_Os01g37960 |
| At1g52570 | ((At3g15730,At1g52570),(LOC_Os01g07760,LOC_Os05g07880)) | LOC_Os01g07760,LOC_Os05g07880 |

| | | |
|---|---|---|
| At1g53310 | ((At3g14940,At1g53310),(LOC_Os08g27840,LOC_Os09g14670)) | LOC_Os08g27840,LOC_Os09g14670 |
| At1g53830 | ((At3g14310,At1g53830),(LOC_Os05g50460)) | LOC_Os05g50460 |
| At1g55020 | ((At1g55020),(LOC_Os03g49260,LOC_Os03g52860)) | LOC_Os03g49260,LOC_Os03g52860 |
| At1g55850 | ((At1g55850),(LOC_Os02g49332)) | LOC_Os02g49332 |
| At1g59900 | ((At1g61130,At1g59900),(LOC_Os06g13720)) | LOC_Os06g13720 |
| At1g60140 | ((At1g60140,At1g23870),(LOC_Os09g20990)) | LOC_Os09g20990 |
| At1g60390 | ((At1g60390),(LOC_Os08g29200)) | LOC_Os08g29200 |
| At1g60590 | ((At1g60590),(LOC_Os06g01460)) | LOC_Os06g01460 |
| At1g61720 | ((At2g18850,At1g61720),(LOC_Os04g53780,LOC_Os04g53800,LOC_Os04g53810,LOC_Os04g53830,LOC_Os04g5385[illegible] | LOC_Os04g53780,LOC_Os04g53800,LOC_Os04g53810,LOC_Os04g53830,LOC_Os04g53850,LO[illegible] |
| At1g62380 | ((At1g12010,At1g62380),(LOC_Os09g27750,LOC_Os09g27820)) | LOC_Os09g27750,LOC_Os09g27820 |
| At1g62940 | ((At1g62940),(LOC_Os03g30260)) | LOC_Os03g30260 |
| At1g63290 | ((At1g63290),(LOC_Os09g32810)) | LOC_Os09g32810 |
| At1g63770 | ((At1g63770),(LOC_Os08g44860)) | LOC_Os08g44860 |
| At1g64440 | ((At1g64440,At4g23920),(LOC_Os04g43690)) | LOC_Os04g43690 |
| At1g65930 | ((At1g65930),(LOC_Os01g46610)) | LOC_Os01g46610 |
| At1g66520 | ((At1g66520,At4g17360,At1g31220),(LOC_Os09g04490)) | LOC_Os09g04490 |
| At1g66530 | ((At1g66530,At4g26300),(LOC_Os01g06510)) | LOC_Os01g06510 |
| At1g67070 | ((At1g67070),(LOC_Os06g02620,LOC_Os06g51050,LOC_Os09g22090)) | LOC_Os06g02620,LOC_Os06g51050,LOC_Os09g22090 |
| At1g67090 | ((At5g38430,At5g38420,At5g38410,At1g67090),(LOC_Os10g35190)) | LOC_Os10g35190 |
| At1g68010 | ((At1g68010),(LOC_Os02g01150)) | LOC_Os02g01150 |
| At1g68020 | ((At1g68020),(LOC_Os09g23350)) | LOC_Os09g23350 |
| At1g68460 | ((At1g68460,At1g69210),(LOC_Os02g31160,LOC_Os07g06090,LOC_Os11g11100,LOC_Os12g09250)) | LOC_Os02g31160,LOC_Os07g06090,LOC_Os11g11100,LOC_Os12g09250 |
| At1g68530 | ((At1g25450,At1g68530),(LOC_Os03g12030)) | LOC_Os03g12030 |
| At1g68750 | ((At1g68750),(LOC_Os01g02050)) | LOC_Os01g02050 |
| At1g69190 | ((At1g69190),(LOC_Os01g29150,LOC_Os06g39880)) | LOC_Os01g29150,LOC_Os06g39880 |
| At1g69640 | ((At1g14290,At1g69640),(LOC_Os06g12250)) | LOC_Os06g12250 |
| At1g69740 | ((At1g69740),(LOC_Os01g72930)) | LOC_Os01g72930 |
| At1g70290 | ((At1g70290),(LOC_Os08g31980)) | LOC_Os08g31980 |

| | | |
|---|---|---|
| At1g70310 | ((At5g19530,At1g70310),(LOC_Os01g58870,LOC_Os02g49770,LOC_Os05g41480)) | LOC_Os01g58870,LOC_Os02g49770,LOC_Os05g41480 |
| At1g70500 | ((At1g70500,At1g23460),(LOC_Os09g29170)) | LOC_Os09g29170 |
| At1g70710 | ((At1g70710,At1g23210),(LOC_Os02g50040)) | LOC_Os02g50040 |
| At1g70730 | ((At1g23190,At1g70730),(LOC_Os03g50480)) | LOC_Os03g50480 |
| At1g71920 | ((At5g10330,At1g71920),(LOC_Os08g25010)) | LOC_Os08g25010 |
| At1g72330 | ((At1g17290,At1g72330),(LOC_Os07g42600)) | LOC_Os07g42600 |
| At1g72520 | ((At1g72520),(LOC_Os03g08220)) | LOC_Os03g08220 |
| At1g72550 | ((At1g72550),(LOC_Os05g48510)) | LOC_Os05g48510 |
| At1g72680 | ((At1g72680),(LOC_Os10g11810,LOC_Os11g40690)) | LOC_Os10g11810,LOC_Os11g40690 |
| At1g72810 | ((At1g72810,At1g13630),(LOC_Os05g47640)) | LOC_Os05g47640 |
| At1g73250 | ((At1g73250),(LOC_Os03g17740)) | LOC_Os03g17740 |
| At1g73370 | ((At1g73370),(LOC_Os02g58480,LOC_Os04g17650,LOC_Os04g24430)) | LOC_Os02g58480,LOC_Os04g17650,LOC_Os04g24430 |
| At1g73880 | ((At1g73880),(LOC_Os06g17260)) | LOC_Os06g17260 |
| At1g73920 | ((At1g73920,At1g18460),(LOC_Os06g03930)) | LOC_Os06g03930 |
| At1g74030 | ((At1g74030),(LOC_Os09g20820)) | LOC_Os09g20820 |
| At1g74100 | ((At1g74100),(LOC_Os01g20950)) | LOC_Os01g20950 |
| At1g74320 | ((At1g74320),(LOC_Os05g45880)) | LOC_Os05g45880 |
| At1g74470 | ((At1g74470),(LOC_Os05g07090)) | LOC_Os05g07090 |
| At1g74710 | ((At1g74710,At1g24290),(LOC_Os02g12570,LOC_Os02g50680)) | LOC_Os02g12570,LOC_Os02g50680 |
| At1g74910 | ((At1g74910),(LOC_Os03g11050)) | LOC_Os03g11050 |
| At1g75880 | ((At1g05900,At1g44575,At1g75880,At1g75890,At2g38860,At3g54600),(LOC_Os11g16580)) | LOC_Os11g16580 |
| At1g75890 | ((At1g05900,At1g44575,At1g75880,At1g75890,At2g38860,At3g54600),(LOC_Os11g16580)) | LOC_Os11g16580 |
| At1g76090 | ((At1g20330,At1g76090),(LOC_Os03g04340)) | LOC_Os03g04340 |
| At1g76130 | ((At1g76130),(LOC_Os04g33040)) | LOC_Os04g33040 |
| At1g76490 | ((At1g76490),(LOC_Os03g05310)) | LOC_Os03g05310 |
| At1g76550 | ((At1g76550),(LOC_Os02g48360)) | LOC_Os02g48360 |
| At1g76680 | ((At1g76680),(LOC_Os06g11240)) | LOC_Os06g11240 |
| At1g77120 | ((At1g77120),(LOC_Os11g10480,LOC_Os11g10510)) | LOC_Os11g10480,LOC_Os11g10510 |

| | | |
|---|---|---|
| At1g77490 | ((At1g61590,At1g77490),(LOC_Os06g48510)) | LOC_Os06g48510 |
| At1g77590 | ((At1g77590),(LOC_Os03g49380,LOC_Os12g07110)) | LOC_Os03g49380,LOC_Os12g07110 |
| At1g78270 | ((At1g22340,At1g22380,At1g22400,At1g78270),(LOC_Os02g51900)) | LOC_Os02g51900 |
| At1g78570 | ((At1g78570),(LOC_Os03g17000)) | LOC_Os03g17000 |
| At1g78580 | ((At1g78580),(LOC_Os05g44210)) | LOC_Os05g44210 |
| At1g78660 | ((At1g78660),(LOC_Os11g01890)) | LOC_Os11g01890 |
| At1g78680 | ((At1g78680),(LOC_Os03g41100)) | LOC_Os03g41100 |
| At1g79010 | ((At1g79010,At1g16700),(LOC_Os03g56300)) | LOC_Os03g56300 |
| At1g79460 | ((At3g29410,At1g79460),(LOC_Os04g10060)) | LOC_Os04g10060 |
| At1g79470 | ((At1g16350,At1g79470),(LOC_Os08g33390)) | LOC_Os08g33390 |
| At1g79530 | ((At1g79530),(LOC_Os12g13950)) | LOC_Os12g13950 |
| At1g79550 | ((At1g79550),(LOC_Os06g45710)) | LOC_Os06g45710 |
| At1g80050 | ((At1g80050,At5g11160),(LOC_Os03g59480,LOC_Os12g02510)) | LOC_Os03g59480,LOC_Os12g02510 |
| At1g80460 | ((At1g80460),(LOC_Os04g55410)) | LOC_Os04g55410 |
| At1g80820 | ((At1g80820),(LOC_Os02g07350,LOC_Os09g25150)) | LOC_Os02g07350,LOC_Os09g25150 |
| At2g01140 | ((At2g01140),(LOC_Os01g02880)) | LOC_Os01g02880 |
| At2g02050 | ((At2g02050),(LOC_Os03g43910)) | LOC_Os03g43910 |
| At2g02500 | ((At2g02500),(LOC_Os08g41910)) | LOC_Os08g41910 |
| At2g04350 | ((At2g04350),(LOC_Os05g25310)) | LOC_Os05g25310 |
| At2g04842 | ((At2g04842),(LOC_Os02g33500)) | LOC_Os02g33500 |
| At2g05260 | ((At2g05260),(LOC_Os02g18480)) | LOC_Os02g18480 |
| At2g05710 | ((At2g05710),(LOC_Os08g09200)) | LOC_Os08g09200 |
| At2g05990 | ((At2g05990),(LOC_Os08g23810)) | LOC_Os08g23810 |
| At2g06050 | ((At5g20380,At2g06050),(LOC_Os08g35740)) | LOC_Os08g35740 |
| At2g07050 | ((At2g07050),(LOC_Os02g04710)) | LOC_Os02g04710 |
| At2g07751 | ((At2g07751),(LOC_Os04g43540)) | LOC_Os04g43540 |
| At2g13360 | ((At2g13360),(LOC_Os12g37630)) | LOC_Os12g37630 |
| At2g15230 | ((At2g15230),(LOC_Os12g05080)) | LOC_Os12g05080 |

| | | |
|---|---|---|
| At2g15480 | ((At2g15480,At2g15490,At4g34131,At4g34135,At4g34138),(LOC_Os01g41430,LOC_Os01g41450,LOC_Os01g45110,L [illegible] | LOC_Os01g41430,LOC_Os01g41450,LOC_Os01g45110,LOC_Os01g45140 |
| At2g15490 | ((At2g15490),(LOC_Os01g41430,LOC_Os01g41450,LOC_Os01g45110,LOC_Os01g45140)) | LOC_Os01g41430,LOC_Os01g41450,LOC_Os01g45110,LOC_Os01g45140 |
| At2g16570 | ((At2g16570),(LOC_Os01g65260)) | LOC_Os01g65260 |
| At2g16890 | ((At1g10400,At2g16890),(LOC_Os09g21160)) | LOC_Os09g21160 |
| At2g17265 | ((At2g17265),(LOC_Os01g43490,LOC_Os06g04040)) | LOC_Os01g43490,LOC_Os06g04040 |
| At2g17630 | ((At2g17630),(LOC_Os09g19734)) | LOC_Os09g19734 |
| At2g17640 | ((At2g17640,At1g06240),(LOC_Os03g41438,LOC_Os03g49200)) | LOC_Os03g41438,LOC_Os03g49200 |
| At2g19450 | ((At2g19450),(LOC_Os09g19700)) | LOC_Os09g19700 |
| At2g20360 | ((At2g20360),(LOC_Os02g57180)) | LOC_Os02g57180 |
| At2g20420 | ((At2g20420),(LOC_Os02g40830)) | LOC_Os02g40830 |
| At2g21170 | ((At2g21170),(LOC_Os09g36450)) | LOC_Os09g36450 |
| At2g21610 | ((At2g21610),(LOC_Os09g19570)) | LOC_Os09g19570 |
| At2g21770 | ((At2g21770),(LOC_Os03g62090,LOC_Os07g14850,LOC_Os07g24190)) | LOC_Os03g62090,LOC_Os07g14850,LOC_Os07g24190 |
| At2g21790 | ((At2g21790),(LOC_Os02g56100,LOC_Os06g07210)) | LOC_Os02g56100,LOC_Os06g07210 |
| At2g22330 | ((At2g22330),(LOC_Os04g08828)) | LOC_Os04g08828 |
| At2g22570 | ((At2g22570),(LOC_Os02g39400)) | LOC_Os02g39400 |
| At2g22910 | ((At2g22910),(LOC_Os06g43270)) | LOC_Os06g43270 |
| At2g22990 | ((At1g33540,At1g73270,At1g73280,At1g73290,At1g73300,At1g73310,At2g22920,At2g22970,At2g22980,At2g22990,At [illegible] | LOC_Os04g09720,LOC_Os11g42390 |
| At2g23420 | ((At4g16310,At2g23420),(LOC_Os03g62110)) | LOC_Os03g62110 |
| At2g23890 | ((At2g23890),(LOC_Os09g25640)) | LOC_Os09g25640 |
| At2g23900 | ((At2g23900),(LOC_Os06g08170)) | LOC_Os06g08170 |
| At2g24630 | ((At4g31590,At2g24630),(LOC_Os09g25900)) | LOC_Os09g25900 |
| At2g25540 | ((At2g25540,At4g32410),(LOC_Os05g08370)) | LOC_Os05g08370 |
| At2g26080 | ((At4g33010,At2g26080),(LOC_Os01g51410,LOC_Os06g40940)) | LOC_Os01g51410,LOC_Os06g40940 |
| At2g26450 | ((At4g33230,At2g26450),(LOC_Os08g34910,LOC_Os09g26360)) | LOC_Os08g34910,LOC_Os09g26360 |
| At2g26560 | ((At5g04980,At2g26560),(LOC_Os09g28770)) | LOC_Os09g28770 |
| At2g26800 | ((At2g26800),(LOC_Os03g17780)) | LOC_Os03g17780 |
| At2g27450 | ((At2g27450),(LOC_Os02g10320)) | LOC_Os02g10320 |

| | | |
|---|---|---|
| At2g27730 | ((At2g27730),(LOC_Os08g15170)) | LOC_Os08g15170 |
| At2g28760 | ((At2g28760),(LOC_Os03g16980)) | LOC_Os03g16980 |
| At2g28880 | ((At2g28880),(LOC_Os06g48620)) | LOC_Os06g48620 |
| At2g29560 | ((At2g29560),(LOC_Os03g15950)) | LOC_Os03g15950 |
| At2g29590 | ((At2g29590),(LOC_Os06g28970)) | LOC_Os06g28970 |
| At2g29710 | ((At1g07240,At1g07250,At1g07260,At2g29710,At2g29730),(LOC_Os03g53690,LOC_Os07g32020,LOC_Os07g32620,LOC_Os07g32630,LOC_Os07g37690)) | LOC_Os03g53690,LOC_Os07g32020,LOC_Os07g32620,LOC_Os07g32630,LOC_Os07g37690 |
| At2g29730 | ((At1g07240,At1g07250,At1g07260,At2g29710,At2g29730),(LOC_Os03g53690,LOC_Os07g32020,LOC_Os07g32620,LOC_Os07g32630,LOC_Os07g37690)) | LOC_Os03g53690,LOC_Os07g32020,LOC_Os07g32620,LOC_Os07g32630,LOC_Os07g37690 |
| At2g29740 | ((At2g29740,At2g29750),(LOC_Os07g31960)) | LOC_Os07g31960 |
| At2g29750 | ((At2g29740,At2g29750),(LOC_Os07g31960)) | LOC_Os07g31960 |
| At2g30140 | ((At2g30140),(LOC_Os11g31880)) | LOC_Os11g31880 |
| At2g30200 | ((At2g30200),(LOC_Os01g48270,LOC_Os10g42240)) | LOC_Os01g48270,LOC_Os10g42240 |
| At2g30390 | ((At2g30390),(LOC_Os06g08770)) | LOC_Os06g08770 |
| At2g30490 | ((At2g30490),(LOC_Os05g25640)) | LOC_Os05g25640 |
| At2g30550 | (((LOC_Os07g46580)) | LOC_Os07g46580 |
| At2g30970 | ((At2g30970),(LOC_Os02g14110)) | LOC_Os02g14110 |
| At2g31170 | ((At2g31170),(LOC_Os09g38420)) | LOC_Os09g38420 |
| At2g31690 | ((At2g31690),(LOC_Os11g19290)) | LOC_Os11g19290 |
| At2g32260 | ((At2g32260,At1g06620),(LOC_Os03g11520)) | LOC_Os03g11520 |
| At2g32530 | ((At4g15320,At4g15290,At2g32620,At2g32610,At2g32540,At2g32530),(LOC_Os10g20090)) | LOC_Os10g20090 |
| At2g32540 | ((At4g15320,At4g15290,At2g32620,At2g32610,At2g32540,At2g32530),(LOC_Os10g20090)) | LOC_Os10g20090 |
| At2g32610 | ((At4g15320,At4g15290,At2g32620,At2g32610,At2g32540,At2g32530),(LOC_Os10g20090)) | LOC_Os10g20090 |
| At2g32620 | ((At4g15320,At4g15290,At2g32620,At2g32610,At2g32540,At2g32530),(LOC_Os10g20090)) | LOC_Os10g20090 |
| At2g33100 | ((At2g33100),(LOC_Os06g22980)) | LOC_Os06g22980 |
| At2g33220 | ((At2g33220),(LOC_Os03g09210)) | LOC_Os03g09210 |
| At2g34555 | ((At2g34555),(LOC_Os01g55240)) | LOC_Os01g55240 |
| At2g34590 | ((At2g34590,At1g30120),(LOC_Os12g42230)) | LOC_Os12g42230 |
| At2g34630 | ((At2g34630),(LOC_Os07g17120)) | LOC_Os07g17120 |

| | | |
|---|---|---|
| At2g35120 | ((At2g35120),(LOC_Os02g57670)) | LOC_Os02g57670 |
| At2g35370 | ((At2g35370),(LOC_Os10g37180)) | LOC_Os10g37180 |
| At2g35500 | ((At2g35500),(LOC_Os10g42700)) | LOC_Os10g42700 |
| At2g35690 | ((At4g16760,At2g35690),(LOC_Os06g01390)) | LOC_Os06g01390 |
| At2g35840 | ((At2g35840),(LOC_Os01g27880,LOC_Os02g05030,LOC_Os05g05270)) | LOC_Os01g27880,LOC_Os02g05030,LOC_Os05g05270 |
| At2g36390 | ((At2g36390,At5g03650),(LOC_Os02g32660)) | LOC_Os02g32660 |
| At2g36460 | ((At3g52930,At2g36460),(LOC_Os01g67860,LOC_Os05g33380,LOC_Os10g08022)) | LOC_Os01g67860,LOC_Os05g33380,LOC_Os10g08022 |
| At2g36530 | ((At2g36530),(LOC_Os10g08550)) | LOC_Os10g08550 |
| At2g36580 | ((At3g52990,At2g36580),(LOC_Os11g05110)) | LOC_Os11g05110 |
| At2g36700 | ((At2g36700),(LOC_Os06g04150)) | LOC_Os06g04150 |
| At2g36710 | ((At2g36710),(LOC_Os04g57430)) | LOC_Os04g57430 |
| At2g37690 | ((At2g37690),(LOC_Os01g10280,LOC_Os06g01650)) | LOC_Os01g10280,LOC_Os06g01650 |
| At2g38050 | ((At2g38050),(LOC_Os01g05970)) | LOC_Os01g05970 |
| At2g38650 | ((At2g38650),(LOC_Os07g48370)) | LOC_Os07g48370 |
| At2g38670 | ((At2g38670),(LOC_Os11g03050,LOC_Os12g02820)) | LOC_Os11g03050,LOC_Os12g02820 |
| At2g39630 | ((At2g39630),(LOC_Os07g37840)) | LOC_Os07g37840 |
| At2g39770 | ((At2g39770),(LOC_Os01g62840,LOC_Os03g16150,LOC_Os08g13930)) | LOC_Os01g62840,LOC_Os03g16150,LOC_Os08g13930 |
| At2g39930 | ((At2g39930),(LOC_Os06g45120)) | LOC_Os06g45120 |
| At2g40890 | ((At2g40890),(LOC_Os03g31594)) | LOC_Os03g31594 |
| At2g41490 | ((At2g41490,At3g57220),(LOC_Os07g46640)) | LOC_Os07g46640 |
| At2g42010 | ((At2g42010),(LOC_Os10g38060)) | LOC_Os10g38060 |
| At2g42310 | ((At2g42310,At3g57785),(LOC_Os11g35050)) | LOC_Os11g35050 |
| At2g42790 | ((At2g42790,At2g44350),(LOC_Os01g64870,LOC_Os02g13840,LOC_Os02g51880)) | LOC_Os01g64870,LOC_Os02g13840,LOC_Os02g51880 |
| At2g42910 | ((At2g42910),(LOC_Os01g16600,LOC_Os02g48390,LOC_Os05g32060)) | LOC_Os01g16600,LOC_Os02g48390,LOC_Os05g32060 |
| At2g43430 | ((At2g43430),(LOC_Os09g34100)) | LOC_Os09g34100 |
| At2g44160 | ((At3g59970,At2g44160),(LOC_Os03g60090)) | LOC_Os03g60090 |
| At2g44350 | ((At2g42790,At2g44350),(LOC_Os01g64870,LOC_Os02g13840,LOC_Os02g51880)) | LOC_Os01g64870,LOC_Os02g13840,LOC_Os02g51880 |
| At2g44530 | ((At2g44530),(LOC_Os02g03540)) | LOC_Os02g03540 |

| | | |
|---|---|---|
| At2g45150 | ((At2g45150),(LOC_Os02g39920)) | LOC_Os02g39920 |
| At2g45290 | ((At3g60750,At2g45290),(LOC_Os06g04270)) | LOC_Os06g04270 |
| At2g45300 | ((At2g45300),(LOC_Os09g39570)) | LOC_Os09g39570 |
| At2g47180 | ((At2g47180),(LOC_Os03g20120)) | LOC_Os03g20120 |
| At2g47240 | ((At2g47240),(LOC_Os06g47210)) | LOC_Os06g47210 |
| At2g47550 | ((At2g47550),(LOC_Os01g20980,LOC_Os04g55050)) | LOC_Os01g20980,LOC_Os04g55050 |
| At2g47650 | ((At3g62830,At2g47650),(LOC_Os01g21320,LOC_Os05g29990)) | LOC_Os01g21320,LOC_Os05g29990 |
| At2g47690 | ((At2g47690),(LOC_Os03g17660)) | LOC_Os03g17660 |
| At3g01040 | ((At5g15470,At3g01040),(LOC_Os12g38930)) | LOC_Os12g38930 |
| At3g01850 | ((At1g63290,At3g01850),(LOC_Os09g32810)) | LOC_Os09g32810 |
| At3g02100 | ((At3g02100),(LOC_Os03g55020)) | LOC_Os03g55020 |
| At3g02230 | ((At5g15650,At3g02230),(LOC_Os03g40270)) | LOC_Os03g40270 |
| At3g02570 | ((At3g02570),(LOC_Os08g41890)) | LOC_Os08g41890 |
| At3g02870 | ((At3g02870),(LOC_Os03g39000)) | LOC_Os03g39000 |
| At3g02875 | ((At3g02875),(LOC_Os04g40080)) | LOC_Os04g40080 |
| At3g03050 | ((At5g16910,At3g03050),(LOC_Os06g02180)) | LOC_Os06g02180 |
| At3g03100 | ((At3g03100),(LOC_Os05g38640,LOC_Os10g42840)) | LOC_Os05g38640,LOC_Os10g42840 |
| At3g03630 | ((At3g03630),(LOC_Os08g09100)) | LOC_Os08g09100 |
| At3g03780 | ((At5g17920,At3g03780),(LOC_Os12g42876,LOC_Os12g42884)) | LOC_Os12g42876,LOC_Os12g42884 |
| At3g04120 | ((At4g39620,At3g04120,At1g13440),(LOC_Os01g16900)) | LOC_Os01g16900 |
| At3g04790 | ((At3g04790),(LOC_Os07g08030)) | LOC_Os07g08030 |
| At3g04870 | ((At3g04870),(LOC_Os06g29810)) | LOC_Os06g29810 |
| At3g05610 | ((At3g05610,At5g27870),(LOC_Os03g18860,LOC_Os07g49100)) | LOC_Os03g18860,LOC_Os07g49100 |
| At3g05970 | ((At3g05970),(LOC_Os11g04980,LOC_Os12g04990)) | LOC_Os11g04980,LOC_Os12g04990 |
| At3g06200 | ((At3g06200),(LOC_Os03g20460)) | LOC_Os03g20460 |
| At3g06260 | ((At3g06260),(LOC_Os03g24510)) | LOC_Os03g24510 |
| At3g06350 | ((At3g06350),(LOC_Os12g34874)) | LOC_Os12g34874 |
| At3g06650 | ((At5g49460,At3g06650),(LOC_Os01g19450)) | LOC_Os01g19450 |

| | | |
|---|---|---|
| At3g06860 | ((At3g06860),(LOC_Os04g54810)) | LOC_Os04g54810 |
| At3g07400 | ((At3g07400),(LOC_Os08g01390)) | LOC_Os08g01390 |
| At3g07630 | ((At3g07630),(LOC_Os03g17730,LOC_Os07g49390)) | LOC_Os03g17730,LOC_Os07g49390 |
| At3g07820 | ((At5g48140,At3g07840,At3g07830,At3g07820),(LOC_Os10g37899)) | LOC_Os10g37899 |
| At3g07840 | ((At5g48140,At3g07840,At3g07830,At3g07820),(LOC_Os10g37899)) | LOC_Os10g37899 |
| At3g08510 | ((At3g55940,At3g08510),(LOC_Os06g41110)) | LOC_Os06g41110 |
| At3g08590 | ((At3g08590),(LOC_Os02g50330,LOC_Os09g06560)) | LOC_Os02g50330,LOC_Os09g06560 |
| At3g08610 | ((At3g08610),(LOC_Os05g40300)) | LOC_Os05g40300 |
| At3g09930 | ((At4g39970,At3g09930),(LOC_Os08g33540)) | LOC_Os08g33540 |
| At3g10370 | ((At3g10370),(LOC_Os04g14790)) | LOC_Os04g14790 |
| At3g10700 | ((At3g10700),(LOC_Os04g51880)) | LOC_Os04g51880 |
| At3g10850 | ((At3g10850),(LOC_Os03g21460)) | LOC_Os03g21460 |
| At3g11170 | ((At3g11170,At5g05580),(LOC_Os04g24550)) | LOC_Os04g24550 |
| At3g11670 | ((At3g11670),(LOC_Os02g33580)) | LOC_Os02g33580 |
| At3g11710 | ((At3g11710),(LOC_Os01g47080)) | LOC_Os01g47080 |
| At3g11750 | ((At5g03050,At3g11750),(LOC_Os08g44210)) | LOC_Os08g44210 |
| At3g12120 | ((At3g12120),(LOC_Os06g07550)) | LOC_Os06g07550 |
| At3g12670 | ((At3g12670),(LOC_Os01g43020,LOC_Os05g49770)) | LOC_Os01g43020,LOC_Os05g49770 |
| At3g12780 | ((At3g12780),(LOC_Os05g41640)) | LOC_Os05g41640 |
| At3g14075 | ((At3g14075),(LOC_Os02g54010)) | LOC_Os02g54010 |
| At3g14130 | ((At3g14150,At3g14130),(LOC_Os07g42440)) | LOC_Os07g42440 |
| At3g14150 | ((At3g14130,At3g14150),(LOC_Os07g42440)) | LOC_Os07g42440 |
| At3g14310 | ((At3g14310,At1g53830),(LOC_Os05g50460)) | LOC_Os05g50460 |
| At3g14415 | ((At3g14420,At3g14415),(LOC_Os03g57220,LOC_Os07g05820)) | LOC_Os03g57220,LOC_Os07g05820 |
| At3g14420 | ((At3g14420,At3g14415),(LOC_Os03g57220,LOC_Os07g05820)) | LOC_Os03g57220,LOC_Os07g05820 |
| At3g14510 | ((At3g14510),(LOC_Os06g48200)) | LOC_Os06g48200 |
| At3g14530 | ((At3g14530,At3g14550,At3g32040),(LOC_Os07g39270)) | LOC_Os07g39270 |
| At3g14550 | ((At3g14530,At3g14550,At3g32040),(LOC_Os07g39270)) | LOC_Os07g39270 |

| | | |
|---|---|---|
| At3g14940 | ((At3g14940,At1g53310),(LOC_Os08g27840,LOC_Os09g14670)) | LOC_Os08g27840,LOC_Os09g14670 |
| At3g15730 | ((At3g15730,At1g52570),(LOC_Os01g07760,LOC_Os05g07880)) | LOC_Os01g07760,LOC_Os05g07880 |
| At3g16150 | ((At3g16150),(LOC_Os04g55710)) | LOC_Os04g55710 |
| At3g16175 | ((At3g16175),(LOC_Os02g57830)) | LOC_Os02g57830 |
| At3g16785 | ((At3g16785),(LOC_Os05g29050)) | LOC_Os05g29050 |
| At3g17060 | ((At3g17060),(LOC_Os03g19610)) | LOC_Os03g19610 |
| At3g17240 | ((At3g17240,At1g48030),(LOC_Os01g22520,LOC_Os05g06750)) | LOC_Os01g22520,LOC_Os05g06750 |
| At3g18000 | ((At3g18000),(LOC_Os12g26290)) | LOC_Os12g26290 |
| At3g18680 | ((At3g18680),(LOC_Os01g73450)) | LOC_Os01g73450 |
| At3g19160 | ((At3g19160),(LOC_Os03g18620)) | LOC_Os03g18620 |
| At3g19820 | ((At3g19820,At1g03100),(LOC_Os10g25780)) | LOC_Os10g25780 |
| At3g20040 | ((At3g20040),(LOC_Os01g71320)) | LOC_Os01g71320 |
| At3g20440 | ((At1g69220,At3g20440),(LOC_Os03g54780,LOC_Os06g26234)) | LOC_Os03g54780,LOC_Os06g26234 |
| At3g20480 | ((At3g20480),(LOC_Os11g05100)) | LOC_Os11g05100 |
| At3g21230 | ((At3g21230),(LOC_Os08g39370)) | LOC_Os08g39370 |
| At3g21560 | ((At3g21560,At4g15480,At4g15500),(LOC_Os01g48580,LOC_Os05g48380)) | LOC_Os01g48580,LOC_Os05g48380 |
| At3g21720 | ((At3g21720),(LOC_Os07g34520)) | LOC_Os07g34520 |
| At3g21730 | ((At3g21730),(LOC_Os07g03310,LOC_Os07g03400,LOC_Os07g03490)) | LOC_Os07g03310,LOC_Os07g03400,LOC_Os07g03490 |
| At3g21760 | ((At3g21760),(LOC_Os07g32010)) | LOC_Os07g32010 |
| At3g22200 | ((At3g22200),(LOC_Os08g23780)) | LOC_Os08g23780 |
| At3g22250 | ((At3g22250),(LOC_Os05g03550,LOC_Os06g33320)) | LOC_Os05g03550,LOC_Os06g33320 |
| At3g22360 | ((At3g22360),(LOC_Os08g01890)) | LOC_Os08g01890 |
| At3g22400 | ((At3g22400),(LOC_Os03g49380,LOC_Os12g07110)) | LOC_Os03g49380,LOC_Os12g07110 |
| At3g22425 | ((At3g12040,At3g22425),(LOC_Os02g53430)) | LOC_Os02g53430 |
| At3g22960 | ((At3g22960),(LOC_Os01g25484,LOC_Os07g08340)) | LOC_Os01g25484,LOC_Os07g08340 |
| At3g23810 | ((At4g13940,At3g23810),(LOC_Os09g34010)) | LOC_Os09g34010 |
| At3g23820 | ((At5g65560,At3g23820),(LOC_Os10g37340)) | LOC_Os10g37340 |
| At3g23920 | ((At3g23920),(LOC_Os03g04770)) | LOC_Os03g04770 |

| | | |
|---|---|---|
| At3g24090 | ((At3g24090,At4g34740),(LOC_Os11g03900,LOC_Os12g03720)) | LOC_Os11g03900,LOC_Os12g03720 |
| At3g24130 | ((At1g23380,At3g24130,At5g18990),(LOC_Os01g19440)) | LOC_Os01g19440 |
| At3g24503 | ((At3g24503),(LOC_Os01g40860)) | LOC_Os01g40860 |
| At3g25530 | ((At3g25530),(LOC_Os02g35500)) | LOC_Os02g35500 |
| At3g25585 | ((At3g25585,At1g13560),(LOC_Os02g02750)) | LOC_Os02g02750 |
| At3g25860 | ((At3g25860),(LOC_Os08g33440,LOC_Os09g24320)) | LOC_Os08g33440,LOC_Os09g24320 |
| At3g26380 | ((At3g26380),(LOC_Os01g33420)) | LOC_Os01g33420 |
| At3g26650 | ((At3g26650,At1g12900),(LOC_Os03g51900,LOC_Os04g38600)) | LOC_Os03g51900,LOC_Os04g38600 |
| At3g27190 | ((At5g40870,At3g27190),(LOC_Os01g26039)) | LOC_Os01g26039 |
| At3g27300 | ((At5g40760,At3g27300),(LOC_Os02g38840)) | LOC_Os02g38840 |
| At3g27620 | ((At1g67070,At3g27620),(LOC_Os06g02620,LOC_Os06g51050,LOC_Os09g22090)) | LOC_Os06g02620,LOC_Os06g51050,LOC_Os09g22090 |
| At3g29090 | ((At3g29090),(LOC_Os01g47350)) | LOC_Os01g47350 |
| At3g29200 | ((At3g29200),(LOC_Os01g15110,LOC_Os05g05740,LOC_Os06g19980,LOC_Os07g37250,LOC_Os10g42550)) | LOC_Os01g15110,LOC_Os05g05740,LOC_Os06g19980,LOC_Os07g37250,LOC_Os10g42550 |
| At3g29360 | ((At5g39320,At5g15490,At3g29360),(LOC_Os03g55070,LOC_Os12g25690,LOC_Os12g25700)) | LOC_Os03g55070,LOC_Os12g25690,LOC_Os12g25700 |
| At3g30775 | ((At3g30775),(LOC_Os10g40360)) | LOC_Os10g40360 |
| At3g32040 | ((At3g14530,At3g14550,At3g32040),(LOC_Os07g39270)) | LOC_Os07g39270 |
| At3g43190 | ((At5g20830,At3g43190),(LOC_Os03g28330,LOC_Os06g09450,LOC_Os07g42490,LOC_Os07g44970)) | LOC_Os03g28330,LOC_Os06g09450,LOC_Os07g42490,LOC_Os07g44970 |
| At3g44880 | ((At4g36220,At3g44880),(LOC_Os10g36848,LOC_Os01g18320)) | LOC_Os10g36848,LOC_Os01g18320 |
| At3g45140 | ((At3g45140),(LOC_Os02g10120)) | LOC_Os02g10120 |
| At3g46440 | ((At4g14820,At3g46440),(LOC_Os03g16980)) | LOC_Os03g16980 |
| At3g46970 | ((At3g46970),(LOC_Os01g63270)) | LOC_Os01g63270 |
| At3g47450 | ((At3g47450),(LOC_Os02g01440)) | LOC_Os02g01440 |
| At3g47520 | ((At3g47520),(LOC_Os01g61380,LOC_Os08g33720)) | LOC_Os01g61380,LOC_Os08g33720 |
| At3g47833 | ((At3g47833,At5g62575),(LOC_Os09g21470)) | LOC_Os09g21470 |
| At3g48080 | ((At3g48090,At3g48080),(LOC_Os03g26630)) | LOC_Os03g26630 |
| At3g48090 | ((At3g48090,At3g48080),(LOC_Os03g26630)) | LOC_Os03g26630 |
| At3g48110 | ((At3g48110),(LOC_Os06g01400)) | LOC_Os06g01400 |
| At3g48560 | ((At3g48560),(LOC_Os04g32010)) | LOC_Os04g32010 |

| | | |
|---|---|---|
| At3g48730 | ((At3g48730),(LOC_Os08g41990)) | LOC_Os08g41990 |
| At3g51240 | ((At3g51240),(LOC_Os04g43410)) | LOC_Os04g43410 |
| At3g51520 | ((At3g51520),(LOC_Os09g37280)) | LOC_Os09g37280 |
| At3g51820 | ((At3g51820,At3g14930),(LOC_Os05g28200)) | LOC_Os05g28200 |
| At3g51840 | ((At3g51840),(LOC_Os01g06600)) | LOC_Os01g06600 |
| At3g52180 | ((At3g52180),(LOC_Os04g31120)) | LOC_Os04g31120 |
| At3g52930 | ((At3g52930,At2g36460),(LOC_Os01g67860,LOC_Os05g33380,LOC_Os10g08022)) | LOC_Os01g67860,LOC_Os05g33380,LOC_Os10g08022 |
| At3g52940 | ((At3g52940,At3g06190),(LOC_Os01g25189,LOC_Os09g39220)) | LOC_Os01g25189,LOC_Os09g39220 |
| At3g52990 | ((At3g52990,At2g36580),(LOC_Os11g05110)) | LOC_Os11g05110 |
| At3g53130 | ((At3g53130),(LOC_Os10g39930)) | LOC_Os10g39930 |
| At3g53150 | ((At3g53150),(LOC_Os01g08110)) | LOC_Os01g08110 |
| At3g53580 | ((At3g53580),(LOC_Os06g05980,LOC_Os12g31480)) | LOC_Os06g05980,LOC_Os12g31480 |
| At3g53900 | ((At3g53900),(LOC_Os03g15870)) | LOC_Os03g15870 |
| At3g54050 | ((At3g54050),(LOC_Os03g16050)) | LOC_Os03g16050 |
| At3g54470 | ((At3g54470),(LOC_Os01g72250)) | LOC_Os01g72250 |
| At3g55360 | ((At3g55360),(LOC_Os01g05670)) | LOC_Os01g05670 |
| At3g55410 | ((At5g65750,At3g55410),(LOC_Os07g49520)) | LOC_Os07g49520 |
| At3g55440 | ((At3g55440),(LOC_Os01g05490)) | LOC_Os01g05490 |
| At3g55610 | ((At3g55610,At2g39800),(LOC_Os05g38150)) | LOC_Os05g38150 |
| At3g55700 | ((At3g55710,At3g55700),(LOC_Os03g55050)) | LOC_Os03g55050 |
| At3g55710 | ((At3g55710,At3g55700),(LOC_Os03g55050)) | LOC_Os03g55050 |
| At3g55800 | ((At3g55800),(LOC_Os04g16680)) | LOC_Os04g16680 |
| At3g56310 | ((At3g56310),(LOC_Os07g48160)) | LOC_Os07g48160 |
| At3g56940 | ((At3g56940),(LOC_Os01g17170)) | LOC_Os01g17170 |
| At3g57220 | ((At3g57220),(LOC_Os07g46640)) | LOC_Os07g46640 |
| At3g57560 | ((At3g57560),(LOC_Os04g46460)) | LOC_Os04g46460 |
| At3g57610 | ((At3g57610),(LOC_Os03g07840,LOC_Os03g49220)) | LOC_Os03g07840,LOC_Os03g49220 |
| At3g57785 | ((At2g42310,At3g57785),(LOC_Os11g35050)) | LOC_Os11g35050 |

| | | |
|---|---|---|
| At3g58140 | ((At3g58140),(LOC_Os03g57660,LOC_Os12g34860)) | LOC_Os03g57660,LOC_Os12g34860 |
| At3g58750 | ((At3g58750),(LOC_Os01g64870,LOC_Os02g13840,LOC_Os02g51880)) | LOC_Os01g64870,LOC_Os02g13840,LOC_Os02g51880 |
| At3g59760 | ((At3g59760),(LOC_Os09g38755)) | LOC_Os09g38755 |
| At3g59850 | ((At3g59850),(LOC_Os02g43750)) | LOC_Os02g43750 |
| At3g60620 | ((At3g12400,At3g60620,At5g13860),(LOC_Os02g58640,LOC_Os05g46520)) | LOC_Os02g58640,LOC_Os05g46520 |
| At3g60750 | ((At3g60750,At2g45290),(LOC_Os06g04270)) | LOC_Os06g04270 |
| At3g61130 | ((At3g61130),(LOC_Os09g36180,LOC_Os09g36190)) | LOC_Os09g36180,LOC_Os09g36190 |
| At3g61200 | ((At3g61200),(LOC_Os03g43420,LOC_Os07g15500,LOC_Os07g27870,LOC_Os07g27960)) | LOC_Os03g43420,LOC_Os07g15500,LOC_Os07g27870,LOC_Os07g27960 |
| At3g61680 | ((At3g61680),(LOC_Os07g39090)) | LOC_Os07g39090 |
| At3g62110 | ((At3g62110),(LOC_Os03g61800)) | LOC_Os03g61800 |
| At3g62410 | ((At3g62410),(LOC_Os03g17490,LOC_Os03g19380)) | LOC_Os03g17490,LOC_Os03g19380 |
| At3g62660 | ((At3g62660),(LOC_Os03g18890)) | LOC_Os03g18890 |
| At3g62830 | ((At3g62830,At2g47650),(LOC_Os01g21320,LOC_Os05g29990)) | LOC_Os01g21320,LOC_Os05g29990 |
| At3g63250 | ((At3g63250),(LOC_Os02g05840)) | LOC_Os02g05840 |
| At3g63410 | ((At3g63410),(LOC_Os07g08200,LOC_Os07g47830)) | LOC_Os07g08200,LOC_Os07g47830 |
| At4g00110 | ((At4g00110),(LOC_Os03g14540)) | LOC_Os03g14540 |
| At4g00490 | ((At4g00490),(LOC_Os05g40740)) | LOC_Os05g40740 |
| At4g00590 | ((At4g00590,At5g61540),(LOC_Os04g58600)) | LOC_Os04g58600 |
| At4g01890 | ((At3g07970,At4g01890),(LOC_Os01g19170)) | LOC_Os01g19170 |
| At4g02120 | ((At4g02120),(LOC_Os01g23610)) | LOC_Os01g23610 |
| At4g02280 | ((At4g02280),(LOC_Os03g22120)) | LOC_Os03g22120 |
| At4g02580 | ((At4g02580),(LOC_Os08g38610)) | LOC_Os08g38610 |
| At4g02780 | ((At4g02780),(LOC_Os02g17780)) | LOC_Os02g17780 |
| At4g05160 | ((At4g05160),(LOC_Os11g02620,LOC_Os12g02540)) | LOC_Os11g02620,LOC_Os12g02540 |
| At4g07960 | ((At4g07960),(LOC_Os01g56130,LOC_Os05g43530)) | LOC_Os01g56130,LOC_Os05g43530 |
| At4g08390 | ((At4g08390,At4g11860),(LOC_Os05g19630)) | LOC_Os05g19630 |
| At4g08790 | ((At4g08790),(LOC_Os01g58380,LOC_Os06g01690)) | LOC_Os01g58380,LOC_Os06g01690 |
| At4g08900 | ((At4g08900),(LOC_Os04g01590)) | LOC_Os04g01590 |

| | | |
|---|---|---|
| At4g10120 | ((At4g10120),(LOC_Os11g12810)) | LOC_Os11g12810 |
| At4g11010 | ((At4g11010),(LOC_Os05g51700)) | LOC_Os05g51700 |
| At4g11280 | ((At4g11280),(LOC_Os03g08410)) | LOC_Os03g08410 |
| At4g11820 | ((At4g11820),(LOC_Os03g02710,LOC_Os08g43170,LOC_Os09g34960)) | LOC_Os03g02710,LOC_Os08g43170,LOC_Os09g34960 |
| At4g12440 | ((At4g12440),(LOC_Os09g35860)) | LOC_Os09g35860 |
| At4g13780 | ((At4g13780),(LOC_Os06g31210)) | LOC_Os06g31210 |
| At4g13940 | ((At4g13940,At3g23810),(LOC_Os09g34010)) | LOC_Os09g34010 |
| At4g14090 | ((At4g14090,At4g15550),(LOC_Os06g39330)) | LOC_Os06g39330 |
| At4g14210 | ((At4g14210),(LOC_Os03g08570)) | LOC_Os03g08570 |
| At4g14716 | ((At4g14716,At4g14710,At2g26400),(LOC_Os03g06620)) | LOC_Os03g06620 |
| At4g14910 | ((At4g14910),(LOC_Os04g52710)) | LOC_Os04g52710 |
| At4g15210 | ((At4g15210),(LOC_Os03g10350)) | LOC_Os03g10350 |
| At4g15290 | ((At4g15320,At4g15290,At2g32620,At2g32610,At2g32540,At2g32530),(LOC_Os10g20090)) | LOC_Os10g20090 |
| At4g15320 | ((At4g15320,At4g15290,At2g32620,At2g32610,At2g32540,At2g32530),(LOC_Os10g20090)) | LOC_Os10g20090 |
| At4g15480 | ((At3g21560,At4g15480,At4g15500),(LOC_Os01g48580,LOC_Os05g48380)) | LOC_Os01g48580,LOC_Os05g48380 |
| At4g15490 | ((At4g15490),(LOC_Os02g09510)) | LOC_Os02g09510 |
| At4g15500 | ((At3g21560,At4g15480,At4g15500),(LOC_Os01g48580,LOC_Os05g48380)) | LOC_Os01g48580,LOC_Os05g48380 |
| At4g15550 | ((At4g14090,At4g15550),(LOC_Os06g39330)) | LOC_Os06g39330 |
| At4g15560 | ((At4g15560),(LOC_Os05g33840)) | LOC_Os05g33840 |
| At4g16070 | ((At4g16070),(LOC_Os01g20840)) | LOC_Os01g20840 |
| At4g16130 | ((At4g16130),(LOC_Os02g04840,LOC_Os06g48940)) | LOC_Os02g04840,LOC_Os06g48940 |
| At4g16760 | ((At4g16760,At2g35690),(LOC_Os06g01390)) | LOC_Os06g01390 |
| At4g17090 | ((At4g17090),(LOC_Os01g55300,LOC_Os04g46570)) | LOC_Os01g55300,LOC_Os04g46570 |
| At4g17190 | ((At4g17190),(LOC_Os09g28400)) | LOC_Os09g28400 |
| At4g17260 | ((At4g17260),(LOC_Os07g46630)) | LOC_Os07g46630 |
| At4g17300 | ((At4g17300),(LOC_Os07g30200)) | LOC_Os07g30200 |
| At4g17360 | ((At1g66520,At4g17360,At1g31220),(LOC_Os09g04490)) | LOC_Os09g04490 |
| At4g18180 | ((At4g18180,At5g42180),(LOC_Os05g14300,LOC_Os06g07020,LOC_Os08g23790,LOC_Os09g12310)) | LOC_Os05g14300,LOC_Os06g07020,LOC_Os08g23790,LOC_Os09g12310 |

| | | |
|---|---|---|
| At4g18480 | ((At4g18480,At5g45930),(LOC_Os01g62070,LOC_Os04g36062)) | LOC_Os01g62070,LOC_Os04g36062 |
| At4g18780 | ((At4g18780),(LOC_Os01g54620)) | LOC_Os01g54620 |
| At4g19710 | ((At4g19710),(LOC_Os09g12290)) | LOC_Os09g12290 |
| At4g20670 | ((At4g20670),(LOC_Os08g34340)) | LOC_Os08g34340 |
| At4g20960 | ((At4g20960),(LOC_Os01g62380)) | LOC_Os01g62380 |
| At4g22930 | ((At4g22930),(LOC_Os01g54370)) | LOC_Os01g54370 |
| At4g23660 | ((At4g23660),(LOC_Os04g44290)) | LOC_Os04g44290 |
| At4g23900 | ((At4g23900),(LOC_Os01g16940)) | LOC_Os01g16940 |
| At4g23920 | ((At4g23920),(LOC_Os04g43690)) | LOC_Os04g43690 |
| At4g25000 | ((At5g47860,At4g25000,At1g56710),(LOC_Os08g36900)) | LOC_Os08g36900 |
| At4g25570 | ((At4g25570),(LOC_Os02g42890)) | LOC_Os02g42890 |
| At4g26200 | ((At4g26200),(LOC_Os02g15690)) | LOC_Os02g15690 |
| At4g26270 | ((At5g56630,At4g26270),(LOC_Os06g05860,LOC_Os07g12530)) | LOC_Os06g05860,LOC_Os07g12530 |
| At4g26520 | ((At3g10350,At4g26520,At4g26530),(LOC_Os06g40640)) | LOC_Os06g40640 |
| At4g26530 | ((At4g26530),(LOC_Os06g40640)) | LOC_Os06g40640 |
| At4g26770 | ((At4g26770),(LOC_Os04g57550)) | LOC_Os04g57550 |
| At4g27070 | ((At5g54810,At4g27070,At4g13700,At4g11920),(LOC_Os08g04180)) | LOC_Os08g04180 |
| At4g29010 | ((At4g29010),(LOC_Os02g17390)) | LOC_Os02g17390 |
| At4g29210 | ((At4g39650,At4g29210),(LOC_Os01g34080,LOC_Os03g03650)) | LOC_Os01g34080,LOC_Os03g03650 |
| At4g29460 | ((At4g29460,At4g29470),(LOC_Os02g58500)) | LOC_Os02g58500 |
| At4g29470 | ((At4g29460,At4g29470),(LOC_Os02g58500)) | LOC_Os02g58500 |
| At4g29540 | ((At4g29540),(LOC_Os07g05380)) | LOC_Os07g05380 |
| At4g29600 | ((At4g29600),(LOC_Os04g56750)) | LOC_Os04g56750 |
| At4g29840 | ((At4g29840),(LOC_Os01g49890)) | LOC_Os01g49890 |
| At4g30000 | ((At4g30000),(LOC_Os02g11020,LOC_Os03g08900)) | LOC_Os02g11020,LOC_Os03g08900 |
| At4g31180 | ((At4g31180),(LOC_Os02g46130)) | LOC_Os02g46130 |
| At4g31590 | ((At4g31590,At2g24630),(LOC_Os09g25900)) | LOC_Os09g25900 |
| At4g31990 | ((At4g31990),(LOC_Os02g55420)) | LOC_Os02g55420 |

| | | |
|---|---|---|
| At4g32180 | ((At4g32180),(LOC_Os09g36270)) | LOC_Os09g36270 |
| At4g32410 | ((At4g32410),(LOC_Os05g08370)) | LOC_Os05g08370 |
| At4g33010 | ((At4g33010,At2g26080),(LOC_Os01g51410,LOC_Os06g40940)) | LOC_Os01g51410,LOC_Os06g40940 |
| At4g33030 | ((At4g33030),(LOC_Os05g32140)) | LOC_Os05g32140 |
| At4g33070 | ((At4g33070,At5g01320,At5g01330,At5g54960),(LOC_Os03g18220)) | LOC_Os03g18220 |
| At4g33150 | ((At4g33150),(LOC_Os02g54254)) | LOC_Os02g54254 |
| At4g33220 | ((At4g33220,At5g01930),(LOC_Os08g34900)) | LOC_Os08g34900 |
| At4g33230 | ((At4g33230,At2g26450),(LOC_Os08g34910,LOC_Os09g26360)) | LOC_Os08g34910,LOC_Os09g26360 |
| At4g33440 | ((At3g16850,At4g33440),(LOC_Os08g01600)) | LOC_Os08g01600 |
| At4g33670 | ((At4g33670),(LOC_Os09g27040)) | LOC_Os09g27040 |
| At4g34030 | ((At4g34030),(LOC_Os01g56670)) | LOC_Os01g56670 |
| At4g34131 | ((At2g15480,At2g15490,At4g34131,At4g34135,At4g34138),(LOC_Os01g41430,LOC_Os01g41450,LOC_Os01g45110,L[illegible] | LOC_Os01g41430,LOC_Os01g41450,LOC_Os01g45110,LOC_Os01g45140 |
| At4g34135 | ((At2g15480,At2g15490,At4g34131,At4g34135,At4g34138),(LOC_Os01g41430,LOC_Os01g41450,LOC_Os01g45110,L[illegible] | LOC_Os01g41430,LOC_Os01g41450,LOC_Os01g45110,LOC_Os01g45140 |
| At4g34138 | ((At2g15480,At2g15490,At4g34131,At4g34135,At4g34138),(LOC_Os01g41430,LOC_Os01g41450,LOC_Os01g45110,L[illegible] | LOC_Os01g41430,LOC_Os01g41450,LOC_Os01g45110,LOC_Os01g45140 |
| At4g34200 | ((At4g34200),(LOC_Os02g42600)) | LOC_Os02g42600 |
| At4g34230 | ((At4g34230),(LOC_Os02g09490)) | LOC_Os02g09490 |
| At4g34350 | ((At4g34350),(LOC_Os12g13320)) | LOC_Os12g13320 |
| At4g34570 | ((At4g34570),(LOC_Os05g46200)) | LOC_Os05g46200 |
| At4g34640 | ((At4g34640),(LOC_Os07g10130)) | LOC_Os07g10130 |
| At4g34650 | ((At4g34640,At4g34650),(LOC_Os07g10130)) | LOC_Os07g10130 |
| At4g34710 | ((At4g34710),(LOC_Os06g04070)) | LOC_Os06g04070 |
| At4g34840 | ((At4g34840),(LOC_Os08g44280)) | LOC_Os08g44280 |
| At4g35000 | ((At4g35000),(LOC_Os08g43560)) | LOC_Os08g43560 |
| At4g35090 | ((At4g35090),(LOC_Os03g03910)) | LOC_Os03g03910 |
| At4g35360 | ((At4g35360),(LOC_Os06g21980)) | LOC_Os06g21980 |
| At4g35630 | ((At4g35630),(LOC_Os03g06200,LOC_Os04g41020)) | LOC_Os03g06200,LOC_Os04g41020 |
| At4g36220 | ((At4g36220,At3g44880),(LOC_Os10g36848,LOC_Os01g18320)) | LOC_Os10g36848,LOC_Os01g18320 |
| At4g36250 | ((At4g36250,At2g05940),(LOC_Os04g45720)) | LOC_Os04g45720 |

| | | |
|---|---|---|
| At4g36810 | ((At4g36810),(LOC_Os01g55795)) | LOC_Os01g55795 |
| At4g37000 | ((At4g37000),(LOC_Os05g39530)) | LOC_Os05g39530 |
| At4g37670 | ((At4g37670),(LOC_Os01g54270)) | LOC_Os01g54270 |
| At4g37870 | ((At4g37870),(LOC_Os03g15050)) | LOC_Os03g15050 |
| At4g37930 | ((At4g37930),(LOC_Os03g52840)) | LOC_Os03g52840 |
| At4g38270 | ((At4g38270,At1g02020),(LOC_Os10g21890)) | LOC_Os10g21890 |
| At4g38460 | ((At4g38460),(LOC_Os02g44780)) | LOC_Os02g44780 |
| At4g39280 | ((At4g39280),(LOC_Os10g26130)) | LOC_Os10g26130 |
| At4g39350 | ((At4g39350),(LOC_Os03g62090,LOC_Os07g14850,LOC_Os07g24190)) | LOC_Os03g62090,LOC_Os07g14850,LOC_Os07g24190 |
| At4g39640 | ((At4g39640),(LOC_Os01g65330,LOC_Os05g35530)) | LOC_Os01g65330,LOC_Os05g35530 |
| At4g39650 | ((At4g39650,At4g29210),(LOC_Os01g34080,LOC_Os03g03650)) | LOC_Os01g34080,LOC_Os03g03650 |
| At4g39830 | ((At4g39830),(LOC_Os09g20090)) | LOC_Os09g20090 |
| At4g39950 | ((At4g39950),(LOC_Os04g08824)) | LOC_Os04g08824 |
| At5g03650 | ((At5g03650),(LOC_Os02g32660)) | LOC_Os02g32660 |
| At5g03770 | ((At5g03770),(LOC_Os01g63840)) | LOC_Os01g63840 |
| At5g03820 | ((At5g03820,At5g03810),(LOC_Os03g47940)) | LOC_Os03g47940 |
| At5g04140 | ((At5g04140),(LOC_Os07g46460)) | LOC_Os07g46460 |
| At5g04590 | ((At5g04590),(LOC_Os01g20940)) | LOC_Os01g20940 |
| At5g05170 | ((At5g05170),(LOC_Os03g59340,LOC_Os07g10770)) | LOC_Os03g59340,LOC_Os07g10770 |
| At5g05270 | ((At5g05270),(LOC_Os11g02440,LOC_Os12g02370)) | LOC_Os11g02440,LOC_Os12g02370 |
| At5g05580 | ((At3g11170,At5g05580),(LOC_Os04g24550)) | LOC_Os04g24550 |
| At5g05870 | ((At3g55700,At3g55710,At5g05870,At5g05880,At5g05890,At5g05900),(LOC_Os03g55050)) | LOC_Os03g55050 |
| At5g05880 | ((At3g55700,At3g55710,At5g05870,At5g05880,At5g05890,At5g05900),(LOC_Os03g55050)) | LOC_Os03g55050 |
| At5g05890 | ((At3g55700,At3g55710,At5g05870,At5g05880,At5g05890,At5g05900),(LOC_Os03g55050)) | LOC_Os03g55050 |
| At5g05900 | ((At3g55700,At3g55710,At5g05870,At5g05880,At5g05890,At5g05900),(LOC_Os03g55050)) | LOC_Os03g55050 |
| At5g05980 | ((At5g05980),(LOC_Os09g28390)) | LOC_Os09g28390 |
| At5g07370 | ((At5g07370),(LOC_Os01g20206)) | LOC_Os01g20206 |
| At5g07440 | ((At5g07440),(LOC_Os02g43470)) | LOC_Os02g43470 |

| | | |
|---|---|---|
| At5g08300 | ((At5g08300),(LOC_Os07g38970)) | LOC_Os07g38970 |
| At5g08380 | ((At5g08380),(LOC_Os09g34970,LOC_Os11g13800,LOC_Os12g33194,LOC_Os12g36750)) | LOC_Os09g34970,LOC_Os11g13800,LOC_Os12g33194,LOC_Os12g36750 |
| At5g08530 | ((At5g08530),(LOC_Os07g45090)) | LOC_Os07g45090 |
| At5g08570 | ((At5g63680,At5g08570),(LOC_Os04g58110,LOC_Os10g39900)) | LOC_Os04g58110,LOC_Os10g39900 |
| At5g09300 | ((At5g09300),(LOC_Os01g32870)) | LOC_Os01g32870 |
| At5g09420 | ((At5g09420),(LOC_Os02g56540)) | LOC_Os02g56540 |
| At5g09640 | ((At1g33540,At1g73270,At1g73280,At1g73290,At1g73300,At1g73310,At2g22920,At2g22970,At2g22980,At2g22990,At[illegible] | LOC_Os04g09720,LOC_Os11g42390 |
| At5g09660 | ((At5g09660),(LOC_Os03g56280)) | LOC_Os03g56280 |
| At5g09760 | ((At5g09760),(LOC_Os01g65790)) | LOC_Os01g65790 |
| At5g09870 | ((At5g09870,At5g64740),(LOC_Os03g62090,LOC_Os07g14850)) | LOC_Os03g62090,LOC_Os07g14850 |
| At5g10100 | ((At5g10100),(LOC_Os03g22550)) | LOC_Os03g22550 |
| At5g10240 | ((At5g65010,At5g10240),(LOC_Os06g15420)) | LOC_Os06g15420 |
| At5g10330 | ((At5g10330,At1g71920),(LOC_Os08g25010)) | LOC_Os08g25010 |
| At5g10920 | ((At5g10920),(LOC_Os03g19280)) | LOC_Os03g19280 |
| At5g11160 | ((At5g11160),(LOC_Os03g59480,LOC_Os12g02510)) | LOC_Os03g59480,LOC_Os12g02510 |
| At5g11380 | ((At5g11380),(LOC_Os03g32526,LOC_Os04g34670,LOC_Os06g20500)) | LOC_Os03g32526,LOC_Os04g34670,LOC_Os06g20500 |
| At5g11880 | ((At5g11880),(LOC_Os02g24354)) | LOC_Os02g24354 |
| At5g12200 | ((At5g12200),(LOC_Os03g52770,LOC_Os03g61120,LOC_Os06g09230)) | LOC_Os03g52770,LOC_Os03g61120,LOC_Os06g09230 |
| At5g12890 | ((At5g12890),(LOC_Os04g20330)) | LOC_Os04g20330 |
| At5g13110 | ((At5g13110),(LOC_Os07g22350)) | LOC_Os07g22350 |
| At5g13280 | ((At5g13280),(LOC_Os01g70300,LOC_Os07g20544)) | LOC_Os01g70300,LOC_Os07g20544 |
| At5g13520 | ((At5g13520),(LOC_Os03g60460)) | LOC_Os03g60460 |
| At5g13630 | ((At5g13630),(LOC_Os03g20700)) | LOC_Os03g20700 |
| At5g13640 | ((At5g13640),(LOC_Os09g27210)) | LOC_Os09g27210 |
| At5g13710 | ((At5g13710),(LOC_Os05g51530,LOC_Os07g10600)) | LOC_Os05g51530,LOC_Os07g10600 |
| At5g13930 | ((At5g13930),(LOC_Os11g32650)) | LOC_Os11g32650 |
| At5g14220 | ((At5g14220),(LOC_Os02g34430,LOC_Os04g35080)) | LOC_Os02g34430,LOC_Os04g35080 |
| At5g14590 | ((At5g14590),(LOC_Os04g42920)) | LOC_Os04g42920 |

| | | |
|---|---|---|
| At5g14700 | ((At5g14700),(LOC_Os05g50250)) | LOC_Os05g50250 |
| At5g14760 | ((At5g14760),(LOC_Os02g04170)) | LOC_Os02g04170 |
| At5g14800 | ((At5g14800),(LOC_Os10g33690)) | LOC_Os10g33690 |
| At5g14850 | ((At5g14850),(LOC_Os01g39810,LOC_Os02g02110)) | LOC_Os01g39810,LOC_Os02g02110 |
| At5g15470 | ((At5g15470,At3g01040),(LOC_Os12g38930)) | LOC_Os12g38930 |
| At5g15490 | ((At5g39320,At5g15490,At3g29360),(LOC_Os03g55070,LOC_Os12g25690,LOC_Os12g25700)) | LOC_Os03g55070,LOC_Os12g25690,LOC_Os12g25700 |
| At5g16010 | ((At5g16010),(LOC_Os07g06800)) | LOC_Os07g06800 |
| At5g16570 | ((At5g16570),(LOC_Os02g50240)) | LOC_Os02g50240 |
| At5g16910 | ((At5g16910,At3g03050),(LOC_Os06g02180)) | LOC_Os06g02180 |
| At5g17030 | ((At5g17030,At5g17050),(LOC_Os04g42690)) | LOC_Os04g42690 |
| At5g17040 | ((At5g17040),(LOC_Os02g03970,LOC_Os02g45400,LOC_Os02g50770,LOC_Os03g45760,LOC_Os04g20474,LOC_Os06 [illegible] | LOC_Os02g03970,LOC_Os02g45400,LOC_Os02g50770,LOC_Os03g45760,LOC_Os04g20474,LO [illegible] |
| At5g17050 | ((At5g17030,At5g17050),(LOC_Os04g42690)) | LOC_Os04g42690 |
| At5g17230 | ((At5g17230),(LOC_Os06g51290)) | LOC_Os06g51290 |
| At5g17310 | ((At5g17310),(LOC_Os02g02560)) | LOC_Os02g02560 |
| At5g17330 | ((At5g17330),(LOC_Os03g51080)) | LOC_Os03g51080 |
| At5g17420 | ((At5g17420),(LOC_Os09g25490)) | LOC_Os09g25490 |
| At5g17920 | ((At5g17920,At3g03780),(LOC_Os12g42876,LOC_Os12g42884)) | LOC_Os12g42876,LOC_Os12g42884 |
| At5g18670 | ((At5g18670),(LOC_Os03g22790)) | LOC_Os03g22790 |
| At5g18800 | ((At3g06310,At5g18800),(LOC_Os03g01008,LOC_Os04g30740,LOC_Os04g44420)) | LOC_Os03g01008,LOC_Os04g30740,LOC_Os04g44420 |
| At5g18930 | ((At5g18930),(LOC_Os05g04990)) | LOC_Os05g04990 |
| At5g18990 | ((At1g23380,At3g24130,At5g18990),(LOC_Os01g19440)) | LOC_Os01g19440 |
| At5g19220 | ((At5g19220),(LOC_Os03g32620)) | LOC_Os03g32620 |
| At5g19530 | ((At5g19530,At1g70310),(LOC_Os01g58870,LOC_Os02g49770,LOC_Os05g41480)) | LOC_Os01g58870,LOC_Os02g49770,LOC_Os05g41480 |
| At5g19730 | ((At5g19730,At5g55590),(LOC_Os01g53990)) | LOC_Os01g53990 |
| At5g20040 | ((At5g20040),(LOC_Os03g42900,LOC_Os12g40560)) | LOC_Os03g42900,LOC_Os12g40560 |
| At5g20280 | ((At5g20280),(LOC_Os08g20660)) | LOC_Os08g20660 |
| At5g20830 | ((At5g20830,At3g43190),(LOC_Os03g28330,LOC_Os06g09450,LOC_Os07g42490,LOC_Os07g44970)) | LOC_Os03g28330,LOC_Os06g09450,LOC_Os07g42490,LOC_Os07g44970 |
| At5g21060 | ((At5g21060),(LOC_Os12g42870)) | LOC_Os12g42870 |

| | | |
|---|---|---|
| At5g22130 | ((At5g22130),(LOC_Os03g46750)) | LOC_Os03g46750 |
| At5g22800 | ((At5g22800),(LOC_Os06g13660)) | LOC_Os06g13660 |
| At5g23220 | ((At5g23220,At5g23230),(LOC_Os09g29930)) | LOC_Os09g29930 |
| At5g23230 | ((At5g23220,At5g23230),(LOC_Os09g29930)) | LOC_Os09g29930 |
| At5g23300 | ((At5g23300),(LOC_Os01g37910)) | LOC_Os01g37910 |
| At5g25900 | ((At5g25900),(LOC_Os06g37300)) | LOC_Os06g37300 |
| At5g26030 | ((At5g26030),(LOC_Os09g12560)) | LOC_Os09g12560 |
| At5g26710 | ((At5g49930,At5g26710),(LOC_Os10g22380)) | LOC_Os10g22380 |
| At5g26830 | ((At5g26830),(LOC_Os08g19850)) | LOC_Os08g19850 |
| At5g27450 | ((At5g27450),(LOC_Os10g18220)) | LOC_Os10g18220 |
| At5g27470 | ((At5g27470),(LOC_Os01g37837,LOC_Os03g10190)) | LOC_Os01g37837,LOC_Os03g10190 |
| At5g27870 | ((At3g05610,At5g27870),(LOC_Os03g18860,LOC_Os07g49100)) | LOC_Os03g18860,LOC_Os07g49100 |
| At5g35630 | ((At5g35630),(LOC_Os04g56400)) | LOC_Os04g56400 |
| At5g36880 | ((At5g36880),(LOC_Os01g27150,LOC_Os05g05700)) | LOC_Os01g27150,LOC_Os05g05700 |
| At5g37510 | ((At5g37510),(LOC_Os03g50540)) | LOC_Os03g50540 |
| At5g37690 | ((At5g37690),(LOC_Os06g49740)) | LOC_Os06g49740 |
| At5g37710 | ((At5g37710),(LOC_Os06g14490)) | LOC_Os06g14490 |
| At5g38410 | ((At5g38430,At5g38420,At5g38410,At1g67090),(LOC_Os10g35190)) | LOC_Os10g35190 |
| At5g38420 | ((At5g38430,At5g38420,At5g38410,At1g67090),(LOC_Os10g35190)) | LOC_Os10g35190 |
| At5g38430 | ((At5g38430,At5g38420,At5g38410,At1g67090),(LOC_Os10g35190)) | LOC_Os10g35190 |
| At5g38630 | ((At5g38630),(LOC_Os08g29520)) | LOC_Os08g29520 |
| At5g39320 | ((At5g39320,At5g15490,At3g29360),(LOC_Os03g55070,LOC_Os12g25690,LOC_Os12g25700)) | LOC_Os03g55070,LOC_Os12g25690,LOC_Os12g25700 |
| At5g40390 | ((At5g40390),(LOC_Os01g07530)) | LOC_Os01g07530 |
| At5g40760 | ((At5g40760,At3g27300),(LOC_Os02g38840)) | LOC_Os02g38840 |
| At5g40870 | ((At5g40870,At3g27190),(LOC_Os01g26039)) | LOC_Os01g26039 |
| At5g41480 | ((At5g41480),(LOC_Os03g01190)) | LOC_Os03g01190 |
| At5g41890 | ((At5g41890),(LOC_Os05g22970)) | LOC_Os05g22970 |
| At5g42650 | ((At5g42650,At3g18810,At1g49270),(LOC_Os03g55800)) | LOC_Os03g55800 |

| | | |
|---|---|---|
| At5g42800 | ((At5g42800),(LOC_Os06g45840)) | LOC_Os06g45840 |
| At5g42810 | ((At5g42810),(LOC_Os04g56580)) | LOC_Os04g56580 |
| At5g43280 | ((At5g13720,At5g43280),(LOC_Os05g31160,LOC_Os10g32300,LOC_Os10g32348)) | LOC_Os05g31160,LOC_Os10g32300,LOC_Os10g32348 |
| At5g43330 | ((At5g43330,At1g04410),(LOC_Os10g33800)) | LOC_Os10g33800 |
| At5g44030 | ((At5g44030),(LOC_Os10g32980)) | LOC_Os10g32980 |
| At5g44520 | ((At5g44520,At1g80840),(LOC_Os03g56869)) | LOC_Os03g56869 |
| At5g45300 | ((At5g45300),(LOC_Os02g03690)) | LOC_Os02g03690 |
| At5g45930 | ((At4g18480,At5g45930),(LOC_Os01g62070,LOC_Os04g36062)) | LOC_Os01g62070,LOC_Os04g36062 |
| At5g46180 | ((At5g46180),(LOC_Os07g01030)) | LOC_Os07g01030 |
| At5g47050 | ((At5g47050),(LOC_Os02g46220)) | LOC_Os02g46220 |
| At5g47500 | ((At5g47500,At2g17230),(LOC_Os08g34280)) | LOC_Os08g34280 |
| At5g47770 | ((At5g47770),(LOC_Os02g46080,LOC_Os04g49490)) | LOC_Os02g46080,LOC_Os04g49490 |
| At5g47810 | ((At5g47810),(LOC_Os01g65110,LOC_Os03g02030)) | LOC_Os01g65110,LOC_Os03g02030 |
| At5g47840 | ((At5g38660,At5g47840),(LOC_Os01g23680)) | LOC_Os01g23680 |
| At5g48140 | ((At5g48140,At3g07840,At3g07830,At3g07820),(LOC_Os10g37899)) | LOC_Os10g37899 |
| At5g48230 | ((At5g48230),(LOC_Os01g70550,LOC_Os12g08740)) | LOC_Os01g70550,LOC_Os12g08740 |
| At5g48300 | ((At5g48300),(LOC_Os08g25734)) | LOC_Os08g25734 |
| At5g48370 | ((At5g48370),(LOC_Os10g37340)) | LOC_Os10g37340 |
| At5g48840 | ((At5g48840),(LOC_Os01g04860,LOC_Os10g27450)) | LOC_Os01g04860,LOC_Os10g27450 |
| At5g48930 | ((At5g48930),(LOC_Os02g39850)) | LOC_Os02g39850 |
| At5g48960 | ((At5g48960),(LOC_Os01g14810,LOC_Os12g36100)) | LOC_Os01g14810,LOC_Os12g36100 |
| At5g49215 | ((At5g49215),(LOC_Os12g36810)) | LOC_Os12g36810 |
| At5g49460 | ((At5g49460,At3g06650),(LOC_Os01g19450)) | LOC_Os01g19450 |
| At5g49650 | ((At5g49650),(LOC_Os07g44660)) | LOC_Os07g44660 |
| At5g49720 | ((At5g49720),(LOC_Os03g52630)) | LOC_Os03g52630 |
| At5g50370 | ((At5g50370),(LOC_Os04g58580)) | LOC_Os04g58580 |
| At5g50375 | ((At5g50375),(LOC_Os06g04190)) | LOC_Os06g04190 |
| At5g50850 | ((At5g50850,At1g08250),(LOC_Os09g33500)) | LOC_Os09g33500 |

| | | |
|---|---|---|
| At5g51460 | ((At5g51460),(LOC_Os01g68380)) | LOC_Os01g68380 |
| At5g52560 | ((At5g52560),(LOC_Os06g48760,LOC_Os10g35960)) | LOC_Os06g48760,LOC_Os10g35960 |
| At5g52570 | ((At5g52570),(LOC_Os04g48880)) | LOC_Os04g48880 |
| At5g52920 | ((At5g52920),(LOC_Os03g38980,LOC_Os08g23430)) | LOC_Os03g38980,LOC_Os08g23430 |
| At5g53460 | ((At5g53460),(LOC_Os01g48960,LOC_Os05g48200)) | LOC_Os01g48960,LOC_Os05g48200 |
| At5g54080 | ((At5g54080),(LOC_Os06g51160)) | LOC_Os06g51160 |
| At5g54160 | ((At5g54160),(LOC_Os08g06100)) | LOC_Os08g06100 |
| At5g54810 | ((At5g54810,At4g27070,At4g13700,At4g11920),(LOC_Os08g04180)) | LOC_Os08g04180 |
| At5g55810 | ((At5g55810),(LOC_Os02g56980)) | LOC_Os02g56980 |
| At5g56350 | ((At5g56350,At4g21060,At1g78610),(LOC_Os01g16960)) | LOC_Os01g16960 |
| At5g56630 | ((At5g56630,At4g26270),(LOC_Os06g05860,LOC_Os07g12530)) | LOC_Os06g05860,LOC_Os07g12530 |
| At5g56760 | ((At5g56760),(LOC_Os04g56520,LOC_Os10g07574)) | LOC_Os04g56520,LOC_Os10g07574 |
| At5g57590 | ((At5g57590),(LOC_Os03g09090,LOC_Os07g06610)) | LOC_Os03g09090,LOC_Os07g06610 |
| At5g57655 | ((At5g57655),(LOC_Os03g11420)) | LOC_Os03g11420 |
| At5g57850 | ((At5g57850),(LOC_Os02g46120)) | LOC_Os02g46120 |
| At5g58330 | ((At5g58330),(LOC_Os05g48150)) | LOC_Os05g48150 |
| At5g58600 | ((At5g58600),(LOC_Os03g18110)) | LOC_Os03g18110 |
| At5g59440 | ((At5g59440),(LOC_Os05g18770)) | LOC_Os05g18770 |
| At5g59750 | ((At5g59750),(LOC_Os03g04000)) | LOC_Os03g04000 |
| At5g60540 | ((At5g60540),(LOC_Os12g05650)) | LOC_Os12g05650 |
| At5g60600 | ((At5g60600),(LOC_Os02g39160)) | LOC_Os02g39160 |
| At5g61540 | ((At4g00590,At5g61540),(LOC_Os04g58600)) | LOC_Os04g58600 |
| At5g61760 | ((At5g61760),(LOC_Os06g50060)) | LOC_Os06g50060 |
| At5g62530 | ((At5g62530),(LOC_Os10g21000)) | LOC_Os10g21000 |
| At5g62575 | ((At5g62575,At2g40170),(LOC_Os09g21470)) | LOC_Os09g21470 |
| At5g62790 | ((At5g62790),(LOC_Os01g01710,LOC_Os12g21870)) | LOC_Os01g01710,LOC_Os12g21870 |
| At5g62980 | ((At5g62980),(LOC_Os06g06100)) | LOC_Os06g06100 |
| At5g63310 | ((At5g63310),(LOC_Os03g17580,LOC_Os12g36194)) | LOC_Os03g17580,LOC_Os12g36194 |

| | | |
|---|---|---|
| At5g63380 | ((At5g63380),(LOC_Os01g48410)) | LOC_Os01g48410 |
| At5g63400 | ((At5g63400),(LOC_Os04g58580)) | LOC_Os04g58580 |
| At5g63510 | ((At5g63510),(LOC_Os02g30460)) | LOC_Os02g30460 |
| At5g63570 | ((At5g63570),(LOC_Os08g41990)) | LOC_Os08g41990 |
| At5g63680 | ((At5g63680,At5g08570),(LOC_Os04g58110,LOC_Os10g39900)) | LOC_Os04g58110,LOC_Os10g39900 |
| At5g63890 | ((At5g63890),(LOC_Os11g10750)) | LOC_Os11g10750 |
| At5g64050 | ((At5g64050),(LOC_Os02g02860)) | LOC_Os02g02860 |
| At5g64300 | ((At5g64300),(LOC_Os02g36340)) | LOC_Os02g36340 |
| At5g64370 | ((At5g64370),(LOC_Os07g30170)) | LOC_Os07g30170 |
| At5g64440 | ((At5g64440,At5g40200),(LOC_Os04g01250)) | LOC_Os04g01250 |
| At5g64740 | ((At5g09870,At5g64740),(LOC_Os03g62090,LOC_Os07g14850)) | LOC_Os03g62090,LOC_Os07g14850 |
| At5g65010 | ((At5g65010,At5g10240),(LOC_Os06g15420)) | LOC_Os06g15420 |
| At5g65110 | ((At5g65110),(LOC_Os11g39220)) | LOC_Os11g39220 |
| At5g65140 | ((At5g65140),(LOC_Os09g20390)) | LOC_Os09g20390 |
| At5g65690 | ((At4g37870,At5g65690),(LOC_Os03g15050)) | LOC_Os03g15050 |
| At5g65750 | ((At5g65750,At3g55410),(LOC_Os07g49520)) | LOC_Os07g49520 |
| At5g66760 | ((At5g66760),(LOC_Os07g04240)) | LOC_Os07g04240 |
| At5g67590 | ((At5g67590),(LOC_Os07g39710)) | LOC_Os07g39710 |
