# Supplementary material for: Genes encoding hub and bottleneck enzymes of the Arabidopsis metabolic network preferentially retain homeologs through whole genome duplication
Source: BMC Evol Biol. 2010 May 18;10:145. doi: 10.1186/1471-2148-10-145 (PMC2880986; doi:10.1186/1471-2148-10-145)
Supplement: Additional file 6 — Table S6. 173 Arabidopsis WGD-enzymes and their coding homeologs. [file 1471-2148-10-145-S6.PDF]

Table S6. 173 *Arabidopsis* WGD-enzymes and their coding-homeologs

Enzymes	WGD Paralogs			
	Gene 1	Gene 2	Blast E-value	Ks
1.1.1.205	At1g16350	At1g79470	4.10E-181	0.9595
1.1.1.22	At5g39320	At3g29360	1.30E-201	1.0318
1.1.1.271	At1g17890	At1g73250	2.20E-130	1.2645
1.1.1.3	At4g19710	At1g31230	0	2.0129
1.1.1.37	At3g15020	At1g53240	1.40E-128	0.7303
	At1g04410	At5g43330	6.50E-143	0.7474
1.1.1.44	At5g41670	At1g64190	4.00E-197	0.6629
1.1.1.49	At5g40760	At3g27300	3.70E-206	0.7087
1.11.1.11	At4g08390	At1g77490	2.10E-101	1.2699
1.13.99.1	At4g26260	At5g56640	1.30E-122	1.1671
1.14.11.-	At5g51810	At4g25420	1.70E-123	0.7524
1.14.11.12	At5g51810	At4g25420	1.70E-123	0.7524
1.14.11.13	At1g30040	At2g34550	2.80E-104	0.8878
1.14.11.15	At1g80330	At1g15550	5.80E-86	1.9759
1.14.13.-	At4g39950	At2g22330	4.50E-194	1.0186
1.14.13.79	At1g05160	At2g32440	2.00E-168	0.8296
1.14.17.4	At1g12010	At1g62380	4.50E-123	0.8339
1.14.19.2	At5g16230	At3g02610	6.20E-142	0.9326
1.14.99.-	At5g05580	At3g11170	3.30E-171	0.525
	At5g52570	At4g25700	5.50E-98	0.6214
1.2.1.12	At3g26650	At1g12900	4.00E-151	0.7609
	At1g79530	At1g16300	6.00E-154	0.5745
1.2.1.13	At1g12900	At3g26650	4.00E-151	0.7609

1.2.1.25	At3g17240	At1g48030	5.80E-198	1.0029
1.2.1.41	At2g39800	At3g55610	0	0.568
1.2.1.44	At4g30470	At2g23910	2.50E-113	0.7068
	At1g80820	At1g15950	6.50E-116	1.1273
1.2.4.1	At1g30120	At2g34590	1.80E-169	0.7786
1.2.4.4	At3g13450	At1g55510	9.10E-151	0.6444
1.3.1.26	At3g59890	At2g44040	2.40E-111	0.6324
1.3.5.1	At3g27380	At5g40650	1.50E-124	0.7568
1.4.1.2	At3g03910	At5g18170	4.40E-174	0.7176
1.4.4.2	At2g35370	At1g32470	6.20E-59	0.5979
	At4g33010	At2g26080	0	0.7756
1.5.1.20	At3g59970	At2g44160	0	0.6778
1.5.1.3	At4g34570	At2g16370	2.30E-207	0.7805
1.5.99.12	At2g19500	At4g29740	1.60E-149	0.8848
	At2g19500	At5g56970	5.60E-104	2.0866
	At4g29740	At5g56970	7.30E-111	2.0293
1.6.5.3	At1g79010	At1g16700	5.80E-92	0.3639
	At3g06310	At5g18800	2.60E-47	0.9046
	At5g63510	At3g48680	4.50E-109	0.636
1.8.1.4	At3g17240	At1g48030	5.80E-198	1.0029
1.8.1.9	At2g17420	At4g35460	1.10E-129	0.9762
1.8.5.1	At1g19570	At1g75270	1.60E-75	0.8792
2.1.1.103	At1g48600	At3g18000	2.60E-111	0.6796
2.1.1.104	At1g24735	At1g67980	9.10E-66	1.5156
2.1.1.14	At3g03780	At5g17920	0	0.5286
2.1.1.143	At1g76090	At1g20330	7.00E-146	1.3791

2.1.1.41	At1g20330	At1g76090	7.00E-146	1.3791
2.1.1.45	At2g16370	At4g34570	2.30E-207	0.7805
2.1.2.1	At1g22020	At1g36370	4.40E-185	2.272
2.1.2.11	At3g61530	At2g46110	1.60E-139	1.0637
2.2.1.1	At2g45290	At3g60750	0	0.6596
2.2.1.7	At3g21500	At4g15560	0	1.0265
2.3.1.-	At1g25450	At1g68530	2.40E-210	1.2744
2.3.1.1	At4g37670	At2g22910	4.00E-164	0.6779
2.3.1.12	At3g13930	At1g54220	1.60E-164	0.5612
2.3.1.15	At1g01610	At4g00400	1.20E-125	1.0166
2.3.1.16	At2g33150	At1g04710	2.40E-160	0.7163
2.3.1.30	At3g13110	At1g55920	4.40E-105	1.2384
	At4g35640	At2g17640	7.90E-86	0.7212
2.3.1.50	At5g23670	At3g48780	1.30E-206	0.5662
2.3.1.51	At3g11430	At5g06090	8.80E-190	0.9402
2.3.1.9	At2g33150	At1g04710	2.40E-160	0.7163
2.3.3.1	At3g58750	At2g42790	2.30E-195	0.7133
2.3.3.13	At1g18500	At1g74040	0	0.694
2.3.3.8	At3g06650	At5g49460	0	0.5607
	At1g60810	At1g10670	9.60E-188	0.482
2.4.1.-	At2g29750	At1g07240	7.80E-119	2.1681
	At5g66690	At3g50740	1.20E-141	0.9932
	At4g36770	At3g50740	1.20E-76	7.9967
	At3g53150	At2g36760	2.10E-92	3.8793
	At3g11340	At5g05870	6.20E-92	2.0239
2.4.1.111	At5g66690	At3g50740	1.20E-141	0.9932
2.4.1.12	At5g16910	At3g03050	0	1.019

	At5g09870	At5g64740	0	0.7728
	At4g39350	At2g21770	0	0.8085
	At4g31590	At2g24630	0	0.6254
	At2g25540	At4g32410	0	0.7095
2.4.1.120	At4g15480	At3g21560	2.50E-126	1.3786
2.4.1.123	At1g09350	At1g56600	5.30E-126	0.8671
2.4.1.13	At5g20830	At3g43190	0	0.9527
2.4.1.15	At1g16980	At1g78580	0	0.8378
2.4.1.203	At2g29750	At1g07240	7.80E-119	2.1681
	At5g66690	At3g50740	1.20E-141	0.9932
	At5g05870	At3g11340	6.20E-92	2.0239
	At4g36770	At3g50740	1.20E-76	7.9967
	At3g53150	At2g36760	2.10E-92	3.8793
2.4.1.215	At2g29750	At1g07240	7.80E-119	2.1681
	At5g66690	At3g50740	1.20E-141	0.9932
	At5g05870	At3g11340	6.20E-92	2.0239
	At4g36770	At3g50740	1.20E-76	7.9967
	At3g53150	At2g36760	2.10E-92	3.8793
2.4.1.43	At3g28340	At1g70090	2.40E-101	3.5807
	At1g70090	At1g24170	1.10E-145	0.8725
	At4g02130	At1g02720	3.40E-127	0.9029
	At5g15470	At3g01040	1.90E-211	0.5917
2.4.2.11	At4g36940	At2g23420	5.40E-181	0.9327
2.4.2.14	At4g38880	At2g16570	1.40E-152	5.4331
	At4g34740	At2g16570	7.30E-197	0.8811
2.4.2.7	At4g22570	At4g12440	4.00E-74	0.8889
2.4.2.9	At5g40870	At3g27190	2.90E-191	0.6135
2.5.1.-	At1g17050	At1g78510	2.50E-116	0.8694
2.5.1.1	At1g78510	At1g17050	2.50E-116	0.8694
	At4g17190	At5g47770	4.60E-143	0.5598
2.5.1.10	At4g17190	At5g47770	4.60E-143	0.5598
2.5.1.16	At1g23820	At1g70310	8.50E-136	0.6567

2.5.1.27	At1g25410	At1g68460	1.20E-67	2.2479
2.5.1.29	At4g36810	At2g23800	1.20E-101	1.9695
2.5.1.47	At5g28020	At3g04940	2.90E-105	0.7629
	At4g14880	At3g22460	8.80E-117	0.7137
	At3g59760	At2g43750	1.50E-118	0.6141
2.5.1.55	At1g16340	At1g79500	7.20E-126	0.6739
2.5.1.6	At1g02500	At4g01850	1.10E-174	0.6588
2.6.1.-	At3g19710	At1g50090	1.30E-98	1.3931
2.6.1.2	At1g72330	At1g17290	3.50E-191	0.6557
	At1g23310	At1g70580	1.30E-208	0.6325
2.6.1.4	At1g23310	At1g70580	1.30E-208	0.6325
2.6.1.42	At3g49680	At5g65780	1.10E-141	0.8224
2.6.1.52	At4g35630	At2g17630	4.30E-160	0.7887
2.7.1.-	At5g61760	At5g07370	9.60E-97	0.8771
2.7.1.1	At3g20040	At1g50460	4.00E-153	0.819
	At4g29130	At2g19860	7.00E-172	0.8761
2.7.1.11	At5g56630	At4g29220	7.80E-159	1.6135
	At5g56630	At4g26270	1.80E-158	0.8515
2.7.1.140	At5g61760	At5g07370	9.60E-97	0.8771
2.7.1.151	At5g61760	At5g07370	9.60E-97	0.8771
2.7.1.19	At2g47400	At3g62410	9.20E-33	1.5075
2.7.1.2	At2g19860	At4g29130	7.00E-172	0.8761
2.7.1.32	At4g09760	At1g71697	4.90E-99	2.15
2.7.1.33	At2g17320	At4g35360	1.30E-74	0.575
2.7.1.4	At2g19860	At4g29130	7.00E-172	0.8761
2.7.1.40	At5g63680	At5g08570	5.80E-202	0.8107

	At5g56350	At4g26390	1.90E-200	1.1692
	At3g52990	At2g36580	8.10E-189	0.5429
2.7.1.48	At3g27190	At5g40870	2.90E-191	0.6135
2.7.1.6	At5g14470	At3g01640	9.30E-105	0.8366
2.7.1.71	At4g39540	At2g21940	2.10E-63	1.1259
2.7.1.90	At1g76550	At1g20950	0	0.6196
2.7.2.11	At2g39800	At3g55610	0	0.568
2.7.2.3	At3g12780	At1g56190	2.80E-174	0.4821
2.7.2.4	At5g14060	At3g02020	3.90E-192	0.5687
	At4g19710	At1g31230	0	2.0129
2.7.4.6	At4g23900	At4g11010	2.30E-85	0.7046
2.7.4.8	At3g57550	At2g41880	1.30E-106	0.7849
2.7.6.1	At2g35390	At1g32380	2.20E-148	0.5511
2.7.7.10	At3g03250	At5g17310	2.00E-185	0.6063
2.7.7.13	At2g39770	At2g55590		
	At1g09940	At1g58290	3.70E-161	0.8376
2.7.7.27	At4g39210	At2g21590	4.90E-206	0.6501
2.7.7.41	At1g62430	At4g22340	1.50E-155	1.617
	At3g60620	At2g45150	7.50E-109	0.8669
2.7.7.9	At3g03250	At5g17310	2.00E-185	0.6063
2.7.8.15	At3g57220	At2g41490	1.10E-142	0.6339
2.7.8.5	At3g55030	At2g39290	2.50E-76	0.6718
2.8.2.-	At1g18590	At1g74090	6.20E-128	1.1313
3.1.1.11	At5g64640	At5g09760	5.40E-178	0.9373
	At5g49180	At3g06830	4.30E-174	1.2822
	At5g27870	At3g05610	4.20E-207	1.3014
	At5g04970	At3g10720	4.00E-212	0.9367
	At4g33230	At2g26440	3.60E-72	54.1279

	At4g02330	At1g02810	9.20E-197	1.0396
	At4g00190	At2g45220	1.30E-100	4.4891
	At3g62170	At2g47040	1.00E-178	1.2571
	At3g60730	At2g45220	1.70E-79	50.4135
	At3g59010	At2g43050	5.00E-141	0.8989
	At3g14300	At1g53830	6.70E-78	3.2485
3.1.1.3	At2g31690	At1g05800	8.10E-127	0.966
	At2g30550	At1g06800	4.90E-166	1.1227
	At2g31100	At1g06250	3.00E-84	1.1243
	At1g73920	At1g18460	1.80E-157	0.8836
3.1.1.31	At3g49360	At5924410		
	At1g72520	At1g17420	0	0.9386
3.1.1.4	At4g29460	At2g19690	4.20E-36	0.7292
3.1.2.14	At3g25110	At4g13050	3.40E-127	0.9449
3.1.2.4	At3g24360	At4g13360	1.70E-139	0.513
3.1.2.6	At2g31350	At1g06130	2.80E-105	0.4966
3.1.3.12	At1g23870	At1g70290	0	1.1485
	At5g65140	At5g10100	2.50E-103	0.8447
	At5g65140	At2g22190	1.30E-87	2.5808
	At4g39770	At2g22190	2.20E-84	0.9287
	At4g12430	At4g22590	1.90E-136	0.7692
	At1g78090	At1g22210	1.10E-90	0.9491
	At1g35910	At1g22210	5.90E-82	2.4681
3.1.3.4	At2g01180	At1g15080	7.90E-84	5.1237
3.1.4.4	At1g52570	At3g15730	0	1.2274
3.2.1.15	At5g48140	At3g07850	2.70E-79	2.3299
	At4g01890	At1g02460	2.20E-135	0.9915
	At3g59850	At2g43860	6.30E-100	3.4686
	At3g57510	At2g41850	2.90E-119	0.9167
	At1g70500	At1g23460	5.30E-146	0.7261
	At1g70370	At1g23760	9.50E-197	1.3807
	At1g60590	At1g10640	2.00E-162	1.0677
3.2.1.2	At4g00490	At2g45880	1.20E-142	3.034
3.2.1.26	At4g09510	At1g72000	4.80E-197	2.6718
	At4g09510	At1g35580	0	1.3322

	At1g72000	At1g22650	3.90E-203	1.4968
	At1g35580	At1g22650	3.30E-202	10.0457
3.2.1.4	At1g23210	At1g70710	1.10E-194	0.8374
3.2.2.16	At4g34840	At4g38800	7.70E-61	3.31
3.3.1.1	At4g13940	At3g23810	1.50E-204	0.9635
3.5.4.5	At4g29640	At2g19570	1.60E-57	1.085
3.5.5.1	At3g44310	At5g22300	2.70E-109	0.8269
4.1.1.-	At1g67090	At5g38410	2.50E-78	0.3727
4.1.1.15	At1g65960	At2g02000	1.70E-178	4.3088
4.1.1.18	At3g02470	At5g15950	6.00E-129	1.1636
4.1.1.19	At2g16500	At4g34710	0	0.8813
4.1.1.25	At4g28680	At2g20340	2.10E-154	1.5775
4.1.1.31	At1g53310	At3g14940	0	0.7203
4.1.1.33	At2g38700	At3g54250	1.30E-171	0.5564
4.1.1.35	At3g62830	At2g47650	1.30E-184	0.8186
	At2g28760	At3g46440	5.70E-138	1.6555
	At5g59290	At3g46440	4.90E-141	0.7641
	At2g27860	At1g08200	1.20E-171	0.6796
4.1.1.36	At1g48610	At3g18030	2.90E-85	39.9791
4.1.1.39	At5g38410	At1g67090	2.50E-78	0.3727
4.1.1.50	At3g02470	At5g15950	6.00E-129	1.1636
4.1.1.65	At5g57190	At4g25970	1.00E-214	0.6963
4.1.2.13	At2g21330	At4g38970	3.10E-140	0.8428
	At2g36460	At3g52930	1.40E-155	1.0208
4.1.3.1	At1g21440	At1g77060	1.50E-113	0.8643
4.1.3.27	At5g05730	At3g55870	5.20E-173	3.5873

4.2.1.20	At5g54810	At4g27070	3.70E-188	0.6615
4.2.1.46	At3g62830	At2g47650	1.30E-184	0.8186
4.2.1.47	At3g51160	At5g66280	8.50E-156	1.0559
4.2.1.52	At3g60880	At2g45440	4.20E-146	1.0267
4.2.1.65	At5g22300	At3g44310	2.70E-109	0.8269
4.2.1.84	At3g44310	At5g22300	2.70E-109	0.8269
4.2.1.91	At1g08250	At2g27820	1.20E-137	1.4919
	At3g44720	At5g22630	8.40E-148	1.4503
4.4.1.14	At3g61510	At1g01480	1.60E-139	2.4404
	At2g22810	At4g37770	1.00E-171	0.9865
	At5g65800	At4g37770	5.90E-162	2.331
	At5g65800	At3g49700	1.10E-198	0.8717
	At4g37770	At3g49700	4.00E-167	1.9911
4.4.1.5	At1g80160	At1g15380	7.80E-66	0.9428
5.1.3.2	At1g64440	At4g23920	1.80E-124	1.6957
	At1g63180	At1g12780	4.20E-143	0.7989
	At4g23920	At4g10960	2.60E-131	0.7993
5.1.3.5	At2g34850	At1g30620	2.90E-149	0.8076
5.1.3.6	At4g00110	At1g02000	7.70E-182	1.3353
5.3.3.2	At5g16440	At3g02780	3.40E-108	0.5455
5.3.99.6	At3g25780	At1g13280	6.80E-88	0.8118
5.4.2.2	At1g70730	At1g23190	0	0.5601
5.4.3.8	At5g63570	At3g48730	2.00E-181	0.988
5.4.4.2	At1g74710	At1g18870	5.60E-172	0.954
5.4.99.5	At3g29200	At1g69370	1.20E-71	2.1515
6.2.1.12	At3g21230	At1g51680	5.40E-149	2.3695
6.2.1.5	At5g23250	At5g08300	2.10E-134	0.6615

6.3.1.2	At5g37600	At1g66200	5.20E-149	0.5806
	At3g17820	At1g48470	1.10E-137	0.8253
6.3.2.17	At5g05980	At3g55630	2.20E-112	1.6897
6.3.4.2	At4g20320	At1g30820	9.70E-180	2.0286
	At2g34890	At1g30820	7.90E-190	0.5925
6.3.5.4	At5g10240	At5g65010	0	0.5891
6.6.1.1	At4g18480	At5g45930	9.80E-146	1.0676
	At1g20330	At1g76090	7.00E-146	1.3791
