# Supplementary material for: Genes encoding hub and bottleneck enzymes of the Arabidopsis metabolic network preferentially retain homeologs through whole genome duplication
Source: BMC Evol Biol. 2010 May 18;10:145. doi: 10.1186/1471-2148-10-145 (PMC2880986; doi:10.1186/1471-2148-10-145)
Supplement: Additional file 7 — Table S7. 25 Arabidopsis tandem-enzymes and their coding tandem homeologs in the Arabidopsis metabolic network. [file 1471-2148-10-145-S7.PDF]

Table S7. 25 tandem-enzymes and their coding-homeologs of the *Arabidopsis* metabolic network

Enzymes	Tandem paralogs		
	Gene 1	Gene 2	Blast-evalue
1.1.1.195	At1g09510	At1g09500	3.50E-125
	At1g09500	At1g09490	2.50E-124
1.1.1.44	At1g71180	At1g71170	2.60E-136
1.10.3.3	At5g21100	At5g21105	8.60E-241
1.11.1.6	At1g20620	At1g20630	4.30E-223
1.14.99.7	At5g24140	At5g24150	5.00E-181
	At5g24150	At5g24160	
1.3.1.-	At2g34490	At2g34500	1.70E-226
1.3.1.42	At1g76690	At1g76680	5.40E-186
1.8.5.1	At1g19570	At1g19550	2.60E-71
2.1.2.1	At4g13890	At4g13930	1.60E-216
2.4.1.115	At5g17040	At5g17050	6.50E-172
2.5.1.21	At4g34650	At4g34640	1.70E-171
3.1.1.31	At5g24420	At5g24410	5.90E-91
3.2.1.147	At5g25980	At5g26000	1.10E-217
3.2.1.22	At5g08380	At5g08370	7.50E-139
3.4.19.9	At1g78670	At1g78680	6.00E-130
3.5.1.-	At5g56660	At5g56650	9.20E-205
4.2.3.14	At3g25810	At3g25820	1.10E-258
4.2.3.15	At4g16730	At4g16740	5.80E-164
	At3g25810	At3g25820	1.10E-258
4.2.3.16	At3g25810	At3g25820	1.10E-258
4.4.1.8	At4g23590	At4g23600	7.10E-182
4.99.1.6	At2g30750	At2g30770	8.10E-245
5.3.99.6	At3g25770	At3g25780	1.10E-100

	At3g25770	At3g25760	1.50E-119
5.4.99.-	At1g78960	At1g78955	0.00E+00
6.1.1.14	At1g29870	At1g29880	1.80E-160
6.4.1.2	At1g36160	At1g36180	0.00E+00
