# Supplementary material for: Genes encoding hub and bottleneck enzymes of the Arabidopsis metabolic network preferentially retain homeologs through whole genome duplication
Source: BMC Evol Biol. 2010 May 18;10:145. doi: 10.1186/1471-2148-10-145 (PMC2880986; doi:10.1186/1471-2148-10-145)

Figure S1. Comparison of three topological centralities between enzymes retaining tandem paralogs and the other enz ymes. The *p* values were calculated using Manny-Whitney U test, n1 =453 and n2=25 were used.

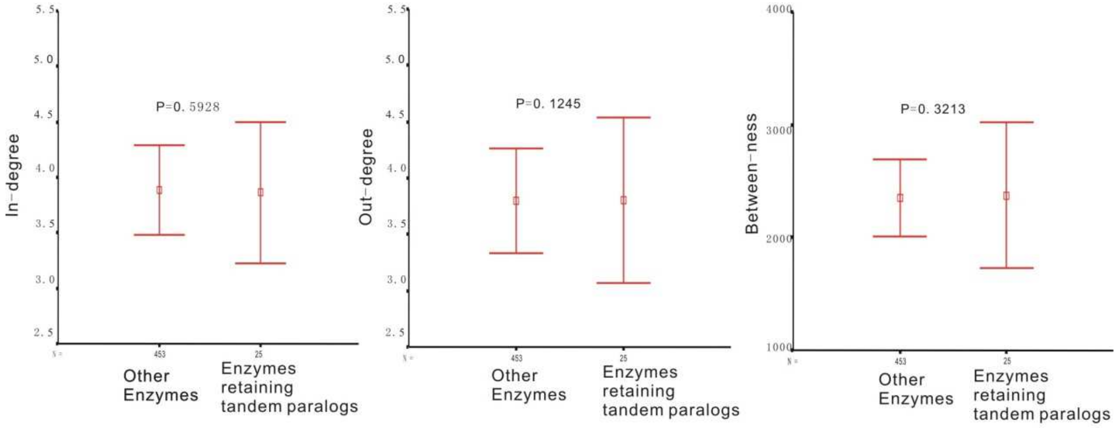

Supplement: Additional file 8 — Figure S1. Comparison of three topological centralities between enzymes retaining tandem-homeologs and the other enzymes. [file 1471-2148-10-145-S8.PDF]
