# Supplementary material for: Genes encoding hub and bottleneck enzymes of the Arabidopsis metabolic network preferentially retain homeologs through whole genome duplication
Source: BMC Evol Biol. 2010 May 18;10:145. doi: 10.1186/1471-2148-10-145 (PMC2880986; doi:10.1186/1471-2148-10-145)
Supplement: Additional file 9 — Table S8. 226 Populus WGD-enzymes and their coding homeologs. [file 1471-2148-10-145-S9.PDF]

Table S8. 226 *Populus* WGD-enzymes and their coding-homeologs

Enzymes	WGD Paralogs			
	Gene 1	Gene 2	Blast E-value	Ks
1.1.1.-	estExt_fgenesh4_pg.C_LG_X0521	fgenesh4_pg.C_LG_VIII001608	3.01	0.94
	gw1.II.2815.1	gw1.XIV.3153.1	0.07	0.3
	gw1.V.3913.1	gw1.VII.3047.1	0.04	0.17
1.1.1.1	estExt_fgenesh4_pg.C_LG_VII0344	eugene3.00050003	2.83	2.15
	estExt_fgenesh4_pm.C_LG_VII0126	eugene3.00050005	2.76	2.13
	gw1.I.5494.1	gw1.XI.942.1	0.05	0.33
1.1.1.100	estExt_Genewise1_v1.C_LG_XIII2039	grail3.0065002601	0.17	0.81
	eugene3.00060925	eugene3.00161238	0.04	0.26
	eugene3.00061411	eugene3.00160678	0.11	0.83
	eugene3.00140927	fgenesh4_pg.C_LG_II002373	0.09	0.28
1.1.1.122	estExt_fgenesh4_pg.C_LG_I2703	estExt_fgenesh4_pm.C_LG_IX0390	2.46	0.97
1.1.1.2	fgenesh4_pg.C_LG_VIII001733	gw1.VIII.78.1	0.67	0.75
	grail3.0006011301	grail3.0009001001	0.02	0.25
1.1.1.205	fgenesh4_pg.C_LG_VIII000715	fgenesh4_pg.C_LG_X001563	0.04	0.28
	fgenesh4_pm.C_LG_XII000372	fgenesh4_pm.C_LG_XV000301	0.08	0.25
1.1.1.21	estExt_Genewise1_v1.C_LG_VI1242	estExt_Genewise1_v1.C_LG_XVI3242	0.19	1.41
1.1.1.219	estExt_fgenesh4_pg.C_LG_IX1058	gw1.IX.3396.1	0.23	0.99
	estExt_fgenesh4_pg.C_LG_X1136	fgenesh4_pg.C_LG_VIII001076	2.49	1.08
	estExt_fgenesh4_pg.C_LG_XVIII0872	estExt_fgenesh4_pm.C_LG_VI0550	0.07	0.28
	estExt_fgenesh4_pm.C_LG_I0317	estExt_Genewise1_v1.C_LG_III0708	2.15	1.46
	estExt_fgenesh4_pm.C_LG_XI0107	gw1.XIII.2712.1	0.66	1.82
	gw1.II.2125.1	gw1.V.2288.1	1.77	0.98
1.1.1.22	eugene3.00041110	eugene3.00101501	2.3	1.25
1.1.1.34	estExt_fgenesh4_pg.C_LG_IX0010	estExt_fgenesh4_pm.C_LG_II0017	2.33	1.34
	estExt_fgenesh4_pm.C_LG_XI0390	fgenesh4_pm.C_LG_I001255	0.06	0.29
1.1.1.37	estExt_Genewise1_v1.C_LG_I4975	fgenesh4_pg.C_LG_IV000431	0.13	1.35
1.1.1.39	estExt_Genewise1_v1.C_LG_XIV1110	eugene3.00021450	2.38	0.89
1.1.1.40	estExt_fgenesh4_pg.C_LG_XVIII0238	eugene3.00061692	0.06	0.21
	estExt_Genewise1_v1.C_LG_III0888	gw1.I.387.1	0.03	0.19
1.1.1.41	estExt_fgenesh4_pg.C_LG_VII0812	eugene3.00050378	4.51	0.98
1.1.1.42	fgenesh4_pg.C_LG_X001588	fgenesh4_pm.C_LG_VIII000318	0.01	0.22
1.1.1.44	estExt_fgenesh4_pm.C_LG_IV0340	gw1.X.458.1	1.88	1.16
	fgenesh4_pm.C_LG_III000453	grail3.0008041101	0.02	0.29

1.1.1.51	fgenesh4_pm.C_LG_VII000217	gw1.V.2994.1	0.09	0.31
1.1.1.95	gw1.I.3925.1	gw1.II.1474.1	0.08	1.27
	gw1.VIII.1454.1	gw1.X.3201.1	0.02	0.3
1.1.3.6	estExt_fgenesh4_pm.C_LG_VIII0711	gw1.II.345.1	2.54	1.37
1.1.99.1	eugene3.00101594	fgenesh4_pg.C_LG_VIII000761	0.2	0.66
1.10.3.3	estExt_fgenesh4_pg.C_LG_III0903	eugene3.00121252	1.38	0.69
	estExt_fgenesh4_pg.C_LG_VI0684	fgenesh4_pg.C_LG_IX001228	0.19	1.19
	estExt_fgenesh4_pg.C_LG_X1635	estExt_fgenesh4_pm.C_LG_VIII0291	0.08	0.42
	estExt_Genewise1_v1.C_LG_II2639	eugene3.00051523	2.58	1.54
	eugene3.00002084	fgenesh4_pg.C_LG_XIV000947	0.03	0.21
	eugene3.00010005	eugene3.00031796	0.02	0.25
1.11.1.11	estExt_fgenesh4_pm.C_LG_IV0530	eugene3.00090344	1.78	1.61
	fgenesh4_pm.C_LG_II000371	gw1.V.5347.1	0.06	0.17
1.11.1.7	estExt_fgenesh4_pg.C_LG_I0096	fgenesh4_pg.C_LG_XVI000455	0.31	1.31
	estExt_fgenesh4_pg.C_LG_III1871	estExt_fgenesh4_pm.C_LG_I0036	2.36	1.26
	estExt_fgenesh4_pg.C_LG_VIII0975	gw1.X.450.1	0.06	0.26
	estExt_fgenesh4_pg.C_LG_XV0039	eugene3.00120328	0.12	0.35
	estExt_fgenesh4_pg.C_LG_XVI1240	fgenesh4_pg.C_LG_XIII000524	2.6	1.7
1.13.11.12	estExt_Genewise1_v1.C_LG_VII3718	gw1.II.2473.1	3.27	1.38
	estExt_fgenesh4_pm.C_LG_X0170	gw1.VIII.1781.1	0.04	0.24
1.13.11.27	eugene3.00051120	fgenesh4_pm.C_LG_II000277	0.05	0.28
1.14.-.-	estExt_fgenesh4_pg.C_LG_II0572	estExt_fgenesh4_pm.C_LG_V0449	1.94	1.29
	estExt_fgenesh4_pg.C_LG_III1229	estExt_Genewise1_v1.C_LG_I8344	1.95	1.56
	estExt_fgenesh4_pg.C_LG_IX1223	fgenesh4_pm.C_LG_I000672	0.04	0.31
	estExt_fgenesh4_pg.C_LG_XII0887	fgenesh4_pg.C_LG_XV000621	0.06	0.34
	estExt_fgenesh4_pg.C_LG_XIV0443	gw1.II.1429.1	0.05	0.24
	estExt_fgenesh4_pg.C_LG_XVII0012	gw1.VII.501.1	0.05	0.26
1.14.11.-	eugene3.00020365	fgenesh4_pg.C_LG_V001376	0.21	0.68
	eugene3.00111265	gw1.I.5592.1	0.29	0.92
	eugene3.00111269	gw1.I.5585.1	0.27	0.52
	fgenesh4_pg.C_LG_I003186	gw1.XI.865.1	0.33	0.54
1.14.11.12	estExt_fgenesh4_pg.C_LG_XV1053	fgenesh4_pg.C_LG_XII001220	3.04	1.04
	estExt_fgenesh4_pm.C_LG_V0022	estExt_fgenesh4_pm.C_LG_V0384	1.96	1.23
	eugene3.00031009	fgenesh4_pm.C_LG_II000736	0.18	1.63
	fgenesh4_pg.C_LG_VI001745	fgenesh4_pg.C_LG_XVIII000349	0.05	0.28
	fgenesh4_pm.C_LG_II000702	fgenesh4_pm.C_LG_XIV000079	0.03	0.22
	gw1.IV.1861.1	gw1.XI.1843.1	0.07	0.35
1.14.11.13	estExt_fgenesh4_pg.C_LG_XI0670	eugene3.00012757	2.85	1.54
	estExt_Genewise1_v1.C_LG_X0681	fgenesh4_pg.C_LG_VIII000899	0.07	0.28
	gw1.II.529.1	gw1.XIV.2239.1	0.08	0.43

1.14.11.15	estExt_fgenesh4_pg.C_LG_I3206	eugene3.00111228	0.08	0.19
	eugene3.00011087	eugene3.00180373	0.4	2.25
	fgenesh4_pg.C_LG_III000353	gw1.VI.2741.1	0.4	2.82
1.14.11.9	eugene3.00002206	eugene3.00040720	0.24	0.56
	eugene3.00051288	fgenesh4_pg.C_LG_II000379	0.15	0.65
1.14.13.11	estExt_fgenesh4_pg.C_LG_XIII0519	grail3.0094002901	1.8	1.31
1.14.13.21	eugene3.00011827	fgenesh4_pm.C_LG_IX000451	0.06	0.45
1.14.14.1	eugene3.00031308	fgenesh4_pg.C_LG_I000592	2.27	1.37
1.14.19.-	eugene3.00140370	grail3.0039012501	2.92	1.57
1.14.99.-	estExt_fgenesh4_pg.C_LG_VIII0590	estExt_fgenesh4_pm.C_LG_VI0352	2.26	0.91
	fgenesh4_pm.C_LG_VI000532	gw1.XVIII.3250.1	0.14	0.25
1.14.99.7	estExt_fgenesh4_pm.C_LG_V0246	gw1.II.3308.1	0.03	0.22
1.17.4.1	estExt_fgenesh4_pg.C_LG_VII0648	eugene3.00051017	0.1	1.13
1.2.1.-	estExt_Genewise1_v1.C_LG_II0707	gw1.IX.2784.1	0.18	1.08
1.2.1.11	eugene3.00081272	eugene3.00100987	0.02	0.27
1.2.1.12	estExt_fgenesh4_pg.C_LG_X0484	estExt_Genewise1_v1.C_LG_XII1463	2.67	1.03
	estExt_fgenesh4_pm.C_LG_V0707	gw1.II.3569.1	0.01	0.23
	estExt_fgenesh4_pm.C_LG_VIII0332	gw1.X.1226.1	0.02	0.17
	estExt_Genewise1_v1.C_LG_I2848	eugene3.00150702	2.54	1.04
1.2.1.27	gw1.I.2279.1	gw1.IX.2840.1	0.02	0.25
1.2.1.5	estExt_fgenesh4_pm.C_LG_V0365	fgenesh4_pg.C_LG_II000742	2.31	1.57
1.2.4.1	estExt_fgenesh4_pm.C_LG_I0194	gw1.III.1461.1	0.01	0.15
1.2.4.2	estExt_fgenesh4_pg.C_LG_VIII0421	estExt_Genewise1_v1.C_LG_X2288	0.07	0.2
1.2.4.4	eugene3.00050925	gw1.V.2491.1	0.15	1.49
1.3.1.-	estExt_fgenesh4_pg.C_LG_X1518	fgenesh4_pg.C_LG_VIII000736	2.37	1.34
	estExt_fgenesh4_pm.C_LG_II0164	estExt_Genewise1_v1.C_LG_V1376	3.14	1.12
	eugene3.00090496	gw1.IV.588.1	3.67	0.88
	grail3.0006037601	grail3.0010045601	3.02	1.57
1.3.1.9	eugene3.00031909	eugene3.00160402	0.23	1.15
1.3.3.1	estExt_fgenesh4_pg.C_LG_V0296	gw1.VII.2403.1	0.02	0.22
1.3.3.3	gw1.XII.1260.1	gw1.XV.1956.1	1.06	0.8
1.3.3.4	fgenesh4_pg.C_LG_II001916	fgenesh4_pg.C_LG_XIV000523	0.03	0.2

1.3.3.6	estExt_fgenesh4_pm.C_LG_V0077	estExt_fgenesh4_pm.C_LG_VII0199	0.04	0.24
1.3.99.-	eugene3.00002400	gw1.XIV.3183.1	0.03	0.2
1.3.99.5	estExt_fgenesh4_pg.C_LG_X1849	estExt_Genewise1_v1.C_LG_VIII1722	3.65	1.34
	estExt_fgenesh4_pg.C_LG_X2215	estExt_fgenesh4_pm.C_LG_VIII0046	3.07	0.95
	eugene3.00090424	fgenesh4_pg.C_LG_IV001381	0.06	0.19
1.3.99.7	estExt_Genewise1_v1.C_LG_VI1706	estExt_Genewise1_v1.C_LG_XVI3692	2.21	1.18
1.4.1.3	estExt_fgenesh4_pg.C_LG_XIX0409	eugene3.00130573	0.2	0.38
1.4.3.6	estExt_fgenesh4_pg.C_LG_XV0575	eugene3.00120775	0.02	0.21
1.4.7.1	estExt_fgenesh4_pg.C_LG_VI0304	estExt_fgenesh4_pg.C_LG_XVI0276	0.04	0.22
1.8.1.4	gw1.VIII.2248.1	gw1.X.574.1	0.03	0.22
1.8.7.1	gw1.I.1513.1	gw1.IX.3567.1	0.01	0.25
2.1.1.10	eugene3.00051204	fgenesh4_pg.C_LG_II000468	2.64	1.87
	fgenesh4_pg.C_LG_VIII001390	grail3.0175003501	2.28	1.3
2.1.1.14	estExt_fgenesh4_pg.C_LG_XIII0289	estExt_Genewise1_v1.C_LG_XIX1125	1.88	1.68
2.1.1.37	fgenesh4_pm.C_LG_IV000376	gw1.XIX.153.1	0.04	0.21
2.1.1.41	estExt_fgenesh4_pm.C_LG_I0739	gw1.IX.3368.1	0.03	0.21
2.1.1.76	estExt_Genewise1_v1.C_LG_XIV1942	fgenesh4_pm.C_LG_II000840	3.24	2.1
	eugene3.00021675	fgenesh4_pm.C_LG_XI000417	0.41	2.37
	gw1.I.5581.1	gw1.XIV.1942.1	0.51	2.1
2.1.2.1	estExt_fgenesh4_pm.C_LG_X0942	gw1.VIII.2633.1	2.14	1.3
2.1.2.11	fgenesh4_pm.C_LG_XIV000150	gw1.II.2913.1	0.85	0.68
2.2.1.1	estExt_Genewise1_v1.C_LG_XIV0740	eugene3.00021349	0.11	0.27
2.2.1.2	grail3.0008017101	grail3.0047006401	3.56	1.52
2.3.1.-	estExt_fgenesh4_pg.C_LG_IV1464	gw1.IX.1159.1	2.55	1.64
	grail3.0001096401	grail3.0053004202	1.98	0.7
	gw1.V.2788.1	gw1.VII.1776.1	0.03	0.21
2.3.1.12	estExt_fgenesh4_pm.C_LG_VIII0502	gw1.X.260.1	0.04	0.26
2.3.1.39	estExt_fgenesh4_pg.C_LG_IX0832	gw1.I.2270.1	0.19	0.25
2.3.1.41	estExt_fgenesh4_pm.C_LG_III0476	gw1.I.8529.1	0.01	0.19
	gw1.III.1759.1	gw1.XVIII.628.1	1.53	0.84
2.3.1.50	estExt_fgenesh4_pm.C_LG_XII0379	estExt_Genewise1_v1.C_LG_XV2341	2.84	0.73

2.3.1.57	gw1.VIII.1065.1	gw1.X.2734.1	0.19	0.87
2.3.1.61	estExt_Genewise1_v1.C_LG_XI2803	grail3.0032001601	1.46	0.48
2.3.1.74	estExt_fgenesh4_pg.C_LG_I0450	grail3.0047019001	2.46	1.39
	eugene3.00041296	gw1.IX.1383.1	0.06	0.32
	eugene3.00100729	gw1.V.1049.1	3.93	1.22
2.4.1.-	estExt_fgenesh4_pm.C_LG_XIII0357	eugene3.00111083	2.28	2.16
	eugene3.00041059	eugene3.00170460	0.01	0.25
	gw1.II.1063.1	gw1.XIV.923.1	0.02	0.18
	gw1.VI.1825.1	gw1.XVI.2407.1	0.02	0.19
	gw1.XIII.1801.1	gw1.XIX.735.1	0.02	0.22
2.4.1.11	estExt_fgenesh4_pg.C_LG_I3141	eugene3.00111290	2.58	1.08
2.4.1.117	estExt_fgenesh4_pg.C_LG_VIII0457	estExt_fgenesh4_pg.C_LG_X1846	2.54	1
2.4.1.14	eugene3.00181112	fgenesh4_pg.C_LG_XVIII000433	0.15	1.25
2.4.1.25	estExt_fgenesh4_pg.C_LG_VI0252	gw1.XVI.1067.1	0.03	0.18
2.4.1.34	estExt_fgenesh4_pg.C_LG_I0109	estExt_fgenesh4_pg.C_LG_III1876	0.05	0.24
	fgenesh4_pg.C_LG_II000551	gw1.V.662.1	0.05	0.26
2.4.1.69	eugene3.00010212	gw1.III.2370.1	0.29	0.62
	eugene3.00031740	gw1.I.6923.1	0.07	0.29
	gw1.I.6932.1	gw1.III.2367.1	0.28	1.01
2.4.1.91	eugene3.00011560	gw1.X.1927.1	0.88	2.25
	eugene3.00060078	eugene3.00160115	0.49	0
	eugene3.00060085	eugene3.00160109	0.39	0
	eugene3.00070068	gw1.IX.2389.1	1.07	1.73
	fgenesh4_pg.C_LG_IV001159	fgenesh4_pm.C_LG_XVII000147	0.11	0.19
	gw1.I.3549.1	gw1.XVII.405.1	0.23	1.35
2.4.2.14	gw1.IV.836.1	gw1.IX.1498.1	0.01	0.24
2.4.2.18	estExt_Genewise1_v1.C_LG_XII1779	fgenesh4_pg.C_LG_XV000271	0.05	0.17
	fgenesh4_pg.C_LG_X002040	fgenesh4_pm.C_LG_VIII000119	0.04	0.31
	fgenesh4_pm.C_LG_II000697	gw1.XIV.883.1	0.03	0.22
	gw1.XII.1858.1	gw1.XV.1584.1	0.61	0.79
2.4.2.19	estExt_fgenesh4_pm.C_LG_VIII0550	gw1.X.6595.1	0.58	0.42
2.4.99.4	gw1.IV.1517.1	gw1.IX.834.1	0.03	0.22
2.5.1.16	gw1.VIII.1889.1	gw1.X.5941.1	0.02	0.32
2.5.1.18	estExt_fgenesh4_pg.C_LG_III1026	gw1.I.9007.1	0.16	0.38
	estExt_fgenesh4_pg.C_LG_XI1201	fgenesh4_pg.C_LG_I003091	2.36	0.83
	estExt_fgenesh4_pm.C_LG_XI0374	eugene3.00012834	3.27	0.86
	eugene3.00002586	fgenesh4_pg.C_LG_X000434	0.35	0.6
	eugene3.00060229	eugene3.00160180	1.9	1.49

	fgenesh4_pg.C_LG_VIII001547	gw1.X.4762.1	0.27	1.71
2.5.1.21	estExt_fgenesh4_pg.C_LG_IV1345	estExt_fgenesh4_pm.C_LG_IX0217	2.63	0.88
2.5.1.8	gw1.VIII.295.1	gw1.X.4133.1	0.03	0.22
	gw1.VIII.574.1	gw1.X.185.1	0.04	0.35
2.6.1.1	estExt_fgenesh4_pg.C_LG_VII0526	fgenesh4_pg.C_LG_V000190	0.03	0.25
	estExt_fgenesh4_pm.C_LG_XVIII0158	grail3.0013044701	2.92	1.34
	gw1.VI.2755.1	gw1.XVI.226.1	0.65	0.57
2.6.1.2	estExt_fgenesh4_pm.C_LG_X0229	eugene3.00081764	1.4	1.1
2.6.1.44	estExt_fgenesh4_pm.C_LG_XVI0476	gw1.VI.969.1	0.03	0.23
	gw1.V.3011.1	gw1.VII.1999.1	0.95	0.87
2.6.1.45	estExt_fgenesh4_pg.C_LG_I1607	estExt_Genewise1_v1.C_LG_IX3694	2.12	2.02
2.7.1.-	estExt_fgenesh4_pg.C_LG_I0469	eugene3.00031446	0.05	0.22
	estExt_fgenesh4_pg.C_LG_II0333	estExt_Genewise1_v1.C_LG_X4439	0.27	1.33
	estExt_fgenesh4_pg.C_LG_II1481	fgenesh4_pm.C_LG_I000354	0.11	0.91
	estExt_fgenesh4_pg.C_LG_III1037	eugene3.00021475	2.24	2.15
	estExt_fgenesh4_pg.C_LG_III1285	fgenesh4_pm.C_LG_I000244	0.06	0.27
	estExt_fgenesh4_pg.C_LG_III1296	fgenesh4_pg.C_LG_I000643	0.03	0.21
2.7.1.1	fgenesh4_pg.C_LG_IX001086	fgenesh4_pm.C_LG_I000617	0.04	0.22
2.7.1.107	estExt_fgenesh4_pg.C_LG_VI1310	grail3.0020019502	2.91	0.86
	estExt_Genewise1_v1.C_LG_V2649	estExt_Genewise1_v1.C_LG_VII1667	0.09	0.26
2.7.1.137	estExt_fgenesh4_pg.C_LG_X1515	estExt_fgenesh4_pm.C_LG_VIII0340	0.07	0.22
	eugene3.00031835	fgenesh4_pm.C_LG_I000017	0.02	0.31
	fgenesh4_pm.C_LG_VI000364	gw1.XVI.3855.1	0.02	0.19
	gw1.I.4198.1	gw1.IX.2641.1	1.06	0.85
2.7.1.21	gw1.II.3111.1	gw1.VI.2277.1	0.14	1.59
2.7.1.25	estExt_fgenesh4_pm.C_LG_IX0365	gw1.I.4122.1	0.05	0.19
2.7.1.4	eugene3.00121190	eugene3.00151131	0.04	0.22
	fgenesh4_pg.C_LG_II000395	gw1.V.1131.1	0.05	0.23
	fgenesh4_pm.C_LG_IV000247	gw1.II.2365.1	0.05	0.24
	estExt_fgenesh4_pg.C_LG_XVIII0751	eugene3.00121190	0.06	0.27
2.7.1.40	estExt_fgenesh4_pg.C_LG_III1798	fgenesh4_pm.C_LG_I000007	3.22	2.55
	estExt_fgenesh4_pg.C_LG_X0733	estExt_fgenesh4_pm.C_LG_VIII0682	0.15	0.32
	fgenesh4_pg.C_LG_XIX000384	fgenesh4_pm.C_LG_XIII000262	0.03	0.22
	gw1.I.4193.1	gw1.IX.2629.1	0.03	0.24
2.7.1.6	estExt_fgenesh4_pg.C_LG_VIII0903	estExt_Genewise1_v1.C_LG_X0656	0.02	0.18
2.7.1.67	eugene3.00041158	eugene3.00090539	0.03	0.21
2.7.1.68	eugene3.00011188	gw1.III.1211.1	1.56	1.36

	fgenesh4_pg.C_LG_VIII001149	gw1.X.6602.1	0.07	0.22
	gw1.II.1775.1	gw1.V.5135.1	0.03	0.29
2.7.1.71	estExt_fgenesh4_pg.C_LG_VII0602	fgenesh4_pg.C_LG_II000575	2.53	1.14
2.7.1.90	eugene3.00020029	fgenesh4_pg.C_LG_V001718	0.04	0.22
2.7.1.91	estExt_fgenesh4_pg.C_LG_VIII0005	estExt_Genewise1_v1.C_LG_X3002	2.24	1.23
2.7.2.3	estExt_fgenesh4_pm.C_LG_VIII0335	estExt_fgenesh4_pm.C_LG_X0655	2.22	1.41
	eugene3.00080776	grail3.0154005402	1.06	0.49
2.7.2.4	gw1.II.423.1	gw1.XIV.3082.1	0.03	0.24
2.7.4.3	estExt_fgenesh4_pg.C_LG_I2284	estExt_Genewise1_v1.C_LG_XV2037	3.64	0.86
	estExt_fgenesh4_pg.C_LG_XII0928	estExt_fgenesh4_pm.C_LG_XV0275	2.38	1.8
	gw1.VI.634.1	gw1.XVIII.3392.1	0.1	0.12
	gw1.VIII.2309.1	gw1.X.2474.1	0.04	0.18
2.7.4.6	eugene3.00031128	grail3.0008036902	2.47	1.4
2.7.4.8	gw1.VIII.102.1	gw1.X.4176.1	0.02	0.27
2.7.7.15	gw1.VI.1940.1	gw1.XVI.788.1	0.02	0.3
2.7.7.22	estExt_fgenesh4_pg.C_LG_VI0713	grail3.0022021203	1.76	0.61
2.7.7.27	estExt_Genewise1_v1.C_LG_IX1717	fgenesh4_pg.C_LG_IV001321	0.04	0.25
2.7.7.4	estExt_fgenesh4_pg.C_LG_V1509	fgenesh4_pg.C_LG_II000227	0.03	0.25
	estExt_Genewise1_v1.C_LG_VIII2439	grail3.0175000802	2.97	2.69
2.7.7.6	estExt_Genewise1_v1.C_LG_XVIII1865	fgenesh4_pg.C_LG_VI001900	1.3	0.2
	eugene3.00031677	fgenesh4_pg.C_LG_I000263	0.04	0.28
	eugene3.00100701	fgenesh4_pg.C_LG_VIII001443	0.02	0.14
	fgenesh4_pg.C_LG_VIII001003	fgenesh4_pg.C_LG_X001243	0.03	0.19
	fgenesh4_pm.C_LG_VI000552	gw1.XVIII.3123.1	0.03	0.16
2.7.7.7	estExt_Genewise1_v1.C_LG_I1046	estExt_Genewise1_v1.C_LG_IX3930	3.42	1.12
	estExt_Genewise1_v1.C_LG_VIII2373	gw1.X.4958.1	0.01	0.2
	fgenesh4_pg.C_LG_XII000894	gw1.XV.1878.1	0.08	0.21
	gw1.V.3062.1	gw1.VII.2124.1	8.07E-03	0.17
2.7.7.8	eugene3.00031096	fgenesh4_pm.C_LG_I000327	0.01	0.4
	gw1.XIII.2192.1	gw1.XIX.1195.1	0.03	0.21
2.7.7.9	estExt_fgenesh4_pg.C_LG_I1954	estExt_fgenesh4_pg.C_LG_IX0932	0.03	0.17
2.7.8.11	grail3.0001027901	gw1.IV.1202.1	2.13	1.54
2.7.8.15	gw1.VI.2283.1	gw1.XVI.1421.1	0.01	0.22
2.7.8.5	estExt_fgenesh4_pm.C_LG_VIII0154	gw1.X.2456.1	0.05	0.16

2.8.2.-	eugene3.00031757	gw1.I.6873.1	4.42	0.96
	gw1.I.6866.1	gw1.III.627.1	0.72	0.82
	gw1.IV.2378.1	gw1.XI.1965.1	1.91	1.01
3.1.-.-	estExt_fgenesh4_pg.C_LG_VI0807	grail3.0010000701	3.29	1.42
	estExt_Genewise1_v1.C_LG_XVI3628	grail3.0022013602	2.06	1.69
3.1.1.-	estExt_fgenesh4_pm.C_LG_XIII0124	estExt_Genewise1_v1.C_LG_XIV2782	2.24	2.03
3.1.1.11	estExt_fgenesh4_pg.C_LG_XI0229	gw1.XI.436.1	1.86	1.12
	estExt_Genewise1_v1.C_LG_VII1401	fgenesh4_pg.C_LG_V000014	0.1	0.41
	eugene3.00011207	eugene3.00030085	0.09	0.29
	eugene3.00021342	eugene3.00121247	0.36	1.84
	eugene3.00030089	fgenesh4_pg.C_LG_I001266	0.04	0.29
	eugene3.00080087	eugene3.00102398	0.02	0.15
3.1.1.23	eugene3.00030304	fgenesh4_pm.C_LG_I000456	4.16	1.43
	eugene3.00102057	fgenesh4_pg.C_LG_VIII000401	0.06	0.21
	fgenesh4_pg.C_LG_X002025	gw1.VIII.973.1	0.06	0.4
	gw1.II.2920.1	gw1.XIV.2533.1	0.19	0.37
	gw1.VI.1970.1	gw1.XVIII.1123.1	0.04	0.24
3.1.1.3	estExt_fgenesh4_pg.C_LG_IX1026	gw1.I.1579.1	0.05	0.23
3.1.1.31	eugene3.00150084	fgenesh4_pg.C_LG_XII000285	5.05	1.14
3.1.1.4	estExt_fgenesh4_pg.C_LG_V0735	eugene3.00020952	2.46	1.47
	estExt_fgenesh4_pm.C_LG_II0445	grail3.0002001503	3.38	1.07
	estExt_Genewise1_v1.C_LG_II4211	grail3.0083005001	2.03	1.17
3.1.1.5	estExt_fgenesh4_pg.C_LG_III0323	grail3.0084003101	2.26	1.18
	estExt_fgenesh4_pg.C_LG_VI1766	fgenesh4_pm.C_LG_XVIII000124	0.03	0.16
3.1.2.22	fgenesh4_pg.C_LG_II001248	gw1.III.895.1	0.23	1.45
3.1.3.-	estExt_fgenesh4_pg.C_LG_VI1095	eugene3.00160551	0.62	0.48
	gw1.VIII.1527.1	gw1.X.2995.1	1.26	1.31
3.1.3.12	estExt_Genewise1_v1.C_LG_XV2838	eugene3.00121254	0.07	0.28
	fgenesh4_pg.C_LG_V000859	grail3.0011016001	2.11	1.03
	gw1.XII.661.1	gw1.XV.542.1	0.05	0.37
3.1.3.18	estExt_fgenesh4_pg.C_LG_VI0678	estExt_fgenesh4_pm.C_LG_IX0598	0.29	1.22
	eugene3.00081423	grail3.0006002201	2.07	1.01
3.1.3.2	estExt_fgenesh4_pg.C_LG_II0269	estExt_fgenesh4_pg.C_LG_IX0440	0.16	0.77
	estExt_fgenesh4_pg.C_LG_V1468	estExt_fgenesh4_pm.C_LG_IV0474	0.23	1.03
	estExt_fgenesh4_pg.C_LG_XIII0422	fgenesh4_pm.C_LG_XI000369	0.07	0.24
	estExt_fgenesh4_pg.C_LG_XVIII0431	gw1.XVIII.27.1	2.62	1.78
	estExt_fgenesh4_pm.C_LG_X0716	fgenesh4_pg.C_LG_VIII000629	0.07	0.23
	fgenesh4_pm.C_LG_XII000013	gw1.VIII.852.1	0.25	2.62
3.1.3.37	estExt_fgenesh4_pg.C_LG_VIII0539	eugene3.00101874	2.78	1.98

3.1.3.4	gw1.II.351.1	gw1.IV.3349.1	0.08	0.21
3.1.3.41	estExt_fgenesh4_pg.C_LG_VIII0664	gw1.X.1500.1	9.48E-03	0.18
3.1.3.5	eugene3.00002658	eugene3.00141466	0.2	0.15
3.1.3.7	estExt_fgenesh4_pg.C_LG_IV0248	eugene3.00110970	5.26	1.17
3.1.4.11	estExt_fgenesh4_pg.C_LG_I1618	fgenesh4_pm.C_LG_VIII000270	0.23	1.48
	eugene3.00080618	fgenesh4_pg.C_LG_I001629	2.32	1.96
	eugene3.00101828	gw1.IX.3737.1	3.66	1.9
3.1.4.4	estExt_Genewise1_v1.C_LG_XIV0962	fgenesh4_pg.C_LG_II001399	0.04	0.15
	eugene3.00020142	eugene3.00051506	0.04	0.28
	eugene3.00181173	fgenesh4_pg.C_LG_VI001806	0.25	1.79
3.1.4.46	gw1.VI.2197.1	gw1.XVIII.3294.1	0.04	0.23
3.2.1.-	eugene3.00060581	gw1.XVIII.258.1	0.04	0.17
	eugene3.00150506	fgenesh4_pm.C_LG_XII000271	0.02	0.19
3.2.1.114	eugene3.00011928	gw1.XVII.756.1	0.3	0.29
3.2.1.15	estExt_Genewise1_v1.C_LG_X5311	gw1.VIII.357.1	2.46	2.26
	fgenesh4_pg.C_LG_I003312	gw1.XI.1147.1	0.04	0.25
	gw1.I.9423.1	gw1.XVIII.1568.1	0.61	1.92
3.2.1.18	eugene3.00120811	eugene3.00150592	0.14	0.26
	fgenesh4_pg.C_LG_VIII000253	fgenesh4_pg.C_LG_X002082	0.2	0.34
3.2.1.2	estExt_fgenesh4_pm.C_LG_VIII0731	gw1.X.4692.1	0.04	0.21
	gw1.I.8484.1	gw1.III.2008.1	0.05	0.24
3.2.1.20	eugene3.00081119	gw1.X.243.1	0.04	0.28
	eugene3.00111310	fgenesh4_pg.C_LG_I003114	0.13	0.64
3.2.1.21	estExt_fgenesh4_pg.C_LG_XIII0259	estExt_fgenesh4_pg.C_LG_XIX0430	0.02	0.24
	estExt_fgenesh4_pm.C_LG_X0551	fgenesh4_pg.C_LG_II001817	0.25	0.75
	eugene3.00150368	gw1.XII.1817.1	0.51	0.5
	fgenesh4_pg.C_LG_II000867	fgenesh4_pm.C_LG_V000313	0.2	0.92
	fgenesh4_pm.C_LG_III000026	gw1.I.26.1	0.04	0.3
	gw1.I.4104.1	gw1.IX.2546.1	1.73	0.97
3.2.1.23	estExt_fgenesh4_pm.C_LG_IV0471	estExt_Genewise1_v1.C_LG_VII3760	3.28	0.95
	fgenesh4_pg.C_LG_IX000330	gw1.IV.1165.1	0.05	0.24
	fgenesh4_pm.C_LG_II000364	fgenesh4_pm.C_LG_VII000166	0.48	2.27
	gw1.V.2620.1	gw1.V.5394.1	0.24	1.26
3.2.1.28	estExt_fgenesh4_pg.C_LG_I0760	fgenesh4_pg.C_LG_III001208	0.09	0.24
3.2.1.39	estExt_fgenesh4_pg.C_LG_XVIII1099	gw1.VI.1360.1	0.05	0.19
	eugene3.00002053	eugene3.00141056	4.45	1.72
	eugene3.00002262	gw1.IV.3627.1	0.04	0.21
	eugene3.00010060	grail3.0136005601	3.49	1.45

	eugene3.00020825	gw1.V.5112.1	0.34	1.32
	eugene3.00080499	gw1.I.1293.1	0.28	1.81
3.2.1.4	estExt_fgenesh4_pg.C_LG_II1746	estExt_fgenesh4_pg.C_LG_XIV0665	0.04	0.23
	estExt_fgenesh4_pm.C_LG_II0108	estExt_fgenesh4_pm.C_LG_V0631	0.09	0.21
	estExt_fgenesh4_pm.C_LG_III0465	fgenesh4_pg.C_LG_I000818	0.03	0.26
	fgenesh4_pg.C_LG_IV001366	gw1.IX.1559.1	0.03	0.41
	fgenesh4_pm.C_LG_I000271	gw1.III.1493.1	0.97	1.1
3.2.1.58	estExt_Genewise1_v1.C_LG_VII1905	gw1.V.2919.1	0.08	0.33
3.2.1.67	eugene3.00010865	grail3.0039009101	0.19	1.32
	eugene3.00081775	gw1.X.5283.1	0.03	0.21
	fgenesh4_pg.C_LG_II001877	gw1.VIII.2124.1	0.3	2.65
	fgenesh4_pg.C_LG_II001910	gw1.XIV.2085.1	0.04	0.17
	gw1.X.736.1	gw1.XIV.2207.1	1.23	1.58
3.2.1.84	gw1.V.2605.1	gw1.VII.1603.1	0.03	0.33
3.3.2.6	estExt_fgenesh4_pm.C_LG_VIII0150	eugene3.00102094	0.04	0.27
3.4.-.-	estExt_fgenesh4_pg.C_LG_III0301	estExt_fgenesh4_pm.C_LG_I0435	2.79	1.24
	estExt_fgenesh4_pg.C_LG_VI1605	estExt_Genewise1_v1.C_LG_XVIII0768	2.68	1.13
3.4.11.2	estExt_fgenesh4_pm.C_LG_III0416	eugene3.00010884	0.06	0.24
3.4.11.5	eugene3.00021531	fgenesh4_pg.C_LG_XIV000341	0.05	0.2
3.5.1.-	fgenesh4_pg.C_LG_VI001354	gw1.XVI.2349.1	0.22	1.23
	gw1.II.3341.1	gw1.V.5337.1	0.03	0.22
	gw1.XII.682.1	gw1.XV.125.1	0.06	0.27
3.5.3.1	eugene3.00021348	eugene3.00140133	3.9	1.47
3.5.4.12	eugene3.00120910	eugene3.00150755	3.04	1.33
3.5.4.25	fgenesh4_pm.C_LG_XVII000028	gw1.I.3631.1	0.02	0.23
	gw1.V.2687.1	gw1.VII.1682.1	1.55	1.1
3.5.5.1	fgenesh4_pg.C_LG_XVI000624	gw1.VI.1794.1	0.08	0.22
3.6.1.-	fgenesh4_pg.C_LG_XII000954	fgenesh4_pg.C_LG_XV000704	0.03	0.24
3.6.1.15	estExt_Genewise1_v1.C_LG_XIII1902	fgenesh4_pm.C_LG_XIX000083	0.06	0.23
3.6.1.5	estExt_fgenesh4_pm.C_LG_X0646	fgenesh4_pg.C_LG_VIII000753	0.07	0.17
4.1.1.1	estExt_fgenesh4_pg.C_LG_IV0428	gw1.XI.3391.1	0.03	0.25
	estExt_fgenesh4_pm.C_LG_XVI0442	eugene3.00060891	4.76	1.45
4.1.1.15	estExt_fgenesh4_pm.C_LG_VIII0733	eugene3.00002589	0.14	1.27
	eugene3.00120058	grail3.0038018902	2.72	1.48
	fgenesh4_pg.C_LG_V000294	gw1.VII.2372.1	0.03	0.18
	fgenesh4_pg.C_LG_VIII001255	gw1.X.6258.1	0.03	0.29

4.1.1.20	estExt_fgenesh4_pg.C_LG_IV1314	eugene3.00090483	0.03	0.2
4.1.1.33	grail3.0090014401	grail3.0106013901	2.34	1.06
4.1.1.50	eugene3.00101272	gw1.IV.4194.1	0.2	0.89
	gw1.VIII.2435.1	gw1.X.4229.1	0.04	0.25
4.1.2.13	estExt_fgenesh4_pm.C_LG_IX0211	estExt_Genewise1_v1.C_LG_IV0774	3.09	1.64
4.1.3.27	eugene3.00100957	gw1.VIII.845.1	0.04	0.24
	gw1.IX.3826.1	gw1.X.1797.1	0.13	1.28
4.2.1.17	estExt_fgenesh4_pg.C_LG_VIII0916	grail3.0006050201	2.21	1.34
4.2.1.19	gw1.VIII.669.1	gw1.X.5883.1	0.21	0.45
4.2.1.20	estExt_fgenesh4_pg.C_LG_II0422	estExt_fgenesh4_pg.C_LG_V1322	1.28	0.79
	fgenesh4_pg.C_LG_XIII000422	gw1.XI.1757.1	0.25	2.23
4.2.1.3	estExt_fgenesh4_pg.C_LG_II2062	gw1.XIV.3318.1	0.03	0.18
4.2.1.46	estExt_fgenesh4_pg.C_LG_X1860	estExt_fgenesh4_pm.C_LG_VIII0190	2	1.44
4.2.1.51	estExt_fgenesh4_pg.C_LG_IV0012	estExt_fgenesh4_pg.C_LG_XI0043	3.59	0.9
4.2.1.52	estExt_fgenesh4_pg.C_LG_II1351	grail3.0035005702	1.22	0.51
4.2.1.70	estExt_fgenesh4_pg.C_LG_II0130	gw1.V.1974.1	0.05	0.35
	gw1.VI.2308.1	gw1.XVI.1402.1	0.06	0.33
4.2.1.9	estExt_fgenesh4_pg.C_LG_I0451	estExt_fgenesh4_pm.C_LG_III0598	0.08	0.29
4.2.1.92	eugene3.00090572	fgenesh4_pg.C_LG_IV001237	0.14	0.51
4.2.2.2	estExt_fgenesh4_pg.C_LG_I2215	eugene3.00081702	0.21	1.61
	estExt_fgenesh4_pm.C_LG_VI0607	estExt_Genewise1_v1.C_LG_VIII1759	0.24	1.62
	estExt_Genewise1_v1.C_LG_III0932	gw1.II.376.1	0.12	0.67
	eugene3.00100518	eugene3.00120841	1.8	1.3
	fgenesh4_pg.C_LG_I000068	fgenesh4_pg.C_LG_III001856	0.06	0.41
	fgenesh4_pg.C_LG_IV001141	gw1.XVII.937.1	0.07	0.35
4.3.2.2	gw1.VIII.877.1	gw1.X.1884.1	2.58	1.16
4.3.3.2	eugene3.00150409	gw1.XII.136.1	4.68	0.54
4.4.1.14	estExt_fgenesh4_pg.C_LG_I0872	estExt_fgenesh4_pm.C_LG_II0741	0.21	1.96
	eugene3.00140049	fgenesh4_pg.C_LG_II001052	0.21	2.12
4.4.1.16	eugene3.00130020	eugene3.00140573	0.31	1.13
4.99.1.1	gw1.VI.1271.1	gw1.XVIII.848.1	1.12	1.03
5.1.1.7	estExt_Genewise1_v1.C_LG_VI1708	gw1.XVI.3802.1	0.04	0.17

5.1.3.-	eugene3.00061339	eugene3.00180906	1.02	0.68
	grail3.0018019001	grail3.0033029301	0.21	1.34
5.1.3.2	estExt_fgenesh4_pg.C_LG_VI0193	eugene3.00160140	0.03	0.27
	estExt_fgenesh4_pg.C_LG_XI1340	eugene3.00012996	2.81	1
	fgenesh4_pg.C_LG_I000802	grail3.0018042701	0.04	0.19
5.3.1.1	estExt_fgenesh4_pg.C_LG_I1744	eugene3.00091331	2.59	1.02
	estExt_Genewise1_v1.C_LG_IV0988	estExt_Genewise1_v1.C_LG_IX1362	2.53	1.3
	estExt_Genewise1_v1.C_LG_X2172	grail3.0049021504	0.19	0.23
5.3.1.16	gw1.II.4025.1	gw1.IV.3492.1	0.03	0.25
5.3.1.4	estExt_fgenesh4_pm.C_LG_IV0065	fgenesh4_pg.C_LG_XI000339	0.04	0.2
	estExt_fgenesh4_pm.C_LG_VI0047	gw1.XVI.673.1	0.05	0.34
	estExt_Genewise1_v1.C_LG_X5925	gw1.VIII.813.1	2.25	1.39
	eugene3.00020052	eugene3.00051602	0.03	0.14
	fgenesh4_pg.C_LG_IX000516	gw1.VII.3437.1	0.31	2.56
	fgenesh4_pm.C_LG_IV000088	fgenesh4_pm.C_LG_XIII000421	0.19	1.2
5.3.99.5	gw1.VI.124.1	gw1.XVIII.665.1	0.04	0.25
5.4.2.1	eugene3.00050750	gw1.II.1557.1	1.67	1.26
5.4.2.2	estExt_fgenesh4_pg.C_LG_XV1054	eugene3.00121193	0.1	0.25
5.4.3.8	eugene3.00150799	gw1.XII.1787.1	2.2	0.86
5.4.99.5	gw1.VIII.1802.1	gw1.X.927.1	0.57	0.6
5.5.1.4	estExt_fgenesh4_pm.C_LG_V0078	estExt_fgenesh4_pm.C_LG_VII0202	1.97	1.25
6.-.-.-	estExt_fgenesh4_pm.C_LG_XV0458	estExt_Genewise1_v1.C_LG_I8988	2.44	1.69
6.1.1.1	fgenesh4_pm.C_LG_VIII000033	gw1.X.3221.1	0.01	0.32
6.1.1.12	eugene3.00090812	fgenesh4_pm.C_LG_I001093	0.03	0.2
6.1.1.18	fgenesh4_pg.C_LG_VIII001094	gw1.X.163.1	0.12	0.31
6.1.1.20	estExt_fgenesh4_pg.C_LG_IX0484	gw1.IV.516.1	2.25	1.13
6.1.1.9	fgenesh4_pg.C_LG_II002297	gw1.IV.3484.1	0.05	0.24
6.2.1.12	estExt_fgenesh4_pg.C_LG_XV0666	fgenesh4_pg.C_LG_X000472	0.42	1.72
	estExt_fgenesh4_pg.C_LG_XVI0255	fgenesh4_pg.C_LG_VI000295	2.58	1.56
	eugene3.00020113	fgenesh4_pg.C_LG_V001627	0.05	0.25
	fgenesh4_pg.C_LG_III000782	grail3.0099003002	2.02	1.65
	fgenesh4_pg.C_LG_III001773	grail3.0100002702	3.49	1.77
	fgenesh4_pm.C_LG_I000177	gw1.III.2054.1	1.66	1.02
6.2.1.3	estExt_fgenesh4_pg.C_LG_III1161	estExt_Genewise1_v1.C_LG_I8598	2.47	1.1
	fgenesh4_pg.C_LG_V001648	gw1.II.2059.1	1.93	0.84

	gw1.III.703.1	gw1.V.3702.1	0.34	0.49
6.3.1.2	estExt_fgenesh4_pg.C_LG_VII0739	estExt_Genewise1_v1.C_LG_V3325	0.14	0.45
6.3.2.-	eugene3.00080619	fgenesh4_pg.C_LG_X001699	0.51	0.29
6.3.2.17	eugene3.00101917	gw1.VIII.881.1	2.63	1.05
6.3.2.2	estExt_fgenesh4_pm.C_LG_III0405	estExt_Genewise1_v1.C_LG_I8937	2.13	1.69
6.3.3.1	fgenesh4_pm.C_LG_XIV000303	gw1.II.2846.1	0.02	0.17
6.3.4.2	estExt_Genewise1_v1.C_LG_III0729	gw1.I.8101.1	0.02	0.2
	fgenesh4_pm.C_LG_VIII000317	gw1.X.1387.1	0.02	0.19
6.3.5.3	eugene3.00080522	gw1.X.2075.1	0.09	0.2
6.3.5.4	estExt_fgenesh4_pm.C_LG_I0800	estExt_Genewise1_v1.C_LG_IX3026	2.67	1.5
