# Supplementary figures and images for: Genes encoding hub and bottleneck enzymes of the Arabidopsis metabolic network preferentially retain homeologs through whole genome duplication
Source: BMC Evol Biol. 2010 May 18;10:145. doi: 10.1186/1471-2148-10-145 (PMC2880986; doi:10.1186/1471-2148-10-145)

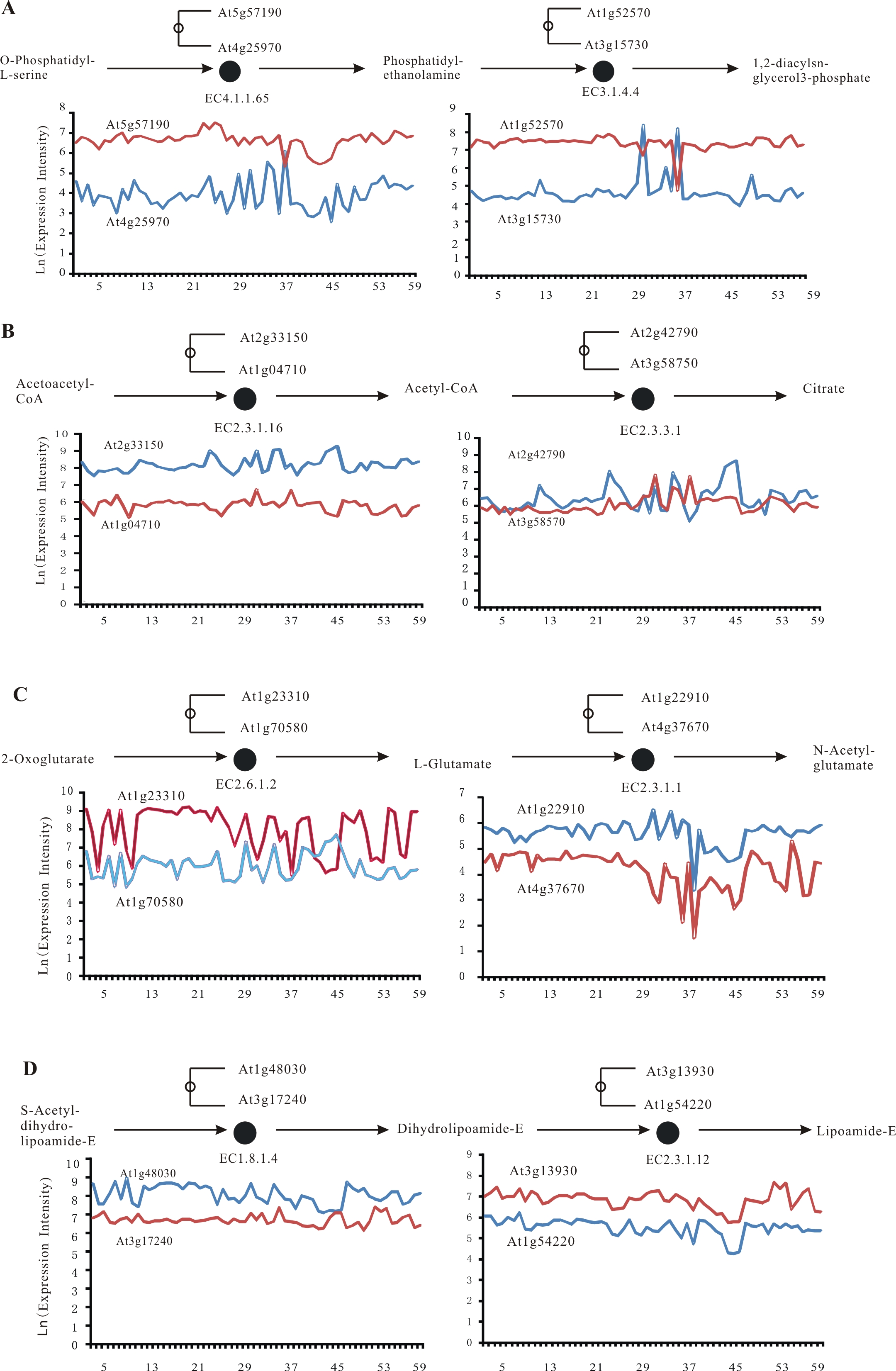

Supplement: Additional file 10 — Figure S2. The expression profiles of four connected enzyme-homeologs in the 59 developmental stages. [file 1471-2148-10-145-S10.JPEG]
